# Coinhibition of the MEK/RTK pathway has high therapeutic efficacy in KRAS-mutant non-small cell lung cancer

**DOI:** 10.1038/s41392-025-02382-w

**Published:** 2025-09-12

**Authors:** Jun Lu, Minjuan Hu, Yikai Zhao, Tianqing Chu, Wei Zhang, Yijia Zhou, Xinlei Cai, Jun Wu, Liang Hu, Chunlei Shi, Liwen Xiong, Aiqin Gu, Huimin Wang, Yanwei Zhang, Yuqing Lou, Runbo Zhong, Zhiqiang Gao, Hongyu Liu, Chao Zhou, Yingli Wu, Liang Zhu, Hua Zhong, Hongbin Ji, Baohui Han

**Affiliations:** 1 Department of Respiratory and Critical Care Medicine, Shanghai Chest Hospital, Shanghai Jiao Tong University School of Medicine, Shanghai, China; 2 Shanghai Institute of Thoracic Oncology, Shanghai Chest Hospital, Shanghai Jiao Tong University School of Medicine, Shanghai, China; 3 Translational Medical Research Platform for Thoracic Oncology, Shanghai Chest Hospital, Shanghai Jiao Tong University School of Medicine, Shanghai, China; 4 Department of Biobank, Shanghai Chest Hospital, Shanghai Jiao Tong University School of Medicine, Shanghai, China; 5 State Key Laboratory of Cell Biology, Shanghai Institute of Biochemistry and Cell Biology, Center for Excellence in Molecular Cell Science, Chinese Academy of Sciences, Shanghai, China; 6 School of Life Science and Technology, ShanghaiTech University, Shanghai, China; 7 Key Laboratory of Systems Health Science of Zhejiang Province, School of Life Science, Hangzhou Institute for Advanced Study, University of Chinese Academy of Sciences, Hangzhou, China; 8 School of Life Science, East China Normal University, Shanghai, China; 9 Key Laboratory of Cell Differentiation and Apoptosis of the Chinese Ministry of Education, Shanghai Jiao Tong University School of Medicine, Shanghai, China; 10 Department of Pharmacology and Chemical Biology, Shanghai Jiao Tong University School of Medicine, Shanghai, China

**Keywords:** Lung cancer, Lung cancer

## Abstract

Oncogenic KRAS mutations are frequently detected in NSCLC. It remains a major challenge to target all KRAS mutants. MEK inhibitors are considered candidates for treating KRAS-mutant NSCLC; however, their easy adaptive resistance precludes further application. Here, we found that MEK inhibitor-trametinib treatment results in the feedback activation of multiple receptor tyrosine kinases (RTKs) and that treatment with the pan-RTK inhibitor anlotinib effectively inhibits the progression of KRAS-mutant NSCLC. Furthermore, we evaluated this strategy in a clinical study (NCT04967079) involving 33 advanced non-G12C KRAS-mutant NSCLC patients. The phase Ia containing 13 patients showed that the recommended phase 2 dose (RP2D) is trametinib (2 mg) plus anlotinib (8 mg), the objective response rate (ORR) is 69.2% (95% CI: 38.6-90.9), the median progression-free survival (PFS) is 6.9 months (95% CI: 3.9 to could not be evaluated), disease control rate (DCR) is 92% (95% CI: 64.0–99.8) and the rate of adverse events (AEs) ≥grade 3 is 23%. The phase Ib containing 20 patients demonstrated the high efficacy of this combinational therapy with RP2D, with the ORR at 65% (95% CI: 40.8–84.6), the median PFS is 11.5 months (95% CI: 8.3–15.5), the median overall survival (OS) is 15.5 months (95% CI: 15.5 to could not be evaluated), the DCR at 100% (95% CI: 83.2–100.0), the median duration of overall response (DoR) is 9.3 months (95% CI: 2.5–12.1), and the rate of AEs ≥ grade 3 at 35%. Overall, this study provides a potential combinational therapeutic strategy for KRAS-mutant NSCLC through the cotargeting of MEK and RTKs.

## Introduction

Non-small cell lung cancer (NSCLC) accounts for one of the most common malignancies.^1–3^ Oncogenic kirsten rat sarcoma 2 viral oncogene homolog (KRAS) mutation is a common driver gene for initiating and promoting NSCLC development, and its frequency ranges from 6% to 33%.^4–7^ This oncogenic mutation promotes the activation of downstream signaling pathways, including the mitogen-activated protein kinase/extracellular signal-regulated kinase (MEK/ERK) and phosphatidylinositol-3-kinase/protein kinase B (PI3K/AKT) cascades, by facilitating the conversion of RAS from its inactive guanosine 5’-diphosphate (GDP)-bound state to the active guanosine triphosphate (GTP)-bound form.^5,8–10^ Owing to the specificity of the RAS protein-binding domain, the oncogenic KRAS gene has been considered undruggable in recent decades.^9^ Nevertheless, a significant breakthrough occurred in 2021 with the introduction of the first inhibitor against KRAS^*G12C*^, sotorasib, which has demonstrated clinical effectiveness.^11^ Subsequently, several KRAS^*G12C*^ inhibitors, such as adagrasib and divarasib, have been developed successively.^12,13^ These agents targeting the KRAS^*G12C*^ mutation represent breakthrough therapeutic options for patients with KRAS-mutant NSCLC.

However, KRAS^*G12C*^ only accounts for 25% to 43% of all oncogenic KRAS mutations in NSCLC patients,^6,7,14^ and the remaining KRAS mutant subtypes (such as KRAS^*G12D*^, KRAS^*G12S*^, KRAS^*G12V*^, and KRAS^*Q61H*^) present distinct biological challenges. Unlike KRAS^*G12C*^, non-G12C KRAS mutant subtypes typically lack reactive cysteine residues and may exhibit different GTP-binding dynamics, making them less amenable to covalent inhibition and more difficult to target with small molecules.^15,16^ To date, effective targeted therapies for these non-G12C KRAS mutations remain elusive, highlighting a significant unmet clinical need.

MEK inhibitors are considered important candidates for treating KRAS-mutant NSCLC.^17–19^ This is attributed to their ability to block the KRAS downstream MEK-ERK signal,^20^ thus limiting the activation of KRAS signaling and impeding tumor development. However, the setbacks associated with MEK inhibitor-trametinib monotherapy as well as the combination of the MEK inhibitor selumetinib with chemotherapy in KRAS-mutant NSCLC patients have led to substantial limitations in overcoming this subtype of NSCLC through the use of MEK inhibitors.^17–19^ An investigation of the molecular mechanisms revealed that the limited therapeutic efficacy of MEK inhibitors is due to MEK inhibitor-induced ERK feedback activation and bypass activation (such as the PI3K-AKT-mammalian target of rapamycin (mTOR) pathway),^21,22^ leading to adaptive resistance in KRAS-mutant NSCLC cells. Moreover, studies have revealed that multiple signaling molecules (including fibroblast growth factor receptor 1 (FGFR1), src homology-2 protein tyrosine phosphatase (SHP2), and AXL receptor tyrosine kinase (AXL)) are involved in adaptive resistance to the MEK inhibitor trametinib.^21–25^ However, data demonstrating the high therapeutic effectiveness of MEK inhibitor-based combinational strategies for KRAS-mutant NSCLC patients in the clinic are lacking.^26^ Therefore, further understanding the underlying molecular mechanism of adaptive resistance to MEK inhibitors could contribute to the screening of key synergistic inhibitors to develop novel combination strategies. These findings provide a theoretical basis for the treatment of KRAS-mutant NSCLC patients in the clinic.

In this study, we further investigated the molecular mechanism of adaptive resistance to the MEK inhibitor trametinib in KRAS-mutant NSCLC cells and revealed that trametinib-induced multiple receptor tyrosine kinase (RTK) activation plays a key role in adaptive resistance, suggesting that coinhibition of the MEK/RTK pathways via trametinib plus anlotinib administration has potential value in the treatment of KRAS-mutant NSCLC in preclinical and clinical settings.

## Results

### Trametinib-induced short-term proliferation inhibition in KRAS-mutant NSCLC cells via inhibition of the MEK-ERK pathway

**Fig. 1:**
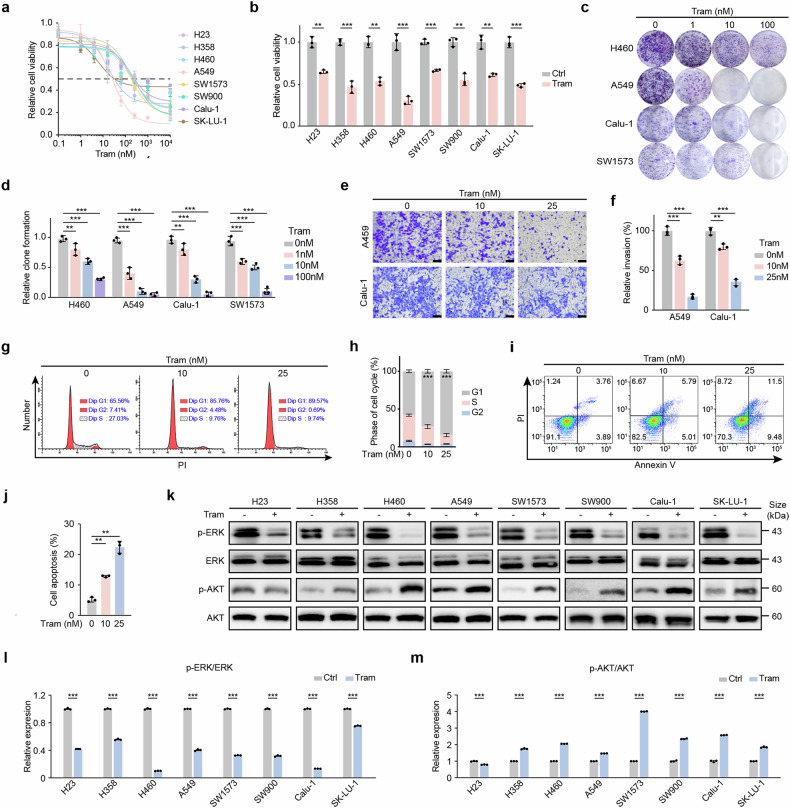
Mechanistic analyses of trametinib resistance in KRAS-mutant NSCLC. **a** Viability of human KRAS-mutant lung cancer cell lines (H23, H358, SW1573, Calu-1: KRAS^*G12C*^; H460: KRAS^*Q61H*^; A549: KRAS^*G12S*^; SW900: KRAS^*G12V*^; SK-LU-1: KRAS^*G12D*^) after 72 h of treatment with the indicated doses of trametinib. **b** Viability of human KRAS-mutant NSCLC cell lines after 72 h of trametinib treatment at 64 nM. **c**, **d** Colony formation (**c**) and statistical analyses (**d**) of H460, A549, Calu-1 and SW1573 cells after 10 days of treatment with the indicated doses of trametinib. **e**, **f** Boyden chamber transwell invasion (**e**) and statistical analyses (**f**) of A549 and Calu-1 cells after 24 h of treatment with the indicated doses of trametinib. Scale bar: 50 μm. **g**, **h** Cell cycle analyses of A549 cells after 24 h of treatment with the indicated doses of trametinib via PI staining (**g**) and quantification of the cell cycle phases (**h**). **i**, **j** Apoptosis analyses of A549 cells after 24 h of treatment with the indicated doses of trametinib via Annexin V and PI staining (**i**) and related statistical analyses (**j**). **k**‒**m** Western blot analyses (**k**) and related statistical analyses of the p-ERK/ERK ratio (**l**) and p-AKT/AKT ratio (**m**) in human KRAS-mutant NSCLC cell lines after 48 h of 10 nM trametinib treatment. All data comparisons were conducted via two-tailed Student’s t test. The data are presented as the means ± SEMs. *n* = 3, **P < 0.01, ***P < 0.001

We first treated the KRAS-mutant human NSCLC cell lines (A549, KRAS^*G12S*^; H460, KRAS^*Q61H*^; SK-LU-1, KRAS^*G12D*^; SW900, KRAS^*G12V*^; H23, KRAS^*G12C*^; H358, KRAS^*G12C*^; SW1573, KRAS^*G12C*^; and Calu-1, KRAS^*G12C*^) with trametinib for 3 days. Consistent with previous reports,^21,22^ trametinib significantly inhibited the growth of all the KRAS-mutant cell lines after short-term treatment (Fig. 1a, b). Notably, cell growth arrest, cell invasion inhibition, G1 phase cycle arrest and increased apoptosis were observed (Fig. 1c–j). These results suggest that trametinib has the ability to induce significant short-term cell toxicity in KRAS-mutant NSCLC cells. Furthermore, we also observed significant downregulation of ERK phosphorylation after trametinib administration to various KRAS-mutant NSCLC cells (Fig. 1k, l). However, AKT phosphorylation, which is indicative of downstream PI3K‒AKT pathway activation, was upregulated in most cell lines except H23 cells (Fig. 1k, m). On the basis of these results and previous reports,^21,22^ we concluded that trametinib-induced short-term proliferation inhibition in KRAS-mutant NSCLC cells was attributed mainly to the inhibition of the MEK-ERK pathway.

**Fig. 2:**
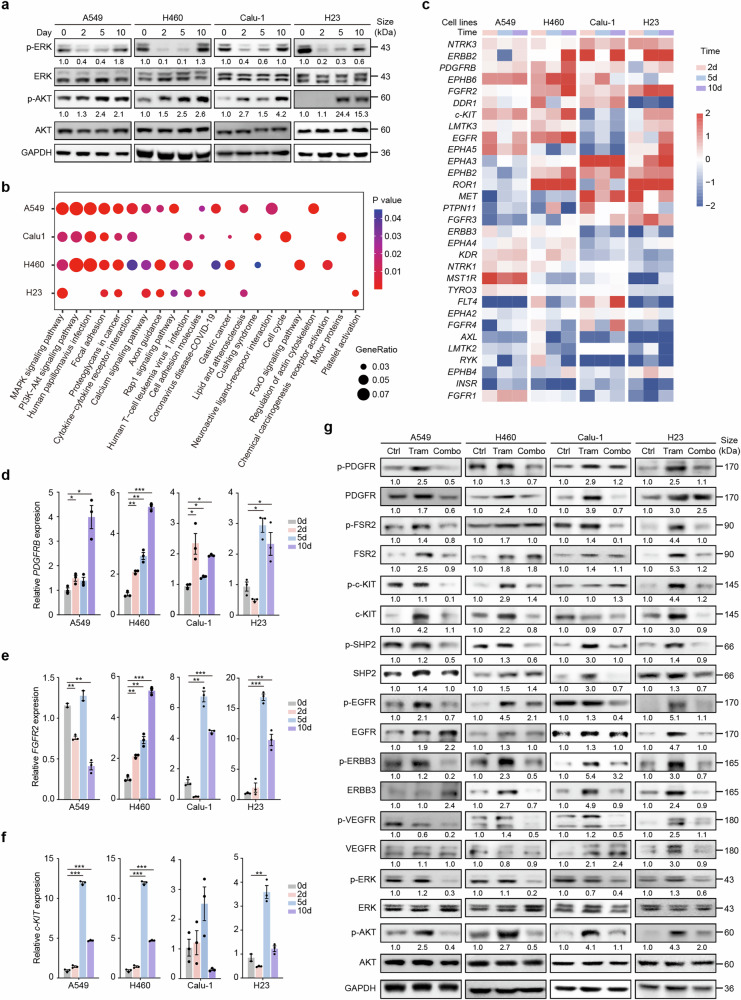
Adaptive resistance to trametinib in KRAS-mutant NSCLC cells is attributed to the activation of ERK/AKT signaling and RTK signaling. **a** A549 (KRAS^*G12S*^), Calu-1 (KRAS^*G12C*^), H23 (KRAS^*G12C*^), and H460 (KRAS^*Q61H*^) cells were treated with 10 nM trametinib for 0, 2, 5, or 10 days and subjected to immunoblotting with the indicated antibodies. **b** Bubble plot revealing the KEGG pathways enriched with the DEGs from the RNA-seq data of the A549, Calu1, H23, and H460 cell lines after trametinib treatment for 10 days. The area of each circle reflects the proportion of genes related to specific pathways among the DEGs. The color of the circles represents the range of *P* values. **c** RTK gene expression changes in A549, Calu1, H23, and H460 cells following treatment with trametinib for 2 days, 5 days, or 10 days relative to the 0-day control. **d**‒**f** Gene expression of c-Kit, FGFR2 and PDGFRB was detected via quantitative reverse transcription polymerase chain reaction (qRT‒PCR) in A549, Calu-1, H460 and H23 cells after treatment with 10 nM trametinib for 0, 2, 5, or 10 days. *n* = 3. **g** A549, Calu-1, H460 and H23 cells were treated with trametinib monotherapy (10 nM) or trametinib (10 nM) and anlotinib (1 μM) combination therapy for 10 days and subjected to immunoblotting with the indicated antibodies. Relative densitometric values are indicated below each band. All data comparisons were conducted via two-tailed Student’s t test. The data are presented as the means ± SEMs. *P < 0.05, **P < 0.01, ***P < 0.001

However, a drawback of single trametinib treatment is the induction of AKT phosphorylation, which is an important element contributing to maintaining NSCLC cell survival. These findings suggest that trametinib-induced short-term proliferation inhibition in KRAS-mutant NSCLC cells can be attenuated via the activation of AKT phosphorylation (Supplementary Fig. 1a). Interestingly, long-term trametinib treatment (10 days) resulted in a rebound in ERK phosphorylation and further upregulation of AKT phosphorylation, suggesting rapid adaptive resistance to MEK inhibitors in KRAS-mutant lung cancer (Fig. 2a). These phenomena suggest that the activation of underlying upstream signals could account for the rebound of ERK and the upregulation of AKT.

### Long-term trametinib treatment triggers feedback activation of RTKs in KRAS-mutant NSCLC

To further understand the underlying mechanisms, we performed transcriptomic analysis of 4 KRAS-mutant NSCLC cell lines (A549, Calu1, H460 and H23) treated with trametinib at several time points (Supplementary Fig. 1b, c). After 2 days of trametinib treatment in A549 cells, the top 1000 upregulated genes were enriched in pathways such as pathways related to cancer, the PI3K-AKT signaling pathway, the mitogen-activated protein kinase (MAPK) signaling pathway, and the apoptosis pathway; moreover, the top 1000 downregulated genes were enriched in pathways such as metabolic pathways, complement and coagulation cascades, tyrosine metabolism, and the MAPK signaling pathway (Supplementary Fig. 1d). As the administration time increased to 5 days or 10 days, the enriched pathways of the top 1000 upregulated genes shifted to the EGFR-TKI resistance pathway, p53 signaling pathway and chemokine signaling pathway, alongside the persisting PI3K-AKT signaling pathway and MAPK signaling pathway; the pattern of the top 1000 downregulated genes enriched altered from metabolism-oriented signaling pathways to synthesis-oriented signaling pathways (Supplementary Fig. 1e, 1f). With increasing administration time, “regulation of angiogenesis” was significantly enriched in the top 1000 upregulated genes, whereas for the top 1000 downregulated genes, the enriched biological process pattern changed from metabolism-oriented processes to synthesis-oriented processes (Supplementary Fig. 1g-1l). These results demonstrated that significant alterations in multiple signals and biological processes could be involved in the development of trametinib resistance in A549 cells. Furthermore, we performed similar analyses on A549 cells and 3 other KRAS-mutant NSCLC cell lines, H460, Calu-1, and H23. The integrative transcriptome data revealed significant upregulation of both the MAPK and PI3K-AKT pathways after 10 days of trametinib treatment in A549 cells, H460 cells, Calu-1 cells and H23 cells (Fig. 2b), which was consistent with the western blot data (Fig. 2a).

The factors responsible for the upregulation of the MAPK and PI3K-AKT pathways in trametinib-induced adaptive resistance in KRAS-mutant NSCLC remain unclear. Therefore, we further analyzed the enriched pathways associated with the genes whose expression was altered after trametinib treatment. Interestingly, the results suggested that the differentially expressed genes are enriched in the angiogenesis pathway rather than the RAS pathway at different time points after trametinib treatment (Supplementary Fig. 2a-2f). The angiogenesis pathway involves various RTK signals, which are known to activate both the downstream MAPK pathway and the PI3K-AKT pathway.^27,28^ Therefore, we focused on the dynamic changes in RTK expression. In long-term observations, as trametinib-induced apoptosis gradually diminished in A549 and H23 cells (Supplementary Fig. 2g-2i), we concurrently observed the activation of multiple RTKs, such as platelet-derived growth factor receptor (PDGFR), FGFR, and tyrosine-protein kinase KIT (c-KIT) (Fig. 2c). Similar results for RTK activation were also observed in H460 and Calu-1 cells (Fig. 2c). Real‒polymerase chain reaction (PCR) quantitative data further demonstrated that the mRNA levels of multiple RTKs, especially PDGFRB, FGFR2 and c-KIT, were significantly increased after 5 or 10 days of trametinib treatment (Fig. 2d–f, Supplementary Fig. 2j-2l).

Furthermore, the protein levels of multiple RTKs (both phosphorylated and nonphosphorylated) were examined in KRAS-mutant NSCLC cells following trametinib treatment for 10 days. We observed the upregulation of p-PDGFR, PDGFR, protein fibroblast growth factor receptor substrate 2 (FRS2), p-FRS2, p-c-KIT, c-KIT, p-SHP2, SHP2, epidermal growth factor receptor (EGFR), p-EGFR, and p-ERBB3 in A549 cells; the upregulation of p-PDGFR, PDGFR, p-FRS2, FRS2, p-SHP2, SHP2, p-EGFR, EGFR, receptor tyrosine-protein kinase erbB-3 (ERBB3), p-ERBB3, vascular endothelial growth factor receptor (VEGFR), and p-VEGFR in Calu-1 cells; the upregulation of p-PDGFR, PDGFR, p-FRS2, FRS2, p-c-KIT, c-KIT, p-SHP2, SHP2, p-EGFR, EGFR, p-ERBB3, ERBB3, and p-VEGFR in H460 cells; and the upregulation of p-PDGFR, PDGFR, p-FRS2, FRS2, p-c-KIT, c-KIT, p-SHP2, SHP2, p-EGFR, EGFR, p-ERBB3, ERBB3, p-VEGFR, and VEGFR in H23 cells (Fig. 2g). These results demonstrated that different RTKs are activated in various KRAS-mutant NSCLC cell lines following long-term trametinib treatment. The upregulation of ERK and AKT may also be ascribed to the activation of these RTKs, which together are involved in the development of adaptive resistance to the MEK inhibitor trametinib (Supplementary Fig. 2m).

### Combined treatment with the MEK inhibitor trametinib and the RTK inhibitor anlotinib dramatically suppressed KRAS-mutant NSCLC growth

Here, we hypothesized that targeting multiple RTKs via the pan-RTK inhibitor anlotinib might overcome adaptive resistance to trametinib. Anlotinib is known to target various RTKs, including VEGFR1-3, PDGFRα-β, c-KIT and FGFR1-4, demonstrating high efficacy with substantial suppression of cell proliferation in multiple cancers.^29–33^ To investigate the potential antitumor efficacy of anlotinib monotherapy in KRAS-mutant NSCLC, we first performed a retrospective analysis of patients harboring various KRAS mutations enrolled in the ALTER-0303 study,^31^ a phase III clinical trial (NCT02388919) evaluating the efficacy of anlotinib in the third-line or later treatment of advanced NSCLC. Among the 29 patients harboring KRAS mutations, 25 (86.2%) had mutations occurring in exon 2, while 4 (13.8%) had mutations occurring in exons 3 or 4; 18 (62.1%) had previously undergone two regimens of systemic therapy, and 11 (37.9%) had received more than three lines of therapy (Supplementary Fig. 3a). Other clinical information, including age, sex, smoking history, Eastern Cooperative Oncology Group (ECOG) score, pathology, and stage, for these patients is shown in Supplementary Fig. 3a. These advanced pan-KRAS-mutant NSCLC patients (*n* = 29) who received anlotinib monotherapy had a total objective response rate (ORR) of 27.6%, a median progression-free survival (PFS) of 127 days, and a median overall survival (OS) of 240 days (Supplementary Fig. 3b-d). These results demonstrated that patients, regardless of which KRAS mutation they harbor, have the potential to benefit from anlotinib monotherapy. Furthermore, we analyzed patients who achieved a partial response (PR), and the results suggested that patients harboring various KRAS mutation subtypes, including G12C, G12V, A146, and Q61K, achieved a durable response to anlotinib monotherapy (Supplementary Fig. 3e). We subsequently tested the inhibitory effectiveness of anlotinib in various KRAS-mutant NSCLC cell lines, including G12C, G12D, G12S, G12V, and Q61H, in vitro. The results also demonstrated a significant inhibitory effect regardless of the KRAS mutation subtype (Supplementary Fig. 3f, g). These results suggest that although the pan-RTK inhibitor anlotinib has potential therapeutic efficacy in pan-KRAS-mutated NSCLC, its efficacy is not impressive, especially in the clinic.

Therefore, we next sought to use the pan-RTK inhibitory effect of anlotinib to block the adaptive resistance of trametinib and develop a novel combination strategy for KRAS-mutant NSCLC. Here, we first investigated whether the inhibition of ERK/AKT is sustained when KRAS-mutant NSCLC cells are exposed to long-term administration of trametinib plus anlotinib. The results indicated that 10 days of treatment with both trametinib and anlotinib prevented nearly all the rebound of ERK phosphorylation and AKT phosphorylation in various KRAS-mutant NSCLC cells (Fig. 2g). More importantly, multiple RTKs, including PDGFR, FSR (FGFR), c-KIT, EGFR, ERBB3, and VEGFR, as well as downstream SHP2 phosphorylation, were continuously suppressed when both trametinib and anlotinib were administered long-term (Fig. 2g). These results indicate that the RTK inhibitor anlotinib effectively blocks the MEK inhibitor trametinib-induced activation of multiple RTKs, resulting in long-term inhibition of the RTK pathway, MEK-ERK pathway, and AKT pathway. These findings suggest a potential synergistic inhibitory effect in KRAS-mutant NSCLC cells.

**Fig. 3:**
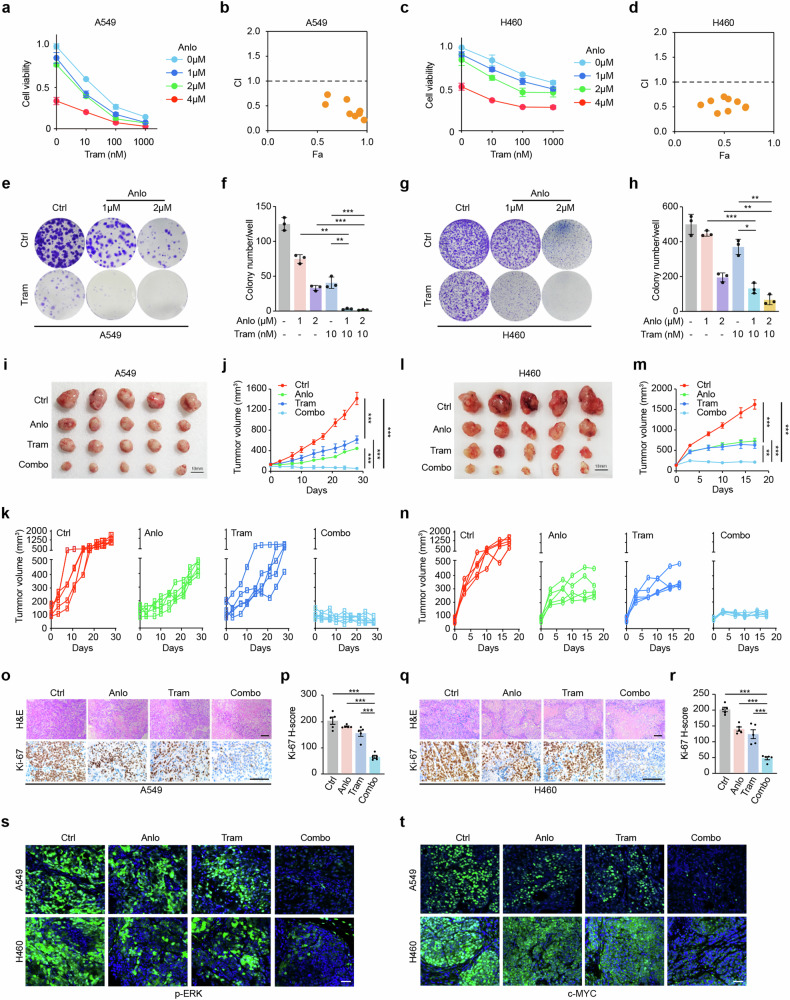
Combining MEK and pan-RTKs with trametinib and anlotinib significantly suppressed KRAS-mutant NSCLC progression. **a**–**d** Line graph showing the effects of combined trametinib and anlotinib treatments in A549 cells (**a**) and H460 cells (**c**), respectively. Synergistic analysis of the combined trametinib and anlotinib treatments in A549 cells (**b**) and H460 cells (**d**), respectively. CI < 1, synergism; CI = 1, additivity; CI > 1, antagonism. *n* = 3. **e**–**h** Clonogenic assay of A549 cells (**e**) and H460 (**g**) cells after the indicated treatments. Statistical analyses of A549 (**f**) and H460 (**h**) cells. *n* = 3. **i**–**n** A549 cells (**i**–**k**) or H460 cells (**l**–**n**) were subcutaneously injected into nude mice for xenograft assays and then subjected to the following treatments: Ctrl: vehicle, Anlo: anlotinib (1.5 mg/kg), Tram: trametinib (0.3 mg/kg) or Combo: trametinib (0.3 mg/kg) plus anlotinib (1.5 mg/kg). *n* = 5 for each group. Tumor gross inspection of the A549 xenograft model (**i**) and H460 xenograft model (**l**). Tumor growth curves of the A549 xenograft model in different groups (**j**) as well as independent xenograft tumors (**k**). Tumor growth curves of the H460 xenograft model in different groups (**m**) as well as independent xenograft tumors (**n**). *n* = 5. **o**–**r** Represe**n**tative H&E and Ki-67 staining of A549 (**o**) or H460 (**q**) xenograft tumors. Quantification of Ki67 staining of A549 (**p**) or H460 (**r**) tumors. Scale bar: 100 µm. Anlotinib; Trame, Trametinib; Combo, combination. *n* = 5. **s**, **t** Immunofluorescence analyses of p-ERK (**s**) and c-Myc (**t**) in A549 and H460 xenograft tumors. Scale bar: 50 μm. *n* = 5. All data comparisons were conducted via two-tailed Student’s t test. The data are presented as the means ± SEMs. *P < 0.05, **P < 0.01, ***P < 0.001

This finding confirmed that the combination of trametinib with anlotinib had a significant synergistic antiproliferative effect on different KRAS-mutant NSCLC cell lines, including H23, Calu-1, SW1573, H358, A549, SK-LU-1, H460 and SW900, in vitro (Fig. 3a–d, Supplementary Fig. 4a). Moreover, this combined strategy also significantly suppressed colony formation in A549 and H460 cells (Fig. 3e–h). Other synergistic inhibitory phenotypes, including cell cycle arrest, promotion of apoptosis, and inhibition of cell migration and invasion, were also observed in A549 cells (Supplementary Fig. 4b-4i). Consistent with the high synergistic inhibitory efficacy, our transcriptome analysis revealed underlying reasons, mainly attributed to the inhibition of the cell cycle pathway and DNA replication pathway (Supplementary Fig. 4j-4l). Further examination at the protein level suggested significant inhibition of cyclin E2 (CCNE2), cyclin-dependent kinase 2 (CDK2), and cyclin D1 (CCND1) expression after coinhibition of the MEK pathway and RTK pathways (Supplementary Fig. 4m, n). Moreover, poly (ADP‒ribose) polymerase (PARP) expression was markedly inhibited, whereas cleaved PARP expression was significantly increased (Supplementary Fig. 4m, n). Importantly, cellular myelocytomatosis oncogene (c-MYC) expression was nearly blocked (Supplementary Fig. 4 m, 4n). These results provide potential insight into the above phenotypes induced by combined treatment with the MEK inhibitor trametinib and the RTK inhibitor anlotinib.

Using xenograft tumor models derived from A549, H460, H23 and Calu-1 cells, we further demonstrated the high efficacy of combined treatment with trametinib and anlotinib (Fig. 3i–n, Supplementary Fig. 5a-5f). The average tumor volume in the combination treatment group was significantly lower than that in either the trametinib or anlotinib monotherapy groups (Fig. 3j, m; Supplementary Fig. 5b, 5e). All the tumors in the combined treatment groups continuously shrank without signs of rebound (Fig. 3k, n, Supplementary Fig. 5c, 5 f). Additionally, analysis of the maximum percentage reduction from baseline indicated that the combined treatment induced more significant tumor shrinkage in H460- and H23-derived xenograft tumor models than in those derived from A549 and Calu-1 cells (Supplementary Fig. 5g). The synergistic inhibitory effectiveness was further confirmed by dramatic decreases in Kiel 67 (Ki67), p-ERK, and c-MYC staining (Fig. 3o–t, Supplementary Fig. 5h-k). No significant decrease in body weight was observed following the combined strategy, except in the Calu-1 model, indicating favorable body tolerance (Supplementary Fig. 5l-5o). Furthermore, in vivo experiments further demonstrated that the combination of trametinib and anlotinib synergistically promoted apoptosis (Supplementary Fig. 5p). Collectively, these results suggest that coinhibition of the MEK pathway and RTK pathways via trametinib plus anlotinib can induce long-term proliferation inhibition in KRAS-mutant NSCLC cells both in vitro and in vivo (Supplementary Fig. 5q).

### The MEK inhibitor trametinib plus the RTK inhibitor anlotinib induces synergistic antitumor efficacy in KRAS-mutant NSCLC, potentially via the MEK/RTK-IGFBP2-RTK signaling loop

**Fig. 4:**
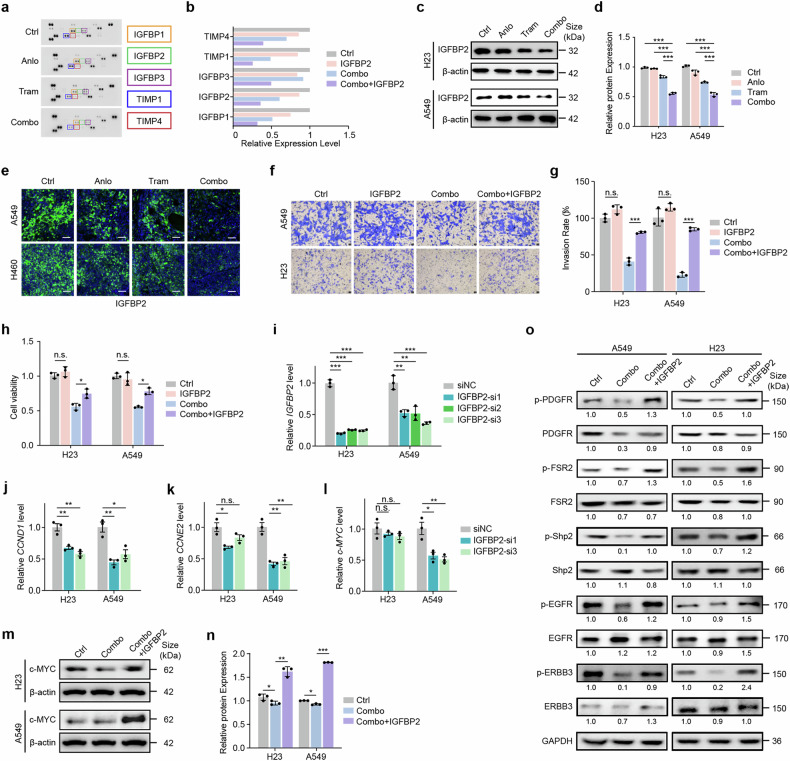
IGFBP2 contributes to the high antitumor efficacy of MEK inhibitor-trametinib and RTK inhibitor-anlotinib combination treatments. **a**, **b** Angiogenesis-related protein chip analyses (**a**) and related statistical quantification (**b**) of A549 cells after 24 h of trametinib (10 nM), anlotinib (1 μM) or combination treatment. **c**, **d** Western blot analyses (**c**) and related statistical analyses (**d**) of the IGFBP2 levels in A549 and H23 cells after 48 h of trametinib (10 nM), anlotinib (1 μM) or combination treatment. *n* = 3. **e** Immunofluorescence analyses of IGFBP2 levels in A549 and H460 xenograft tumors. Scale bar: 50 μm. *n* = 5. **f**, **g** Invasion analyses of A549 and H23 cells treated with trametinib plus anlotinib, IGFBP2, or both for 24 h (**f**) and related statistical analyses (**g**). Scale bar: 50 μm. *n* = 3. **h** Viability of A549 and H23 cells treated with trametinib plus anlotinib, IGFBP2 or both for 24 h. *n* = 3. **i**‒**l** RT‒PCR quantification of IGFBP2 (**i**), CCND1 (**j**), CCNE2 (**k**), and c-MYC (**l**) mRNA levels in A549 and H23 cells upon IGFBP2 knockdown. *n* = 3. **m, n** Western blot analyses of c-MYC levels in A549 cells treated with trametinib plus anlotinib or supplemented with IGFBP2 for 24 h (**m**) and related statistical analyses (**n**). *n* = 3. **o** Western blot analyses of RTK expression in A549 and H23 cells treated with trametinib plus anlotinib or supplemented with IGFBP2 for 10 days. Statistical analyses were performed via two-tailed Student’s t test. The data are presented as the means ± SEMs. *P < 0.05, **P < 0.01, ***P < 0.001

A deeper investigation into the underlying synergistic mechanism of the MEK inhibitor trametinib combined with the RTK inhibitor anlotinib will provide the theoretical foundation necessary for the clinical translation of this strategy. While our above results illustrate that coinhibition of the MEK and RTK pathways is crucial for antitumor efficacy in KRAS-mutant NSCLC (Figs. 2g, 3, Supplementary Fig. 4, 5), the precise synergistic mechanism driving this effect remains to be elucidated. Considering that the RTK inhibitor anlotinib acts as an antiangiogenic drug, we performed angiogenesis-related protein chip screening on KRAS-mutant NSCLC cells treated with the combined strategy. Our data revealed significant downregulation of insulin-like growth factor binding protein (IGFBP) family proteins (including IGFBP1, IGFBP2, and IGFBP3) and thymidylate kinase Tmp (TMP) family proteins (TMP1 and TMP4) following treatment with trametinib plus anlotinib (Fig. 4a, b). Notably, the expression of IGFBP2, known for its role in regulating cancer cell proliferation and metastasis,^34–36^ was significantly downregulated after combined treatment in H23 and A549 cells (Fig. 4c, d), which was also confirmed in A549- and H460-derived xenograft tumor models (Fig. 4e). We subsequently observed that supplementation with exogenous IGFBP2 attenuated the suppression of cell invasion and viability induced by the combined treatment (Fig. 4f–h). Moreover, knockdown of IGFBP2 led to significant inhibition of the transcription of cell cycle-related genes, notably CCND1 and CCNE2, in H23 and A549 cells and the oncogene c-MYC in A549 cells (Fig. 4i–l). Furthermore, the combined treatment-induced decrease in the c-MYC protein level could be rescued via the addition of exogenous IGFBP2 (Fig. 4m, n). More importantly, the combined treatment-induced inactivation of RTK pathways (including PDGFR, FSR (FGFR), EGFR, and ERBB3) along with downstream SHP2 could be reversed upon the addition of exogenous IGFBP2 (Fig. 4o). These results suggest that the synergistic antitumor activity induced by coinhibition of the MEK and RTK pathways is mediated through a reduction in IGFBP2. This is evidenced by the regulatory role of IGFBP2 in genes associated with tumor cell growth. The addition of exogenous IGFBP2 can rejuvenate RTK inactivation induced by coinhibition of the MEK and RTK pathways. Taken together, our preclinical data suggest that the combination strategy of trametinib plus anlotinib synergistically induces antitumor activity in KRAS-mutant NSCLC, potentially via the MEK/RTK-IGFBP2-RTK signaling loop.

### Definition of RP2D via clinical evaluation of combined MEK inhibitor-trametinib and RTK inhibitor-anlotinib therapy in non-G12C KRAS-mutant patients

**Fig. 5:**
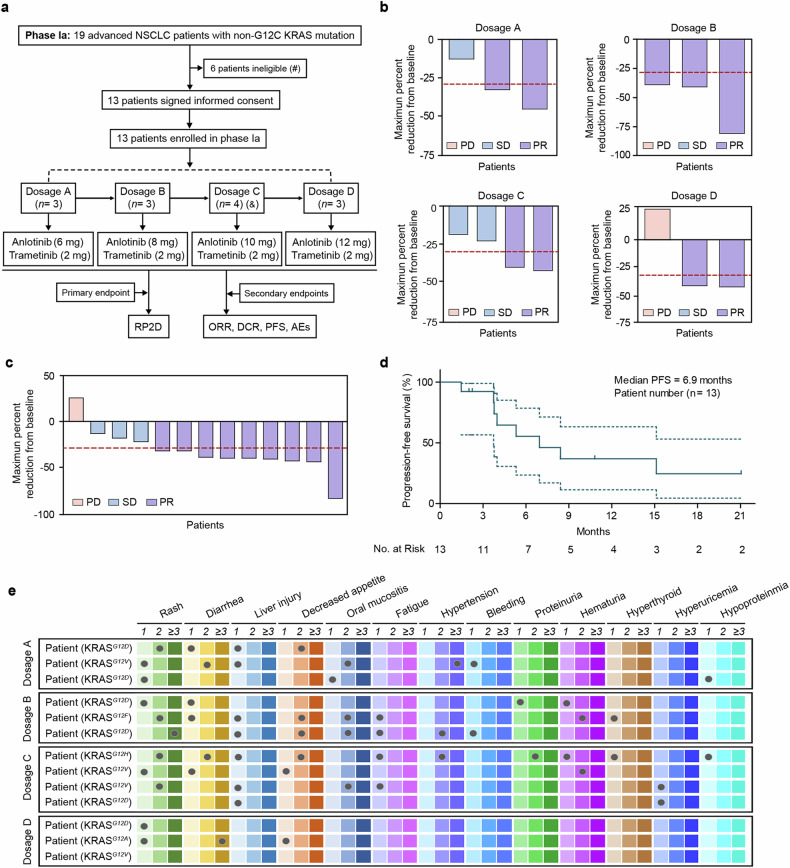
Analysis of therapeutic efficacy in a phase Ia clinical study. **a** Flowchart of the phase Ia clinical study. The symbols (#) and (&) indicate specific patient conditions: (#) denotes patients with untreated active brain metastases, those who received systemic antitumor therapy and radiation therapy within 28 days, or those with other exclusion criteria; (&) denotes that the size of dosage C was 4 because the last 2 patients were enrolled simultaneously. **b** Waterfall plots showing the percentages of tumor regression/progression. Dosage A, 3 patients received the combined regimen of trametinib (2 mg) plus anlotinib (6 mg). At dosage B, 3 patients received trametinib (2 mg) plus anlotinib (8 mg), whereas at dosage C, 4 patients received trametinib (2 mg) plus anlotinib (10 mg). At dosage D, 3 patients received the combination treatment of trametinib (2 mg) plus anlotinib (12 mg). **c** Waterfall plots showing the percentages of tumor regression/progression in the 13 enrolled advanced NSCLC patients. **d** Kaplan‒Meier plots of progression-free survival (PFS). **e** The adverse event (AE) profiles are presented for the A, B, C, and D dosage groups. The color gradient, from light to dark, represented different grades of AEs. The light color indicates Grade 1, the medium color indicates Grade 2, and the dark color represents Grade 3 and above. Each dot represents a patient who experienced an AE

On the basis of the above results, we initiated a clinical trial (phase Ia) to define the recommended phase 2 dose (RP2D) and evaluate the efficacy and safety of combined trametinib plus anlotinib therapy in advanced NSCLC patients with non-G12C KRAS mutations. The primary endpoint of phase Ia is to define the RP2D. A total of 19 NSCLC patients with various non-G12C KRAS mutations participated in the initial screening process. Among them, 6 patients were excluded due to untreated active brain metastases, recent systemic antitumor therapy and radiation therapy within 28 days, or other factors outlined in the exclusion criteria. The remaining 13 qualified non-G12C KRAS-mutant patients were sequentially assigned to 4 dosage groups to evaluate RP2D (Fig. 5a): dosage A received combination therapy with trametinib (2 mg) and anlotinib (6 mg) (*n* = 3, KRAS^*G12D*^, KRAS^*G12V*^ or KRAS^*G12D*^, respectively); dosage B received a dosage of 2 mg trametinib plus 8 mg anlotinib (*n* = 3, KRAS^*G12D*^, KRAS^*G12F*^ or KRAS^*G12D*^, respectively); dosage C received a dosage of 2 mg trametinib plus 10 mg anlotinib (*n* = 4, KRAS^*Q61H*^, KRAS^*G12V*^, KRAS^*G12V*^ or KRAS^*G12D*^, respectively); and dosage D received a dosage of 2 mg trametinib plus 12 mg anlotinib (*n* = 3, KRAS^*G12D*^, KRAS^*G12A*^ or KRAS^*G12V*^, respectively). Among these patients, 4 (30.8%) were females and 9 (69.2%) were males, with 7 (53.8%) having a history of smoking and 6 (46.2%) not (Table 1). Additionally, 1 (7.7%) patient was diagnosed with adenosquamous carcinoma (ASC), 1 (7.7%) with pulmonary sarcomatoid carcinoma (PSC), and 11 (84.6%) with adenocarcinoma (ADC) (Table 1). With respect to ECOG performance status (ECOG PS), 3 (23.1%) patients had an ECOG PS of 0, and 10 (76.9%) had an ECOG PS of 1. Six (46.2%) patients had <3 metastases, and 7 (53.8%) had ≥3 metastases. The average age was 61 years, with a range from 36--75 years (Table 1).

**Table 1:** Characteristics of the Patients at Baseline

Clinical information	Phase Ia (*n* = 13)	Phase Ib (*n* = 20)
Age (Median, y)	**61 (36** ~ **75)**	**62 (43** ~ **75)**
Gender		
Female	**4 (30.8%)**	**8 (40.0%)**
Male	**9 (69.2%)**	**12 (60.0%)**
Smoking history		
No	**6 (46.2%)**	**9 (45.0%)**
Yes	**7 (53.8%)**	**11 (55.0%)**
ECOG score		
0	**3 (23.1%)**	**6 (30.0%)**
1	**10 (76.9%)**	**14 (70.0%)**
Pathology		
ADC	**11 (84.6%)**	**17 (85.0%)**
NSCLC-NOS	**0 (0%)**	**3 (15.0%)**
ASC	**1 (7.7%)**	**0 (0%)**
PSC	**1 (7.7%)**	**0 (0%)**
KRAS mutation		
Exon 2	**12 (92.3%)**	**16 (80.0%)**
Exon 3	**0 (0%)**	**2 (10.0%)**
Other exons	**1 (7.7%)**	**2 (10.0%)**
Prior treatment		
0 line	**6 (46.2%)**	**0 (0%)**
1 line	**1 (7.7%)**	**11 (55.0%)**
2 lines	**6 (46.2%)**	**7 (35.0%)**
≥3 lines	**0 (0%)**	**2 (10.0%)**
Number of metastases		
<3	**6 (46.2%)**	**12 (60.0%)**
≥3	**7 (53.8%)**	**8 (40.0%)**

*ADC* adenocarcinoma, *ASC* adenosquamous carcinoma, *PSC* pulmonary sarcomatoid carcinoma, *NSCLC-NOS* non-small cell lung cancer, not otherwise specified, *ECOG* Eastern Cooperative Oncology Group

Among the 13 patients, 6 (46.2%) received the combination regimen of trametinib plus anlotinib as the first-line therapy, 1 (7.7%) received the second-line therapy, and 6 (46.2%) received the third-line therapy (Table 1). One patient from dosage C discontinued therapy, and 2 patients from dosage D were missed due to the COVID-19 pandemic. Up to the cutoff date (26-Mar-2024), the average follow-up time in this cohort was 15.6 months (range: 1.9–35.2 months). The confirmed ORR of these patients was 69.2% (9/13) (95% confidence interval (CI): 38.6–90.9), and the disease control rate (DCR) was 92% (12/13) (95% CI: 64.0–99.8) (Fig. 5b, c). The median PFS was 6.9 months (95% CI: 3.9 to not be evaluated) (Fig. 5d). The safety results indicated acceptable toxicity when the combination regimen of trametinib plus anlotinib was administered to non-G12C KRAS-mutant NSCLC patients (Fig. 5e). In summary, the most common adverse events (AEs) were rash (85%), diarrhea (62%), liver injury, including elevations in AST, ALT, ALP, and related markers (54%), decreased appetite (46%) and oral mucositis (38%) (Table 2). Three patients (23%) experienced grade 3 AEs, presenting with symptoms of rash, diarrhea, and hypertension (Table 2).

**Table 2:** Adverse events for phase Ia and Ib

	Adverse events	Grade 1	Grade 2	Grade 3	Grade 4 & 5	Total
Phase Ia	Rash, n (%)	6 (55)	4 (36)	1 (9)	0 (0)	11 (85)
	Diarrhea, n (%)	6 (75)	1 (12.5)	1 (12.5)	0 (0)	8 (62)
	Liver injury, n (%)	7 (100)	0 (0)	0 (0)	0 (0)	7 (54)
	Decreased appetite, n (%)	2 (33)	4 (67)	0 (0)	0 (0)	6 (46)
	Oral mucositis, n (%)	1 (20)	4 (80)	0 (0)	0 (0)	5 (38)
	Fatigue, n (%)	4 (100)	0 (0)	0 (0)	0 (0)	4 (31)
	Hypertension, n (%)	0 (0)	2 (67)	1 (33)	0 (0)	3 (23)
	Bleeding, n (%)	3 (100)	0 (0)	0 (0)	0 (0)	3 (23)
	Proteinuria, n (%)	1 (50)	1 (50)	0 (0)	0 (0)	2 (15)
	Hematuria, n (%)	1 (50)	1 (50)	0 (0)	0 (0)	2 (15)
	Hyperthyroid, n (%)	2 (100)	0 (0)	0 (0)	0 (0)	2 (15)
	Hyperuricemia, n (%)	2 (100)	0 (0)	0 (0)	0 (0)	2 (15)
	Hypoproteinemia, n (%)	2 (100)	0 (0)	0 (0)	0 (0)	2 (15)
	Paronychia, n (%)	1 (100)	0 (0)	0 (0)	0 (0)	1 (8)
	Epistaxis, n (%)	1 (100)	0 (0)	0 (0)	0 (0)	1 (8)
	Hyponatremia, n (%)	1 (100)	0 (0)	0 (0)	0 (0)	1 (8)
	Pyrexia, n (%)	1 (100)	0 (0)	0 (0)	0 (0)	1 (8)
Phase Ib	Rash, n (%)	6 (38)	5 (31)	5 (31)	0 (0)	11 (80)
	Diarrhea, n (%)	4 (40)	5 (50)	1 (10)	0 (0)	10 (50)
	Hypertension, n (%)	5 (56)	4 (44)	0 (0)	0 (0)	9 (45)
	Decreased appetite, n (%)	5 (62.5)	3 (37.5)	0 (0)	0 (0)	8 (40)
	Fatigue, n (%)	6 (75)	1 (12.5)	1 (12.5)	0 (0)	8 (40)
	Weight Loss, n (%)	6 (100)	0 (0)	0 (0)	0 (0)	6 (30)
	Bleeding, n (%)	4 (100)	0 (0)	0 (0)	0 (0)	4 (20)
	Oral mucositis, n (%)	1 (33)	2 (67)	0 (0)	0 (0)	3 (15)
	Hyperuricemia, n (%)	3 (100)	0 (0)	0 (0)	0 (0)	3 (15)
	Hypoproteinemia, n (%)	3 (100)	0 (0)	0 (0)	0 (0)	3 (15)
	Liver injury, n (%)	2 (100)	0 (0)	0 (0)	0 (0)	2 (10)
	Anemia, n (%)	2 (100)	0 (0)	0 (0)	0 (0)	2 (10)
	Hyponatremia, n (%)	2 (100)	0 (0)	0 (0)	0 (0)	2 (10)
	Hematochezia, n (%)	2 (100)	0 (0)	0 (0)	0 (0)	2 (10)
	Emesis, n (%)	1 (100)	0 (0)	0 (0)	0 (0)	1 (5)
	Alopecias, n (%)	1 (100)	0 (0)	0 (0)	0 (0)	1 (5)
	Retinal cell exfoliation, n (%)	1 (100)	0 (0)	0 (0)	0 (0)	1 (5)
	Dizziness, n (%)	1 (100)	0 (0)	0 (0)	0 (0)	1 (5)
	Hypothyroidism, n (%)	0 (0)	1 (100)	0 (0)	0 (0)	1 (5)
	Emesis	1 (100)	0 (0)	0 (0)	0 (0)	1 (5)
	Epistaxis, n (%)	1 (100)	0 (0)	0 (0)	0 (0)	1 (5)

To define the RP2D, we conducted a comprehensive comparison of the maximum tolerated dose (MTD), ORR, and average tumor shrinkage rate for each dosage group. MTD served as the primary indicator. However, no MTD was observed within the indicated anlotinib dosage range. Therefore, the ORR and average tumor shrinkage rate were sequentially utilized to define the RP2D. Our results indicated that the ORR of dosage A was 66.7%, with an average tumor shrinkage of 30%; that of dosage B was 100%, with an average tumor shrinkage of 56%; that of dosage C was 50%, with an average tumor shrinkage rate of 28%; and that of dosage D was 66.7%, with an average tumor shrinkage rate of 17% (Fig. 5b). In terms of the ORR, the 3 patients in dosage B (harboring the KRAS^*G12D*^, KRAS^*G12F*^ and KRAS^*G12D*^ mutations) who received 2 mg of trametinib plus 8 mg of anlotinib demonstrated the highest drug response. On the basis of these results, we suggest that the dosage of 2 mg of trametinib plus 8 mg of anlotinib should be further investigated in an extended cohort to evaluate the ORR of the combination strategy.

Furthermore, we investigated the associations between clinical characteristics and the ORR and PFS. Interestingly, the ORRs of female patients and male patients were 50% and 78%, respectively, whereas the median PFSs of female patients and male patients were undefined and 6.9 months, respectively (95% CI: 0.09–1.82; hazard ratio (HR): 0.41) (Supplementary Table 1). Patients with a history of smoking had a higher ORR (86%), whereas those without a smoking history had a more favorable PFS (8.4 months) (Supplementary Table 1). Furthermore, patients with ECOG PS = 1, pathological type of ADC, KRAS mutation in exon 2, prior treatment ≥1 line, or number of metastases ≥3 received a greater ORR and PFS benefit (Supplementary Table 1). Specifically, the 6 treatment-naive patients enrolled in phase Ia had an ORR of 66.7% and a median PFS of 5.3 months (range: 2.2 months to 15.1 months) (Supplementary Table 1).

### Evaluation of the ORR in non-G12C KRAS-mutant NSCLC patients treated with trametinib plus anlotinib—a phase Ib study

**Fig. 6:**
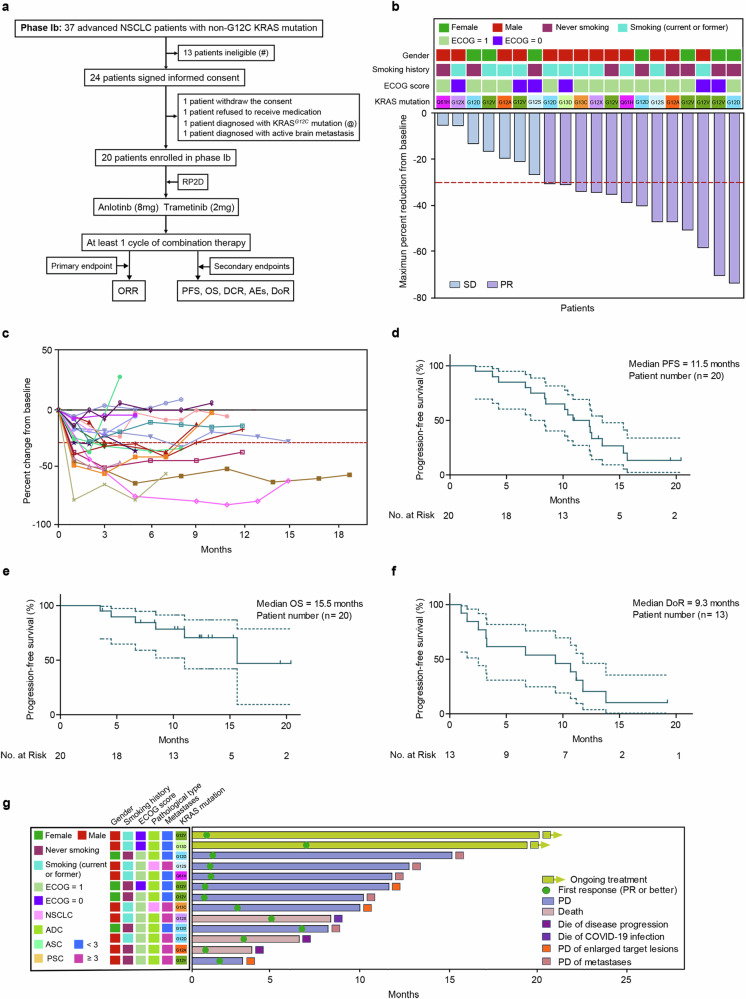
Analysis of therapeutic efficacy in phase Ib clinical studies. **a** Flowchart of the phase Ib clinical study. The symbols (#) and (@) indicate specific patient conditions: (#) denotes patients with untreated active brain metastases, those receiving systemic antitumor therapy and radiation therapy within 28 days, or those with other exclusion criteria; (@) denotes patients diagnosed with KRAS^*G12C*^ mutation upon later pathological examination. **b** Waterfall plots showing the percentages of tumor regression in the 20 enrolled advanced NSCLC patients. **c** Percent change from baseline over time is shown. **d** Kaplan‒Meier plots of progression-free survival (PFS). **e** Kaplan‒Meier plots of overall survival (OS). **f** Kaplan‒Meier plots of the duration of overall response (DoR). **g** DoR analysis of the 20 KRAS-mutant NSCLC patients enrolled in phase Ib

To further evaluate the ORR, we initiated a phase Ib clinical trial using a combination regimen of trametinib (2 mg) and anlotinib (8 mg). A total of 37 advanced NSCLC patients with non-G12C KRAS mutations who had received at least 1^st^-line standard therapy participated in the screening process (Fig. 6a). Among them, 13 patients with untreated active brain metastases, recent systemic antitumor therapy, radiation therapy within 28 days, or other exclusionary factors did not fulfill the eligibility criteria. The remaining 24 patients signed informed consent. Among these 24 patients, 4 failed to continue the trial (1 withdrew consent, 1 refused to receive medication, 1 was confirmed to have the KRAS^*G12C*^ mutation in the following pathological report, and 1 was diagnosed with active brain metastasis) (Fig. 6a). All 20 eligible patients received combination therapy with 2 mg of trametinib plus 8 mg of anlotinib (Fig. 6a). Among these patients, 6 (30%) harbored the KRAS^*G12V*^ mutation, 4 (20%) harbored the KRAS^*G12D*^ mutation, and 10 (50%) harbored other types of KRAS mutations (including G12A, G12S, G12X, G13C, G13D, and Q61H). Eight patients (40%) were female, and 12 (60%) were male, with 11 (55%) having a history of smoking and 9 (45%) without such a history (Table 1). Three patients (15%) were diagnosed with non-small cell lung cancer, not otherwise specified (NSCLC-NOS), and 17 (85%) were diagnosed with ADC (Table 1). Six (30%) had an ECOG PS = 0, and 14 (70%) had an ECOG PS = 1 (Table 1). Twelve (60%) had fewer than 3 metastases, whereas 8 (40%) had 3 or more metastases (Table 1). The average age was 62 years, ranging from 43 to 75 years (Table 1). Among the 20 patients, 11 (55%) were treated with the combination regimen of anlotinib plus trametinib as the 2^nd^-line therapy, and 9 (45%) received it as 3^rd^-line or later therapy (Table 1).

Up to the cutoff date (26-Mar-2024), the average follow-up time in this cohort was 13.3 months (range: 3.4–21.2 months). The overall ORR was 65% (13/20) (95% CI: 40.8–84.6), and the DCR was 100% (20/20) (95% CI: 83.2–100.0) (Fig. 6b, c). Sixteen (80%) patients experienced events (10 patients with progressive disease (PD) with enlarged targeted lesions or metastases, 3 patients who died from COVID-19 infection, and 3 patients who died from disease progression). The average tumor shrinkage rate was 35.6% (range: 5–84%) (Fig. 6b). The majority of patients achieved a durable response, and all patients achieved disease control following administration of the combination regimen (Fig. 6c). The median PFS was 11.5 months (95% CI: 8.3–15.5) (Fig. 6d), and the median OS was 15.5 months (95% CI: 15.5 to not be evaluated) (Fig. 6e). Among the 13 patients who achieved PR, 3 patients (23.1%) died, 8 patients (61.5%) experienced PD, and the remaining 2 patients (15.4%) were still receiving treatment (Fig. 6f, g). The median duration of overall response (DoR) was 9.3 months (95% CI: 2.5–12.1) (Fig. 6f). The AEs caused by the combination regimen are shown in Table 2. The results revealed that the most common AEs were rash (80%), diarrhea (50%), hypertension (45%), decreased appetite (40%) and fatigue (40%) (Table 2). Seven patients (35%) reported grade 3 AEs with symptoms of rash, hypertension, and fatigue (Table 2).

Subgroup analysis was also performed during the phase Ib trial (Supplementary Table 2). The results indicated that patients who were male, had an ECOG PS of 1, had a KRAS mutation in exons other than exon 2, or had ≥3 metastases had a favorable ORR (Supplementary Table 2). The patients who were female, nonsmokers, had an ECOG PS of 0, had a pathological type of ADC, had a KRAS mutation in exons other than exon 2, had prior treatment of 1 line, or had <3 metastases received greater PFS benefits (Supplementary Table 2).

### Integrative analysis of the phase I (phase Ia plus phase Ib) clinical trial

We further performed an integrative analysis involving 33 NSCLC patients from a phase I study (phase Ia plus phase Ib) (Supplementary Fig. 6a). The comprehensive clinical information for all enrolled non-G12C patients is presented in Supplementary Table 3. The average age was 61.8 years, ranging from 36 to 75 years. Among them, 21 patients (63.6%) were male, and 12 (36.4%) were female; 18 (54.5%) had a history of smoking, while 15 (45.5%) did not; 28 (84.8%) were diagnosed with ADC, 3 (9.1%) with NSCLC-NOS, 1 (3%) with ASC, and 1 (3%) with PSC. Nine patients (27.3%) had an ECOG PS score of 0, 24 (72.7%) had a score of 1, 18 (54.5%) had fewer than 3 metastases, and 15 (45.5%) had 3 or more metastases. Twenty-eight patients (84.8%) harbored KRAS^*Exon 2*^ mutations, 2 (6.1%) harbored KRAS^*Exon 3*^ mutations, and 3 (9.1%) harbored other KRAS mutations. Six patients (18.2%) were treatment naive, 12 (36.4%) had received 1^st^-line therapy, 13 (39.4%) had received 2^nd^-line therapies, and 2 (6.1%) had received 3^rd^-line or later therapies.

Among the 33 enrolled non-G12C KRAS-mutant NSCLC patients, the median PFS was 10.3 months (95% CI: 7.0–15.1), and the median OS was 19.7 months (95% CI: 15.5 to not be evaluated) (Supplementary Fig. 6b, 6c). Furthermore, the overall ORR was 66.7% (22/33) (95% CI: 42.1–77.1), with an average tumor shrinkage rate of 34.4% (range: -26% ~ 84%), and the majority of patients achieved a durable response (22/33) or disease control (the DCR was 97%, 95% CI: 89.4–100.0) (Supplementary Fig. 7). Among the 22 patients who achieved PR, 18 (81.8%) achieved their first response within 4 cycles, with 4 (18.2%) patients still receiving treatment as of the cutoff date (Supplementary Fig. 8a). However, 12 patients (54.5%) ultimately achieved PD with enlarged target lesions or metastases, 4 patients ultimately died of other acute diseases, COVID-19 infection or disease progression, and 2 patients were missed due to the COVID-19 pandemic (Supplementary Fig. 8a). The 22 patients ultimately achieved a median DoR of 9.3 months (95% CI: 3.3--11.2) (Supplementary Fig. 8b).

Among the 33 patients, 10 (30%) harbored KRAS^*G12V*^, 10 (30%) harbored KRAS^*G12D*^, and 13 (40%) harbored other types of KRAS mutations [including G12A (3, 9%), Q61H (3, 9%), G12S (2, 6%), G12X (2, 6%), G12F (1, 3%), G13C (1, 3%), and G13D (1, 3%)] (Supplementary Fig. 9a). The patients harboring the KRAS^*G12V*^ mutation had an ORR of 50% and a median PFS of 10.3 months (Supplementary Fig. 9b-9d). Among them, 2 patients experienced a PFS exceeding 18 months and were still receiving ongoing treatment until the cutoff date (Supplementary Fig. 9c). Patients harboring KRAS^*G12D*^ had an ORR of 80% and a median PFS of 11.7 months (Supplementary Fig. 10), whereas those harboring other KRAS mutations had an ORR of 69% and a median PFS of 11.1 months (Supplementary Fig. 11). Interestingly, the majority of patients with KRAS^*other*^ mutations were male or nonsmokers, whereas all patients with the KRAS^*G12D*^ mutation had an ECOG score of 1, were diagnosed with ADC, and had fewer distant metastases (Supplementary Fig. 10a, 11a). Patients with the KRAS^*G12V*^ mutation did not differ in sex, smoking history, ECOG score, pathological type, or metastasis status (Supplementary Fig. 9b). Among the 33 enrolled patients, AEs with frequencies above 10% included rash (82%), diarrhea (55%), decreased appetite (42%), hypertension (36%), fatigue (36%), liver injury (27%), oral mucositis (24%), weight loss (18%), bleeding (15%), hyperuricemia (15%), and hypoproteinemia (15%) (Supplementary Table 4).

Furthermore, in the phase I study, patients who were male, or had a history of smoking, or had an ECOG score of 1, or had pathological type of ADC, or had KRAS mutation in exon 2, or had 2 or more lines of standard treatment, or had 3 or more metastases, received a favorable ORR (Supplementary Table 5). On the other hand, patients who were female, nonsmokers, had an ECOG score of 0, had pathological type of ADC, had KRAS mutation on KRAS mutation in other exons besides exon 2, had received 1^st^-line of standard treatment, or had fewer than 3 metastases, received more benefit in terms of PFS (Supplementary Table 5). Collectively, these results suggest that the combination strategy of trametinib plus anlotinib has potential clinical value for advanced NSCLC patients with non-G12C KRAS mutations.

## Discussion

Our study aimed to develop a novel combined strategy involving coinhibition of the MEK/RTK pathway for the treatment of various KRAS-mutant NSCLCs. KRAS-mutant NSCLC has been considered undruggable in recent decades^9^ because of the various KRAS mutants, including G12C, G12D, G12S, G12V, Q61H, etc.,^6,7,14^ as well as structural challenges associated with the KRAS protein.^20^ However, recent advances have shattered this conventional wisdom. Agents such as sotorasib,^11^ adagrasib^12^ and divarasib,^13^ which target the KRAS^*G12C*^ mutant, have demonstrated effectiveness in clinical trials for NSCLC patients. In addition to these findings, recent studies have also made important advances in the development of small-molecule RAS inhibitors as well as their mechanisms for inhibiting the RAS pathway.^37–39^ However, developing therapeutic approaches for pan-KRAS-mutant NSCLC via MEK-based combined strategies remains a challenge.

Numerous efforts have focused on targeting KRAS-driven tumors via the inhibition of downstream effectors via MEK inhibitors.^17–19,40^ However, the efficacy of MEK inhibitor monotherapy is transient and can lead to feedback activation of ERK1/2 as well as other bypass activations (PI3K-AKT, FGFR, SHP2, AXL, etc.), leading to diminished inhibitory effectiveness.^21–25,41,42^ To further elucidate the underlying mechanism of adaptive resistance in KRAS-mutant NSCLC following treatment with the MEK inhibitor trametinib, we identified multiple upregulated RTKs (PDGFR, FGFR, c-KIT, etc.) that may be responsible for adaptive resistance. Additionally, we also observed that the activation of RTKs occurs in a cell context-dependent manner. Therefore, the combination of MEK inhibitors and RTK inhibitors has emerged as a promising therapeutic approach for pan-KRAS-mutant NSCLC.

Owing to the distinct sets of RTKs activated in different KRAS-mutant NSCLC cells following MEK inhibitor-trametinib treatment, the heterogeneity of this adaptive resistance makes the use of combination therapies with a MEK inhibitor and a single-target RTK inhibitor impractical.^26^ To address this issue, we screened a pan-RTK inhibitor, anlotinib, which has demonstrated antitumor activity via the inhibition of VEGFR1, VEGFR2, VEGFR3, c-KIT, PDGFRα, FGFR1, FGFR2, and FGFR3,^30–33,43–45^ to block the adaptive resistance of the MEK inhibitor trametinib. Our findings indicated that treatment with the MEK inhibitor trametinib plus the RTK inhibitor anlotinib significantly inhibited the growth of KRAS-mutant NSCLC cells (G12C, G12D, G12V, G12S, and Q61H). Additionally, this combined strategy induced tumor shrinkage in various KRAS-mutant NSCLC xenograft models (G12C, G12S, and Q61H) as well as in various KRAS-mutant NSCLC patients (G12D, G12F, G12S, G12V, and Q61H).

The combined strategy of the MEK inhibitor trametinib plus the RTK inhibitor anlotinib effectively inhibits the feedback activation of ERK and results in long-term inhibition of AKT in KRAS-mutant NSCLC, preliminarily explaining the synergistic antitumor activity. These findings may be attributed to anlotinib blocking the adaptive activation of RTKs, thus further precluding the feedback activation of ERK and AKT induced by long-term trametinib treatment. Further investigation suggested that the mechanism of the synergistic antitumor activity of the combined strategy in KRAS-mutant NSCLC is potentially mediated through the MEK/RTK-IGFBP2-RTK signaling loop. This preliminary illustration of the synergistic mechanism of the MEK inhibitor trametinib combined with the RTK inhibitor anlotinib provides a theoretical basis for the clinical translation of this novel combined strategy for KRAS-mutant NSCLC.

Nevertheless, the clinical translation of mechanistic studies is a crucial determinant of their potential benefits for patients. Previous attempts by scientists to develop various combination strategies (such as MEK inhibitors plus FGFR1 inhibitors, chemotherapy, SHP2 or SOS1 inhibitors) based on mechanistic or clinical studies have not shown significant benefits for patients with KRAS mutations.^17,18,21,22,46^ Interestingly, the successful application of the MEK inhibitor trametinib combined with the B-Raf proto-oncogene serine/threonine kinase (BRAF) inhibitor dabrafenib for the treatment of BRAF^*V600*^-mutant NSCLC has renewed confidence in the development of novel combination strategies for KRAS-mutant NSCLC.^47–49^ In our study, treatment with the MEK inhibitor trametinib plus the RTK inhibitor anlotinib markedly decreased the tumor burden of targeted lesions in a phase I cohort of 33 advanced NSCLC patients harboring various non-G12C KRAS mutations.

One of the most concerning points in phase I clinical studies for cancer is the occurrence of AEs induced by combination therapy. Our results indicated that there were no grade 4 or 5 AEs observed after trametinib plus anlotinib therapy. The most common grade 3 AEs observed were rash, diarrhea, and hypertension, and the majority of the AEs were grade 1 or 2. Interestingly, some changes were observed in the AE profile after combination therapy compared with that after trametinib or anlotinib alone.^19,31,50^ In the trametinib phase II clinical study, the most common grade 3 AEs were hypertension, rash, and diarrhea,^19^ whereas in the anlotinib phase III clinical study, the most common grade 3 AEs were hypertension, hyponatremia, and liver injury.^31^ Despite the combination of trametinib and anlotinib, the incidence of grade 3 hypertension did not increase, but the incidence of rash increased. Additionally, an intriguing phenomenon observed in phase Ia is that patients from dosage D experienced fewer AEs than those from dosages A-C did. The potential reasons may be attributed to data bias induced by the small sample size, as well as the missed follow-up for the 2 patients due to the COVID-19 pandemic. Overall, the toxicity profile induced by combination therapy with trametinib and anlotinib for non-G12C KRAS-mutant NSCLC patients appears to be acceptable, with manageable AEs.

The efficacy of trametinib plus anlotinib for non-G12C KRAS-mutant NSCLC patients was an important point of interest in this clinical trial. Our results suggested promising results: in phase Ia, the ORR was 69%, with a median PFS of 6.9 months. In phase Ib, the ORR was 65%, with a median PFS of 11.5 months and a median OS of 15.5 months. The overall ORR for 33 patients in phase I was 66.7%, with a median PFS of 10.3 months and a median OS of 19.7 months. In studies where KRAS-mutant NSCLC patients received the MEK inhibitor trametinib plus docetaxel, the ORR was only 12% ~ 17%, with a PFS of approximately 4.0 months.^19,50^ In other studies in which KRAS-mutant NSCLC patients received the MEK inhibitor selumetinib plus docetaxel, the ORR was 20% ~ 37%, with a PFS of 3.9 ~ 5.3 months and an OS of 8.7 ~ 9.4 months.^17,18^ Similarly, for KRAS-mutant NSCLC patients receiving anlotinib monotherapy, the ORR was 28%, the median PFS was 4.2 months, and the median OS was 8.0 months. The phase I study demonstrated a significantly favorable ORR, PFS, and OS for non-G12C KRAS-mutant NSCLC patients treated with the combination therapy of trametinib plus anlotinib. Therefore, we propose that the RTK inhibitor anlotinib may effectively prevent the development of adaptive resistance to trametinib, thereby enhancing the therapeutic efficacy of MEK inhibition.

During the clinical study, the majority of patients experienced the COVID-19 pandemic, and unfortunately, some died or were lost due to COVID-19 infection. Despite treatment interruptions due to COVID-19 infection, some patients still achieved favorable therapeutic efficacy after treatment resumed. Overall, the combination treatment involving trametinib plus anlotinib demonstrated positive therapeutic efficacy, and the coinhibition of MEK and RTKs did not increase the toxicity observed with single inhibition of MEK or RTKs. However, the study’s limitation lies in its small sample size, with 33 patients with non-G12C KRAS-mutant NSCLC included. Therefore, further validation of the therapeutic efficacy of this regimen through randomized controlled trials (RCTs) is imperative in the future. Another important consideration is the need for further investigation into the underlying mechanisms of the synergistic inhibitory effect induced by combined treatment with the MEK inhibitor trametinib and the RTK inhibitor anlotinib. A deeper understanding of the molecular basis of this synergistic antitumor effect will help establish a novel theoretical foundation for the development of new therapeutic agents, ultimately offering additional survival benefits for patients with this subtype of NSCLC.

In summary, our findings suggest that the combination of the MEK inhibitor trametinib and the RTK inhibitor anlotinib has high antitumor efficacy for KRAS-mutant NSCLC, with the underlying molecular mechanism involving the MEK/RTK-IGFBP2-RTK signaling loop. A clinical investigation involving 33 advanced non-G12C KRAS-mutant NSCLC patients suggested that trametinib plus anlotinib treatment has promising therapeutic efficacy and disease control. Overall, this study represents a comprehensive attempt from preclinical studies to clinical studies in the development of a potential therapeutic strategy through the coinhibition of MEK/RTK pathways in NSCLC patients harboring KRAS mutations.

## Materials and methods

### Cell lines and cell culture

All the KRAS-mutant NSCLC cell lines, including H23 (KRAS^*G12C*^), H358 (KRAS^*G12C*^), SW1573 (KRAS^*G12C*^), Calu1 (KRAS^*G12C*^), SK-LU-1 (KRAS^*G12D*^), SW900 (KRAS^*G12V*^), A549 (KRAS^*G12S*^), and H460 (KRAS^*Q61H*^), were purchased from the American Type Culture Collection and confirmed by STR profiling. The cells were maintained in a humidified incubator at 37 °C with 5% CO_2_ and grown in RPMI 1640 or DMEM supplemented with 10% FBS and penicillin (100 U/ml)/streptomycin (100 μg/ml) (Gibco/Thermo Fisher). All the cell lines used were negative for mycoplasma.

### Reagents and antibodies

Trametinib (S2673) was purchased from Selleck Chemicals (Houston, USA). Anlotinib was provided by Chia Tai Tianqing Pharmaceutical Group Co., Ltd. (Nanjing, China). The recombinant human IGFBP2 (rhIGFBP2) protein was purchased from R&D Systems (Cat. 674-B2-025). For in vitro treatments, trametinib and anlotinib were prepared in dimethyl sulfoxide (DMSO; Sigma‒Aldrich, S-002-M). All drugs were stored in aliquots at −80 °C until use. For in vivo treatments, 37.5 mg of anlotinib was dissolved in 1 ml of ddH_2_O as a 100× stock solution. A total of 7.5 mg of trametinib was dissolved in 1 ml of DMSO as a 100× stock solution. A TUNEL kit was purchased from Beyotime (C1088). The following antibodies were used in the western blot analysis: anti-IGFBP2 (Abcam, ab188200), anti-AKT (CST, 9272), anti-p-AKT (CST, 4060), anti-ERK (CST, 4695), anti-p-ERK (CST, 4370), anti-CyclinE2 (ABclonal, A9305), anti-CDK2 (ABclonal, A18000), anti-CyclinD1 (ABclonal, A19038), anti-PARP (ABclonal, A19596), anti-cleaved-p-FRS2 (CST, 3864S), anti-c-Myc (Abcam, ab32072), anti-ACTB (ABclonal, AC026), anti-ErbB3 (Abcam, ab255607), anti-p-ErbB3 (Abcam, ab76469), anti-FRS2 (Abcam, ab183492), anti-p-FRS2 (CST, 3864S), anti-PDGFR (Abcam, ab32570), anti-p-PDGFR (CST, 3170 T), anti-c-Kit (Abcam, ab32363), anti-p-c-Kit (CST, 2478 T), anti-SHP2 (Abcam, ab32083), anti-p-SHP2 (Abcam, ab62322), anti-VEGFR (CST, 2478 T), anti-p-VEGFR (Abcam, ab134191).

### Cell viability assays

The proliferative activity of the cells was determined via cell counting kit-8 (CCK8) assays (Dojindo, CK04), with the number of viable cells evaluated after 2 h in medium containing CCK8. The conversion of the tetrazolium salt WST-8 to formazan was measured at 450 nm via a plate reader. A previous study showed that the dose and effect can be interchanged via defined parameters in the median effect equation.^51^ The CompuSyn software developed on the basis of this theory was used to calculate the CI (combination index) between anlotinib and trametinib as previously described.^52^ Drug interactions were quantitatively determined, where CIs <1, = 1 and >1 indicate synergistic, additive and antagonistic effects, respectively.

### Clonogenic assay

For the clonogenic assay, one thousand cells were seeded in 6-well plates and treated with the indicated drugs (trametinib/anlotinib monotherapy or combination therapy). The medium was replaced every three days. When harvested, the colonies were stained with crystal violet (0.5%, w/v) and counted to calculate the relative colony formation rate. Each confirmed clone contained at least 50 cells. Colony images were scanned, and the colony area percentage was quantified via ImageJ software (National Institutes of Health, NIH).

### Flow cytometry

Briefly, 0.5 × 10^6^ cells were cultured overnight in six-well plates before treatment. For the apoptosis assay, Annexin V-FITC/PI staining (Annexin V-FITC/PI apoptosis kit, Multi Sciences, AT101C) was performed according to the manufacturer’s instructions, and a BD LSRFortessa™ Cell Analyzer (BD Biosciences, BD FACSCanto II) was used to determine the percentage of apoptotic cells. The data were analyzed via FlowJo v.10.

For cell cycle analysis, the cells were incubated with DNA staining solution and permeabilization solution according to the manufacturer’s instructions for 20 min and analyzed with a BD LSRFortessa™ Cell Analyzer (BD Biosciences, BD FACSCanto II). The results were analyzed by Modfit LT software, and the proportion of cells in different phases of the cell cycle was determined.

### Wound healing scratch assay and transwell assay

For the wound healing scratch experiment, 1 × 10^5^ cells were seeded on 6-well plates for 24 h. Scratch wounding was performed with a 200 μL pipet tip. The vehicle, anlotinib (1 μM), trametinib (10 nM) or their combination were added to each well as indicated. A phase contrast microscope (Nikon, Japan) was used to capture images at 0 h and 24 h, and the migration rate was calculated through the change in wound width. For the invasion test, the chamber was coated with 100 μL of Matrigel (Corning, USA; Cat. No.356234) overnight, and 50,000 cells were added to the upper chamber along with the corresponding drug mixture prepared with serum-free medium. The drug treatments were as indicated, including trametinib single agent (0, 10, or 25 nM), different combinations of anlotinib (0 or 1 μM) and trametinib (0 or 10 nM) or dual drugs plus rhIGFBP2 (200 ng/ml). After 24 h of treatment, the cells were stained, photographed and counted.

### RNA-seq library, protein chip assay, and bioinformatic analysis

For adaptive resistance analysis, KRAS-mutant NSCLC cells were treated with trametinib (10 nM) for 2, 5, or 10 days in 6-well plates. The library method for RNA-seq was performed according to our previous study.^29^ Briefly, cell sample lysis and total RNA isolation were performed via TRIzol reagent (Life Technologies, Inc., USA) and an RNA isolation kit (Qiagen, Germany). Next, procedures for mRNA isolation and residual genomic DNA degradation were performed, and then, 100 ng of mRNA from each sample was reverse-transcribed into cDNA and fragmented into 100--300 bp pieces. End-repair and adapter-ligation were performed via the NEBNext Ultra Directional RNA Library Prep Kit (NEB, USA). Amplification and sequencing were performed sequentially via PCR and an Illumina Next500 system (Illumina, USA). The samples were subjected to 10 cycles in a thermal cycler with PCR Master Mix supplemented with Q5 High-Fidelity DNA Polymerase (NEB, USA). The PCR products were quantified via an Agilent 2100 bioanalyzer (Agilent, USA), and standard paired-end sequencing with 75-bp reads was performed via the sequencing platform. Four KRAS-mutant NSCLC cell lines (A549, Calu-1, H460 and H23) were used for trametinib adaptive resistance analysis. Three samples from each timepoint were collected for sequencing and bioinformatic analysis. For combination analysis, A549 cells were treated with trametinib (10 nM), anlotinib (1 μM) or combination treatment for 24 h in 6-well plates. One sample for each treatment was collected for sequencing and bioinformatic analysis. Gene Ontology (GO) cluster and Kyoto Encyclopedia of Genes and Genomes (KEGG) pathway cluster analyses were performed via a public bioinformatics resource platform named the Database for Annotation, Visualization and Integrated Discovery (DAVID, https://david.ncifcrf.gov/). The R package was used to perform gene set enrichment analysis (GSEA) of the samples. For the protein chip assay, A549 cells were treated with trametinib (10 nM), anlotinib (1 μM) or combination treatment for 24 h in 6-well plates. Three samples from each treatment group were collected for subsequent angiogenesis-related protein chip detection.

### Quantitative real-time PCR

Total RNA was then extracted from the cells, and cDNA was synthesized from the RNA via HiScript III RT SuperMix for qPCR (Vazyme, R323-01). ChamQ SYBR Color qPCR Master Mix (Vazyme, Q411-02/03) was used for real-time PCR according to the manufacturer’s instructions. The expression of the target genes was normalized against that of ACTB and compared among groups via the ΔΔCT method. The sequences of primers used in this study were as follows: FGFR1-F, CCCGTAGCTCCATATTGGACA, FGFR1-R, TTTGCCATTTTTCAACCAGCG; FGFR2-F, GGTGGCTGAAAAACGGGAAG, FGFR2-R, AGATGGGACCACACTTTCCATA; IGF1R-F, AGGATATTGGGCTTTACAACCTG, IGF1R-R, GAGGTAACAGAGGTCAGCATTTT; AXL-F, GTGGGCAACCCAGGGAATATC, AXL-R, GTACTGTCCCGTGTCGGAAAG; KIT-F, CGTTCTGCTCCTACTGCTTCG, KIT-R, CCCACGCGGACTATTAAGTCT; DDR1-F, CCGACTGGTTCGCTTCTACC, DDR1-R, CGGTGTAAGACAGGAGTCCATC; PDGFRB-F, TGATGCCGAGGAACTATTCATCT, PDGFRB-R, TTTCTTCTCGTGCAGTGTCAC; VEGFR-F, GTGATCGGAAATGACACTGGAG, VEGFR-R, CATGTTGGTCACTAACAGAAGCA; EGFR-F, AGGCACGAGTAACAAGCTCAC, EGFR-R, ATGAGGACATAACCAGCCACC; ERBB2-F, TGCAGGGAAACCTGGAACTC, ERBB2-R, ACAGGGGTGGTATTGTTCAGC; ERBB3-F, GGTGATGGGGAACCTTGAGAT, ERBB3-R, CTGTCACTTCTCGAATCCACTG; ACTB-F, CATGTACGTTGCTATCCAGGC, ACTB-R, CTCCTTAATGTCACGCACGAT.

### Western blot analysis

Whole-cell lysates of the cell lines were prepared in lysis buffer (10% SDS, 1 mM DTT, and glycerin) and incubated at 100 °C for 10 min. Equal volumes of proteins were resolved via SDS‒PAGE and transferred onto PVDF membranes. After being blocked with 5% BSA (Avantor, 0332), the membrane was incubated with the following primary antibodies at 4 °C overnight. The membrane was subsequently incubated with a secondary antibody coupled with horseradish peroxidase (ABclonal, China; goat anti-rabbit, AS014) for 1 h at room temperature. Protein expression was assessed via Pierce ECL Western Blotting Substrate (Thermo Fisher Scientific) and detected via SAGECREATION (Sage Creation Science Co., Beijing).

### Mouse studies

All the mice used in this study were housed under specific pathogen-free conditions at the animal facility of Shanghai Chest Hospital at an ambient temperature of approximately 21–23 °C under a humidity of 40–60% and a light‒dark cycle of 12–12 h. All the procedures were performed with the approval of the Institutional Animal Care and Use Committee of Shanghai Chest Hospital (approval no. KS(Y)22323). Six-week-old male BALB/c athymic (nu^+^/nu^+^) mice were injected subcutaneously with ~5×10^6^ A549, H460, H23 or Calu-1 cells diluted in 100 μl of Matrigel-containing (BD Biosciences, Milan, Italy) PBS solution to generate cell line-derived xenograft models. When the tumors reached 100 ~ 150 mm^3^, the mice were randomly divided into four groups (5‒6 mice per group) to receive the following treatments (ddH_2_O, anlotinib 1.5 mg/kg, trametinib 0.3 mg/kg or the combination, oral gavage, five times per week). Monitoring of tumor growth and body weight was continued until the tumor volume reached approximately 1500mm^3^, after which the mice were sacrificed. Tumors were measured twice per week via the following formula: tumor volume=1/2 × length × (width)^2^. The maximum percent reduction from baseline was calculated for the treatment groups.

### Immunohistochemistry and immunofluorescence

The tumor tissues derived from the xenograft models were fixed with formaldehyde, embedded in paraffin and cut into slices with a thickness of 4 micrometers. HE staining was performed with hematoxylin and eosin. For Ki67 detection, after endogenous peroxidase inactivation, antigen retrieval and blocking, the sections were incubated with the corresponding primary antibodies (ABclonal, anti-Ki67, A2094, 1:200) overnight at 4 °C. The primary antibodies used in the immunofluorescence experiment were anti-IGFBP2 (Abcam, ab188200, 1:500), anti-p-ERK (CST, 4370, 1:500), and anti-c-Myc (Abcam, ab32072, 1:500). After washing with buffer, the corresponding horseradish peroxidase (HRP)-conjugated or fluorescein isothiocyanate (FITC)-conjugated secondary antibody was used for incubation. Observation under the microscope was performed on an optical microscope or a laser confocal microscope (Zeiss).

### siRNA interference

The sequence of IGFBP2 was obtained from GenBank (Accession Nos. NM_000597.3, NM_001313990.2, NM_001313992.2 and NM_001313993.2). Three sequence-specific siRNAs targeting IGFBP2 with a length of 19 nt and a 3’ dTdT overhang were designed.

All the above siRNAs were mixed with Lipofectamine 3000 and P3000 reagent (Invitrogen, L3000150) and transfected according to the manufacturer’s instructions. After 4–6 h, the transfection mixture was replaced with complete growth medium. Cell lysates were collected 24 ~ 48 h after transfection for detection of the IGFBP2 transcription level.

### Retrospective efficacy analysis of patients with KRAS-mutant NSCLC in the ALTER0303 clinical trial

ALTER0303 is a phase III clinical trial (NCT02388919) to examine the efficacy of anlotinib in advanced NSCLC (stage IIIB/IV) patients who have received chemotherapy or driver gene inhibitors for at least two lines of therapy. In total, 437 patients were included in this study, and 294 patients received anlotinib monotherapy. Among the 294 patients, 29 patients had confirmed KRAS mutations in tissue (utilizing a commercial 68-gene panel from Burning Rock Biotech) or blood (utilizing a commercial 168-gene panel from Burning Rock Biotech). Clinical information, including age, sex, smoking history, ECOG score, pathology, stage, KRAS mutation type, and prior treatment information, was analyzed. The maximum percent reduction from baseline for each KRAS-mutant NSCLC patient was calculated on the basis of the therapeutic data. Analysis of the durable response was performed for patients who achieved the therapeutic efficacy of PR. In addition, the median PFS and median OS for the 29 patients with KRAS-mutant NSCLC were calculated via the Kaplan‒Meier method.

### Design and patients in the phase I trial

We conducted a phase I trial (ClinicalTrials.gov Identifier: NCT04967079) at Shanghai Chest Hospital, Shanghai Jiao Tong University School of Medicine, following a single-center, open-label, single-cohort design. The aim of this phase I study was to determine the RP2D and assess the preliminary efficacy of a treatment regimen (trametinib plus anlotinib) in advanced NSCLC patients with non-G12C KRAS mutations. All enrolled patients were required to have at least one measurable lesion according to the Response Evaluation Criteria in Solid Tumors version 1.1 (RECIST 1.1) guidelines. The study drugs trametinib and anlotinib were orally administered once daily. Anlotinib was given from day 1 to day 14 of the 21-day cycle.

The phase I clinical study was divided into two parts: phase Ia and phase Ib. Phase Ia focused on dosage escalation, whereas phase Ib involved an expansion cohort. The dosage escalation was planned with 2 mg of trametinib in combination with the following indicated anlotinib doses: 6 mg (Dosage A), 8 mg (Dosage B), 10 mg (Dosage C), or 12 mg (Dosage D). In this dose-escalation trial, if none of the 3 subjects in the initial dosage group experienced dose-limiting toxicity (DLT), then a higher dose was explored. This process continued until a 12 mg dosage was reached. However, if 1 out of 3 subjects developed DLT in any of the indicated dosage groups, an additional 3 subjects would be recruited, resulting in a total of 6 subjects in that dosage group. If only 1 of these 6 subjects experienced DLT, it would prompt consideration for further dose escalation or termination of the trial with this dosage determined as the MTD. If more than 1 of the 6 subjects experienced DLT, it would signal exploration in a lower dosage group or termination of the entire trial.

Phase Ia aimed to enroll at least 12 patients (ultimately 13 patients) to define the RP2D. The determination of RP2D relies on several indices: first, the MTD, followed by the ORR and the mean tumor shrinkage rate. If the highest dose of anlotinib (12 mg) did not reach the MTD, the ORR would be the main index for defining the RP2D. If 2 or more dosage groups had the same ORR, the average tumor shrinkage rate would subsequently replace the MTD and ORR in determining the RP2D. Once the RP2D of the phase Ia cohort was confirmed, additional patients could be enrolled in the phase Ib cohort for expanded observation. Phase Ib included 20 patients and was aimed at evaluating the ORR. In both phase Ia and phase Ib, therapeutic efficacy evaluation was scheduled according to RECIST version 1.1 every 4–8 weeks. After the investigators’ evaluation, the assessment cycle could be extended to 12 weeks or longer because of uncontrollable factors during the treatment period. Dosing continued until PD, unacceptable side effects, withdrawal of consent, or termination of the study.

Eligible participants included advanced NSCLC patients with activating non-G12C KRAS mutations detected in either tissue or blood. The main inclusion criteria for this study were as follows: age between 18 and 75 years; confirmation of non-G12C KRAS mutation through tissue (utilizing a commercial 68-gene panel from Burning Rock Biotech) or blood (using a commercial 1021-gene panel from Gene plus Biotech); and a diagnosis of advanced or metastatic NSCLC with an ECOG PS of 0 or 1. The main exclusion criteria were untreated active brain metastases; patients harboring the KRAS^*G12C*^ mutation; and patients who received systemic antitumor therapy or radiation therapy within 28 days prior to study initiation. In addition, in phase Ia, eligibility criteria allowed for the inclusion of patients who had previously received standard therapies or were treatment naive. Treatment-naive patients were required to have refused immunotherapy/chemotherapy for individual or irresistible reasons and to be enrolled in a clinical trial. For phase Ib, only patients who had received at least 1^st^-line standard therapy were eligible. Neither phase Ia nor phase Ib patients with a history of treatments involving MEK inhibitors (trametinib, selumetinib, etc.) and RTK inhibitors (anlotinib, sorafenib, apatinib, cabozantinib, etc.) were considered ineligible. The detailed methods of the inclusion/exclusion criteria for the clinical trial are available in the supplementary protocol.

### Oversight of phase I study

The clinical study was funded by Shanghai Chest Hospital, Shanghai Jiao Tong University School of Medicine, in collaboration with Chia Tai Tianqing Pharmaceutical Group Co., Ltd., and Novartis. The study was designed by Baohui Han, who provided guidance throughout the study. The study protocol received approval from the institutional ethics review board at Shanghai Chest Hospital, Shanghai Jiao Tong University School of Medicine, in accordance with the International Council for Harmonization Good Clinical Practice guidelines, the Declaration of Helsinki, and local regulations governing clinical research (Ethical approval no. IS21117). Written informed consent was obtained from all patients enrolled in this study.

### Endpoints of phase I study

In phase Ia, the primary endpoint is the determination of the RP2D. The secondary endpoints for phase Ia include the ORR, DCR, PFS, and incidence of AEs. In phase Ib, the primary endpoint is the ORR, whereas secondary endpoints include PFS, OS, DCR, DoR, and AEs. AEs were coded according to the system organ category (SOC) and preferred terminology in MedDRA. The severity of AEs (SAEs) was graded according to the NCI Common Terminology Criteria for Adverse Events (CTCAE) version 5.0. This analysis was conducted on patients who received at least one dose of the therapeutic drugs. Safety evaluation, along with therapeutic efficacy evaluation, was performed every 4–8 weeks. The assessment cycle could be extended to 12 weeks or longer on the basis of the investigators’ evaluation.

### Statistical analysis

Three independent biological replicates were performed in all the experiments. All the statistical analyses were performed with GraphPad Prism 9 (GraphPad Software). The sample sizes and the statistical tests used are specified in the figure legends.

The therapeutic efficacy of trametinib plus anlotinib treatment was assessed according to the RECIST 1.1 guidelines. Briefly, a reduction of more than 30% in the longest diameter of a target lesion—or in the sum of the longest diameters of multiple target lesions—is defined as a PR. An increase of more than 20% is defined as PD, whereas changes that fall between PR and PD are considered SD. For lymph node lesions, measurements are based on the short diameter. Categorical variables, including binary outcomes, were summarized via frequency counts and percentages. PFS and OS outcomes were analyzed via the Kaplan‒Meier method. Descriptive summaries are reported for PFS and OS endpoints without censoring.

In phase Ia, the sample size was determined according to the “3 + 3” principle in the anlotinib ramp-up trial. As no DLTs were observed in non-G12C KRAS-mutant NSCLC patients across all dosage groups, the sample size for each anlotinib dosage was set at 3. However, for dosage C, where patients received trametinib (2 mg) plus anlotinib (10 mg), the sample size was 4 because of the simultaneous enrollment of the remaining 2 patients. Since the MTD was not reached within the indicated dosage range, the determination of RP2D was based on the ORR and average tumor shrinkage rate. The ORR was summarized via frequency counts and percentages, whereas the average tumor shrinkage rate was summarized via the percentage of the mean reduction in the length diameter of the targeted lesions.

In phase Ib, the standard-of-care for advanced NSCLC patients with non-G12C KRAS mutations treated in this setting is docetaxel plus ramucirumab (REVEL study^53^), which is associated with an ORR of up to 23%. Assuming that trametinib plus anlotinib will yield an ORR of at least 55% in this setting, a sample size of 20 evaluable patients would be sufficient for the lower bound of a 2-sided 95% CI (Clopper‒Pearson method) to exclude a benchmark ORR of 23%. Categorical variables, including binary outcomes, were summarized via frequency counts and percentages.

The response was summarized via frequency counts and percentages, alongside the exact 95% CI calculated via the Clopper‒Pearson method. The time-to-event end points were summarized via Kaplan–Meier estimates and 95% CIs. Descriptive summaries were reported for PFS/OS/DoR endpoints without censoring.

## Supplementary information

Supplementary Figures and Tables

Protocol for clinical trial

Raw WB

## Data Availability

The raw RNA-seq data can be obtained from the GEO database under accession number GSE247512. The other data that support the findings of this study are available from the corresponding author (Dr. Baohui Han: 18930858216@163.com & Dr. Hongbin Ji: hbj@sibcb.ac.cn & Hua Zhong: eddiedong8@hotmail.com) 24 months after study completion.
