# Supplementary material for: Coinhibition of the MEK/RTK pathway has high therapeutic efficacy in KRAS-mutant non-small cell lung cancer
Source: Signal Transduct Target Ther. 2025 Sep 12;10:299. doi: 10.1038/s41392-025-02382-w (PMC12426211; doi:10.1038/s41392-025-02382-w)
Supplement: Supplementary file 1 — Supplementary Figures and Tables [file 41392_2025_2382_MOESM1_ESM.docx]

Supplementary Materials for

Co-inhibition of MEK/RTK pathways induces high therapeutic efficacy in KRAS- mutant non-small cell lung cancer

Jun Lu^1,2,3,4,11^, Minjuan Hu^1,11^, Yikai Zhao^5,11^, Tianqing Chu^1^, Wei Zhang^1^, Yijia Zhou^5,6^, Xinlei Cai^7^, Jun Wu^8^, Liang Hu^5^, Chunlei Shi^1^, Liwen Xiong^1^, Aiqin Gu^1^, Huimin Wang^1^, Yanwei Zhang^1^, Yuqing Lou^1^, Runbo Zhong^1^, Zhiqiang Gao^1^, Hongyu Liu^1^, Chao Zhou^1^, Yingli Wu^9^, Liang Zhu^10^, Hua Zhong^1,3,*^, Hongbin Ji^5,6,7,*^, Baohui Han^1,2,3,*^

Correspondence to: [18930858216@163.com,](mailto:18930858216@163.com) [hbji@sibcb.ac.cn,](mailto:hbji@sibcb.ac.cn) [eddiedong8@hotmail.com](mailto:eddiedong8@hotmail.com)

**This PDF file includes:**

Figures. S1 to S11 Tables S1 to S5

1

**Figure. S1.**


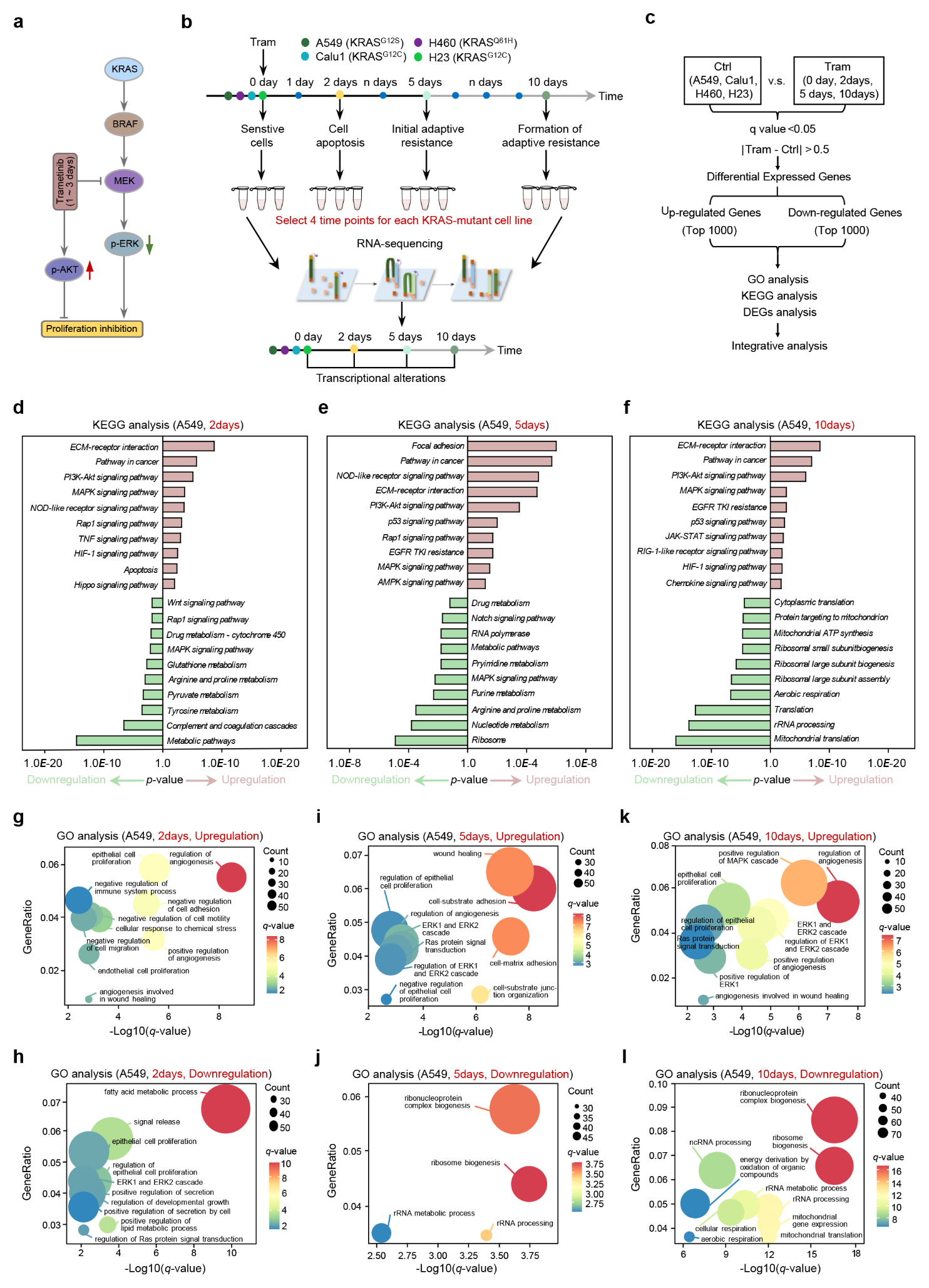


**Figure. S1. Transcriptome analysis of MEK inhibitor-trametinib adaptive resistance in KRAS-mutant NSCLC cells. a** Schematic diagram of trametinib- induced short-term proliferation inhibition in KRAS-mutant NSCLC cells. **b** Schematic diagram for formation of adaptive resistance, sample collection and RNA- sequencing. **c** Analysis flowchart of transcriptome. **d-f** KEGG analysis of top 1000 altered genes in A549 cells. (**d**) A549 cells were treated with trametinib for 2 days. (**e**) A549 cells were treated with trametinib for 5 days. (**f)** A549 cells were treated with trametinib for 10 days. **g-l** Gene ontology (GO) analysis of top 1000 altered genes in A549 cells. (**g, h**) A549 cells were treated with trametinib for 2 days. (**i, j**) A549 cells were treated with trametinib for 5 days. (**k, l**) A549 cells were treated with trametinib for 10 days.

**Figure. S2.**


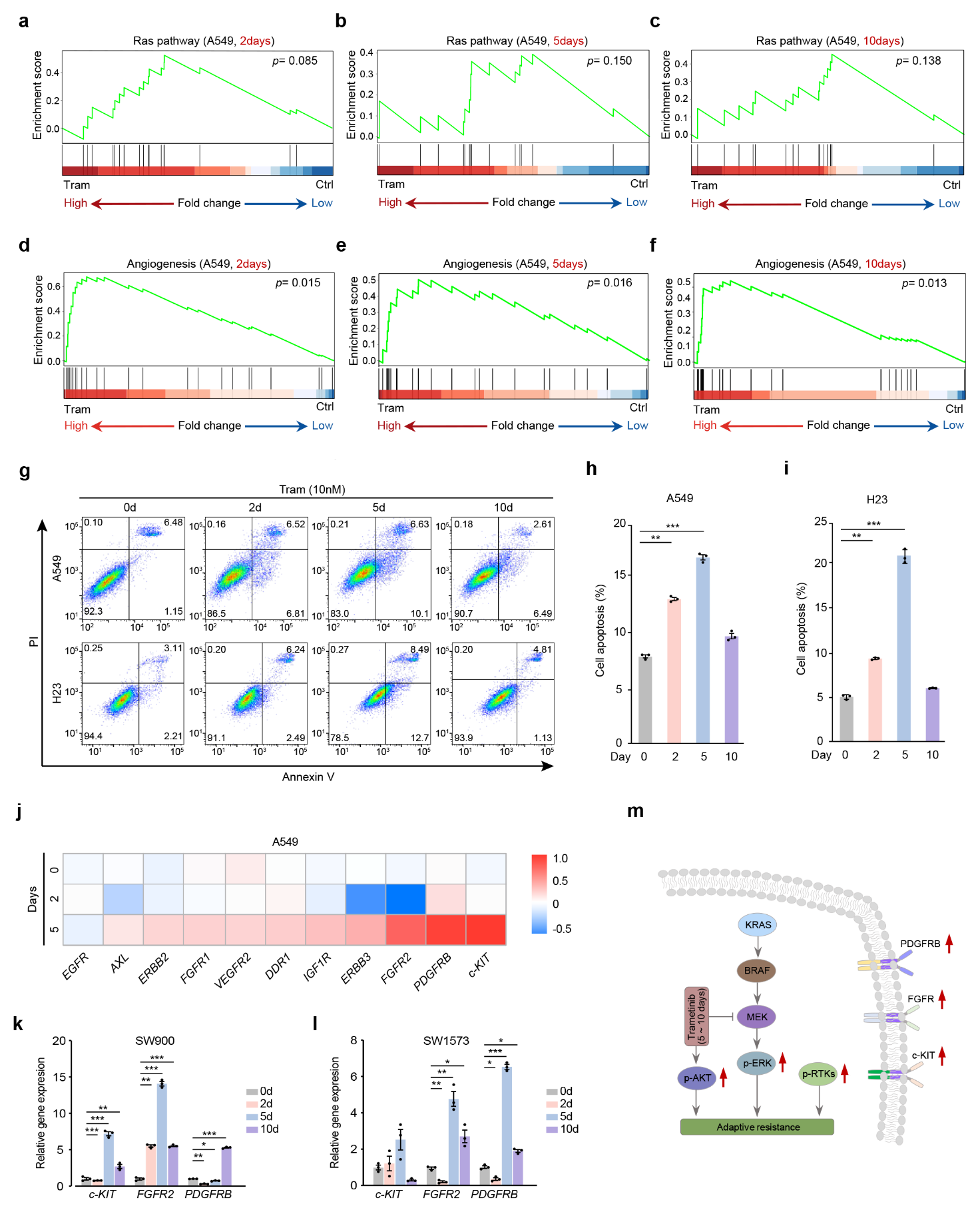


**Figure. S2. Activation of RTK pathways contributes to adaptive resistance of trametinib in KRAS-mutant NSCLC cells. a-c** GSEA of differential expressed genes in A549 cells with trametinib treatment for 2 days (**a**), 5 days (**b**) and 10 days (**c**) respectively. Ras pathway enrichment scores were shown at different time points. *n*=

3. **d-f** Angiogenesis pathway enrichment scores were shown at 2 days (**d**), 5 days (**e**) and 10 days (**f**) respectively. *n*= 3. **g-i** Apoptosis analyses of A549 and H23 cells after 10 nM trametinib treatments via Annexin V and PI staining (**g**) and related statistical analyses of A549 cells (**h**) and H23 cells (**i**). *n*= 3. **j** Heat map showing the mRNA levels of various RTKs in A549 cells after 10 nM trametinib treatments detected by RT-qPCR. **k, l** mRNA levels of c-Kit, FGFR2 and PDGFRB in SW900 (**k**) and SW1573 (**l**) cells after 10 nM trametinib treatments detected by RT-qPCR. *n*= 3. **m** Schematic diagram for adaptive resistance mechanism of trametinib in KRAS-mutant NSCLC cells. Statistical analyses were performed using Student’s t test, two-tailed. Data were presented as mean ± SEM. **P*＜0.05, ***P*＜0.01, ****P*＜0.001.

**Figure. S3.**


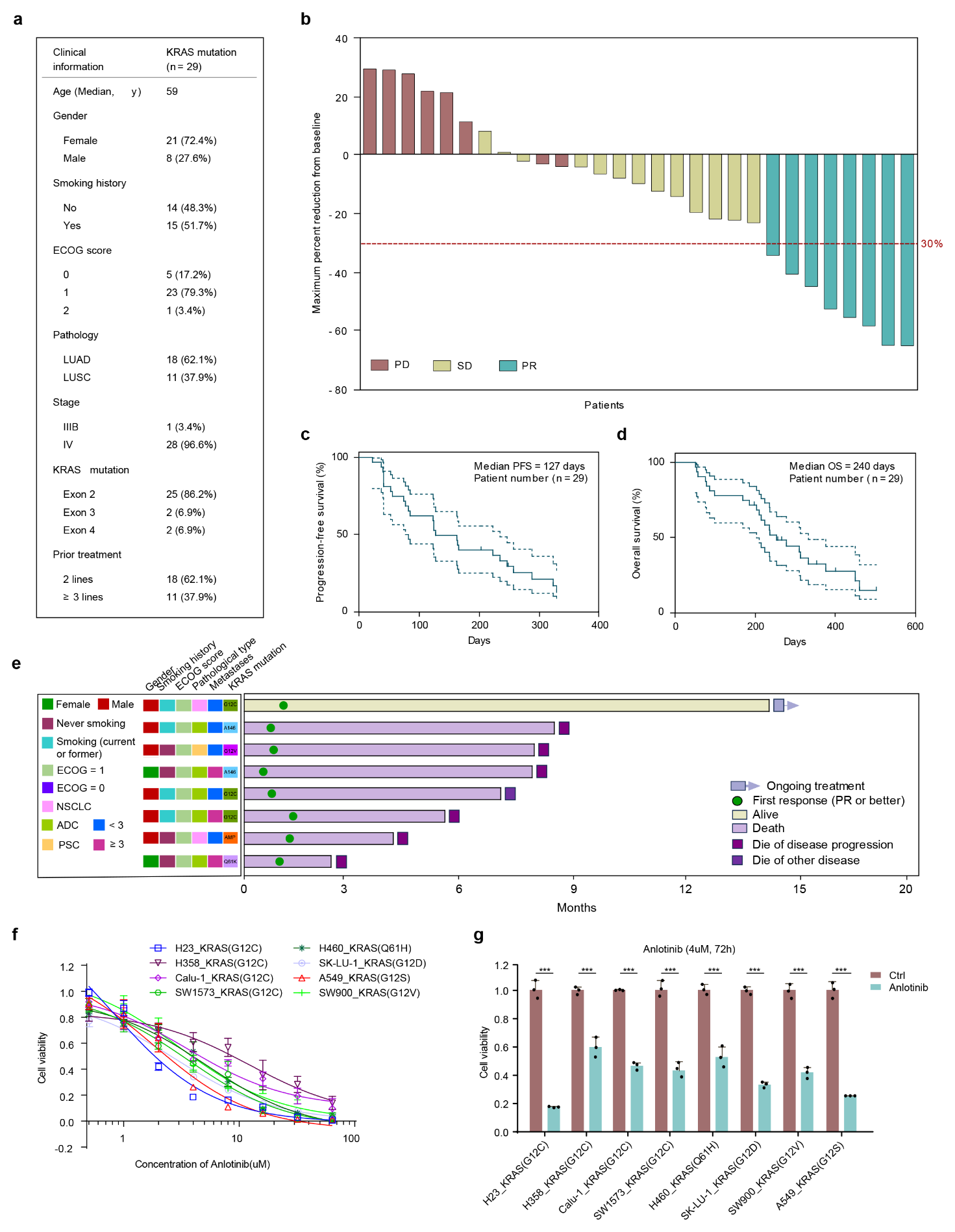


**Figure. S3. pan-RTKs inhibitor-anlotinib has potential therapeutic efficacy in pan-KRAS mutant NSCLC. a** Baseline characteristics of the 29 advanced NSCLC patients harboring various KRAS mutations enrolled in ALTER0303 study. All patients received anlotinib monotherapy during the clinical trial. **b** Waterfall plots showing the percentages of tumor regression in the 29 KRAS-mutant NSCLC patients who received anlotinib monotherapy. **c** Kaplan-Meier plots of progression-free survival (PFS). *n*= 29. **d** Kaplan-Meier plots of overall survival (OS). *n*= 29. **e** Duration of response analysis of the 29 KRAS-mutant NSCLC patients. Clinical information of gender, smoking history, ECOG score, pathological type, metastases and KRAS mutation for each patient was shown. **f** Cell viability curves of different KRAS-mutant lung cancer cells (including H23, A549, H358, H460, Calu-1, SW900, SW1573 ad SK-LU-1) treated with increasing concentrations of anlotinib for 72 hours. *n*= 3. **g** Cell viabilities of different KRAS-mutant lung cancer cells treated with 1 uM anlotinib for 72 hours. *n*= 3. Statistical analyses were performed using Student’s t test, two-tailed. Data were presented as mean ± SEM. ****P*＜0.001.

**Figure. S4.**


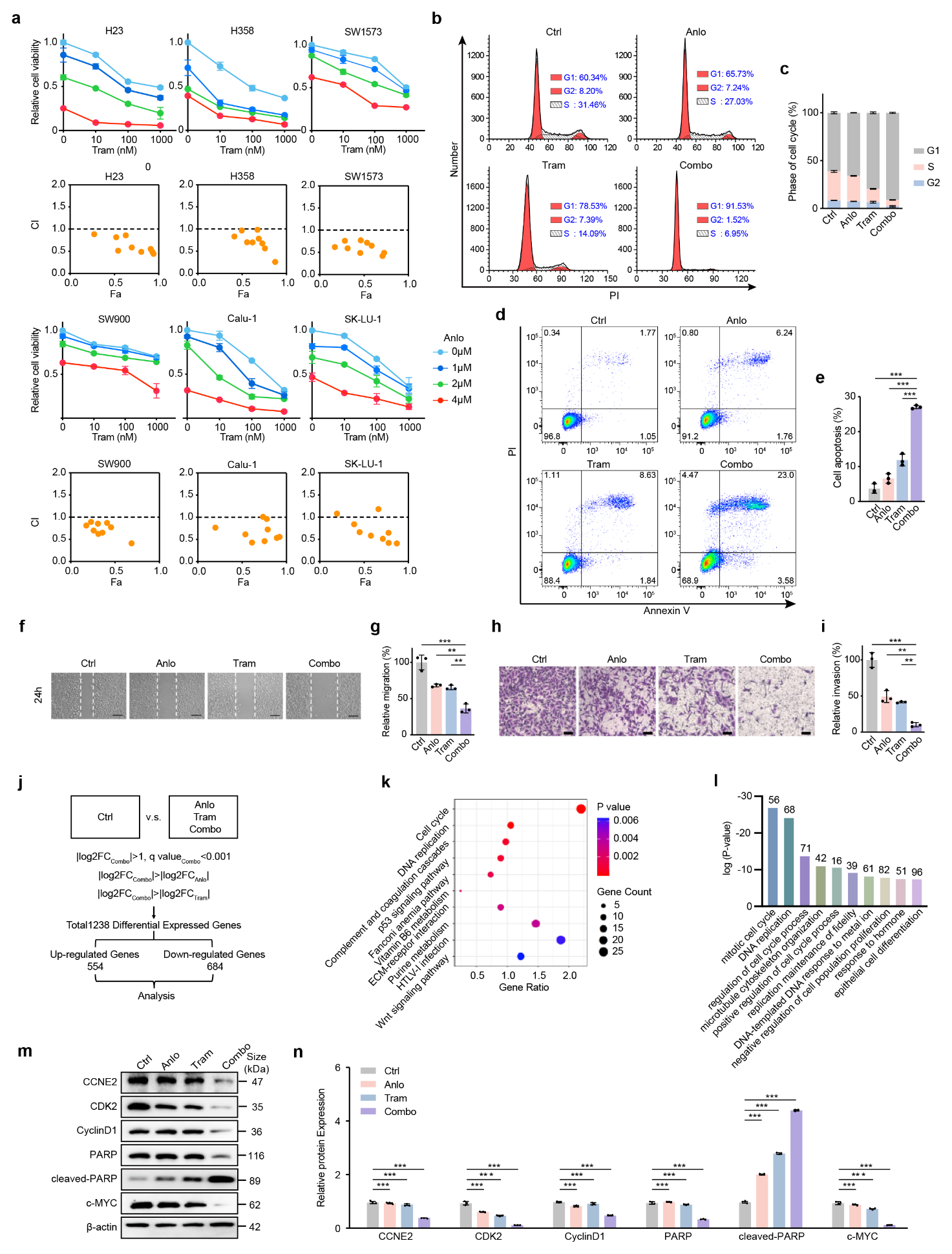


**Figure. S4. Combined trametinib and anlotinib treatment demonstrates potent synergistic anti-tumor activity in KRAS-mutant NSCLC cells *in vitro*. a** Viability of human KRAS-mutant NSCLC cell lines (H23, H358, SW1573, SW900, Calu-1, SK-LU-1) after 3-day of trametinib (10nM) and anlotinib (1μM) combination treatments. The dot graph showed the synergistic effect of the two drug combinations, CI < 1, synergism; CI = 1, additivity; CI > 1, antagonism. **b, c** Cell cycle analyses of A549 cells after 48-hour of trametinib (10nM), or anlotinib (1μM), or combination treatments via PI staining (**b**) and the quantification of cell cycle phases (**c**). **d, e** Apoptosis analyses of A549 cells after 48-hour of trametinib (10nM) or anlotinib (1μM) or combination treatments via Annexin V and PI staining (**d**) and related statistical analyses (**e**). **f, g** Cell migration (**f**) and statistical analyses (**g**) of A549 cells measured by wound healing assay after 24-hour of trametinib (10nM) or anlotinib (1μM) or combination treatments. Scale bar: 100 μm. **h, i** Boyden chamber transwell invasion (**h**) and related statistical analyses (**i**) of A549 cells after 24-hour of trametinib (10nM) or anlotinib (1μM) or combination treatments. Scale bar: 50 μm. **j** Schematic overview of RNA-seq data analyses. **k** Representative dot plots of 10 significantly enriched KEGG pathways in A549 cells after 24-hour of trametinib (10nM) or anlotinib (1μM) or combination treatments. **l** Top 10 gene ontology (GO) enrichments. **m, n** Western blot analyses of cell cycle-related proteins in A549 cells after 48-hour of trametinib (10nM) or anlotinib (1μM) or combination treatments (**m**) and related statistical analyses (**n**). Statistical analyses were performed using Student’s t test, two-tailed. Data were presented as mean ± SEM. *n*= 3, ***P*＜0.01, ****P*＜0.001.


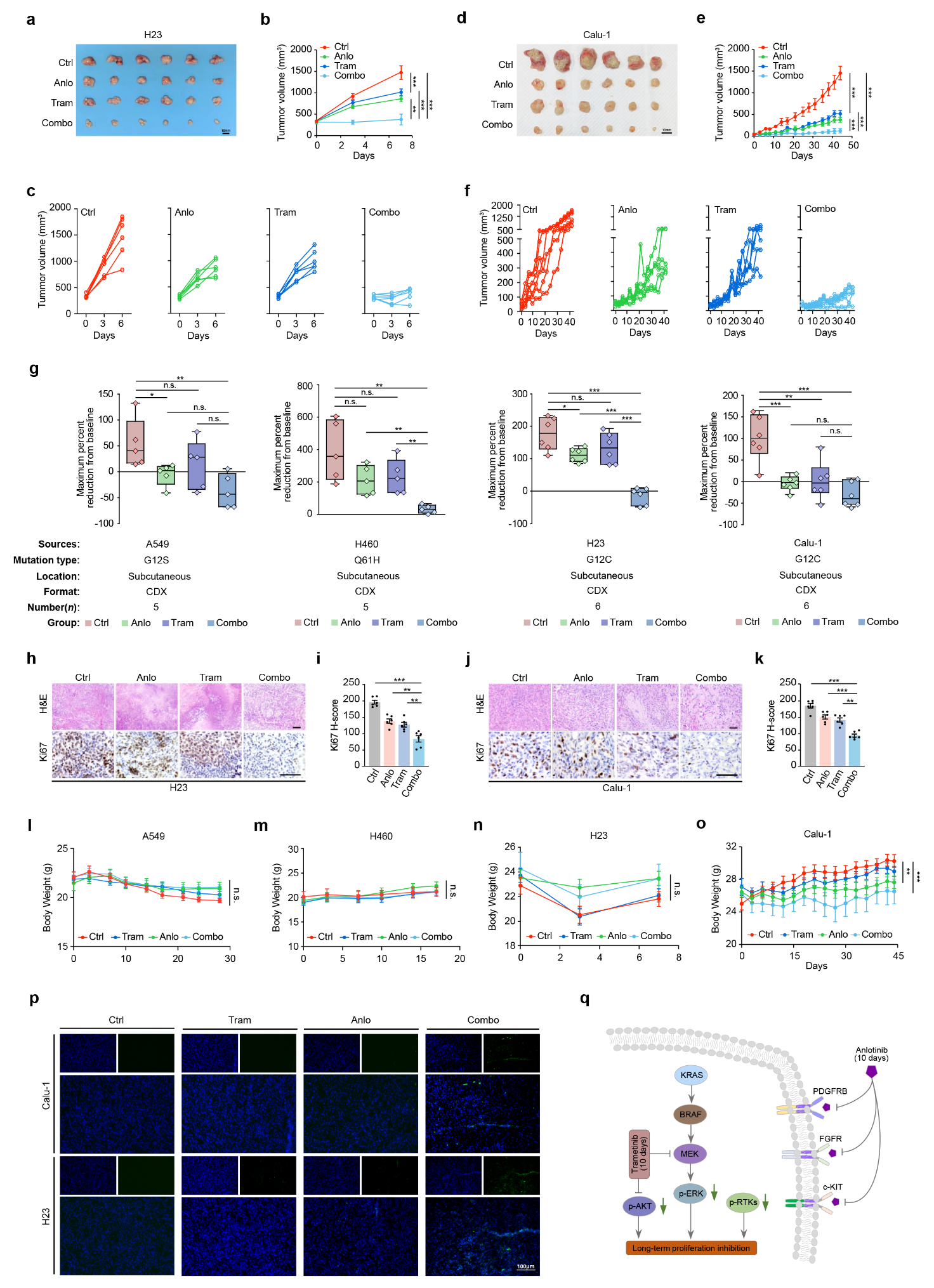
**Figure. S5.**

**Figure. S5. Combined trametinib and anlotinib treatment exhibits synergistic anti-tumor activity in xenograft models of KRAS-mutant NSCLC. a-c** H23 cells xenograft models were treated with anlotinib (1.5mg/kg), trametinib (0.3mg/kg) or combination. Gross inspection of H23 xenograft model (**a**), and the tumor growth curves for different groups (**b**) as well as independent xenograft tumor (**c**) were shown. *n*= 6 for each group. **d-f** Calu-1 cells xenograft models were treated with anlotinib (1.5mg/kg), trametinib (0.3mg/kg) or combination. (**d**) Tumor gross inspection of Calu-1 xenograft models. Tumor growth curves of Calu-1 xenograft model for different groups (**e**) as well as independent xenograft tumor (**f**). *n*= 6 for each group. **g** Analysis of maximum percent reduction from baseline for treatment groups. Each symbol represents one mouse. Source and mutation type of cell line, location and format of xenograft tumor, and numbers of each group were shown. *n*= 5 for A549 and H460 xenograft models. *n*= 6 for H23 and Calu-1 xenograft models. **h, i** Pathological analyses of H&E staining and Ki67 staining (**h**) as well as statistical analyses of Ki67 staining (**i**) for H23 xenograft tumors with indicated treatments were shown. Scale bar, 100 µm. *n*= 6. **j, k** Pathological analyses (**j**) and statistical analyses (**k**) of Calu-1 xenograft tumors with indicated treatments were shown. Scale bar, 100 µm. *n*= 6. **l-o** Changes of body weights in A549 (**l**), H460 (**m**), H23 (**n**), and Calu-1 (**o**) xenograft mice, respectively. *n*= 5 for A549 and H460 xenograft models. Scale bar, 100 µm. *n*= 6 for H23 and Calu-1 xenograft models. **p** TUNEL staining in tumor tissue from H23 and Calu-1 xenograft tumors with indicated treatments were shown. *n*= 6 for H23 and Calu-1 xenograft models. Scale bar: 100 µm. **q** Schematic diagram for co-inhibition of MEK/RTKs pathways induces long-term proliferation inhibition for KRAS-mutant NSCLC. Statistical analyses were performed using Student’s t test, two-tailed. Data were presented as mean ± SEM. **P*＜0.05, ***P*＜0.01, ****P*＜0.001.

**Figure. S6.**


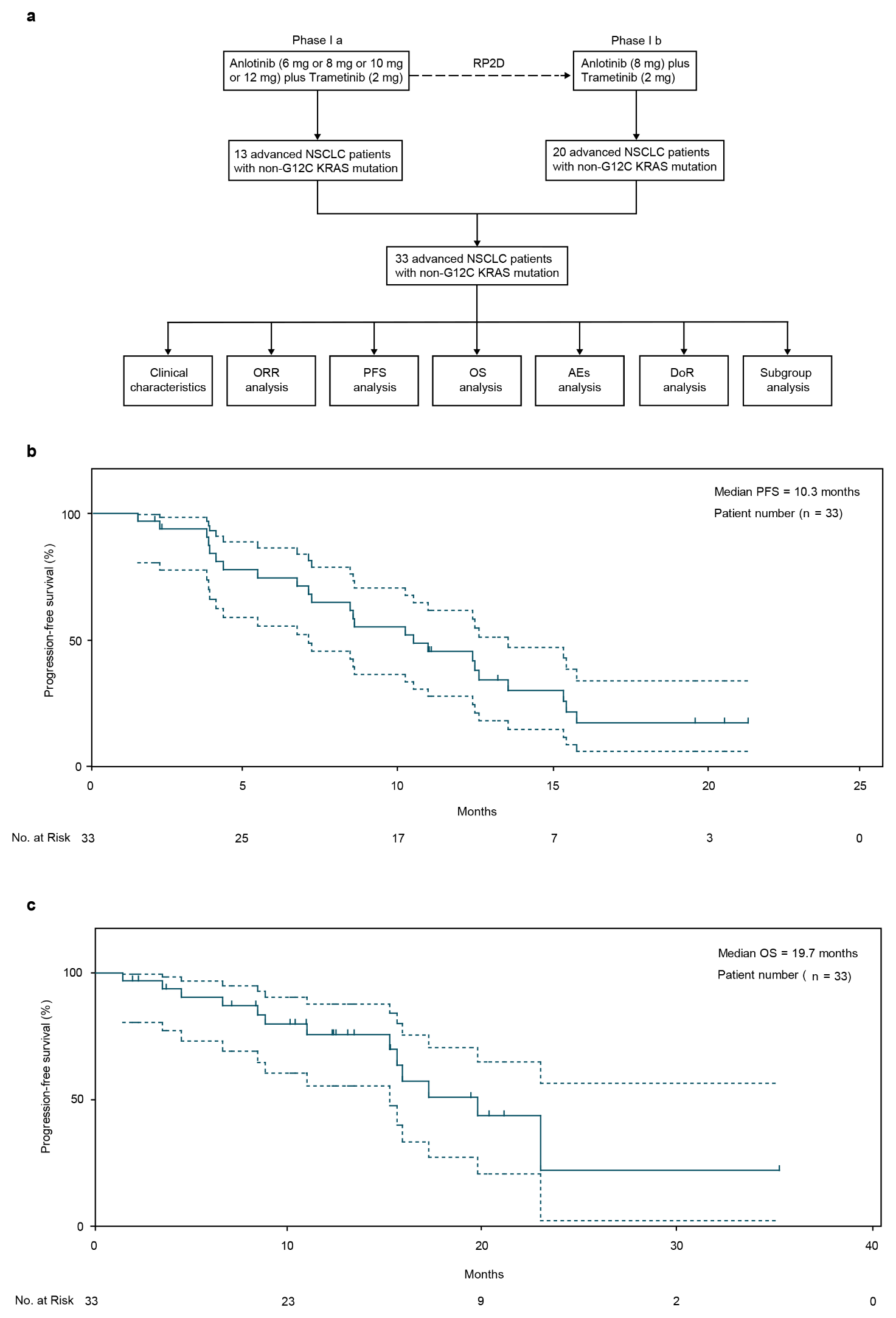


**Figure. S6. Integrative analysis for phase I clinical study. a** Flowchart of integrative analysis on phase I clinical study. **b** Kaplan-Meier plots of progression- free survival (PFS). **c** Kaplan-Meier plots of overall survival (OS).

**Figure. S7.**


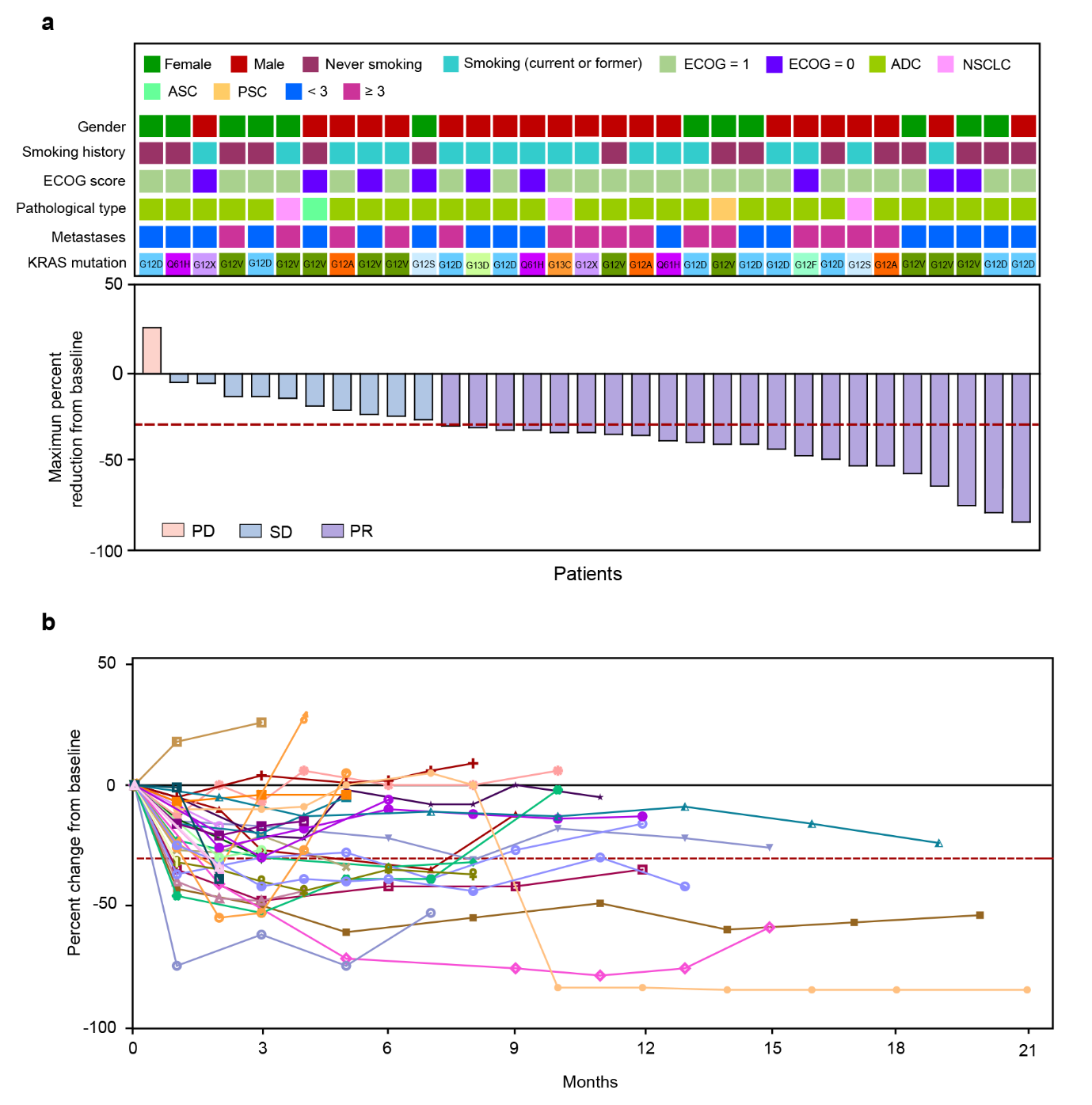


**Figure. S7. Therapeutic efficacy analysis of the 33 enrolled non-G12C KRAS mutant lung cancer patients. a** Waterfall plots showing the percentages of tumor regression/progression in phase I clinical study (phase Ia + phase Ib). **b** Percent change from baseline over time was shown for all enrolled 33 patients.

**Figure. S8.**


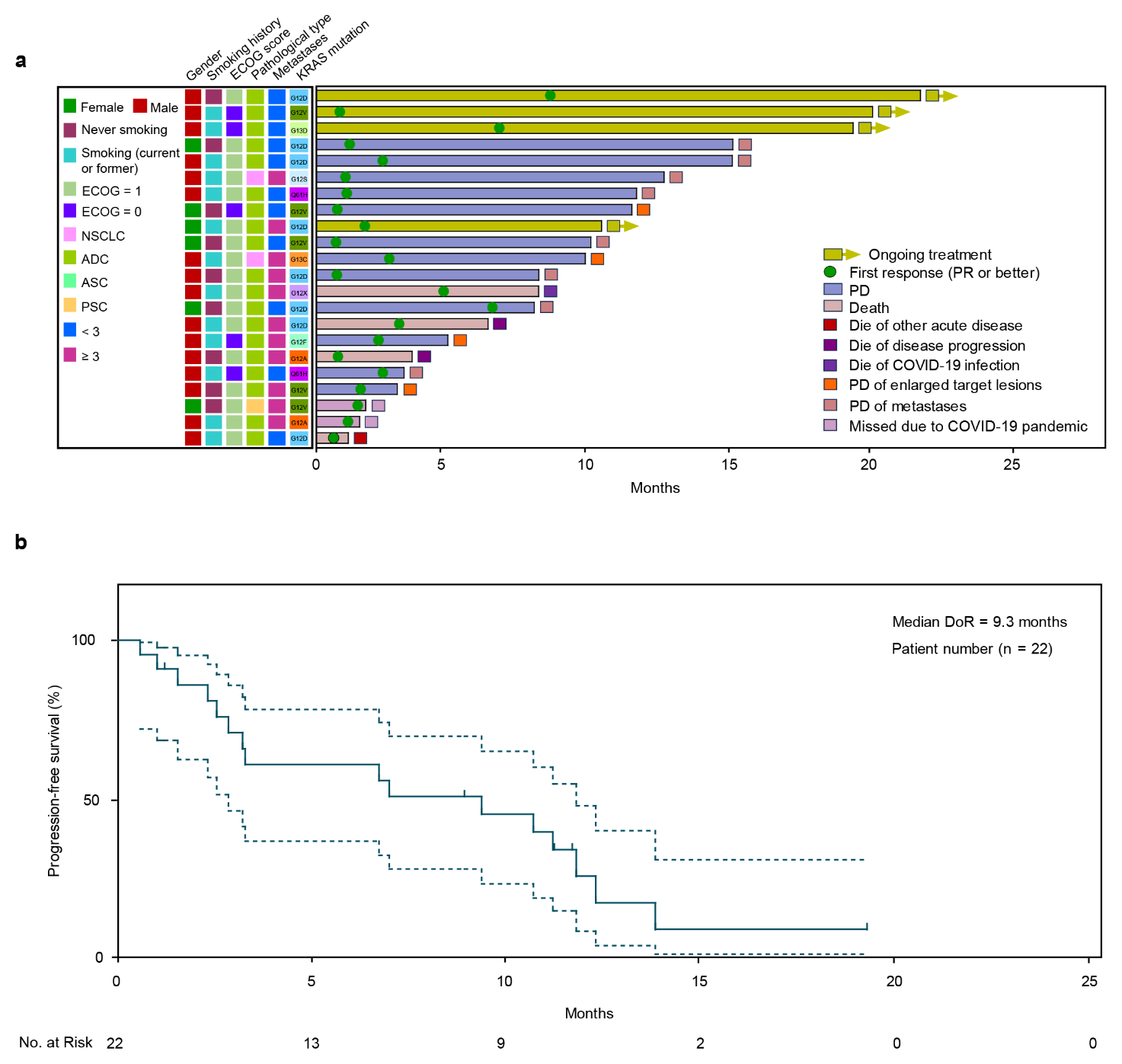


**Figure. S8. Duration of overall response analysis of the 33 enrolled non-G12C KRAS mutant lung cancer patients. a** Duration of overall response (DoR) analysis of phase I clinical study (phase Ia + phase Ib). **b** Kaplan-Meier plots of duration of overall response (DoR).

**Figure. S9.**


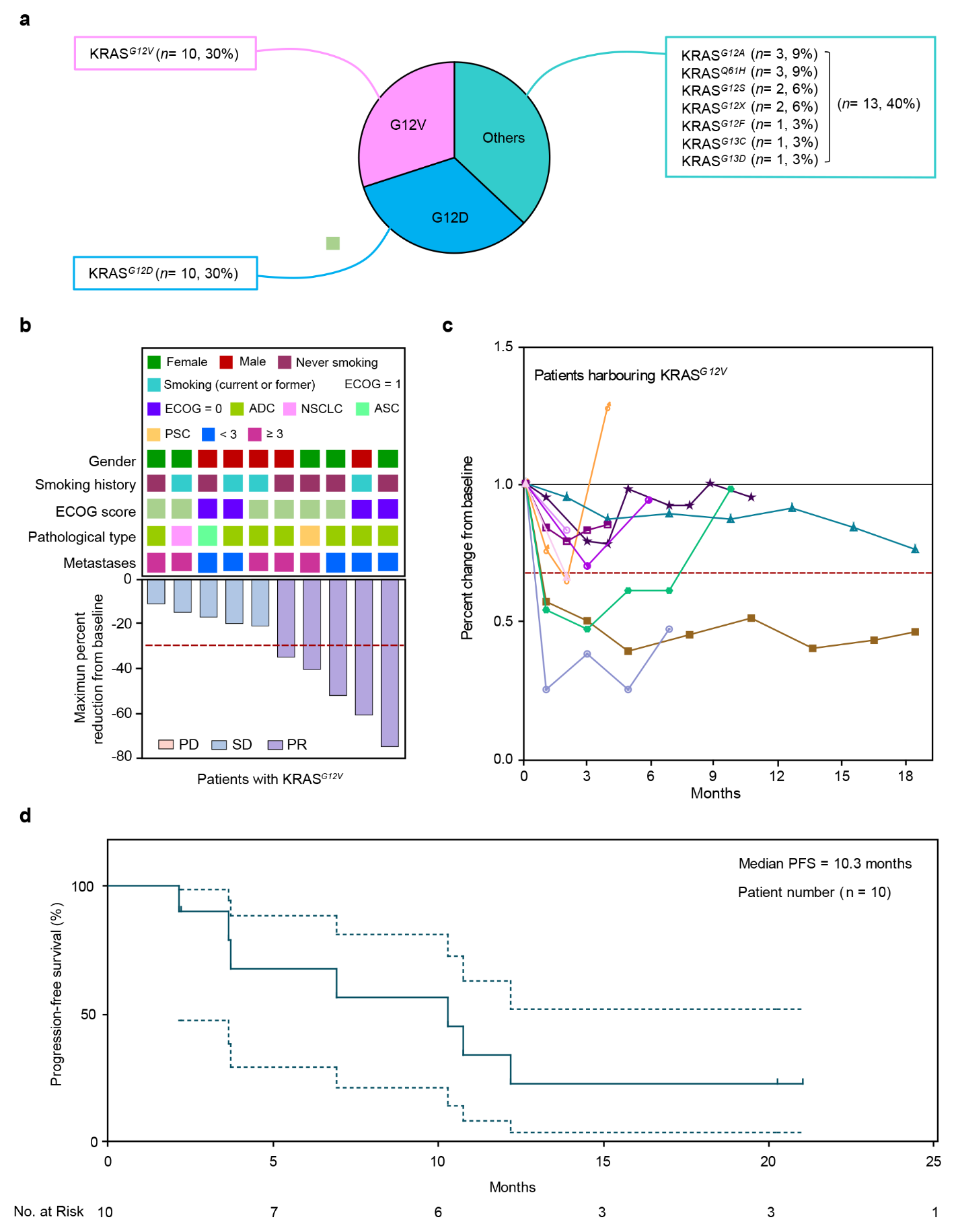


**Figure. S9. Analysis of the therapeutic efficacy of trametinib plus anlotinib in KRAS*^G12V^* mutation subtypes. a** Analysis of KRAS-mutant subtypes. **b** Waterfall plots for percentage of tumor reductions on the patients harboring KRAS*^G12V^* mutation. **c** Change in sum of largest tumor diameters over time for the patients harboring KRAS*^G12V^* mutation. **d** Kaplan-Meier plots of progression-free survival (PFS).

**Figure. S10.**


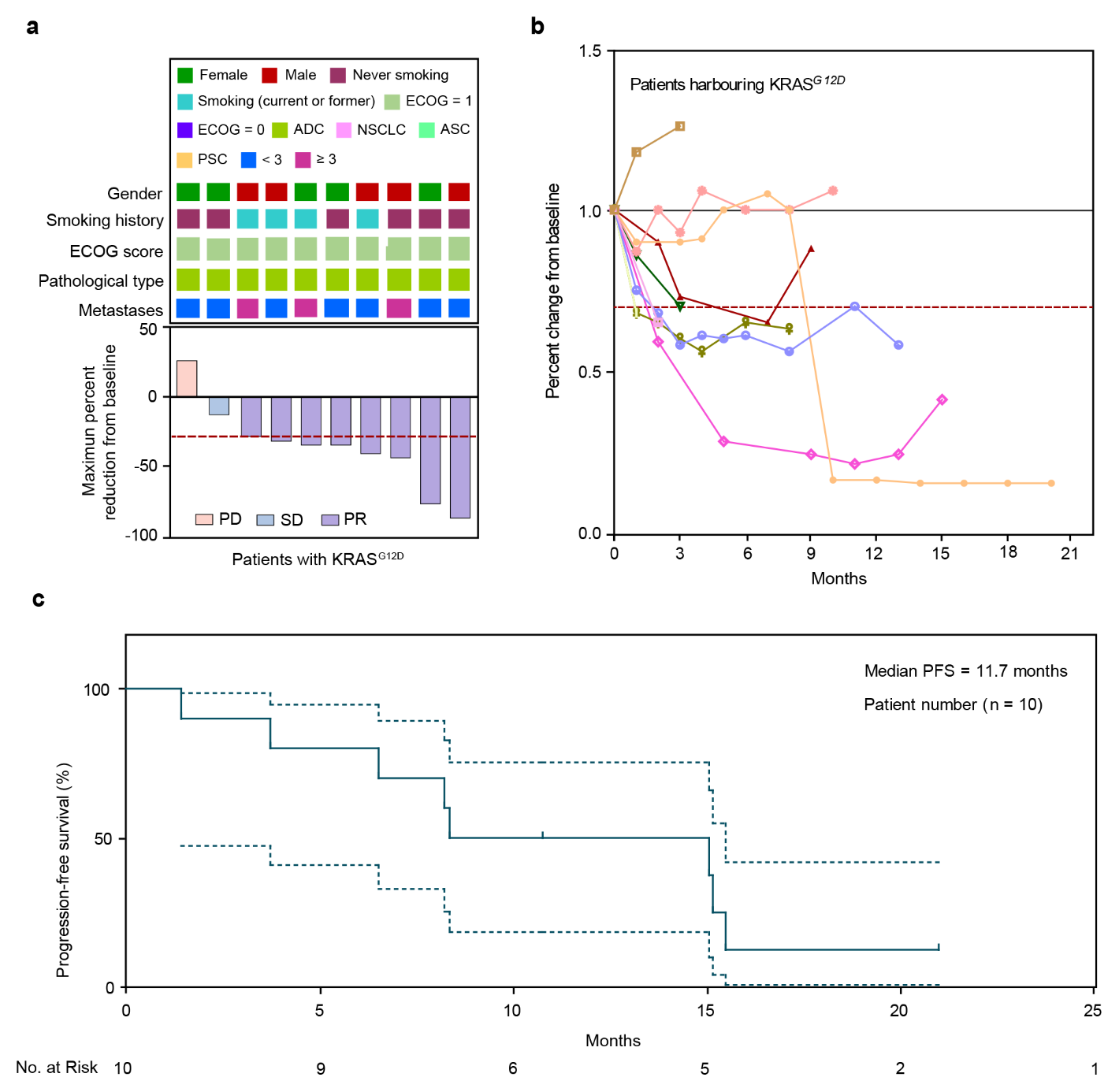


**Figure. S10. Analysis of the therapeutic efficacy of trametinib plus anlotinib strategy in KRAS*^G12D^* mutation subtypes. a** Waterfall plots for percentage of tumor reductions on the patients harboring KRAS*^G12D^* mutation. **b** Change in sum of largest tumor diameters over time for the patients harboring KRAS*^G12D^* mutation. **c** Kaplan- Meier plots of progression-free survival (PFS).

**Figure. S11.**


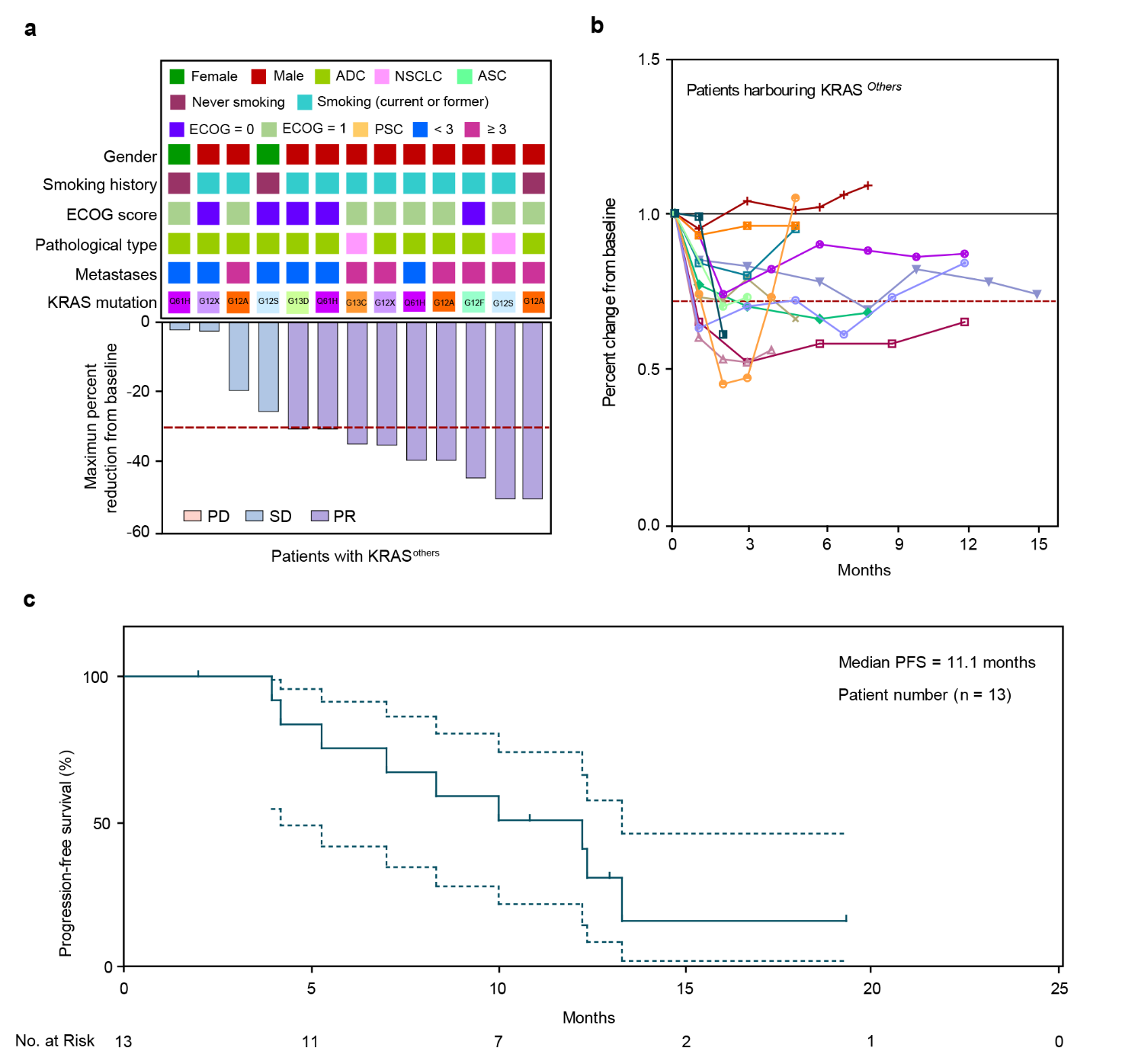


**Figure. S11. Analysis of the therapeutic efficacy of trametinib plus anlotinib strategy in KRAS*others* (KRAS*G12A, G12F, G12S, G12X, G13C, G13D, Q61H*) mutation subtypes. a** Waterfall plots for percentage of tumor reductions on the patients harboring other KRAS mutation (KRAS*^G12A,^ ^G12F,^ ^G12S,^ ^G12X,^ ^G13C,^ ^G13D,^ ^Q61H^*). **b** Change in sum of largest tumor diameters over time for the patients harboring other KRAS mutation (KRAS*^G12A,^ ^G12F,^ ^G12S,^ ^G12X,^ ^G13C,^ ^G13D,^ ^Q61H^*). **c** Kaplan-Meier plots of progression-free survival (PFS).

**Table S1. Subgroup analysis of ORR and PFS for phase Ia.**

|  | **ORR** | **PFS, months (95% CI)** | **HR** |
| --- | --- | --- | --- |
| **Gender** |  |  |  |
| **Female** *vs.* **Male** | **50%** *vs.* **78%** | **Undefined** *vs.* **6.9 (0.09 – 1.82)** | **0.41** |
| **Smoking history** |  |  |  |
| **No** *vs.* **Yes** | **50%** *vs.* **86%** | **8.4** *vs.* **6.9 (0.15 – 2.47)** | **0.60** |
| **ECOG score** |  |  |  |
| **0** *vs.* **1** | **66.7%** *vs.* **70%** | **3.9** *vs.* **15.1 (1.03 – 66.8)** | **8.42** |
| **Pathology** |  |  |  |
| **ADC** *vs.* **Other** | **72.7%** *vs.* **50%** | **8.4** *vs.* **3.7 (0 – 2.20)** | **0.03** |

**KRAS mutation**

**Exon 2** *vs.* **Other 72.7%** *vs.* **50% 8.4** *vs.* **3.9 (0 – 5.13) 0.20**

**Prior treatment**

| **0 line** *vs.* **≥ 1 line** | **66.7%** *vs.* **71.4%** | **5.3** *vs.* **Undefined (0.63 – 11.22)** | **2.65** |
| --- | --- | --- | --- |
| **Number of metastases** |  |  |  |
| **< 3** *vs.* **≥ 3** | **66.7%** *vs.* **71.4%** | **3.8** *vs.* **8.4 (0.54 – 9.53)** | **2.27** |

**ORR, objective response rate; PFS, progression-free survival; ADC, adenocarcinoma; ECOG, Eastern Cooperative Oncology Group.**

**Table S2. Subgroup analysis of ORR and PFS for in phase Ib.**

|  | **ORR** | **PFS, months (95% CI)** | **HR** |
| --- | --- | --- | --- |
| **Gender** |  |  |  |
| **Female** *vs.* **Male** | **50%** *vs.* **75%** | **12.3** *vs.* **10.4 (0.35 – 2.58)** | **0.96** |
| **Smoking history** |  |  |  |
| **No** *vs.* **Yes** | **66.7%** *vs.* **63.6%** | **12.2** *vs.* **10.7 (0.43 – 3.14)** | **1.17** |
| **ECOG score** |  |  |  |
| **0** *vs.* **1** | **50%** *vs.* **71.4%** | **Undefined** *vs.* **9.2 (0.10 – 0.76)** | **0.28** |
| **Pathology** |  |  |  |
| **ADC** *vs.* **Other** | **64.7%** *vs.* **66.7%** | **12.2** *vs.* **10 (0.10 – 2.22)** | **0.46** |

**KRAS mutation**

**Exon 2** *vs.* **Other 62.5%** *vs.* **75% 10.5** *vs.* **12.3 (0.50 – 4.60) 1.51**

**Prior treatment**

| **1 line** *vs.* **≥ 2 line** | **63.6%** *vs.* **66.7%** | **12.2** *vs.* **10.3 (0.22 – 1.62)** | **0.60** |
| --- | --- | --- | --- |
| **Number of metastases** |  |  |  |
| **< 3** *vs.* **≥ 3** | **58.3%** *vs.* **75%** | **12.4** *vs.* **6.8 (0.03 – 0.38)** | **0.10** |

**ORR, objective response rate; PFS, progression-free survival; ADC, adenocarcinoma; ECOG, Eastern Cooperative Oncology Group.**

**Table S3. Integrative analysis of patient characteristics for phase Ia plus phase Ib.**

| **Clinical information** | **KRAS mutation (excluding KRAS*G12C*) (*n*= 33)** |
| --- | --- |
| **Age (Median, y）**  **Gender** | **61.8** |
| **Female** | **12 (36.4%)** |
| **Male** | **21 (63.6%)** |

**Smoking history**

**No 15 (45.5%)**

**Yes 18 (54.5%)**

**ECOG score**

| **0** | **9 (27.3%)** |
| --- | --- |
| **1** | **24 (72.7%)** |
| **Pathology** |  |
| **ADC** | **28 (84.8%)** |
| **NSCLC-NOS** | **3 (9.1%)** |
| **ASC** | **1 (3.0%)** |
| **PSC** | **3 (3.0%)** |
| **KRAS mutation** |  |
| **Exon 2** | **28 (84.8%)** |
| **Exon 3** | **2 (6.1%)** |
| **Other exons** | **3 (9.1%)** |
| **Prior treatment** |  |
| **0 line** | **6 (18.2%)** |
| **1 line** | **12 (36.4%)** |
| **2 lines** | **13 (39.4%)** |
| **≥ 3 lines** | **2 (6.1%)** |

**Number of metastases**

**< 3 18 (54.5%)**

**≥ 3 15 (45.5%)**

**ADC, adenocarcinoma; ASC, adenosquamous carcinoma; PSC, pulmonary sarcomatoid carcinoma; NSCLC-NOS, non-small cell lung cancer, not otherwise specified; ECOG, Eastern Cooperative Oncology Group.**

**Table S4. Integrative analysis of adverse events for phase Ia and Ib.**

| **Adverse events** | **Grade 1** | **Grade 2** | **Grade 3** | **Grade 4 & 5** | **Total** |
| --- | --- | --- | --- | --- | --- |
| **Rash, n (%)** | 12(44) | 9(33) | 6(22) | 0(0) | 27(82) |
| **Diarrhea, n (%)** | 10(56) | 6(33) | 2(11) | 0(0) | 18(55) |
| **Decreased appetite, n (%)** | 7(50) | 7(50) | 0(0) | 0(0) | 14(42) |
| **Hypertension, n (%)** | 5(42) | 6(50) | 1(8) | 0(0) | 12(36) |
| **Fatigue, n (%)** | 10(83) | 1(8) | 1(8) | 0(0) | 8(36) |
| **Liver injury, n (%)** | 9(100) | 0(0) | 0(0) | 0(0) | 9(27) |
| **Oral mucositis, n (%)** | 2(25) | 6(75) | 0(0) | 0(0) | 8(24) |
| **Weight Loss, n (%)** | 6(100) | 0(0) | 0(0) | 0(0) | 6(18) |
| **Bleeding, n (%)** | 5(100) | 0(0) | 0(0) | 0(0) | 5(15) |
| **Hyperuricemia, n (%)** | 5(100) | 0(0) | 0(0) | 0(0) | 5(15) |
| **Hypoproteinemia, n (%)** | 5(100) | 0(0) | 0(0) | 0(0) | 5(15) |
| **Hyponatremia, n (%)** | 3(100) | 0(0) | 0(0) | 0(0) | 3(9) |
| **Hematuria, n (%)** | 1(50) | 1(50) | 0(0) | 0(0) | 2(6) |
| **Anemia, n (%)** | 2(100) | 0(0) | 0(0) | 0(0) | 2(6) |
| **Hyperthyroid, n (%)** | 2(100) | 0(0) | 0(0) | 0(0) | 2(6) |
| **Hematochezia, n (%)** | 2(100) | 0(0) | 0(0) | 0(0) | 2(6) |
| **Hypothyroidism, n (%)** | 0(0) | 1(100) | 0(0) | 0(0) | 1(3) |
| **Pyrexia, n (%)** | 1(100) | 0(0) | 0(0) | 0(0) | 1(3) |
| **Paronychia, n (%)** | 1(100) | 0(0) | 0(0) | 0(0) | 1(3) |
| **Emesis, n (%)** | 1(100) | 0(0) | 0(0) | 0(0) | 1(3) |
| **Alopecias, n (%)** | 1(100) | 0(0) | 0(0) | 0(0) | 1(3) |
| **Retinal cell exfoliation, n (%)** | 1(100) | 0(0) | 0(0) | 0(0) | 1(3) |
| **Dizziness, n (%)** | 1(100) | 0(0) | 0(0) | 0(0) | 1(3) |
| **Emesis** | 1(100) | 0(0) | 0(0) | 0(0) | 1(3) |
| **Epistaxis, n (%)** | 1(100) | 0(0) | 0(0) | 0(0) | 1(3) |

|  | **ORR** | **PFS, months (95% CI)** | **HR** |
| --- | --- | --- | --- |
| **Gender** |  |  |  |
| **Female** *vs.* **Male** | **50%** *vs.* **80%** | **12.4** *vs.* **8.4 (0.31 – 1.59)** | **0.70** |
| **Smoking history** |  |  |  |
| **No** *vs.* **Yes** | **60%** *vs.* **72.2%** | **11.2** *vs.* **10.0 (0.38 – 1.91)** | **0.85** |
| **ECOG score** |  |  |  |
| **0** *vs.* **1** | **55.6%** *vs.* **70.8%** | **12.2** *vs.* **10.0 (0.27 – 1.55)** | **0.65** |
| **Pathology** |  |  |  |
| **ADC** *vs.* **Other** | **67.9%** *vs.* **60%** | **10.7** *vs.* **10 (0.08 – 1.49)** | **0.35** |
| **KRAS mutation** |  |  |  |
| **Exon 2** *vs.* **Other** | **64.3%** *vs.* **50%** | **10.3** *vs.* **12.2 (0.50 – 4.60)** | **1.51** |
| **Prior treatment** |  |  |  |
| **1 line** *vs.* **≥ 2 line** | **58.3%** *vs.* **73.3%** | **12.2** *vs.* **10.3 (0.23 – 1.38)** | **0.56** |
| **Number of metastases** |  |  |  |
| **< 3** *vs.* **≥ 3** | **61.1%** *vs.* **73.3%** | **12.2** *vs.* **7 (0.19 – 1.14)** | **0.47** |

**Table S5. Integrative subgroup analysis of ORR and PFS for phase Ia and Ib.**

**ORR, objective response rate; PFS, progression-free survival; ADC, adenocarcinoma; ECOG, Eastern Cooperative Oncology Group.**
