# Supplementary material for: Coinhibition of the MEK/RTK pathway has high therapeutic efficacy in KRAS-mutant non-small cell lung cancer
Source: Signal Transduct Target Ther. 2025 Sep 12;10:299. doi: 10.1038/s41392-025-02382-w (PMC12426211; doi:10.1038/s41392-025-02382-w)
Supplement: Supplementary file 2 — Protocol for clinical trial [file 41392_2025_2382_MOESM2_ESM.pdf]

This supplement contains the following items:

1. Original protocol, final protocol, summary of changes.

2. Original statistical analysis plan, final statistical analysis plan, summary of changes.

Clinical approval No.: IS2117 (version 1.0)

Protocol No.: ATRAS-LC-1.0

Version No. and Date: 1.0/2021.02.21

Combined MEK Inhibitor Trametinib and RTK Inhibitor Anlotinib Therapy in non-G12C KRAS-Mutant Lung Cancer patients

Study Protocol

Sponsor: Shanghai Chest Hospital, Shanghai Jiao Tong University School of Medicine/ Chia Tai Tianqing Pharmaceutical Group Co, Ltd/ Novartis

Principal Investigator: Professor Baohui Han

Confidentiality statement

The information contained in this document, especially unpublished data, belongs to the sponsor of this study. Since you are treated as a researcher, potential researcher or consultant, it is provided to you as a confidential for you, your research team and independent ethics committee/institutional review committee. It must be clear that, except in the case of obtaining the informed consent of the patient who may take the drug, this information cannot be disclosed to others without the written authorization of the sponsor.

Content

Trial Protocol Synopsis 5
1. Introduction 16
1.1. Background 16
2. Study design 19
2.1. Overview 19
2.2. Part A: Dose Escalation 19
2.3. Part B: dose expansion 22
3. Study objectives 24
3.1. Part A 24
4. Subject selection 25
4.1. Inclusion criteria 25
4.2. Exclusion criteria 26
4.3. Enrollment of subjects 28
4.4. Procedures for handling wrongly enrolled subjects 28
4.5. Restrictions 28
5. Research treatment and execution 30
5.1. Treatment 30
5.1.1. Dose Escalation (Part A) 31
5.1.2. Dose Expansion (Part B) 32
5.1.3. Definition of dose-limiting toxicity 32
5.1.4. Safety Review Committee 33
5.1.5. Effectiveness evaluation 33
5.1.6. Toxicity management 34
5.1.7. Treatment time 37
5.1.8. Treatment compliance and inventory 37
6. Benefit/risk and ethical evaluation 38
6.1. Potential benefits 38
6.2. Potential risks 38
6.2.1. Gastrointestinal tract diseases 38
6.2.2. Skin diseases 39

6.2.3. Cardiovascular system 39
6.2.4. Respiratory system 40
6.2.5. Liver diseases 40
6.2.6. Thyroid 41
6.2.7. Hematopoietic function 41
6.2.8. Hyperlipidemia 41
6.2.9. Reproductive organs 42
6.2.10. CYP450 induction/inhibition 42
7. Discontinue study drugs and withdraw from the study 43
7.1. Procedure for patients to stop study drug 43
7.2. Procedures for patients who started using study drug incorrectly 44
7.3. Procedures for withdrawing from the study 44
8. Research plan and collection of research variables 45
8.1. The detailed steps of the study plan are shown in the table below 45
8.2. Enrollment/screening period 48
8.3. Treatment period 50
8.4. Safety follow-up period 51
8.5. Progressive follow-up period 51
8.6. Survival follow-up period 51
9. Research Evaluation 52
9.1. Effectiveness evaluation 52
9.2. Safety assessment 54
9.2.1. Laboratory safety assessment 54
9.2.2. Physical examination 55
9.2.3. Electrocardiogram 55
9.2.4. Echocardiography/MUGA Scan 56
9.2.5. Vital signs 56
10. Safety report and medical management 57
10.1. Definition of adverse events 57
10.2. Definition of serious adverse events 57
10.3. Adverse Event Record 58
10.3.1. Time period for collecting adverse events 58

10.3.2. Follow-up of unresolved adverse events 58
10.3.3. Variables 58
10.3.4. Causality collection 59
10.3.5. Adverse events based on signs and symptoms 59
10.3.6. Adverse events based on inspections and trials 59
10.3.7. Disease progression 60
10.4. Report of serious adverse events 60
11. Statistical methods 61
11.1. Definition of study endpoint 61
11.2. Calculation of sample size 62
11.3. Statistical analysis 62
12. Ethical considerations 64
References 64
Appendix material 67
Abbreviations 72

Trial Protocol Synopsis

Basic information of drug clinical trial:

- Clinical approval No.: IS2117 (version 1.0)
- Protocol No.: ATRAS-LC-1.0
- Version No. and Date: 1.0/2021.02.21
- Principal Investigator: Baohui Han
- Unit: Shanghai Chest Hospital, Shanghai Jiao Tong University School of Medicine
- Email: 18930858216@163.com

Trial title:

Combined MEK Inhibitor Trametinib and RTK Inhibitor Anlotinib Therapy in non-G12C KRAS-Mutant Lung Cancer patients

Study objectives:

Part A

Primary objective: To define the recommended phase 2 dose (RP2D) of the combined strategy of trametinib and anlotinib for advanced non-small cell lung cancer (NSCLC) patients with non-G12C KRAS mutation.

Secondary objective: To evaluate the progression-free survival (PFS), objective response rate (ORR), disease control rate (DCR) and safety of the combined strategy of trametinib and anlotinib for advanced NSCLC patients with non-G12C KRAS mutation.

Part B

Primary objective: To evaluate the ORR of the combined strategy of trametinib and anlotinib for advanced NSCLC patients with KRAS mutation (excluding $KRAS^{G12C}$).

Secondary objective: To evaluate the PFS, overall survival (OS), DCR, duration of response (DoR) and safety of the combined strategy of trametinib and anlotinib for advanced NSCLC patients with non-G12C KRAS mutation.

Trial design:

- This is a phase I, open-label, single-center study aimed at exploring the potential therapeutic efficacy of trametinib (oral) and anlotinib (oral) in the treatment of advanced NSCLC with KRAS mutations (excluding $KRAS^{G12C}$). The purpose of this study is to explore the best combined dosage under the premise of ensuring patient safety through close safety monitoring. This study is mainly divided into two parts: Part A, dose escalation; Part B, dose expansion. If the valuable ORR and RP2D are reached in part A, part B will be started; if RP2D is not reached in part A, part B will not be started. The therapeutic efficacy and companion diagnostics of the patients enrolled in part A can be reported as a case report or small cohort report before the completion of the clinical trial. The number of subjects is determined according to the actual situation of dose climbing.

Research phase	Timeline（Part A）	Timeline (Part B, if activated)
Estimated date of enrollment of the first subject	July 2021	March 2022
Estimated date of enrollment of the last subject	June 2022	December 2022
Estimated date of the last visit of the last subject	September 2023	February 2024

Part A: Dose escalation

The purpose of designing part A is to determine the RP2D of trametinib and anlotinib based on the evaluation of the ORR and safety data for further clinical evaluation. If the ORR and RP2D are reached, the results can be reported, and the part B will be performed. Here, we decide whether perform to explore the next dose evaluation based on the toxicity of first 28-day cycle administration. The 28-day evaluation period was chosen because it is expected that the major toxicity that caused the discontinued dose in this type of phase I oncology studies (hematology, gastrointestinal tract, liver enzymes) will occur during this period. The dose exploration cohort adopts a "3 + 3"

design. The therapeutic efficacy and companion diagnostics of the patients enrolled in part A can be reported as a case report or small cohort report before the completion of the clinical trial.

The two drugs involved in this study, anlotinib and trametinib, have been marketed, and the standard dosages and adverse reactions have been relatively clear. However, considering that the interaction mechanism of the two drugs has not yet been fully clarified, in order to fully ensure the safety of patients' medication and to explore effective and tolerable clinical combination doses for further clinical trials, we designed three dose gradients.

Cohort	Anlotinib Dose	Trametinib Dose
1	8mg, (d1-d14 per 21-day cycle) orally every day	2mg, orally every day
2	10mg, (d1-d14 per 21-day cycle) orally every day	2mg, orally every day
3	12mg, (d1-d14 per 21-day cycle) orally every day	2mg, orally every day

Based on the observed toxicity, incremental doses of anlotinib are gradually explored. For the determination of maximum tolerated dose (MTD), only the dose-limiting toxicity (DLT) of the first treatment cycle is evaluated. After reviewing the safety data for each dose level, the sponsor and the investigator will jointly determine the DLT.

For a climbing trial designed according to the "3+3" principle, if 0 of the 3 subjects in a given dose level cohort experience DLT, then a higher dose will be explored. If 1 out of 3 subjects develops DLT, the dose level group will recruit 3 more subjects, for a total of 6 subjects. If only 1 of these 6 subjects develops DLT, it is recommended to explore a higher dose, or stop the trial and choose this dose as the MTD (when the dose group is the highest dose). If more than 1 of the 6 subjects has DLT, it indicates that the exploration can be carried out in the lower dose group, or the entire trial can be stopped (when the dose group is the lowest dose group).

If among the 3 subjects enrolled in a given dose group, more than 1 subject with DLT is observed, it is suggested that the exploration can be carried out in a lower dose group. If only 3 subjects are enrolled in the low-dose group, 3 additional subjects will be recruited. At this time, there will be a total of 6 subjects in this dose group for evaluation.

Repeat the above steps to observe the number of subjects with DLT among the 6 subjects.

In order to confirm the MTD, 6 subjects at this dose level will be evaluated for DLT and intolerable toxicity in cycle 1 of treatment (cannot be controlled by dose interruption and/or reduction). The sponsor and investigators will review the safety and clinical data of all subjects to jointly determine the RP2D of anlotinib combined with trametinib.

If the DLT of 2 or more subjects is level 3, then the enrollment of the study will be stopped.

DLT is defined as the occurrence from the first dose of the study treatment (day 1, cycle 0) to the last day of cycle 1 (28 days after the start of dosing), and is not attributable to the disease under study or any disease-related toxicity. Any of the following events that occurred during the first cycle (the first 3 weeks), if it is possible, likely, or definitely related to the drug (according to the National Cancer Institute (NCI) Cancer Therapy Evaluation Program (CTEP) adverse event (AE) reporting requirements), will be considered as a dose-limiting toxicity:

Grade 4 blood toxicity or grade 3 neutropenia with fever ≥38.5°C;

Non-hematological toxicity of grade 3 or above;

Other toxicities greater than baseline, clinically significant and/or unacceptable, and are judged as DLT by the safety review committee.

Any other toxicity of the stopping criteria defined by the protocol (ie, interstitial lung disease or increased QTc with symptoms or signs of severe arrhythmia)

Any other toxicity that caused the interruption of the dosing schedule for more than 7 days.

DLT does not include:

Hair loss of any level

Any level of independent laboratory changes without clinical sequelae or clinical significance.

Therapeutic efficacy evaluation will be assessed according to Response Evaluation

Criteria in Solid Tumors (RECIST) version 1.1 every 4-8 weeks. If the subject has benefited from the efficacy assessment, he/she will continue to receive the next treatment cycle until the progression disease (PD) or unacceptable toxicity occurs to further evaluate the safety, tolerability and efficacy.

Part B: Dose expansion

If RP2D is reached in Part A, another 20 eligible patients will be enrolled and treated with trametinib (2mg QD, oral) + anlotinib (RP2D, once a day, from day 1 to day 14 of the 21-day cycle), until the PD or unacceptable toxicity occurs to further evaluate the safety, tolerability and efficacy. Therapeutic efficacy evaluation will be assessed according to RECIST version 1.1 every 4-8 weeks.

For all eligible patients (Part A and Part B), blood samples will be collected at each evaluation. And if feasible, tissues at PD defined by RECIST will be collected to understand drug resistance. Part A and Part B can be reported respectively.

Figure 1. Trial flow diagram

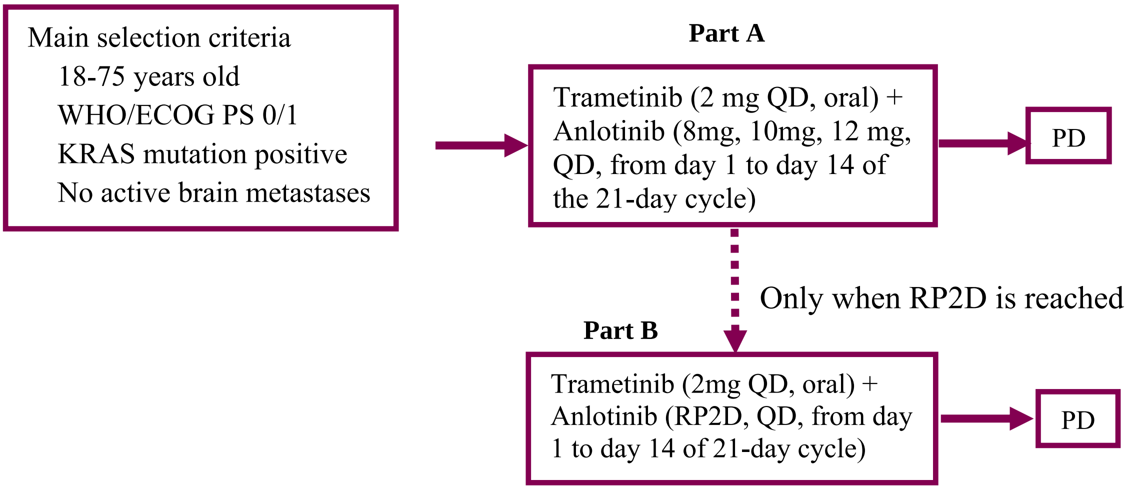

In Part A, in each dose cohort, the first 3 patients must undergo the first cycle observation period. The enrollment of the next dose group must be separated from the previous dose by one medication cycle.

If RP2D is not reached, part B will not be activated.

Inclusion criteria

According to the 8th edition of the American Journal of Critical Care (AJCC)/Union for International Cancer Control (UICC) Tumor Node Metastasis (TNM) NSCLC staging system, locally advanced (stage III B/III C), metastatic or recurrent (stage IV) NSCLC patients confirmed by histology or cytology. The NSCLC patient cannot receive surgery and radical radiotherapy, and at least one measurable lesion is confirmed according to RECIST 1.1.

KRAS mutation positive excluded KRAS^{G12C} mutation

No active brain metastases

Age ≥18 years old and ≤75 years old

ECOG PS score: 0-1

Patients who had previously received at least 1st-line standard therapy. Note: the treatment naïve patients who refused immunotherapy/chemotherapy at 1st-line, and willingly enrolled in the clinical trial are also eligible.

Palliative radiotherapy must be completed 7 days before the first dose of study drug is administered;

The main organs are functioning normally, that is, they meet the following standards:

Good hematopoietic function, defined as absolute neutrophil count ≥1.5×10^9/L, platelet count ≥100×10^9/L, hemoglobin ≥90 g/L [no blood transfusion or no erythropoietin (EPO) within 7 days before enrollment dependence]

The biochemical test results should meet the following standards: BIL <1.25 times the upper limit of normal (ULN); ALT and AST <2.5×ULN; if liver metastasis occurs, ALT and AST <5×ULN; Cr ≤1.5×ULN or creatinine clearance Rate (CCr) ≥60 ml/min; good coagulation function, International Normalized Ratio (INR) and PT ≤1.5 times of ULN; if the subject is receiving anticoagulation therapy, PT should be within the prescribed range of anticoagulation drugs;

Women of childbearing age should agree to take contraceptive measures (such as

intrauterine devices, contraceptives, or condoms) during the study and within 6 months after the study; the serum or urine pregnancy test of non-breastfeeding patients should be negative; male patients should agree to take contraceptive measures during the study period and within 6 months after the study.

The patients voluntarily participated in the study, signed an informed consent form and had good compliance.

The expected survive time is longer than 3 months.

Exclusion criteria

Patients who meet any of the following criteria will be excluded:

Patients with active central nervous system metastasis are excluded. If a subject has received adequate treatment for central nervous system (CNS) metastasis at least 2 weeks before enrollment, and is neurologically restored to baseline levels (except for residual signs or symptoms related to CNS treatment), the subject is eligible. In addition, subjects must stop corticosteroids, or the daily dose of prednisone must be stabilized or gradually reduced to ≤10 mg (or equivalent dose).

Small cell lung cancer (including mixed small cell and non-small cell lung cancer) or hollow central squamous cell carcinoma;

There are obvious bleeding symptoms

Patients who have previously received MEK inhibitors, anlotinib or other multi-targeted anti-angiogenic therapy;

Patients with dysphagia, gastrointestinal resection, chronic diarrhea, intestinal obstruction and other factors that affect oral medication;

Patients who are known to have active brain metastases, spinal cord compression, cancerous meningitis, brain or soft tissue diseases diagnosed by computed tomography (CT) or magnetic resonance imaging (MRI) at the time of screening;

Patients with severe and/or uncontrollable diseases, such as: unstable angina, symptomatic congestive heart failure, myocardial infarction within 6 months, severe uncontrollable arrhythmia; uncontrolled blood pressure (BP, constriction BP > 140

mmHg, diastolic BP > 90 mmHg);

Active or uncontrolled serious infections;

Liver diseases such as cirrhosis, decompensated liver disease, acute or chronic active hepatitis;

Incomplete control of eye inflammation or eye infection, or any condition that may cause the above eye diseases;

Poor diabetes control (fasting blood glucose (FBG) > 10 mmol/L);

Routine urine test results show that urine protein is ≥++, and the 24-hour urine protein quantitative is> 1.0 g;

Active tuberculosis;

Uncontrollable hypercalcemia (calcium ion> 1.5 mmol/L or calcium> 12 mg/dL or corrected serum calcium >ULN), or symptomatic hypercalcemia that requires continued bisphosphonate therapy;

Those who have a history of psychotropic drug abuse and cannot be quit or have mental disorders; Long-term unhealed wounds or fractures;

Patients with known severe allergies (≥Grade 3) to active ingredients and excipients;

Patients who also suffer from other malignant tumors (except radical cervical carcinoma in situ, non-melanoma skin cancer, etc.); patients who have been assessed by the investigator as having concomitant diseases that seriously endanger the patient's safety or affect the patient's completion of the study;

During the clinical trial, the subjects or their sexual partners cannot or refuse to take effective contraceptive measures;

Pregnant or breastfeeding women; Previous treatment including Chinese medicine treatment; Patients who are allergic to any medicine or any ingredient; the patients with a history of treatments involving MEK inhibitors (trametinib, selumetinib, etc.) and RTKs inhibitors (anlotinib, sorafenib, apatinib, cabozantinib, etc.) were considered ineligible.

In other cases, patients who are not eligible for inclusion as assessed by the investigator.

The expected survive time is shorter than 3 months.

Study drugs, dosages, and methods of administration

Cohort	Anlotinib Dose	Trametinib Dose
1	8mg, (d1-d14 per 21-day cycle) orally every day	2mg, orally every day
2	10mg, (d1-d14 per 21-day cycle) orally every day	2mg, orally every day
3	12mg, (d1-d14 per 21-day cycle) orally every day	2mg, orally every day

Statistical methods

The primary endpoint of this study was to determine RP2D based on the incidence of DLT in cycle 1. The specific required sample size will be based on the dose-escalation design of the "3+3" principle, and the DLT situation of each dose group observed during the actual dose escalation process. For the dose expansion phase, it is planned to enroll 20 patients to further clarify the toxicity, tolerability, and efficacy.

All data will be provided for the full analysis set (FAS). All patients who received at least one dose of study drug will be included in the study analysis. For all variables, descriptive statistics will be performed as appropriate. Continuous variables will be summarized by observations, mean, standard deviation, median, minimum, and maximum. Categorical variables will be aggregated by frequency counts and percentages for each category.

The FAS includes all patients who received at least one dose of study drug. The FAS will be used for safety analysis.

The per protocol set (PPS) is a subset of FAS, including all cases that meet the research protocol, has good compliance, does not use any prohibited drugs during the research process. The set of compliance protocols will be used for supporting analysis of effectiveness endpoints.

Evaluation indicators

- **Safety and tolerability**

The safety analysis set (SAS) will include all patients who have received at least one dose of the therapeutic drug. The patient will be evaluated based on the actual treatment

received. The safety and tolerability summary will be based on the safety analysis set. AEs will be coded according to the system organ category (SOC) and preferred terminology in MedDRA. The severity of AE will be graded according to NCI Common Terminology Criteria for Adverse Event (CTCAE) version 5.0. All AEs will be listed, including detailed information collected for each AE (description of event, date/time of onset, duration, severity, severity, relationship with study drug, measures taken, clinical outcome).

- **Tumor response**

Tumor response data will be listed and summarized by dose group, and the following response categories will be used when appropriate: complete response (CR), partial response (PR), stable disease (SD), PD, and non-evaluable (NE). For the definitions of CR, PR, SD and PD, please refer to the RECISIT version 1.1 standard. Where appropriate, the objective tumor response rate will be given with a 95% confidence interval (calculated using the Clopper-Pearson interval).

- **Duration of response**

For the escalation cohort and the expansion cohort, summarize the duration of response of patients, and count the number of patients (%) with remission duration >3; >6; >9; >12 months, draw the Kaplan-Meier curve of median response duration.

- **Progression-free survival and overall survival**

PFS will be summarized in the expansion phase.

PFS will be displayed using Kaplan-Meier curves. The number of events, the median (calculated based on the Kaplan-Meier curve), and the proportion of patients with no events at 6, 12, and 18 months will be summarized.

Where appropriate, a summary of the number and percentage of patients who died, are still under follow-up, lost to follow-up, and withdrawn from the study will be provided.

Ethics

The study protocol (including informed consent form) shall be submitted to the Ethics

Committee for approval, and all subjects entering trial screening must sign an informed consent form. The investigator should carry out the trial in strict accordance with the study protocol and Good Clinical Practice (GCP) requirements, and fully guarantee the legitimate rights and interests and safety of subjects.

1. Introduction

1.1. Background

Lung cancer is the most common cause of cancer-related death worldwide. Approximately 85% to 90% of lung cancer cases are NSCLC, of which KRAS is one of the most common driver genes, occurring in 25-30% of lung adenocarcinomas and 3-5% of squamous cell carcinomas(*1, 2*). The most common KRAS mutations mainly occur at codons 12, 13, and 61, but currently only G12C site has an accessible specific allosteric inhibitor, and G12C mutation accounts for 2.8~13% of NSCLC patients (*3*). Other common mutation sites such as G12V, G12D, Q61H, etc., there is no effective targeted strategy (*4*). The second-line classic docetaxel chemotherapy regimen for the treatment of patients with KRAS mutant NSCLC has a median PFS of less than 3 months and an ORR of only about 10% (*5*).

MEK1/2 is the most critical and the most intensively studied KRAS downstream molecule, and phase II and phase III clinical trials have been carried out. However, the efficacy of MEK inhibitors alone and the maintenance time are limited. With the popularity of combined anti-tumor strategies, a variety of MEK inhibitors combined with other treatment methods have been developed (for example, PI3K/AKT/mTOR inhibitors, FGFR1 inhibitors, IGF1R inhibitors, chemotherapy, etc.)(*6, 7*), which indicates another possible option for future clinical trials, but the existing combination strategy is limited by toxicity or poor efficacy (*8*) (*9*)。

Therefore, we aim to develop a new combination therapy containing MEK inhibitors to treat KRAS-mutant (excluded KRAS^{G12C}) NSCLC patients, and to cover different mutation subtypes as much as possible to form a targeted treatment regimen that is effective for KRAS mutations. Although MEK inhibitors are initially effective against KRAS mutant lung cancer cell lines and patients, relapses will inevitably occur in the short term, mainly due to the induction of multiple RTK genes or ligands. A large number of previous literatures have described the mechanism of MEK inhibitor

resistance in different types of KRAS mutant tumors, involving the upregulation of FGFR1 (*6*), and ERBB in lung cancer; the upregulation of EGFR in colon cancer; and the upregulation of AXL, DDR1, FGFR2, IGF1R, KIT, PDGFRB, VEGFRB, etc. in triple-negative breast cancer (*10*). The diversity of adaptive resistance mechanisms suggests that combined therapy of MEK inhibitors and a single RTK inhibitor is impractical. However, effectively blocking signals from multiple RTK activations may be a viable strategy to prevent drug resistance.

At present, a variety of anti-angiogenic multi-target small molecule tyrosine kinase inhibitors (TKIs) have been approved for the treatment of tumors. We select drugs that cover the above targets (anlotinib) and MEK inhibitors (trametinib) and plan to explore whether the combination of the two drugs can further inhibit the growth of NSCLC. Anlotinib suppresses tumor growth by inhibiting signal pathways involved in angiogenesis (VEGFR1-3, PDGFα-β, FGFR1-4) and cell proliferation (c-KIT, Ret, Aurora-B, c-FMS, DDR1) (*11*). Currently, anlotinib is approved in China for the third-line and later treatment of advanced NSCLC patients, small cell lung cancer, medullary thyroid carcinoma and soft tissue sarcoma (*12-14*). We analyzed the results of the KRAS mutant subgroup in the Phase III clinical trial (ALTER0303) of anlotinib for the treatment of advanced NSCLC in and after the third line. A total of 29 patients carried KRAS mutations, of which 8 cases achieved curative effect of PR, 13 cases were SD, the ORR rate reached 27.6%. The survival time analysis showed that the median PFS of anlotinib single-agent in the posterior line treatment was 4.23 months, and the median OS time was 8.00 months.

Based on the above evidence, we infer that the combined use of anlotinib and trametinib may form an effective strategy for the treatment of KRAS-mutant (excluded $KRAS^{G12C}$) NSCLC patients. We explored *in vitro* cell lines and mouse xenograft models whether the combination of the two drugs can bring better efficacy and whether the toxicity can be tolerated. The results of preclinical data showed that the two drugs showed strong synergistic anti-tumor effects in a variety of NSCLC cell lines carrying different KRAS mutations (G12C, G12D, G12V, G12S, Q61H), and this result was also

validated in xenograft tumor models. There were no significant differences in the weight, mental state and behavior patterns of mice in the control group, single-drug group, and combination-drug group during the entire observation period, suggesting that the combined application of the two drugs did not cause intolerable toxicity. We intend to further explore whether anlotinib combined with trametinib can bring survival benefits to KRAS-mutant (excluded KRAS^{G12C}) NSCLC patients and its possibility as a treatment strategy.

2. Study design

2.1. Overview

This is a phase I, open-label, single-center study aimed at exploring the potential therapeutic efficacy of trametinib (oral) and anlotinib (oral) in the treatment of advanced NSCLC patients with KRAS mutations (excluded KRAS^{G12C}). The purpose of this study is to explore the best combined dosage under the premise of ensuring patient safety through close safety monitoring. This study is mainly divided into two parts: Part A, dose escalation; Part B, dose expansion. If RP2D is reached in part A, part B will be started; if RP2D is not reached in part A, part B will not be started. The number of subjects is determined according to the actual situation of dose climbing.

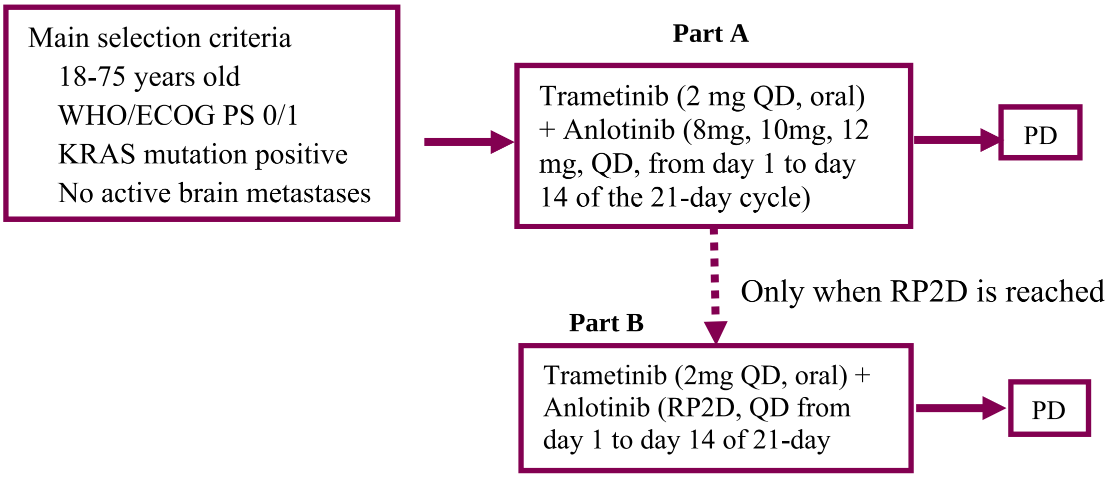

Figure 1. Research flow diagram

In Part A, in each dose cohort, the first 3 patients must undergo the first cycle observation period. The enrollment of the next dose group must be separated from the previous dose by one medication cycle.

If RP2D is not reached, part B will not be activated.

2.2. Part A: Dose Escalation

The purpose of designing part A is to determine the RP2D of trametinib and anlotinib

based on the evaluation of the ORR and safety data for further clinical evaluation. If the ORR and RP2D are reached, the results can be reported, and the part B will be performed. Here, we decide whether perform to explore the next dose evaluation based on the toxicity of first 28-day cycle administration. The 28-day evaluation period was chosen because it is expected that the major toxicity that caused the discontinued dose in this type of phase I oncology studies (hematology, gastrointestinal tract, liver enzymes) will occur during this period. The dose exploration cohort adopts a "3 + 3" design. The therapeutic efficacy and companion diagnostics of the patients enrolled in part A can be reported as a case report or small cohort report before the completion of the clinical trial.

The two drugs involved in this study, anlotinib and trametinib, have been marketed, and the standard dosages and adverse reactions have been relatively clear. However, considering that the interaction mechanism of the two drugs has not yet been fully clarified, in order to fully ensure the safety of patients' medication and to explore effective and tolerable clinical combination doses for further clinical trials, we designed four dose gradients.

Cohort	Anlotinib Dose	Trametinib Dose
1	8mg, (d1-d14 per 21-day cycle) orally every day	2mg, orally every day
2	10mg, (d1-d14 per 21-day cycle) orally every day	2mg, orally every day
3	12mg, (d1-d14 per 21-day cycle) orally every day	2mg, orally every day

Based on the observed toxicity, incremental doses of anlotinib are gradually explored. For the determination of MTD, only the DLT of the first treatment cycle is evaluated. After reviewing the safety data for each dose level, the sponsor and the investigator will jointly determine the DLT.

For a climbing trial designed according to the "3+3" principle, if 0 of the 3 subjects in a given dose level cohort experience DLT, then a higher dose will be explored. If 1 out of 3 subjects develops DLT, the dose level group will recruit 3 more subjects, for a total of 6 subjects. If only 1 of these 6 subjects develops DLT, it is recommended to explore a higher dose, or stop the trial and choose this dose as the MTD (when the dose group

is the highest dose). If more than 1 of the 6 subjects has DLT, it indicates that the exploration can be carried out in the lower dose group, or the entire trial can be stopped (when the dose group is the lowest dose group).

If among the 3 subjects enrolled in a given dose group, more than 1 subject with DLT is observed, it is suggested that the exploration can be carried out in a lower dose group. If only 3 subjects are enrolled in the low-dose group, 3 additional subjects will be recruited. At this time, there will be a total of 6 subjects in this dose group for evaluation. Repeat the above steps to observe the number of subjects with DLT among the 6 subjects.

In order to confirm the MTD, 6 evaluable subjects at this dose level will be evaluated for DLT and intolerable toxicity in cycle 1 of treatment (cannot be controlled by dose interruption and/or reduction). The sponsor and investigators will review the safety and clinical data of all subjects to jointly determine the RP2D of anlotinib combined with trametinib.

If the DLT of 2 or more subjects is level 3, then the enrollment of the study will be stopped.

DLT is defined as the occurrence from the first dose of the study treatment (day 1, cycle 0) to the last day of cycle 1 (28 days after the start of dosing), and is not attributable to the disease under study or any disease-related toxicity. Any of the following events that occurred during the first cycle (the first 3 weeks), if it is possible, likely, or definitely related to the drug (according to the NCI CTEP AE reporting requirements), will be considered as a dose-limiting toxicity:

Grade 4 blood toxicity or grade 3 neutropenia with fever ≥38.5°C;

Non-hematological toxicity of grade 3 or above;

Other toxicities greater than baseline, clinically significant and/or unacceptable, and are judged as DLT by the safety review committee.

Any other toxicity of the stopping criteria defined by the protocol (i.e., interstitial lung disease or increased QTc with symptoms or signs of severe arrhythmia)

Any other toxicity that caused the interruption of the dosing schedule for more than

7 days.

DLT does not include:

Hair loss of any level

Any level of independent laboratory changes without clinical sequelae or clinical significance.

Multiple blood samples will be collected on the first day of the first cycle and the second cycle before dosing and 0.5, 1, 2, 4, 6, 8 and 24 hours after dosing. The investigator will collect 6ml of venous blood at each time point, separate the plasma and store it at -80°C until analysis. The validated high-performance liquid chromatography-tandem mass spectrometry (HPLC-MS/MS) method was used for analysis to understand the plasma concentration-time curves of the two drugs on the first day and steady state of each dose.

Tumor response will be assessed according to RECIST version 1.1 every 4-8 weeks. If the subject has benefited from the efficacy assessment, he/she will continue to receive the next treatment cycle until the PD or unacceptable toxicity occurs to further evaluate the safety, tolerability and efficacy.

2.3. Part B: dose expansion

If RP2D is reached in Part A, another 20 eligible patients will be enrolled and treated with trametinib (2mg QD, oral) + anlotinib (RP2D, once a day, from day 1 to day 14 of the 21-day cycle), until the PD or unacceptable toxicity occurs to further evaluate the safety, tolerability and efficacy. Therapeutic efficacy evaluation will be assessed according to RECIST version 1.1 every 4-8 weeks.

For all eligible patients (Part A and Part B), blood samples will be collected at each evaluation. And if feasible, tissues at PD defined by RECIST will be collected to understand drug resistance. Part A and Part B can be reported respectively.

Accurate identification of genetic changes after progression is not only critical for patient management, but also for advancing the understanding of treatment-induced tumor evolution. Although repeated biopsy is advertised as a tool for monitoring tumor

evolution, this method may be too cumbersome in the real world to be applied to every patient. Obtaining a tissue biopsy for molecular analysis can be challenging. In up to 20% of patients, there is a significant risk of bleeding, infection, and other complications. For up to 49% of patients, doctors cannot perform a biopsy for molecular analysis due to comorbidities or insufficient tumor tissue. Therefore, there is an urgent need for a non-invasive liquid biopsy method to assess tumor mutations and achieve simple repeated detection throughout the treatment process. The isolation and subsequent molecular analysis of non-cellular circulating tumor DNA (ctDNA) in plasma is a powerful tool that can help improve the clinical outcome of many cancer types, including NSCLC. At the same time, by collecting a variety of secreted proteins in plasma, proteomics chips can be used to further explore the biomarkers in response to dual-drug combination therapy and the internal mechanism of dual-drug synergy.

In this study, based on plasma proteomics and ctDNA changes, the biomarkers related to the efficacy of dual-drug combination therapy and the internal biological mechanism of dual-drug synergistic anti-tumor will be explored.

3. Study objectives

3.1. Part A

3.1.1. Main purpose

To evaluate define the RP2D of the combined strategy of trametinib and anlotinib for advanced NSCLC patients with KRAS mutation (excluded $KRAS^{G12C}$).

3.1.2. Secondary purpose

To evaluate the ORR, PFS, DCR and safety of the combined strategy of trametinib and anlotinib for advanced NSCLC patients with KRAS mutation (excluded $KRAS^{G12C}$).

3.2. Part B

3.2.1. Main purpose

To evaluate the ORR of the combined strategy of trametinib and anlotinib for advanced NSCLC patients with KRAS mutation (excluded $KRAS^{G12C}$).

3.2.2. Secondary purpose

To evaluate the PFS, OS, DCR, DoR and safety of the combined strategy of trametinib and anlotinib for advanced NSCLC patients with KRAS mutation (excluded $KRAS^{G12C}$).

3.3. Exploratory purpose

To explore biomarkers related to the efficacy and resistance of dual-drug combination therapy and track changes in mutation profiles.

4. Subject selection

Each subject should meet all the inclusion criteria of this study and not meet any exclusion criteria. Under no circumstances can this rule be an exception.

4.1. Inclusion criteria

According to the 8th edition of the AJCC/UICC TNM NSCLC staging system, locally advanced (stage III B/III C), metastatic or recurrent (stage IV) NSCLC confirmed by histology or cytology. The patient cannot receive surgery and radical radiotherapy, and at least one measurable lesion is confirmed according to RECIST 1.1.

KRAS mutation positive

No active brain metastases

Age ≥18 years old and ≤75 years old

ECOG PS score: 0-1

Patients who had previously received at least 1st-line standard therapy. Note: the treatment naïve patients who refused immunotherapy/chemotherapy at 1st-line, and willingly enrolled in the clinical trial are also eligible

Palliative radiotherapy must be completed 7 days before the first dose of study drug is administered

The main organs are functioning normally, that is, they meet the following standards:

Good hematopoietic function, defined as absolute neutrophil count $\geq 1.5\times 10^9$/L, platelet count $\geq 100\times 10^9$/L, hemoglobin ≥90 g/L [no blood transfusion or no EPO within 7 days before enrollment dependence]

The biochemical test results should meet the following standards: BIL <1.25 times the ULN; ALT and AST <2.5×ULN; if liver metastasis occurs, ALT and AST<5×ULN; Cr≤1.5×ULN or CCr ≥60 ml/min; good coagulation function, INR and PT≤1.5 times of ULN; if the subject is receiving anticoagulation therapy, PT should be within the prescribed range of anticoagulation drugs

Women of childbearing age should agree to take contraceptive measures (such as

intrauterine devices, contraceptives, or condoms) during the study and within 6 months after the study; the serum or urine pregnancy test of non-breastfeeding patients should be negative; male patients should agree to take contraceptive measures during the study period and within 6 months after the study

The patients voluntarily participated in the study, signed an informed consent and had good compliance

The expected survive time is longer than 3 months

4.2. Exclusion criteria

Patients who meet any of the following criteria will be excluded:

Patients with active central nervous system metastasis are excluded. If a subject has received adequate treatment for CNS metastasis at least 2 weeks before enrollment, and is neurologically restored to baseline levels (except for residual signs or symptoms related to CNS treatment), the subject is eligible. In addition, subjects must stop corticosteroids, or the daily dose of prednisone must be stabilized or gradually reduced to ≤10 mg (or equivalent dose).

Small cell lung cancer (including mixed small cell and non-small cell lung cancer) or hollow central squamous cell carcinoma

There are obvious bleeding symptoms

Patients who have previously received MEK inhibitors, anlotinib or other anti-angiogenic therapy

Patients with dysphagia, gastrointestinal resection, chronic diarrhea, intestinal obstruction and other factors that affect oral medication

Patients who are known to have active brain metastases, spinal cord compression, cancerous meningitis, brain or soft tissue diseases diagnosed by CT or MRI at the time of screening

Patients with severe and/or uncontrollable diseases, such as: unstable angina, symptomatic congestive heart failure, myocardial infarction within 6 months, severe uncontrollable arrhythmia; uncontrolled BP (constriction BP > 140 mmHg, diastolic

BP > 90 mmHg)

Active or uncontrolled serious infections

Liver diseases such as cirrhosis, decompensated liver disease, acute or chronic active hepatitis

Incomplete control of eye inflammation or eye infection, or any condition that may cause the above eye diseases

Poor diabetes control (FBG >10 mmol/L)

Routine urine test results show that urine protein is ≥++, and the 24-hour urine protein quantitative is> 1.0 g

Active tuberculosis

Uncontrollable hypercalcemia (calcium ion> 1.5 mmol/L or calcium> 12 mg/dL or corrected serum calcium> ULN), or symptomatic hypercalcemia that requires continued bisphosphonate therapy

Long-term unhealed wounds or fractures

Those who have a history of psychotropic drug abuse and cannot be quit or have mental disorders

Patients with known severe allergies (≥Grade 3) to active ingredients and excipients;

Patients who also suffer from other malignant tumors (except radical cervical carcinoma in situ, non-melanoma skin cancer, etc.); patients who have been assessed by the investigator as having concomitant diseases that seriously endanger the patient's safety or affect the patient's completion of the study;

During the clinical trial, the subjects or their sexual partners cannot or refuse to take effective contraceptive measures;

Pregnant or breastfeeding women; Previous treatment including Chinese medicine treatment; Patients who are allergic to any medicine or any ingredient; the patients with a history of treatments involving MEK inhibitors (trametinib, selumetinib, etc.) and RTKs inhibitors (anlotinib, sorafenib, apatinib, cabozantinib, etc.) were considered ineligible.

In other cases, patients who are not eligible for inclusion as assessed by the investigator.

The expected survive time is shorter than 3 months.

4.3. Enrollment of subjects

All subjects considered for inclusion in the study must sign an informed consent form within 28 days before the start of treatment and before any study-specific screening procedures. The baseline assessment must be performed within 28 days before the start of study treatment, with the exception of pregnancy tests (if applicable). For serum samples, a pregnancy test needs to be performed within 72 hours before the first day of the first cycle. Before study treatment, it is necessary to confirm that the pregnancy test result is negative. The laboratory assessment and accompanying research blood sampling can be carried out within 72 hours before the 1st day of any cycle, and the tumor/imaging assessment can be carried out within 7 days, so that the investigator can obtain the results of the study during the visit.

4.4. Procedures for handling wrongly enrolled subjects

In any case, subjects who do not meet the eligibility criteria should not be included in or receive study medication. There can be no exceptions to this rule. Subjects who have been enrolled but subsequently found to not meet all eligibility criteria shall not start treatment and must withdraw from the study.

If the subject does not meet all eligibility criteria but starts treatment by mistake, the investigator will immediately notify the sponsor, and the sponsor and investigator should discuss whether to continue or terminate the patient's treatment. The sponsor must ensure that all decisions are properly documented.

4.5. Restrictions

The following restrictions apply to patients during the study drug treatment and specific times before and after treatment:

(1) Women with reproductive potential must use reliable contraceptive methods from the beginning of screening to 6 months after the drug is stopped. When in line with the subject’s preferred and general lifestyle, acceptable contraceptive methods include true

abstinence. [Periodic abstinence (for example, calendar, ovulation, symptoms, post-ovulation method), the announcement and withdrawal of abstinence during the trial period are unacceptable contraceptive methods], hormonal contraceptives that are not prone to drug interactions (for example, intrauterine birth control system [IUS] levonorgestrel intrauterine system, medroxyprogesterone injection), copper belt intrauterine device and partner undergoing vasectomy. All hormonal contraceptive methods should be used with condoms and used by male sexual partners during intercourse.

(2) During the trial period (6 months), male patients must be required to use isolation contraceptives (that is, use condoms) during sex with all female partners. The patient should not give birth within 6 months after completing the medication. Patients must avoid donating sperm from the start of administration until 6 months after the discontinuation of study drug treatment. If male patients wish to give birth, they should be advised to arrange for a frozen sperm sample before starting study medication.

(3) If it is medically feasible, the patient should maintain treatment with conventional drugs other than the potent inducer of CYP3A4 (see Appendix B) throughout the study period (90 days after the last dose). If the patient is taking a drug that relies on blood C reactive protein (BCRP) and/or P-glycoprotein (Pgp) for distribution and has a narrow therapeutic index, it should be closely monitored in order to detect the increased exposure and changes in tolerance of the combination. If the patient is taking a drug that relies on CYP3A4, CYP1A2, CYP2C or p-glycoprotein for distribution and has a narrow therapeutic index, the decrease in study drug activity due to the reduced exposure of the combined drug should be closely monitored. Guidance is provided on medications to avoid, medications that require close monitoring, and washout periods (see Appendix B).

5. Research treatment and execution

5.1. Treatment

It is planned to take the combined doses of trametinib (2mg, QD) and anlotinib (8mg, 10mg, 12mg, QD, the first day to the 14th day of the 21-day cycle) respectively. Novartis provides trametinib, and Chia Tai Tianqing Pharmaceutical will provide anlotinib. All study drugs should be kept in a safe place under proper storage conditions. The study drug label on the package specifies proper storage conditions. The label will be produced in accordance with Good Manufacturing Practices (GMP) and Chinese regulatory guidelines. The label text will be translated into Chinese. The investigator's handbook will provide more detailed information.

All eligible patients can obtain anlotinib combined with trametinib treatment by participating in this clinical study. Treatment will continue until RECIST-defined disease progression, unacceptable toxic effects, withdrawal from the study, or death occurs. For patients with RECIST-defined progression, if the patient continues to show clinical benefit and there is no discontinuation standard, the investigator can decide whether to continue the study treatment.

Figure 1. Research flow diagram

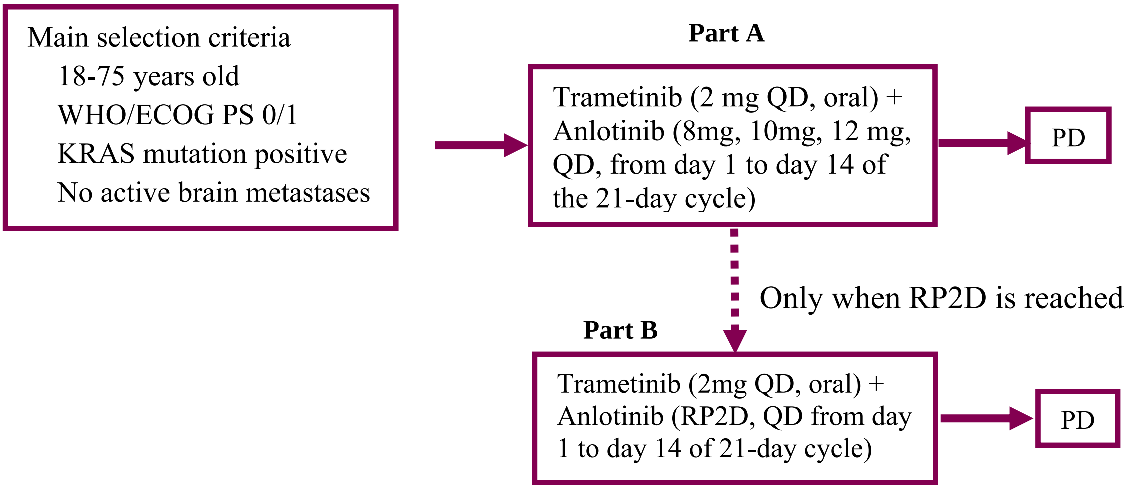

In Part A, in each dose cohort, the first 3 patients must undergo the first cycle observation period. The enrollment of the next dose group must be separated from the

previous dose by one medication cycle.

If RP2D is not reached, part B will not be activated.

5.1.1. Dose Escalation (Part A)

The two drugs involved in this study, anlotinib and trametinib, have been marketed, and their standard dosages and adverse reactions have been relatively clear. However, considering that the interaction mechanism of the two drugs has not yet been fully clarified, in order to fully guarantee the safety of patients' medication and to explore effective and tolerable clinical combination doses for further clinical trials, we designed four doses gradient.

Cohort	Anlotinib Dose	Trametinib Dose
1	8mg, (d1-d14 per 21-day cycle) orally every day	2mg, orally every day
2	10mg, (d1-d14 per 21-day cycle) orally every day	2mg, orally every day
3	12mg, (d1-d14 per 21-day cycle) orally every day	2mg, orally every day

Based on the observed toxicity, incremental doses of anlotinib are gradually explored. For the determination of MTD, only the DLT of the first treatment cycle is evaluated. After reviewing the safety data for each dose level, the sponsor and the investigator will jointly determine the DLT.

For a climbing trial designed according to the "3+3" principle, if 0 of the 3 subjects in a given dose level cohort experience DLT, then a higher dose will be explored. If 1 out of 3 subjects develops DLT, the dose level group will recruit 3 more subjects, for a total of 6 subjects. If only 1 of these 6 subjects develops DLT, it is recommended to explore a higher dose. If more than 1 of the 6 subjects has DLT, it indicates that the exploration can be carried out in the lower dose group, which as MTD. If we can not find more than 1 of the 6 subjects has DLT in Level 3, We will not continue to next dose level.

After part A, the investigators will review the safety and clinical data of all subjects to jointly determine the RP2D of anlotinib combined with trametinib.

5.1.2. Dose Expansion (Part B)

If RP2D is reached in Part A, another 20 eligible patients will be included and receive trametinib (2mg QD, oral) + anlotinib (RP2D, once a day, from day 1 to day 14 of the 21-day cycle), until the disease progression, withdrawal of informed consent, or unacceptable toxicity occurs to further evaluate the safety, tolerability and efficacy.

In the course of the study, once a patient has an AE of CTCAE grade 3 or above, and can recover to grade 1 or below within 2 weeks, the medication can be continued, and the subsequent medication dose will be lowered by one dose based on the original planned medication dose, otherwise the study will be withdrawn. Patients who continue to take the drug after the dose reduction reoccurs CTCAE grade 3 and above AEs during the follow-up process, and the AEs can return to grade 1 or below within 2 weeks, and they can continue to use this drug. The subsequent dose will be reduced by one dose based on the original planned dose, otherwise the study will be withdrawn.

5.1.3. Definition of dose-limiting toxicity

DLT is defined as the occurrence from the first dose of the study treatment (day 1, cycle 0) to the last day of cycle 1 (28 days after the start of dosing), and is not attributable to the disease under study or any disease-related toxicity. Any of the following events that occurred during the first cycle (the first 4 weeks), if it is possible, likely, or definitely related to the drug (according to the NCI CTEP AE reporting requirements), will be considered as a dose-limiting toxicity:

Grade 4 blood toxicity or grade 3 neutropenia with fever ≥38.5°C;

Non-hematological toxicity of grade 3 or above;

Other toxicities greater than baseline, clinically significant and/or unacceptable, and are judged as DLT by the safety review committee.

Any other toxicity of the stopping criteria defined by the protocol (ie, interstitial lung disease or increased QTc with symptoms or signs of severe arrhythmia))

Any other toxicity that caused the interruption of the dosing schedule for more than

7 days.

DLT does not include:

Hair loss of any level

Any level of independent laboratory changes without clinical sequelae or clinical significance.

However, scientific review committee (SRC) will take into account the incidence and type of DLT-type toxicity in cycle 2 and beyond when determining the dose escalation step.

5.1.4. Safety Review Committee

SRC is responsible for evaluating the safety and tolerability of the combination of trametinib and anlotinib, and deciding the next step to ensure patient safety. The SRC will be composed of principal investigators, who will chair the committee and be responsible for routine SRC meetings.

The first SRC meeting will be held after the first patient completes the first cycle of combined therapy. After that, the SRC meeting will be held after the completion of the first cycle of each of the first 3 patients (if necessary) in each dose cohort, and every 2 after 6 months or after the first 3 patients are enrolled. Held once a month.

The decision will include, but is not limited to, stopping further inclusion to ensure patient safety, reducing the dose of combination therapy, and re-evaluating the principles of AE management and dose adjustment to reduce risks.

5.1.5. Effectiveness evaluation

According to the RECIST 1.1 standard, all subjects will be monitored by radiological evaluation every 4-8 weeks to determine changes in tumor size. For OS, every effort will be made to collect survival data for all subjects, including subjects who withdrew from treatment for any reason, are eligible to participate in the study and have not revoked their consent for survival data collection. If the death of the subject is not reported, all dates in this study representing the date of contact of the subject will be

used to determine the subject's last known survival date.

5.1.6. Toxicity management

Dose reductions, dose delays, and dose interruptions are permitted. If a patient has CTCAE grade 3 or higher and/or unacceptable toxicity (any grade), and the investigator believes that the event of concern is particularly related to the study drug (and includes the disease or disease-related process that is not related to the treatment of the patient DLT), the administration will be interrupted, and supportive treatment will be carried out in accordance with the guidelines.

If the toxicity subsides or returns to CTCAE ≤ 2 within 2 weeks after the onset, the study drug treatment can be restarted at the same or lower dose according to the investigator's assessment and according to the dose adjustment rules (Table 3). If the administration is restarted at the same dose level, the patient should be closely monitored 3 days after restarting treatment. If recurrence or worsening of the same toxicity occurs within 3 days, the investigator should consider reducing the dose as appropriate. Once the dose is reduced, the dose of the study drug cannot be restored to the high dose.

If the toxicity has not been relieved to CTCAE ≤ 2 after 2 weeks, the patient must withdraw from the study treatment and continue to observe the patient until the toxicity subsides.

If the dose needs to be interrupted after an AE, the investigator can restart the study drug at the same dose or a reduced dose according to the resolution/improvement of the event. Toxicity subsided within 14 days:

If the same AE recurs and the dose needs to be interrupted later, the study drug must be restarted with a lower dose after the AE improves.

If a different AE occurs and then the dose needs to be interrupted, according to the judgment of the investigator, after the AE is improved, the study drug can be re-administered at the same dose or at a lower dose level.

According to the judgment of the investigator, the lowest possible dose can be allowed,

the dose has been reduced to the lowest dose in the past, and the patient who shows an acceptable response to the interruption of the dose can be restarted with the lowest dose. Based on previous safety data for anlotinib and trametinib, patients were allowed to discontinue one of the drugs and continue with the remaining drug as monotherapy in the event of specific toxicities. And according to the degree of adverse reactions and remission, it is decided whether to delay, adjust or stop the dose of the drug in the follow-up. Adjustment to monotherapy is acceptable due to toxicity considerations. According to the investigator's judgment, for adverse reactions that may be caused by both drugs or some abnormal lipid metabolism, abnormal renal function and bleeding symptoms specific to anlotinib, the dose of anlotinib should be adjusted. If unacceptable toxicity persists after the lowest dose of anlotinib, trametinib monotherapy should be considered. For adverse reactions considered by the investigator to be particularly related to trametinib, the treatment of trametinib should be suspended, and if the use of trametinib cannot be resumed after supportive treatment, anlotinib monotherapy should be used.

a) Lung symptoms

If new or worsening pulmonary symptoms (such as dyspnea) or radiological abnormalities suggesting interstitial lung disease are observed, it is recommended to interrupt the study treatment dose and notify the sponsor research team. The expert group will discuss to rule out other causes such as lymphatic cancer, infection, allergies, cardiogenic edema or pulmonary hemorrhage. Case report form (CRF) will collect the results of the expert panel (including high-resolution computed tomography (HRCT), blood and sputum culture, hematology parameters). If there is a confirmatory HRCT scan, but other causes of respiratory symptoms are excluded, the diagnosis of interstitial lung disease should be considered and study treatment should be permanently terminated.

b) QTc interval prolongation

In view of the possibility of QT changes related to anlotinib, electrolyte abnormalities (hypokalemia, hypomagnesemia, hypocalcemia) must be corrected to the normal range

before the first dose, and tested during the study treatment Electrolyte level. Patients who meet the DLT criteria and have prolonged QTcF (ie, the confirmed QTcF prolongs to an absolute value of >500 milliseconds or an increase of >60 milliseconds from the baseline) should discontinue the study treatment and undergo regular electrocardiogram (ECG) examinations until relief to <481 milliseconds or such as baseline QTcF >481 milliseconds, it returns to the baseline. If the toxicity subsides or returns to ≤1 (<481 milliseconds) within 14 days after the onset, the investigator can restart the study drug at a reduced dose as appropriate. If the QTcF prolonged toxicity does not fall to ≤ Grade 1 within 14 days, the patient will be permanently withdrawn from the study treatment.

c) Keratitis

Signs and symptoms of keratitis, such as acute or worsening: eye inflammation, tearing, sensitivity to light, blurred vision, eye pain and/or red eyes should be referred to an ophthalmologist immediately.

d) Permanent discontinuation due to toxicity

Patients with interstitial lung disease (ILD) or prolonged QTc interval with signs/symptoms of severe arrhythmia will not be allowed to restart study treatment.

e) Bleeding

Bleeding includes hemoptysis, gastrointestinal bleeding, nose bleeding, bronchial bleeding, gum bleeding, gross hematuria, fecal occult blood and cerebral hemorrhage. Patients with grade 2 bleeding should discontinue study treatment and give supportive treatment according to local practice/guidelines. If the toxicity subsides or returns to grade ≤1 within 14 days after the onset, the investigator may restart the study drug at a reduced dose as appropriate. If a patient has level 3 or higher bleeding, and the investigator believes that the event of concern is particularly related to the study drug (and has nothing to do with the disease or disease-related process that the patient is being treated for), the patient should permanently stop the study drug. In addition, symptomatic and supportive treatment should be taken immediately in accordance with local practice/guidelines.

5.1.7. Treatment time

All eligible patients can obtain anlotinib combined with trametinib treatment plan by participating in this clinical trial. Treatment of the study drug will continue until disease progression, unacceptable toxicity, withdrawal from the study, or death as defined by RECIST. For patients with RECIST-defined progression, if the patient continues to show clinical benefit and there is no discontinuation standard, the investigator can decide whether to continue the study treatment at the discretion of the investigator. Progression follow-up defined after RECIST is optional and will be determined by the investigator. AEs were evaluated from the time of obtaining informed consent until at least 30/90 days after the last administration of trametinib or anlotinib.

If the study drug is discontinued for reasons other than disease progression, the patient must continue the RECIST version 1.1 assessment every 6 weeks until the disease progresses or the next-line treatment begins (whichever occurs first).

5.1.8. Treatment compliance and inventory

Study drugs should only be used as directed in this protocol. The detailed information of each patient treated with the study drug will be recorded in the case record form.

The patient should return all unused drugs and empty containers to the investigator.

Researchers at the research center will count all the medicines distributed and destroy them appropriately. The certificate of delivery and destruction will be signed.

6. Benefit/risk and ethical evaluation

6.1. Potential benefits

This is a phase I, open-label, single-center study of trametinib combined with anlotinib in KRAS (excluded $KRAS^{G12C}$) mutation-positive advanced NSCLC patients. This study aims to explore the best combined dosage while ensuring patient safety through close safety monitoring.

Previous studies have confirmed that MEK inhibitors have a certain clinical effect on KRAS-mutated NSCLC patients, but the effective time is short. The recurrence process is often accompanied by the activation of upstream RTK molecules, such as FGFR, VEGFR, c-KIT, etc. This suggests that the combined application of MEK inhibitors and multi-target anti-angiogenesis inhibitors should produce stronger and longer-lasting anti-tumor effects. We further demonstrated this synergistic effect through in vitro cell lines and in vivo mouse xenograft models. It is a potential targeted therapy for NSCLC with different subtypes of KRAS mutations. The recent emergence of G12C inhibitors has provided the accessible allosteric inhibitors for the $KRAS^{G12C}$-mutant NSCLC patients, but G12C accounts for 2.8% ~ 13% of NSCLC. There is still a large proportion of KRAS-mutant NSCLC patients (8.4% ~ 20% of NSCLC patients) who urgently need effective treatment plan. Therefore, we intend to explore the safety and survival benefits of trametinib combined with anlotinib in the treatment of KRAS-mutant (excluded $KRAS^{G12C}$) NSCLC patients.

6.2. Potential risks

The monitoring and management of potential risks are discussed as follows:

6.2.1. Gastrointestinal tract diseases

Patients with intractable nausea, vomiting, and chronic gastrointestinal diseases were

not included in this study. Investigators should also follow general toxicity management guidelines regarding interruptions and dose reductions.

Patients with active bleeding symptoms were excluded. If gastrointestinal bleeding occurs, including stool occult blood (++) or more, hematemesis or vomiting blood, symptomatic treatment should be given. Fasting, acid suppression, blood transfusion, and supportive treatment are given to patients with upper gastrointestinal bleeding. If necessary, octreotide can be used. If lower gastrointestinal bleeding occurs, hemostasis, blood transfusion, and supportive treatment should be given. If the bleeding cannot be controlled, surgery must be performed.

6.2.2. Skin diseases

This study did not include any specific dermatological exclusion criteria, but patients with unresolved AEs greater than CTCAE Grade 1 in previous treatments will be excluded from the study. For skin disease patients with any CTCAE-level skin reaction that the investigator believes is causally related to the study drug, dermatological treatment should be taken. Investigators should also follow general toxicity management guidelines regarding dose interruption and reduction. Photography can be taken to record any clinically important findings.

6.2.3. Cardiovascular system

Patients with risk factors for unstable cardiac conditions and prolonged QT interval will be excluded from this study. Where feasible, the simultaneous use of conventional drugs that may prolong the QT interval will be restricted, but patients can receive any drugs that are clinically indicated for the treatment of AEs. Throughout the study, electrolytes and vital signs assessments, including pulse rate and BP will be monitored regularly. Within 24 hours after the first study drug is taken, and at the assumed steady state (day 8 of continuous dosing), a series of three repeated digital ECG assessments will be performed, and a single record will be taken at the beginning of each subsequent treatment cycle ECG. The researcher or designated doctor will check each ECG before

leaving the hospital, and if appropriate, consult a local cardiologist to treat the patient immediately. ECG data suggest that a cardiologist should analyze heart rate, PR, R-R, and QT intervals. If the ECG abnormality at screening or baseline is deemed by the investigator to be clinically significant, it should be reported as a concurrent condition. For patients with cardiac risk factors that may affect left ventricular ejection fraction (LVEF), cardiac monitoring should be considered, including assessment of LVEF at baseline and during treatment. For patients with relevant cardiac signs/symptoms during treatment, cardiac monitoring, including LVEF assessment, should be considered.

Patients with uncontrolled hypertension were excluded. In the case of elevated BP, you need to actively communicate with your doctor. In the case of elevated BP, conventional antihypertensive therapy can be controlled. If BP is difficult to control, the target drug dose can be lowered or cancelled.

6.2.4. Respiratory system

Patients with a history of interstitial lung disease, drug-induced interstitial lung disease, radiation pneumonitis requiring steroid therapy, or any evidence of clinically active interstitial lung disease will be excluded from this study. If new or worsening pulmonary symptoms (such as dyspnea) or radiological abnormalities suggesting interstitial lung disease are observed, it is recommended to discontinue the dose of the study treatment and notify the sponsor research team. The expert group will discuss to rule out other causes such as lymphatic cancer, infection, allergies, cardiogenic edema or pulmonary hemorrhage. The results of the discussion will be collected. If there are confirmed HRCT imaging findings, but other causes of respiratory symptoms are excluded, the diagnosis of interstitial lung disease should be considered, and study treatment should be permanently terminated.

6.2.5. Liver diseases

Patients with any signs of severe or uncontrollable systemic liver disease, including

known hepatitis B, hepatitis C, human immunodeficiency virus (HIV) or liver enzyme abnormalities (defined as AST or ALT >2.5 × ULN, if there is no evidence of liver metastasis, total bilirubin >1.5 × ULN; AST or ALT with liver metastasis >5 × ULN, total bilirubin > 3 × ULN) does not participate in the study. During the study period, liver function tests will be regularly monitored during the study period and recorded.

6.2.6. Thyroid

During the entire treatment period, the thyroid function of all patients was closely monitored. When Thyroid Stimulating Hormone (TSH) ≥20mU/L or any value of T3, T4, FT3, FT4 is lower than the normal value, the corresponding treatment method should be used.

6.2.7. Hematopoietic function

If any of the following laboratory results (absolute neutrophil count <1.5 × 10^9/L; platelet count <100 × 10^9/L; hemoglobin <90 g/L) shows that patients with insufficient bone marrow reserve will be excluded from the study outside. Before the first dosing, hematological parameters will be monitored, once a week during the first cycle of multiple dosing, once at the beginning of each subsequent cycle, and once at the stop of the drug.

6.2.8. Hyperlipidemia

The treatment of hyperlipidemia should consider the patient's pre-treatment status and eating habits. In addition to diet control, hypercholesterolemia of grade 2 or higher (≥7.75 mmol/L) or hypertriglyceridemia of grade 2 or higher (≥2.5 × ULN) should also be given appropriate HMG-CoA reductase inhibitors (atorvastatin) or lipid-lowering drugs.

6.2.9. Reproductive organs

According to data from animal studies and the drug mechanisms, the use of anlotinib and trametinib in pregnant women may cause harm. There are currently no available data on the use of anlotinib and trametinib in pregnant women. Therefore, all men and women with reproductive potential will be required to take appropriate contraceptive measures during the study period and for an appropriate period thereafter. Women of reproductive potential must have a negative pregnancy test before receiving the first dose of study treatment. There are currently no data on the presence of study drugs or active metabolites in breast milk, and no data on the effect on breastfed infants or milk production. Women who breastfeed will be excluded from the study. According to studies on animals, treatment with anlotinib and trametinib may damage the fertility of men and women.

6.2.10. CYP450 induction/inhibition

All patients must avoid the simultaneous use of other drugs, herbal supplements and/or food intake that are known to have strong inducing effects on CYP3A4 activity. Such drugs must be discontinued within an appropriate time before entering screening and within 3 months after the last dose of study drug. If the patient is taking a drug that relies on BCRP and PgP. for distribution and has a narrow therapeutic index, it should be closely monitored to detect changes in tolerance due to increased exposure to the combined drug. See Appendix B for guidance on drugs to avoid, drugs that require close monitoring, and washout periods. Where medically feasible, except for strong inducers of CYP3A4 and inhibitors of CYP2C8, patients should maintain regular medication throughout the study. Patients can receive any medications that are clinically used to treat AEs.

7. Discontinue study drugs and withdraw from the study

Patients can stop using the study treatment in the following cases:

The patient's decision. The patient is free to withdraw from the study he/she is participating in at any time, without being affected by any prejudice.

Adverse events

Pregnancy

The researcher believes that serious non-compliance with this research protocol

The investigator assesses that the study drug has no clinical benefit

The patient starts to use the study drug incorrectly

Patients who withdrew from the study but can be evaluated will not be replaced. Any patients whose consent is withdrawn and cannot be evaluated will be replaced to ensure the minimum number of patients that can be evaluated.

7.1. Procedure for patients to stop study drug

Once the study drug is permanently disabled, it cannot be reactivated.

Patients who discontinue study treatment do not always automatically withdraw from the study. If the patient withdraws from the study, see section 7.3.

Any patient who discontinues study treatment for reasons other than objective disease progression should undergo tumor assessment scans in accordance with the plan in the protocol. Serious adverse events (SAEs) and concomitant medications related to the study procedure must be collected until the patient is no longer evaluated by RECIST version 1.1 (disease progression or permanent withdrawal from the study).

7.2. Procedures for patients who started using study drug incorrectly

In any case, subjects who do not meet the eligibility criteria should not be included in or receive study medication. There can be no exceptions to this rule. If a patient who does not meet the enrollment criteria is enrolled by mistake or starts treatment by mistake, or the patient does not meet the research criteria after the start, the investigator should decide when to terminate the study of the unqualified patient based on the patient's medical/safety risk.

7.3. Procedures for withdrawing from the study

Patients can freely withdraw from the study (study drugs and evaluation) at any time without affecting further treatment (withdrawal of consent). Such patients are always asked about the reason and whether there are any AEs. If possible, the investigator will examine them, perform the assessments and procedures required for post-study evaluation, and then withdraw from the study. AEs should be followed up; study medication should be returned by the patient.

8. Research plan and collection of research variables

8.1. The detailed steps of the study plan are shown in the table below

	Screening/Enrollment Visit		Treatment visit	Treatment discontinued	RECIST-defined progression follow-up	Safety 90-day follow-up	Survival follow-up
	-28 days	Day 1	Every 4-8 weeks		Every 4-8 weeks		Every 4 weeks
Time window (days)			+/- 7 days	+/- 7 days	+/- 7 days		+/- 7 days
Informed consent	X						
Confirm driver gene mutation status (a)	X						
Demographics and baseline characteristics	X						
Medical/surgical history	X						
Inclusion/exclusion criteria check	X						
Enough matched tissue and/or blood for biomarker analysis (b)	X						
Patient demographics	X						
Medical history	X						
Disease characteristics	X						
Combination therapy	X	X	X	X	X	X	

	Screening/Enrollment Visit		Treatment visit	Treatment discontinued	RECIST-defined progression follow-up	Safety 90-day follow-up	Survival follow-up
Complete blood count	X		X	If clinically indicated	If clinically indicated	If clinically indicated	
Biochemical tests in blood and urine (c)	X		X	X	X	X	
Thyroid function	X		X	X	X	X	
Coagulation	X		X	X	X	X	
Pregnancy test (only for premenopausal female patients)	X (d)						
Physical examination	X		X	X	X	X	
WHO fitness status	X		X	X	X		
ECG record	X		X	X	X	X	
Echocardiogram/MUGA(e)	X		X	X	X	X	
Vision check (slit lamp) (f)			If clinically indicated	If clinically indicated	If clinically indicated	If clinically indicated	
Chest CT	X		X	X	X		
Abdominal CT/BUS (g)	X		If clinically indicated	If clinically indicated	If clinically indicated		
Pelvis CT/BUS	X		If clinically indicated	If clinically indicated	If clinically indicated		
Brain CT/enhanced MRI	X		If clinically indicated	If clinically indicated	If clinically indicated		
Bone ECT	X		If clinically indicated	If clinically indicated	If clinically indicated		
Tumor sample and/or blood	If				If applicable to PD		

	Screening/Enrollment Visit		Treatment visit	Treatment discontinued	RECIST-defined progression follow-up	Safety 90-day follow-up	Survival follow-up
sample	applicable to the baseline						
Tumor evaluation (h)			X (every 4 weeks)	X	X (every 4 weeks)		
SAE, including overdose and pregnancy reports (d)(i)		X	X	X	X	X	
Adverse events		X	X	X	X	X	
Exit and reason				X			
Request for survival status information							X

(a) The driver gene mutation test may have been completed during the screening period.

(b) Tumor sample and blood sample collection: sufficient matched tissue and/or blood for biomarker analysis (formalin fixed paraffin embedded (FFPE) section or fresh tumor tissue or blood, enough for next generation sequencing (NGS) analysis). But the acquisition of tissue is not mandatory when the disease progresses.

(c) Serum or plasma: creatinine, total bilirubin, alkaline phosphatase, aspartate aminotransferase, alanine aminotransferase, albumin, potassium, magnesium, total calcium, sodium, creatine kinase; urine analysis (test paper): hemoglobin/red blood cell/blood, protein/albumin, glucose.

(d) The pregnancy test should be performed in accordance with Chinese standards.

(e) Perform an echocardiogram or multiple uptake gated acquisition (MUGA) scan to assess LVEF at least every 16 weeks during screening (before the first dose) and throughout the treatment period. The form of cardiac function assessment of individual patients must be consistent, that is, if it is used for screening and evaluation of echocardiography, echocardiography should also be used for subsequent scans. It should also be possible to use the same machine and operator

to examine the patient and perform quantitative measurements. If the treatment evaluation performed when the study treatment is stopped is abnormal, a 28-day follow-up evaluation is required to confirm the reversibility of the abnormality.

(f) Patients showing signs and symptoms suggestive of keratitis, such as acute or worsening: eye inflammation, tearing, sensitivity to light, blurred vision, eye pain and/or red eyes should be immediately referred to an ophthalmologist.

(g) Abdominal CT/Bultrasound (BUS): If no lesions are detected at baseline, BUS can be used as a method to identify abdominal lesions; however, if lesions have been detected at baseline and the lesion is the target lesion, BUS should not be used as a measurement Methods: CT scan should be used consistently during the screening and follow-up period as a tumor assessment method; for new lesions detected by BUS, it is recommended to confirm by CT. It is recommended to use brain MR as a diagnostic method and should be used during follow-up.

(h) Tumor evaluation: The investigator uses radiology to evaluate the tumor. However, all aspects of the tumor should be fully covered and recorded initially according to RECIST version 1.1 during the screening period. After that, during the follow-up period, another radiological examination should be performed every 4 to 8 weeks (±7 days). For the same disease, whether it is the screening period or the follow-up period, the same evaluation techniques and methods should be used. Imaging tests used for tumor evaluation include: chest CT scan, abdominal CT scan/BUS, brain-enhanced MRI, and bone ECT (if clinically indicated).

(i) After stopping the study drug, SAEs related to the study procedure should continue to be collected until the disease progresses.

8.2. Enrollment/screening period

a. The enrollment/screening period is 28 days before the start of the study drug. The investigator will determine the eligibility of the patient based on the inclusion/exclusion criteria. The investigator will inform the patient about the potential benefits and risks of the study drugs (trametinib and anlotinib), AE reports, and other information collected within the scope of the study. Subsequently, the researcher will obtain an informed consent form signed by potential patients or their

legally acceptable representatives in accordance with Chinese law.

b. Driver gene mutations: the existence of driver gene mutations should be determined before starting to use investigational drug therapy. The inspection will be performed by NGS or another method (PCR-based method for EGFR detection and fluorescent in situ hybridization (FISH) or immunohistochemistry (IHC) for ALK rearrangement).
c. Baseline demographic characteristics: patient characteristics including age, gender, etc. will be collected.
d. Related medical history: comorbidities and related medical history (including any chronic diseases that currently require medical treatment).
e. Physical examination: it will be recorded at the baseline, and every visit (every 4 weeks) needs to be recorded.
f. Body weight: It will be recorded at the baseline, and every visit (every 4 weeks) needs to be recorded once.
g. Disease characteristics: tumor histology, diagnostic staging, current stage of enrollment study.
h. Medical history: The type of neoadjuvant therapy or adjuvant therapy (if any) received (targeted or non-targeted) and drugs received, start and end date, radiotherapy (yes/no), must be recorded before enrollment, Surgery (yes/no).
i. Related concomitant medications: For SAE, AEs of special concern, AEs that lead to dose adjustment/discontinuation of study drugs.
j. Laboratory evaluation: The researcher evaluates the laboratory parameters as normal or abnormal, and fills in the CRF with detailed information about the abnormality.
k. ECG recording: it will be recorded in the baseline, if there are clinical indications, it needs to be repeated.
l. Visual inspection: it will be recorded in the baseline, if there are clinical indications, it will need to be performed again.
m. Tumor evaluation: A baseline tumor evaluation should be performed within 28 days

before starting the study drug. The imaging evaluation performed at baseline should include: chest CT scan, abdominal CT scan/BUS, brain CT/enhanced MRI (if clinical indications), bone ECT (if clinical indications), fluorodeoxyglucose (FDG) positron emission tomography CT (PET-CT, if any Clinical indications).

AE/SAE: It should be recorded after international classification of functioning (ICF) signature.

8.3. Treatment period

The supply of study medication must always precede the treatment visit.

In addition, during each visit:

Need to screen patients for reportable safety events.

Tumor evaluation (effectiveness evaluation) needs to be recorded in the CRF.

The investigator must evaluate the patient's physical status and confirm that the patient continues to benefit from the study drug when providing the study drug.

If the patient withdraws from treatment and/or completely withdraws from the study, the investigator will record the reason for withdrawal. Please note that if the reason for withdrawal is an event that meets the SAE definition, it must be recorded in the safety and clinical form.

For patients who are lost to follow-up, all reasonable efforts must be made to track toxicity, determine their survival status, and record it in the CRF.

Study drugs must be properly stored and distributed from a safe storage area (or central pharmacy). The research center must maintain an inventory record of the receipt, distribution, destruction, and return of medicines.

Tumor evaluation: The investigator uses radiology to evaluate the tumor. However, all aspects of the tumor should be fully covered and recorded initially according to RECIST version 1.1 during the screening period. After that, radiological examinations should be performed every 4 to 8 weeks (±7 days) during the follow-up period. For the same disease, whether it is the screening period or the follow-up period, the same evaluation techniques and methods should be used. Imaging examinations used for

tumor evaluation include: chest CT scan, abdominal CT scan/BUS (if clinical indications), brain-enhanced MRI (if clinical indications), and bone ECT (if clinical indications).

8.4. Safety follow-up period

The patient must be followed up during the designated follow-up period (30 days after the last dose of study drug is administered) to collect SAEs that occurred during this period, and provide other reports as required.

8.5. Progressive follow-up period

After the study drug is discontinued for reasons other than disease progression, the patient will continue to be evaluated every 4 weeks until objective progress is achieved, the next course of treatment is started or the follow-up is lost, whichever occurs first. Patients who continue to receive treatment after achieving objective progress due to clinical benefits will be assessed for tumors and the investigator's response assessment will be collected.

Blood samples and/or tumor tissue can be collected when disease progression is achieved.

AE/SAE/radiation-induced damage: It should be collected from the informed consent of the entire treatment period, including the follow-up period.

8.6. Survival follow-up period

As the disease progresses, the patient, the patient's family or the patient's current doctor must be contacted every 4 weeks for survival information. The patients who were lost to follow-up whose last contact status was recorded as "alive" will be reviewed.

9. Research Evaluation

The investigator should ensure that the data is recorded on the case report form specified in the research protocol and recorded in accordance with the instructions provided.

Researchers ensure the accuracy, completeness and timeliness of the recorded data, and respond to data queries. The investigator will sign the case report form. A copy of the complete medical record report form will be archived in the research center.

9.1. Effectiveness evaluation

The RECIST version 1.1 standard will be used to assess the patient's response to treatment by determining the ORR, disease control rate (DCR), duration of response (DOR) and PFS.

Within 28 days after starting treatment, CT/BUS of the chest and abdomen (including liver and adrenal glands) at baseline were used for imaging evaluation. Then check every 4 to 8 weeks ± 7 days until objective disease progression occurs or the study is withdrawn. In addition, other areas should be checked based on the patient's signs and symptoms. Any other areas suspected of having a new disease should also undergo appropriate imaging examinations. If an unplanned evaluation is performed and the patient has no disease progression, every effort should be made to conduct a follow-up evaluation at the scheduled visit.

The classification of objective tumor response assessment will be based on RECIST version 1.1 response criteria: CR, PR, SD and PD. Calculate the progression of the target lesion (TL) and compare it with the time when the tumor burden is minimal (that is, the sum of the smallest diameters previously recorded in the study). In the absence of disease progression, the tumor response (CR, PR, SD) is calculated and compared with the baseline tumor measurements obtained before starting treatment. CR/PR/SD should be confirmed at least 4 weeks later and accompanied by the follow-up interval specified in the study plan.

If there is progressive disease, the progress will be recorded as a visit response (RECIST version 1.1).

If the investigator has doubts about the occurrence of disease progression, especially the response to NTL (non-target lesion) or the emergence of new lesions, it is recommended to continue treatment until the next planned evaluation or earlier (if there are clinical indications), and reassess the patient's status. If repeated scans confirm disease progression, the date of the initial scan should be declared as the date of disease progression.

In order to be judged as "clearly progressing" on the basis of non-targeted diseases, non-targeted diseases must have an overall level of substantial deterioration. In this way, even if the targeted disease has SD or PR, the overall tumor burden has been increased enough and it is worth stopping treatment. A moderate "increase" in the size of one or more non-target lesions is usually not enough to ensure a clear state of disease progression.

It is important to follow the assessment schedule as strictly as possible.

9.2. Safety assessment

9.2.1. Laboratory safety assessment

The laboratory safety assessment parameters are listed in the table:

Clinical chemistry	Hematology
Serum (S) / Plasma (P)-Albumin	Blood (B)-hemoglobin
S/P-ALT	B-leukocytes
S/P-AST	B-hematocrit
S/P-Alkaline Phosphatase	B-Red blood cell (RBC) count
S/P-total bilirubin	B-Absolute white blood cell classification count:
S/P-total calcium	Neutrophils
S/P-creatinine	Lymphocytes
S/P-glucose (fasting only on PK days)	Monocyte
S/P-Lactate Dehydrogenase (LDH) 2	Basophils
S/P-magnesium	Eosinophils
S/P-potassium	B-platelet count
S/P-Sodium	B-reticulocyte
S/P-urea nitrogen or blood urea nitrogen	Urinalysis
S/P-lipase	U-glucose
S/P-Amylase	U-protein
S/P-total cholesterol	U-blood
S/P-total triglycerides	Coagulation
S/P- High Density Lipoprotein	P-prothrombin time (PT)
S/P- Low Density Lipoprotein	P-activated partial thromboplastin time (APTT)
Thyroid function	P-thrombin time (TT)
S-Triiodothyronine (T3)	P-fibrinogen (Fbg)
S-thyroxine (T4)	P-D-dimer
S-TSH	P- INR
S-free T3	
S-free T4	

Blood and urine samples will be collected at the time specified in the study plan to determine clinical chemistry, hematology, thyroid function, coagulation function, and urinalysis. If the researcher believes that there are clinical indications, other safety samples can be collected. Acquisition date, time and results (value, unit and reference range) will be recorded in the appropriate CRF.

Clinical chemistry, hematology, thyroid function, coagulation function and urinalysis will be performed in Shanghai Chest Hospital, Shanghai Jiao Tong University School

of Medicine.

In addition, during the screening visit, pregnancy tests are only performed on women with reproductive potential (according to the standard clinical practice of the research center, blood or urine tests are acceptable).

Researchers should evaluate existing results regarding clinically relevant abnormalities. Laboratory results should be signed and dated, and stored in the research center as the source data of laboratory variables.

Electrolyte abnormalities (hypokalemia, hypomagnesemia, hypocalcemia) must be corrected to within the normal range before the first dose, and electrolyte levels should be monitored during the study treatment.

9.2.2. Physical examination

A physical examination will be performed, including evaluation of the following: general appearance, skin, head and neck (including ears, eyes, nose, and throat), respiratory system, cardiovascular, abdomen, lymph nodes, thyroid, abdomen, and central nervous system.

9.2.3. Electrocardiogram

All patients will undergo a 12-lead digital electrocardiogram during the study visit. After the patient rests on the supine side for at least 10 minutes before the specified time, a 12-lead ECG will be obtained, which should be recorded at a speed of 25 mm/sec. All ECGs should be recorded in the same position of the patient. For each time point, three ECG results should be recorded in approximately 5 minutes. The digital ECG records will be collected, analyzed and stored by the central ECG supplier. If there is an abnormality in the evaluation during the treatment when the study treatment is stopped, a 28-day follow-up evaluation is required to confirm the reversibility of the abnormality.

9.2.4. Echocardiography/MUGA Scan

Echocardiography or MUGA scans will be performed at screening (before the first treatment) and at least every 16 weeks throughout the treatment period to assess LVEF. The method of cardiac function assessment must be consistent within the patient (i.e., if echocardiography is used for screening assessment, echocardiography should also be used for subsequent scans). As far as possible, the same machine and operator should be used to examine patients and perform quantitative measurements. If there is an abnormality in the evaluation during the treatment when the study treatment is stopped, a 28-day follow-up evaluation is required to confirm the reversibility of the abnormality.

9.2.5. Vital signs

Pulse and BP

After resting for 10 minutes, BP and pulse rate in the supine position will be measured. As indicated in the research plan, evaluation will be performed at the time of the visit. In addition, if there are clinical indications, it should be at the discretion of the investigator. If applicable, any change in vital signs should be recorded as an AE.

Weight and height

Body weight assessment will be performed at the time of screening, and then on day 1 of each cycle and at the discontinuation visit. Evaluate height only during screening.

10. Safety report and medical management

The principal investigator is responsible for ensuring that all personnel involved in the research are familiar with the content of this section.

10.1. Definition of adverse events

AEs refer to adverse medical conditions or deterioration of the original medical conditions after or during exposure to the study drugs, regardless of whether there is a causal relationship with the drug. A bad medical condition may be symptoms (for example, nausea, chest pain), physical signs (for example, tachycardia, enlarged liver), or abnormal test results (for example, laboratory test results, electrocardiogram). In clinical studies, AEs include adverse medical conditions that occur at any time, including the lead-in period or wash-out period, even if no research treatment has been performed.

The term AE includes serious and non-serious AEs.

10.2. Definition of serious adverse events

SAE refer to AEs that occur during any research phase (i.e., lead-in period, treatment period, washout period, follow-up period), which meet one or more of the following criteria:

Causes death

Immediate life threatening

Need to be hospitalized or extend the current hospital stay

Cause permanent or severe disability/loss of function or severely disrupt normal life functions

Congenital malformations or birth defects

It is a major medical event that may endanger the subject or may require medical intervention to prevent one of the above outcomes

10.3. Adverse Event Record

10.3.1. Time period for collecting adverse events

AEs and SAEs will be collected from the time of informed consent throughout the treatment period, including the follow-up period (90 days after the last study drug administration).

10.3.2. Follow-up of unresolved adverse events

In the study, the investigator will follow up any AEs that the subject has not resolved in the last AE assessment based on medical indications, but no further records are required in the CRF.

10.3.3. Variables

The severity of AE is classified according to NCI CTCAE 5.0, using a 5-point scale (1-5 grades), and is reported in detail on CRF. The grades of AEs not included in CTCAE are as follows:

CTCAE grade	Equal:	definition
Level 1	Mild	Feel uncomfortable and do not interfere with normal activities of daily life.
Level 2	Moderate	Feeling of discomfort is sufficient to reduce or affect activities of daily living; treatment or medical intervention is not indicated, although these measures can improve the patient's overall health or symptoms.
Level 3	Severe	Inability to work or normal activities of daily life; treatment or medical intervention is required to improve the patient's overall health or symptoms; delay in treatment has no direct harmful effect on the patient's survival.
Level 4	Life-threatening or disabling	Directly endanger life or cause permanent mental or physical illness, affecting work or normal activities of daily life; treatment or medical intervention is required to maintain life.
Level 5	Fatal	AE causes death

10.3.4. Causality collection

The investigator will evaluate the causal relationship between the trial drug and each AE, and answer "yes" or "no" to the question "Do you think the event may be caused by the trial drug?"

For SAE, the causal relationship between other drugs and the research process will also be evaluated. Please note that for SAEs that may be related to any research process, causality is "yes".

For guidance on the interpretation of causality issues, please see Appendix A of the "Clinical Research Protocol".

10.3.5. Adverse events based on signs and symptoms

All AEs reported by the subject spontaneously or when answering open-ended questions from the researcher: "Did you have any health problems since the last visit/last interview?" or found through observation. The information will be collected and recorded in the CRF. When collecting AEs, it is best to record the diagnosis, not the signs and symptoms. However, if the diagnosis result is known and there are other signs or symptoms that are not normally included in the diagnosis, the diagnosis and each sign or symptom will be recorded separately.

10.3.6. Adverse events based on inspections and trials

The results of laboratory tests and vital signs specified in the protocol will be summarized in the clinical study report. Therefore, compared to the baseline set by the protocol, if the deterioration meets any SAE criteria or the reason for discontinuation of the trial drug treatment, it should only be reported as an AE.

If the deterioration of laboratory test values/vital signs is related to clinical signs and symptoms, the signs or symptoms will be reported as an AE, and the related laboratory results/vital signs will be treated as other information. Investigators use clinical terms as much as possible for reporting, rather than laboratory terms (for example, anemia

and low hemoglobin values). In the absence of clinical signs or symptoms, clinically relevant deterioration of non-mandatory parameters should be reported as an AE. Deterioration of laboratory test values clearly due to disease progression should not be reported as AE/SAE.

Compared with the baseline assessment, any new or aggravated clinically relevant abnormal medical results found during the physical examination will be reported as an AE.

10.3.7. Disease progression

Disease progression can be considered to be the deterioration of the subject's disease caused by the disease under investigation by the experimental drug. It may be an increase in the severity of the disease under study and/or an increase in disease symptoms. New metastasis or progression to existing metastases should be considered disease progression, not AEs. Events that are clearly due to disease progression should not be reported as AEs during the study period.

The progression of the malignant tumor in the study, including the progression of signs and symptoms, should not be reported as a serious AE. Hospitalization due to signs and symptoms of disease progression should not be reported as a serious AE.

10.4. Report of serious adverse events

The investigator is responsible for notifying the ethics committee and sponsor of any SAE.

The investigator must inform the local ethics committee of any SAE within 24 hours.

All SAEs that lack important or relevant information should be followed up immediately. The investigator should inform the sponsor representative of any follow-up information of previously reported SAEs within 24 hours after learning.

11. Statistical methods

The analysis will be performed by the researcher or other designated third-party provider. A comprehensive statistical analysis plan (SAP) will be prepared, and any subsequent revisions will be recorded and final revisions will be made before reporting the data. All data will be provided for the complete analysis set. All patients who received at least 1 dose of study drug will be included in the study analysis.

11.1. Definition of study endpoint

In order to achieve the purpose of this study, data on the following endpoints will be collected:

Part A

Primary endpoint: To define the RP2D of the combined strategy of trametinib and anlotinib for advanced NSCLC patients with KRAS mutation (excluded $KRAS^{G12C}$).

Secondary endpoint: To evaluate the ORR, PFS, OS and safety of the combined strategy of trametinib and anlotinib for advanced NSCLC patients with KRAS mutation (excluded $KRAS^{G12C}$).

Part B

Primary endpoint: To evaluate the ORR of the combined strategy of trametinib and anlotinib for advanced NSCLC patients with KRAS mutation (excluded $KRAS^{G12C}$).

Secondary endpoint: To evaluate the PFS, OS and safety of the combined strategy of trametinib and anlotinib for advanced NSCLC patients with KRAS mutation (excluded $KRAS^{G12C}$).

Exploratory endpoint: dynamic changes of biomarkers and mutation profiles related to the efficacy and drug resistance of trametinib combined with anlotinib

11.2. Calculation of sample size

The primary endpoint of this study was to determine RP2D based on the incidence of DLT in cycle 1. For the dose-escalation stage (Part A), "3 + 3" principle was used. The sponsor and investigators will review the safety and clinical data of all subjects to jointly determine RP2D of anlotinib combined with trametinib. If RP2D is reached in Part A, another 20 eligible patients will be enrolled and treated with trametinib (2mg QD, oral) + anlotinib (RP2D, once a day, from day 1 to day 14 of the 21-day cycle), until the PD or unacceptable toxicity occurs to further evaluate the safety, tolerability and efficacy.

11.3. Statistical analysis

All data will be provided for the FAS. All patients who received at least one dose of study drug will be included in the study analysis. For all variables, descriptive statistics will be performed as appropriate. Continuous variables will be summarized by observations, mean, standard deviation, median, minimum, and maximum. Categorical variables will be aggregated by frequency counts and percentages for each category.

The FAS includes all patients who received at least one dose of study drug. The FAS will be used for all efficacy and safety analysis.

The PPS is a subset of FAS, including all cases that meet the research protocol, has good compliance, does not use any prohibited drugs during the research process. The set of compliance protocols will be used for supporting analysis of effectiveness endpoints.

- Safety and tolerability

The safety analysis set will include all patients who have received at least one dose of the therapeutic drug. The patient will be evaluated based on the actual treatment received. The safety and tolerability summary will be based on the safety analysis set.

AE will be coded according to the preferred term of SOC and MedDRA terminology. The severity of AE will be graded according to NCI CTCAE version 5.0. A list of all

AEs will be listed, including detailed information collected for each AE. (Description of event, date/time of onset, duration, severity, severity, relationship with study drug, measures taken, clinical outcome).

- Tumor response

Tumor response data will be listed and summarized by dose group, and the following response categories will be used when appropriate: CR, PR, SD, PD, and NE. For the definitions of CR, PR, SD and PD, please refer to the RECISIT version 1.1 standard. Where appropriate, the objective tumor response rate is given with a 95% confidence interval (calculated using the Clopper-Pearson interval).

- Duration of response

The response duration of the responding patients will be summarized, and the number of responding patients (%) for the duration of remission> 3;> 6;> 9;> 12 months will be given. The Kaplan Meier chart and the median duration of response with 95% confidence interval (95% CI, calculated based on the Kaplan Meier chart) will be given.

- PFS and OS

The PFS of the expansion phase will be summarized. PFS will be displayed using Kaplan-Meier curves. The number of events, the median (calculated based on the Kaplan-Meier curve), and the proportion of patients with no events at 6, 12, and 18 months will be summarized. Where appropriate, a summary of the number and percentage of patients who have died, are still undergoing follow-up, lost follow-up, and dropped out of the study will be provided.

- Biomarkers and mutation profiles

All patients participating in the biomarker study will be included in the analysis set.

12. Ethical considerations

This research will be conducted in accordance with legal and regulatory requirements, as well as the International Code of Ethics for Biomedical Research Involving Human Subjects (International Council of Medical Scientific Organizations, 2002) and the Declaration of Helsinki (World Medical Association, 1996 and 2008). In addition, this research will be conducted in accordance with the plan and applicable Chinese regulatory requirements and laws.

Unless required by law, all parties should ensure the protection of the subject's personal data and must not include the subject's name in any sponsor form, report, publication or any other disclosure. The subject's name, address, date of birth, and other identifiable data will be replaced with an alphanumeric code composed of a numbering system. The informed consent must comply with International Conference on Harmonization (ICH) GCP, regulatory requirements and legal requirements. The informed consent used in this study and any changes made during the study must be pre-approved by Institutional Review Board (IRB)/ Independent Ethics Committee (IEC) before use. Researchers must ensure that each research subject or his legal representative fully understands the nature and purpose of the research and the possible risks associated with participation. The researcher or the personnel designated by the researcher shall obtain the written informed consent of each subject or the subject's legal representative before conducting any specific research activities. The investigator will retain the original copy of the informed consent that each subject has signed.

References

1. M. Drosten, M. Barbacid, Targeting the MAPK Pathway in KRAS-Driven Tumors. *Cancer Cell* **37**, 543-550 (2020).
2. P. Liu, Y. Wang, X. Li, Targeting the untargetable KRAS in cancer therapy. *Acta Pharm Sin B* **9**, 871-879 (2019).

3. D. S. Hong *et al.*, KRAS(G12C) Inhibition with Sotorasib in Advanced Solid Tumors. *N Engl J Med* **383**, 1207-1217 (2020).
4. A. R. Moore, S. C. Rosenberg, F. McCormick, S. Malek, RAS-targeted therapies: is the undruggable drugged? *Nat Rev Drug Discov* **19**, 533-552 (2020).
5. P. A. Janne *et al.*, Selumetinib Plus Docetaxel Compared With Docetaxel Alone and Progression-Free Survival in Patients With KRAS-Mutant Advanced Non-Small Cell Lung Cancer: The SELECT-1 Randomized Clinical Trial. *Jama* **317**, 1844-1853 (2017).
6. E. Manchado *et al.*, A combinatorial strategy for treating KRAS-mutant lung cancer. *Nature* **534**, 647-651 (2016).
7. C. Sun *et al.*, Intrinsic resistance to MEK inhibition in KRAS mutant lung and colon cancer through transcriptional induction of ERBB3. *Cell reports* **7**, 86-93 (2014).
8. A. W. Tolcher *et al.*, A phase IB trial of the oral MEK inhibitor trametinib (GSK1120212) in combination with everolimus in patients with advanced solid tumors. *Annals of oncology : official journal of the European Society for Medical Oncology* **26**, 58-64 (2015).
9. H. Singh, D. L. Longo, B. A. Chabner, Improving Prospects for Targeting RAS. *Journal of clinical oncology : official journal of the American Society of Clinical Oncology* **33**, 3650-3659 (2015).
10. C. Fedele *et al.*, SHP2 Inhibition Prevents Adaptive Resistance to MEK Inhibitors in Multiple Cancer Models. *Cancer Discov* **8**, 1237-1249 (2018).
11. Y. Sun *et al.*, Safety, pharmacokinetics, and antitumor properties of anlotinib, an oral multi-target tyrosine kinase inhibitor, in patients with advanced refractory solid tumors. *Journal of hematology & oncology* **9**, 105 (2016).
12. B. Han *et al.*, Effect of Anlotinib as a Third-Line or Further Treatment on Overall Survival of Patients With Advanced Non-Small Cell Lung Cancer: The ALTER 0303 Phase 3 Randomized Clinical Trial. *JAMA Oncol* **4**, 1569-1575

(2018).

13. Y. Chi *et al.*, Safety and Efficacy of Anlotinib, a Multikinase Angiogenesis Inhibitor, in Patients with Refractory Metastatic Soft-Tissue Sarcoma. *Clinical cancer research : an official journal of the American Association for Cancer Research* **24**, 5233-5238 (2018).

14. D. Wu *et al.*, A phase II study of anlotinib in 45 patients with relapsed small cell lung cancer. *Int J Cancer* **147**, 3453-3460 (2020).

Appendix material

Appendix A Other safety information

Further guidance on the definition of SAE

- Life threatening

"Life-threatening" means that the subject is immediately at risk of death when an AE occurs, or it is suspected that the use or continued use of the drug will cause the subject's death. "Life-threatening" does not mean that the AE occurs in a more serious form, which may lead to death (for example, hepatitis that has healed without liver failure).

- Hospitalization

Outpatient treatment in the emergency room itself is not a serious AE, although the cause may be (for example, bronchospasm, laryngeal edema). If the disease or disease existed before the subject was included in the study, and the disease did not worsen in an unexpected manner during the study, the hospitalization and/or surgery planned before or during the study is not considered an AE.

- Major medical incident or medical intervention

Major medical events may not immediately endanger life or cause death, hospitalization, disability or incapacity, but may endanger the subject or may require medical intervention to prevent one or more of the outcomes listed in the definition of a serious AE Under the circumstances, medical and scientific judgments should be made to determine whether the case is serious. These should generally be considered serious.

Merely stopping the use of suspicious drugs does not mean that this is a major medical incident; medical judgment must be used.

• Angioedema is not severe enough to require intubation, but requires intravenous hydrocortisone treatment

• Hepatotoxicity caused by paracetamol (acetaminophen) overdose requires N-acetylcysteine treatment

• Intensive treatment of allergic bronchospasm in the emergency room or at home

• Unbalanced blood quality (such as neutropenia or anemia that requires blood

transfusion) or convulsions, but will not lead to hospitalization

- A guide to explaining causality issues

When evaluating causality, the following factors need to be considered to determine whether there is a "reasonable possibility" that the drug may cause an AE.

• Time course. Exposure to suspicious drugs. Did the subject actually take the suspicious drug? Does the AE have a reasonable time relationship with the taking of the suspicious drug?

• Consistent with known drug characteristics. Are AEs consistent with previous knowledge of suspected drugs (pharmacology and toxicology) or drugs of the same pharmacological category? Or can AE be predicted from its pharmacological properties?

• Experience to stimulate. Did the AE heal or improve when the dose of the suspected drug was stopped or reduced?

• There is no other reason. AE cannot be reasonably explained by other causes (such as underlying diseases, other drugs, other hosts, or environmental factors).

• Reinvigorating experience. If the suspicious drug is reintroduced after the drug is stopped, will the AE happen again? It is generally not recommended to re-inspire.

• Laboratory testing. A specific laboratory investigation (if performed) has confirmed this relationship.

- In difficult situations, other factors can be considered, such as:

• Is this a recognized feature of drug overdose?

• Is there a known mechanism of action?

"Relevant" causality refers to the "reasonable possibility" of causality in individual cases after reviewing relevant data. The term "reasonable possibility" of causality usually refers to facts (evidence) or arguments that indicate the existence of causation. Perform causality assessment based on existing data that includes enough information to make informed judgments. If the information in the case is limited or insufficient, the incident may be assessed as "irrelevant."

The causality of the deterioration of the research disease due to lack of validity should be classified as unreasonable possibility.

Appendix B Guidance on potential interactions of concomitant drugs

The use of any natural/herbal products or other "civil treatments" is discouraged, but the use of these products and the use of all vitamins, nutritional supplements and all other combined drugs must be recorded in the electronic documents.

It is strongly recommended that drugs that induce CYP3A4 metabolism should not be used in combination with anlotinib

Anlotinib is metabolized by CYP3A4 and CYP3A5 enzymes.

A drug-drug interaction study of anlotinib in patients showed that when co-administered with a strong inducer of CYP3A4, the effectiveness of anlotinib may be impaired. In vitro and in vivo studies have shown that trametinib is mainly metabolized by deacetylation alone or in combination with oxidation. The metabolites after deacetylation are further metabolized by glucuronidation. CYP3A4 oxidation is regarded as a secondary metabolic pathway. Deacetylation is mediated by carboxyl esterase 1b, 1c, and 2, and other hydrolases may also play a role. Based on the lower dose and lower clinical systemic exposure relative to the in vitro inhibitory value or induction value, trametinib is not considered to be an in vivo inhibitor or inducer of these enzymes or transporters, and it is unlikely to pass through the CYP enzyme or transporter. Protein interactions significantly affect the pharmacokinetics of other drugs.

In this study, any patient receiving anlotinib should not use the following strong CYP3A4 inducers.

Contraindicated drugs	Withdrawal period before starting study drug treatment
Carbamazepine, phenobarbital, phenytoin, rifampicin, rifabutin, rifapentin	3 weeks
St. John's Wort	
Phenobarbital	5 weeks

This list is not exhaustive, and similar contraindications will apply to other drugs known to strongly modulate the activity of CYP3A4. Appropriate medical judgment is required.

Appendix C CTCAE V5.0

V5.0CTCAE classification	Level 1	Mild; asymptomatic or mild; only clinical or diagnostic observation; or no intervention is indicated
	level 2	Moderate; minimal, partial or non-invasive intervention; or restrict age-appropriate instrumental activities of daily living
	Level 3	Severe or medically significant, but not immediately life-threatening; leading to hospitalization or prolonged hospitalization; disabled; or restricting self-care activities of ADL in daily life
	level 4	Life-threatening consequences or emergency intervention
	Level 5	AE-related deaths
Severity	A serious adverse event refers to any adverse event that meets one or more of the following criteria at any dose or during the administration of any study drug:	
	†Causes or causes death;	
	†Life-threatening; or, the researcher believes that the adverse event puts the patient at direct risk of death (Note: This does not include any adverse event that occurs in a more serious form or is allowed to continue to occur, which may lead to death.);	
	†Causes permanent or severe disability/loss of function (i.e., adverse events cause the patient's ability to perform normal life functions to be severely affected);	
	† is a congenital abnormality/birth defect (in the offspring of subjects who used the drug without considering the time of diagnosis);	
	†The investigator judged it to be a major medical event; although the event is not fatal, life-threatening or requires hospitalization, according to appropriate medical judgment, the event may endanger the subject and requires medical or surgical intervention to prevent it from occurring One of the above endings (marked as †). Such events can also be regarded as serious adverse events.	
	† is a congenital abnormality/birth defect (in the offspring of subjects who used the drug without considering the time of diagnosis);	
duration	Record the start and end date of the adverse event. If it is less than 1 day, please specify the appropriate time length and unit.	
the measures taken	Does the study of adverse drug events lead to the discontinuation of the study drug?	
Relationship with the product	Will study drugs cause adverse events? Medically qualified investigators should provide evaluation results of the relationship between study drugs and adverse events. The investigator should sign the name/date (first letter) on the original document or worksheet to support the evaluation of the causality of the adverse event and ensure that the evaluation of the causality meets the medical qualifications. The signed documents must be kept within the prescribed supervision period. The following standards will serve as a reference guide to help investigators assess the relationship between study drugs and adverse events based on existing information.	
	Exposed	Is there evidence that the subject has actually been exposed to the study drug, such as true past medical history, acceptable compliance assessment (drug count, log, etc.), expected pharmacological effects, and drug/metabolite effects in the specimens collected in the body Measurements?
	Time course	Is there a reasonable time sequence between AE and study drug treatment?
	possible reason	Does the adverse event occur at the same time as the adverse event caused by the drug?
	Deprovocation test	Has the study drug been discontinued or has the dose/exposure/frequency reduced?<br>If yes, is the AE cured or improved?<br>If it is, the deprovocation test result is positive. If it is not, the result of the de-provocation test is negative.<br>Note: If (1) an adverse event leads to death or permanent disability; (2) despite the continued use of the study drug, the AE can still be cured/improved; (3) the test is a single-dose drug test; (4) the study drug is only administered Once, the standard does not apply.
	Reprovocation	Is the subject repeatedly exposed to the study drug during the trial?

	test	If yes, does the AE recur or worsen? If it is, the result of the re-provocation test is positive. If it is not, the result of the re-provocation test is negative.<br>Note: If (1) the AE results in death or permanent disability, or (2) the test is a single-dose test, or (3) the study drug is administered only once, then this standard does not apply.<br>Note: Planning a reprovocation test for serious AEs that may be caused by the study drug or re-exposure to the study drug may pose a serious potential risk to the subject/patient. In this case, it is not recommended to conduct a re-provocation test unless the continued use of the study drug is beneficial to the patient, and there is no alternative treatment available, and the test can only be performed with the approval of the sponsor's clinical director.
	Consistent with research treatment characteristics	Are the clinical/pathological manifestations of the adverse event consistent with the previous data records of the study drug or the pharmacological and toxicological trials of such drugs?
A medically qualified investigator should report the evaluation results of the relationship between the study drug and AE in the case report form/worksheet based on his/her best clinical judgment, including consideration of the above factors.		
Causality record	Investigators should evaluate the possible correlation between adverse events and study drugs with reference to the following five criteria: 1) The occurrence of adverse events is consistent with the time of drug use; 2) The adverse events are related to the known adverse reactions of the study drugs; 3) Adverse events cannot be explained in other ways; 4) Adverse reactions disappear after drug withdrawal; 5) Adverse events recur after administration.<br>The result will be judged as "definitely relevant", "probably relevant", "may be relevant", "may not be relevant", "not relevant". The three results, which are determined to be related, likely to be related, and possibly related, are determined as adverse reactions, and the incidence of adverse events is calculated based on this. For details, see 5.2.4.	

Abbreviations

**Phrases and terminology**	**Explanation**
AE	Adverse event
AJCC	American Journal of Critical Care
ALT	Alanine aminotransferase
ANC	Absolute neutrophil count
APTT	Activated partial thromboplastin time
AST	Aspartate aminotransferase
BCRP	Blood C reactive protein
BP	Blood pressure
BUS	Bultrasound
CCr	Creatinine clearance rate
CI	Confidence interval
CNS	Central nervous system
CR	Complete response
CRF	Case Report Form
CT	Computed tomography
CTCAE	Common Terminology Criteria for Adverse Events
ctDNA	Circulating tumor DNA
CTEP	Cancer Therapy Evaluation Program
DCR	Disease control rate
DLT	Dose-limiting toxicity
DOR	Duration of remission
ECG	Electrocardiogram
ECOG	Eastern Cooperative Oncology Group Performance Status
EGFR	Epidermal growth factor receptor
EPO	Erythropoietin
FAS	Full analysis set
FBG	Fasting blood glucose
Fbg	Fibrinogen
FDG	Fluorodeoxyglucose
FFPE	Formalin fixed paraffin embedded
FISH	Fluorescent in situ hybridization
GCP	Good Clinical Practice
GMP	Good Manufacturing Practices
HIV	Human immunodeficiency virus
HRCT	High-resolution computed tomography
ICF	International classification of functioning
ICH	International Conference on Harmonization
IEC	Independent Ethics Committee

IHC	Immunohistochemistry
ILD	Interstitial lung disease
INR	International Normalized Ratio
IRB	Institutional Review Board
LDH	Lactate Dehydrogenase
LVEF	Left ventricular ejection fraction
MTD	Maximum tolerated dose
MRI	Magnetic resonance imaging
MUGA	Multiple uptake gated acquisition
NCI	National Cancer Institute
NGS	Next generation sequencing
NSCLC	Non-small cell lung cancer
NTL	Non-target lesion
ORR	Objective response rate
OS	Overall survival
PD	Progression disease
PET-CT	Positron emission tomography computed tomography
PFS	Progression free survival
Pgp	P-glycoprotein
PPS	Per protocol set
PR	Partial response
PT	Prothrombin time
QD	*Quaque die*
RBC	Red blood cell
RECIST	Response Evaluation Criteria in Solid Tumors
RP2D	Recommended phase 2 dose
RTK	Receptor tyrosine kinase
SAE	Serious adverse event
SAS	Safety analysis set
SD	Stable disease
SOC	System organ category
SRC	Scientific review committee
T3	S-Triiodothyronine
T4	S-thyroxine
TL	Target lesion
TKI	Tyrosine kinase inhibitor
TNM	Tumor Node Metastasis
TSH	Thyroid Stimulating Hormone
TT	Thrombin time
UICC	Union for International Cancer Control
ULN	Upper limit of normal
WHO	World Health Organization

Summary of changes for protocol

Document	Version date	Summary of changes
Original protocol, Version 1.0	2021.02.21	N/A
Amendment 1, Version 2.0	2021.07.15	1. Update the evaluation cycle. Add: "After the investigators' evaluation, the assessment cycle can extend to 12 weeks or longer due to the uncontrollable factors are also be accepted during the treatment period."
Amendment 2, Version 3.0	2022.05.27	1. Update the inclusion criteria. Add 2 items in inclusion criteria, "Part A: advanced NSCLC patients with non-G12C mutations who have previously received standard treatment or treatment naïve; Part B: advanced NSCLC patients with non-G12C mutations who have previously received standard 1st or more treatment".<br>2. Update the dosage design in part A. Add a group of anlotinib (6mg) plus trametinib (2mg). The combined doses of trametinib (2mg, QD) and anlotinib (6mg, 8mg, 10mg, 12mg, QD, the first day to the 14th day of the 21-day cycle) respectively.<br>3. Update the "Calculation of sample size". Part A: "The primary endpoint of this study was to determine RP2D based on the incidence of DLT in cycle 1. For the dose-escalation stage (Part A), "3 + 3" principle was used. Briefly, trametinib is set to a single dose (2mg QD, oral) throughout the part A. Anlotinib is set to four levels of dose (6mg,

		8mg, 10mg, 12mg, once a day, from day 1 to day 14 of the 21-day cycle). Three patients will be enrolled in primary combination treatment (trametinib, 2mg QD, oral; anlotinib, 6mg, once a day, from day 1 to day 14 of the 21-day cycle). If one patient occurs DLT in cycle 1, another 3 patients will be enrolled to further verification the safety. If two or more patients occur DLT in cycle 1, it is suggested that the exploration can be carried out in a lower dose group. If no patient occurs DLT in cycle 1, next dose combination treatment will be performed. If two patients screened and enrolled in a same dose group simultaneously, four patients in a single dose group are allowable. The sponsor and investigators will review the safety and clinical data of all subjects to jointly determine RP2D of anlotinib combined with trametinib". Part B: "If RP2D is reached in Part A, another 20 eligible patients will be enrolled and treated with trametinib (2mg QD, oral) + anlotinib (RP2D, once a day, from day 1 to day 14 of the 21-day cycle), until the PD or unacceptable toxicity occurs to further evaluate the safety, tolerability and efficacy. The primary endpoint for evaluation of efficacy of part B is ORR. The standard-of-care for advanced NSCLC patients with non-G12C KRAS mutations treated in this setting is docetaxel, which is associated with an ORR of up to 23%. Assuming trametinib plus anlotinib will result in an ORR of at least 55%

		in this setting, a sample size of approximately 20 evaluable patients would be sufficient for the lower bound of a 2-sided 95% confidence interval (Clopper-Pearson method) to exclude an ORR of 23%. If an ORR of at lower 40%, the part B study will be terminated".<br>4. Add the effects of COVID-19 on the clinical trial. Add: "For the patients who discontinued the therapy due to COVID-19 infection or COVID-19-related reasons, they will continue to receive trametinib plus anlotinib therapy if the investigators think the patients still can receive benefit from the medication".
Amendment 3, Version 4.0	2022.05.09	1. Delete the PPS-related analysis in the statis methods section.<br>2. Re-define the tumor response: "Tumor response data will be listed and summarized by dose group, and the following response categories will be used when appropriate: complete response (CR), partial response (PR), stable disease (SD), PD, and non-evaluable (NE). For the definitions of CR, PR, SD and PD, please refer to the RECISIT version 1.1 standard. Where appropriate, the objective tumor response rate will be given with a 95% confidence interval (calculated using the Clopper-Pearson interval).<br>Objective response rate (ORR) is defined as the percent of patients documented to have a confirmed CR or PR.<br>Descriptive statistics (frequency and percentage) for ORR based on response assessments by investigator. Patients who

		cannot be assessed for response will be counted as not evaluable. Descriptive statistics (frequency and percentage) for CR and PR rate will be presented overall".<br>3. Re-define the duration of response: "Duration of Response (DOR) in months is defined as the time from date of the first documentation of objective response (CR or PR) to the first documentation of PD or to death due to any cause in the absence of documented PD. DOR will only be calculated for the subgroup of patients achieving an efficacy of CR or PR. The response duration of the responding patients will be summarized, and the number of responding patients (%) for the duration of remission> 3; > 6; > 9; > 12 months will be given. The Kaplan Meier chart and the median duration of response with 95% confidence interval (95% CI, calculated based on the Kaplan Meier chart) will be given. DOR will be evaluated based on response assessments by the investigator".<br>4. Re-define the progression-free survival: "PFS is defined as the time from the date of first treatment to the date of first PD or death due to any cause in the absence of documented PD, whichever occurs first. PFS (in days or months) will be calculated.<br>The PFS of the part A, part B, overall, subgroups will be summarized. PFS will be displayed using Kaplan-Meier curves.<br>PFS will be displayed using Kaplan-Meier curves. The number of events, the median

		(calculated based on the Kaplan-Meier curve), and the proportion of patients with no events at 6, 12, and 18 months will be summarized.<br>Where appropriate, a summary of the number and percentage of patients who died, are still under follow-up, lost to follow-up, and withdrawn from the study will be provided. For the patients who discontinued the therapy due to COVID-19 infection or COVID-19-related reasons, they will continue to receive trametinib plus anlotinib therapy if the investigators think the patients still can receive benefit from the medication".<br>5. Add integrative analyses: "For the patients enrolled in phase I clinical study (including part A and part B), the clinical characteristics, ORR, PFS, and AEs will be analyzed".<br>6. Add subgroup analyses: "According to the mutation types of non-G12C KRAS, the FAS will be divided into KRASG12V subtype, KRASG12D subtype, and KRASother subtype. ORR and PFS will be calculated based on the different KRAS mutation subtypes. In addition, tumor shrinkage (percent change from baseline) will be presented for different KRAS mutation subtypes".

Clinical approval No.: IS2117 (version 4.0)

Protocol No.: ATRAS-LC-4.0

Version No. and Date: 4.0/2023.05.09

Combined MEK Inhibitor Trametinib and RTK Inhibitor Anlotinib Therapy in non-G12C KRAS-Mutant Lung Cancer patients

Study Protocol

Sponsor: Shanghai Chest Hospital, Shanghai Jiao Tong University School of Medicine/ Chia Tai Tianqing Pharmaceutical Group Co, Ltd/ Novartis

Principal Investigator: Professor Baohui Han

Confidentiality statement

The information contained in this document, especially unpublished data, belongs to the sponsor of this study. Since you are treated as a researcher, potential researcher or consultant, it is provided to you as a confidential for you, your research team and independent ethics committee/institutional review committee. It must be clear that, except in the case of obtaining the informed consent of the patient who may take the drug, this information cannot be disclosed to others without the written authorization of the sponsor.

Content

Trial Protocol Synopsis ... 5
1. Introduction ... 17
1.1. Background ... 17
2. Study design ... 20
2.1. Overview ... 20
2.2. Part A: Dose Escalation ... 20
2.3. Part B: dose expansion ... 23
3. Study objectives ... 25
3.1. Part A ... 25
4. Subject selection ... 26
4.1. Inclusion criteria ... 26
4.2. Exclusion criteria ... 27
4.3. Enrollment of subjects ... 29
4.4. Procedures for handling wrongly enrolled subjects ... 29
4.5. Restrictions ... 29
5. Research treatment and execution ... 31
5.1. Treatment ... 31
5.1.1. Dose Escalation (Part A) ... 32
5.1.2. Dose Expansion (Part B) ... 33
5.1.3. Definition of dose-limiting toxicity ... 33
5.1.4. Safety Review Committee ... 34
5.1.5. Effectiveness evaluation ... 34
5.1.6. Toxicity management ... 35
5.1.7. Treatment time ... 38
5.1.8. Treatment compliance and inventory ... 38
6. Benefit/risk and ethical evaluation ... 39
6.1. Potential benefits ... 39
6.2. Potential risks ... 39
6.2.1. Gastrointestinal tract diseases ... 39
6.2.2. Skin diseases ... 40

6.2.3. Cardiovascular system 40
6.2.4. Respiratory system 41
6.2.5. Liver diseases 41
6.2.6. Thyroid 42
6.2.7. Hematopoietic function 42
6.2.8. Hyperlipidemia 42
6.2.9. Reproductive organs 43
6.2.10. CYP450 induction/inhibition 43
7. Discontinue study drugs and withdraw from the study 44
7.1. Procedure for patients to stop study drug 44
7.2. Procedures for patients who started using study drug incorrectly 45
7.3. Procedures for withdrawing from the study 45
8. Research plan and collection of research variables 46
8.1. The detailed steps of the study plan are shown in the table below 46
8.2. Enrollment/screening period 49
8.3. Treatment period 51
8.4. Safety follow-up period 52
8.5. Progressive follow-up period 52
8.6. Survival follow-up period 52
9. Research Evaluation 53
9.1. Effectiveness evaluation 53
9.2. Safety assessment 55
9.2.1. Laboratory safety assessment 55
9.2.2. Physical examination 56
9.2.3. Electrocardiogram 56
9.2.4. Echocardiography/MUGA Scan 57
9.2.5. Vital signs 57
10. Safety report and medical management 58
10.1. Definition of adverse events 58
10.2. Definition of serious adverse events 58
10.3. Adverse Event Record 59
10.3.1. Time period for collecting adverse events 59

10.3.2. Follow-up of unresolved adverse events 59
10.3.3. Variables 59
10.3.4. Causality collection 60
10.3.5. Adverse events based on signs and symptoms 60
10.3.6. Adverse events based on inspections and trials 60
10.3.7. Disease progression 61
10.4. Report of serious adverse events 61
11. Statistical methods 62
11.1. Definition of study endpoint 62
11.2. Calculation of sample size 63
11.3. Statistical analysis 63
12. Ethical considerations 67
References 67
Appendix material 70
Abbreviations 75

Trial Protocol Synopsis

Basic information of drug clinical trial:

- Clinical approval No.: IS2117 (version 4.0)
- Protocol No.: ATRAS-LC-4.0
- Version No. and Date: 4.0/2023.05.09
- Principal Investigator: Baohui Han
- Unit: Shanghai Chest Hospital, Shanghai Jiao Tong University School of Medicine
- Email: 18930858216@163.com

Trial title:

Combined MEK Inhibitor Trametinib and RTK Inhibitor Anlotinib Therapy in non-G12C KRAS-Mutant Lung Cancer patients

Study objectives:

Part A

Primary objective: To define the recommended phase 2 dose (RP2D) of the combined strategy of trametinib and anlotinib for advanced non-small cell lung cancer (NSCLC) patients with non-G12C KRAS mutation.

Secondary objective: To evaluate the progression-free survival (PFS), objective response rate (ORR), disease control rate (DCR) and safety of the combined strategy of trametinib and anlotinib for advanced NSCLC patients with non-G12C KRAS mutation.

Part B

Primary objective: To evaluate the ORR of the combined strategy of trametinib and anlotinib for advanced NSCLC patients with KRAS mutation (excluding $KRAS^{G12C}$) who previous had been received 1st or more lines standard therapy.

Secondary objective: To evaluate the PFS, overall survival (OS), DCR, duration of response (DoR) and safety of the combined strategy of trametinib and anlotinib for advanced NSCLC patients with non-G12C KRAS mutation who previous had been

received 1st or more lines standard therapy.

Trial design:

- This is a phase I, open-label, single-center study aimed at exploring the potential therapeutic efficacy of trametinib (oral) and anlotinib (oral) in the treatment of advanced NSCLC with KRAS mutations (excluding KRAS^{G12C}). The purpose of this study is to explore the best combined dosage under the premise of ensuring patient safety through close safety monitoring. This study is mainly divided into two parts: Part A, dose escalation; Part B, dose expansion. If the valuable ORR and RP2D are reached in part A, part B will be started; if RP2D is not reached in part A, part B will not be started. The therapeutic efficacy and companion diagnostics of the patients enrolled in part A can be reported as a case report or small cohort report before the completion of the clinical trial. The number of subjects is determined according to the actual situation of dose climbing.

Research phase	Timeline（Part A）	Timeline (Part B, if activated)
Estimated date of enrollment of the first subject	July 2021	March 2022
Estimated date of enrollment of the last subject	June 2022	December 2022
Estimated date of the last visit of the last subject	September 2023	February 2024

Part A: Dose escalation

The purpose of designing part A is to determine the RP2D of trametinib and anlotinib based on the evaluation of the ORR and safety data for further clinical evaluation. If the ORR and RP2D are reached, the results can be reported, and the part B will be performed. Here, we decide whether perform to explore the next dose evaluation based on the toxicity of first 28-day cycle administration. The 28-day evaluation period was chosen because it is expected that the major toxicity that caused the discontinued dose

in this type of phase I oncology studies (hematology, gastrointestinal tract, liver enzymes) will occur during this period. The dose exploration cohort adopts a "3 + 3" design. The therapeutic efficacy and companion diagnostics of the patients enrolled in part A can be reported as a case report or small cohort report before the completion of the clinical trial.

The two drugs involved in this study, anlotinib and trametinib, have been marketed, and the standard dosages and adverse reactions have been relatively clear. However, considering that the interaction mechanism of the two drugs has not yet been fully clarified, in order to fully ensure the safety of patients' medication and to explore effective and tolerable clinical combination doses for further clinical trials, we designed four dose gradients.

Cohort	Anlotinib Dose	Trametinib Dose
1	6mg, (d1-d14 per 21-day cycle) orally every day	2mg, orally every day
2	8mg, (d1-d14 per 21-day cycle) orally every day	2mg, orally every day
3	10mg, (d1-d14 per 21-day cycle) orally every day	2mg, orally every day
4	12mg, (d1-d14 per 21-day cycle) orally every day	2mg, orally every day

Based on the observed toxicity, incremental doses of anlotinib are gradually explored. For the determination of maximum tolerated dose (MTD), only the dose-limiting toxicity (DLT) of the first treatment cycle is evaluated. After reviewing the safety data for each dose level, the sponsor and the investigator will jointly determine the DLT.

For a climbing trial designed according to the "3+3" principle, if 0 of the 3 subjects in a given dose level cohort experience DLT, then a higher dose will be explored. If 1 out of 3 subjects develops DLT, the dose level group will recruit 3 more subjects, for a total of 6 subjects. If only 1 of these 6 subjects develops DLT, it is recommended to explore a higher dose, or stop the trial and choose this dose as the MTD (when the dose group is the highest dose). If more than 1 of the 6 subjects has DLT, it indicates that the exploration can be carried out in the lower dose group, or the entire trial can be stopped (when the dose group is the lowest dose group).

If among the 3 subjects enrolled in a given dose group, more than 1 subject with DLT

is observed, it is suggested that the exploration can be carried out in a lower dose group. If only 3 subjects are enrolled in the low-dose group, 3 additional subjects will be recruited. At this time, there will be a total of 6 subjects in this dose group for evaluation. Repeat the above steps to observe the number of subjects with DLT among the 6 subjects.

In order to confirm the MTD, 6 subjects at this dose level will be evaluated for DLT and intolerable toxicity in cycle 1 of treatment (cannot be controlled by dose interruption and/or reduction). The sponsor and investigators will review the safety and clinical data of all subjects to jointly determine the RP2D of anlotinib combined with trametinib.

If the DLT of 2 or more subjects is level 3, then the enrollment of the study will be stopped.

DLT is defined as the occurrence from the first dose of the study treatment (day 1, cycle 0) to the last day of cycle 1 (28 days after the start of dosing), and is not attributable to the disease under study or any disease-related toxicity. Any of the following events that occurred during the first cycle (the first 3 weeks), if it is possible, likely, or definitely related to the drug (according to the National Cancer Institute (NCI) Cancer Therapy Evaluation Program (CTEP) adverse event (AE) reporting requirements), will be considered as a dose-limiting toxicity:

Grade 4 blood toxicity or grade 3 neutropenia with fever ≥38.5°C;

Non-hematological toxicity of grade 3 or above;

Other toxicities greater than baseline, clinically significant and/or unacceptable, and are judged as DLT by the safety review committee.

Any other toxicity of the stopping criteria defined by the protocol (ie, interstitial lung disease or increased QTc with symptoms or signs of severe arrhythmia)

Any other toxicity that caused the interruption of the dosing schedule for more than 7 days.

DLT does not include:

Hair loss of any level

Any level of independent laboratory changes without clinical sequelae or clinical significance.

Therapeutic efficacy evaluation will be assessed according to Response Evaluation Criteria in Solid Tumors (RECIST) version 1.1 every 4-8 weeks. If the subject has benefited from the efficacy assessment, he/she will continue to receive the next treatment cycle until the progression disease (PD) or unacceptable toxicity occurs to further evaluate the safety, tolerability and efficacy. After the investigators' evaluation, the assessment cycle can extend to 12 weeks or longer due to the uncontrollable factors are also be accepted during the treatment period.

Part B: Dose expansion

If RP2D is reached in Part A, another 20 eligible patients will be enrolled and treated with trametinib (2mg QD, oral) + anlotinib (RP2D, once a day, from day 1 to day 14 of the 21-day cycle), until the PD or unacceptable toxicity occurs to further evaluate the safety, tolerability and efficacy. Therapeutic efficacy evaluation will be assessed according to RECIST version 1.1 every 4-8 weeks. After the investigators' evaluation, the assessment cycle can extend to 12 weeks or longer due to the uncontrollable factors are also be accepted during the treatment period.

For all eligible patients (Part A and Part B), blood samples will be collected at each evaluation. And if feasible, tissues at PD defined by RECIST will be collected to understand drug resistance. Part A and Part B can be reported respectively.

In Part A, in each dose cohort, the first 3 patients must undergo the first cycle observation period. The enrollment of the next dose group must be separated from the previous dose by one medication cycle.

If RP2D is not reached, part B will not be activated.

Figure 1. Trial flow diagram

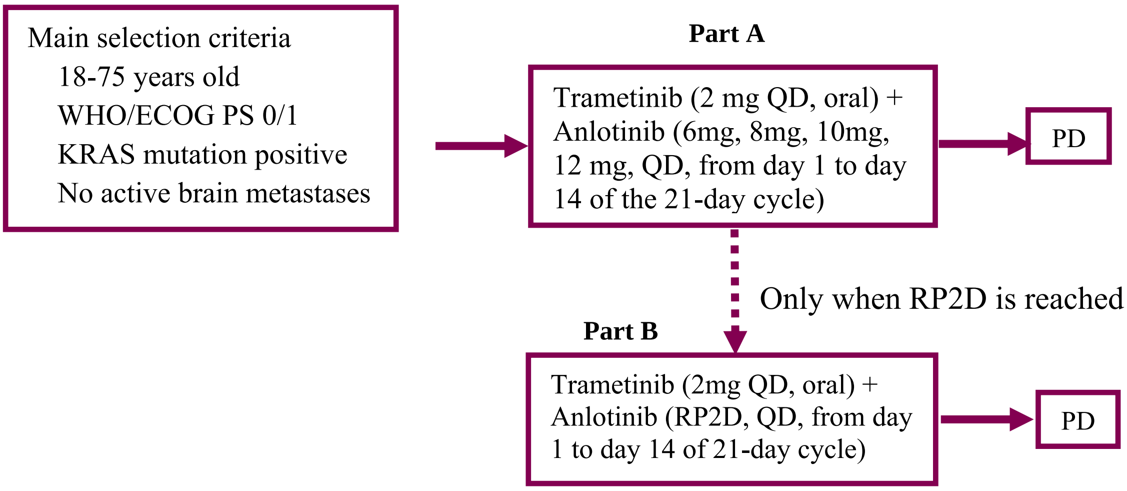

Inclusion criteria

According to the 8th edition of the American Journal of Critical Care (AJCC)/Union for International Cancer Control (UICC) Tumor Node Metastasis (TNM) NSCLC staging system, locally advanced (stage III B/III C), metastatic or recurrent (stage IV) NSCLC patients confirmed by histology or cytology. The NSCLC patient cannot receive surgery and radical radiotherapy, and at least one measurable lesion is confirmed according to RECIST 1.1.

KRAS mutation positive excluded $KRAS^{G12C}$ mutation

No active brain metastases

Age ≥18 years old and ≤75 years old

ECOG PS score: 0-1

Patients who had previously received at least 1st-line standard therapy. Note: the treatment naïve patients who refused immunotherapy/chemotherapy at 1st-line, and willingly enrolled in the clinical trial are also eligible.

Part A: advanced NSCLC patients with non-G12C mutations who have previously received standard treatment or treatment naïve

Part B: advanced NSCLC patients with non-G12C mutations who have previously

received standard 1st or more treatment

Palliative radiotherapy must be completed 7 days before the first dose of study drug is administered;

The main organs are functioning normally, that is, they meet the following standards:

Good hematopoietic function, defined as absolute neutrophil count $\geq 1.5\times10^9$/L, platelet count $\geq 100\times10^9$/L, hemoglobin ≥90 g/L [no blood transfusion or no erythropoietin (EPO) within 7 days before enrollment dependence]

The biochemical test results should meet the following standards: BIL <1.25 times the upper limit of normal (ULN); ALT and AST <2.5×ULN; if liver metastasis occurs, ALT and AST <5×ULN; Cr ≤1.5×ULN or creatinine clearance Rate (CCr) ≥60 ml/min; good coagulation function, International Normalized Ratio (INR) and PT ≤1.5 times of ULN; if the subject is receiving anticoagulation therapy, PT should be within the prescribed range of anticoagulation drugs;

Women of childbearing age should agree to take contraceptive measures (such as intrauterine devices, contraceptives, or condoms) during the study and within 6 months after the study; the serum or urine pregnancy test of non-breastfeeding patients should be negative; male patients should agree to take contraceptive measures during the study period and within 6 months after the study.

The patients voluntarily participated in the study, signed an informed consent form and had good compliance.

The expected survive time is longer than 3 months.

Exclusion criteria

Patients who meet any of the following criteria will be excluded:

Patients with active central nervous system metastasis are excluded. If a subject has received adequate treatment for central nervous system (CNS) metastasis at least 2 weeks before enrollment, and is neurologically restored to baseline levels (except for residual signs or symptoms related to CNS treatment), the subject is eligible. In addition, subjects must stop corticosteroids, or the daily dose of prednisone must be stabilized or

gradually reduced to ≤10 mg (or equivalent dose).

Small cell lung cancer (including mixed small cell and non-small cell lung cancer) or hollow central squamous cell carcinoma;

There are obvious bleeding symptoms

Patients who have previously received MEK inhibitors, anlotinib or other multi-targeted anti-angiogenic therapy;

Patients with dysphagia, gastrointestinal resection, chronic diarrhea, intestinal obstruction and other factors that affect oral medication;

Patients who are known to have active brain metastases, spinal cord compression, cancerous meningitis, brain or soft tissue diseases diagnosed by computed tomography (CT) or magnetic resonance imaging (MRI) at the time of screening;

Patients with severe and/or uncontrollable diseases, such as: unstable angina, symptomatic congestive heart failure, myocardial infarction within 6 months, severe uncontrollable arrhythmia; uncontrolled blood pressure (BP, constriction BP > 140 mmHg, diastolic BP > 90 mmHg);

Active or uncontrolled serious infections;

Liver diseases such as cirrhosis, decompensated liver disease, acute or chronic active hepatitis;

Incomplete control of eye inflammation or eye infection, or any condition that may cause the above eye diseases;

Poor diabetes control (fasting blood glucose (FBG) > 10 mmol/L);

Routine urine test results show that urine protein is ≥++, and the 24-hour urine protein quantitative is> 1.0 g;

Active tuberculosis;

Uncontrollable hypercalcemia (calcium ion> 1.5 mmol/L or calcium> 12 mg/dL or corrected serum calcium >ULN), or symptomatic hypercalcemia that requires continued bisphosphonate therapy;

Long-term unhealed wounds or fractures;

Those who have a history of psychotropic drug abuse and cannot be quit or have mental disorders;

Patients with known severe allergies (≥Grade 3) to active ingredients and excipients;

Patients who also suffer from other malignant tumors (except radical cervical carcinoma in situ, non-melanoma skin cancer, etc.); patients who have been assessed by the investigator as having concomitant diseases that seriously endanger the patient's safety or affect the patient's completion of the study;

During the clinical trial, the subjects or their sexual partners cannot or refuse to take effective contraceptive measures;

Pregnant or breastfeeding women; Previous treatment including Chinese medicine treatment; Patients who are allergic to any medicine or any ingredient; the patients with a history of treatments involving MEK inhibitors (trametinib, selumetinib, etc.) and RTKs inhibitors (anlotinib, sorafenib, apatinib, cabozantinib, etc.) were considered ineligible.

In other cases, patients who are not eligible for inclusion as assessed by the investigator.

The expected survive time is shorter than 3 months.

Study drugs, dosages, and methods of administration

Cohort	Anlotinib Dose	Trametinib Dose
1	6mg, (d1-d14 per 21-day cycle) orally every day	2mg, orally every day
2	8mg, (d1-d14 per 21-day cycle) orally every day	2mg, orally every day
3	10mg, (d1-d14 per 21-day cycle) orally every day	2mg, orally every day
4	12mg, (d1-d14 per 21-day cycle) orally every day	2mg, orally every day

Statistical methods

The primary endpoint of this study was to determine RP2D based on the incidence of DLT in cycle 1. The specific required sample size will be based on the dose-escalation design of the "3+3" principle, and the DLT situation of each dose group observed during the actual dose escalation process. For the dose expansion phase, it is planned to enroll 20 patients to further clarify the toxicity, tolerability, and efficacy.

All data will be provided for the full analysis set (FAS). All patients who received at least one dose of study drug will be included in the study analysis. For all variables,

descriptive statistics will be performed as appropriate. Continuous variables will be summarized by observations, mean, standard deviation, median, minimum, and maximum. Categorical variables will be aggregated by frequency counts and percentages for each category.

The FAS includes all patients who received at least one dose of study drug. The FAS will be used for safety analysis.

Evaluation indicators

- **Safety and tolerability**

The safety analysis set (SAS) will include all patients who have received at least one dose of the therapeutic drugs. The patient will be evaluated based on the actual treatment received. The safety and tolerability summary will be based on the safety analysis set. AEs will be coded according to the system organ category (SOC) and preferred terminology in MedDRA. The severity of AE will be graded according to NCI Common Terminology Criteria for Adverse Event (CTCAE) version 5.0. All AEs will be listed, including detailed information collected for each AE (description of event, date/time of onset, duration, severity, severity, relationship with study drug, measures taken, clinical outcome).

- **Tumor response**

Tumor response data will be listed and summarized by dose group, and the following response categories will be used when appropriate: complete response (CR), partial response (PR), stable disease (SD), PD, and non-evaluable (NE). For the definitions of CR, PR, SD and PD, please refer to the RECISIT version 1.1 standard. Where appropriate, the objective tumor response rate will be given with a 95% confidence interval (calculated using the Clopper-Pearson interval).

Objective response rate (ORR) is defined as the percent of patients documented to have a confirmed CR or PR.

Descriptive statistics (frequency and percentage) for ORR based on response assessments by investigator. Patients who cannot be assessed for response will be

counted as not evaluable. Descriptive statistics (frequency and percentage) for CR and PR rate will be presented overall.

- **Duration of response**

Duration of Response (DOR) in months is defined as the time from date of the first documentation of objective response (CR or PR) to the first documentation of PD or to death due to any cause in the absence of documented PD. DOR will only be calculated for the subgroup of patients achieving an efficacy of CR or PR. The response duration of the responding patients will be summarized, and the number of responding patients (%) for the duration of remission> 3; > 6; > 9; > 12 months will be given. The Kaplan Meier chart and the median duration of response with 95% confidence interval (95% CI, calculated based on the Kaplan Meier chart) will be given. DOR will be evaluated based on response assessments by the investigator.

For the patients who discontinued the therapy due to COVID-19 infection or COVID-19-related reasons, they will continue to receive trametinib plus anlotinib therapy if the investigators think the patients still can receive benefit from the medication.

- **Progression-free survival**

PFS is defined as the time from the date of first treatment to the date of first PD or death due to any cause in the absence of documented PD, whichever occurs first. PFS (in days or months) will be calculated.

The PFS of the part A, part B, overall, subgroups will be summarized. PFS will be displayed using Kaplan-Meier curves.

PFS will be displayed using Kaplan-Meier curves. The number of events, the median (calculated based on the Kaplan-Meier curve), and the proportion of patients with no events at 6, 12, and 18 months will be summarized.

Where appropriate, a summary of the number and percentage of patients who died, are still under follow-up, lost to follow-up, and withdrawn from the study will be provided.

For the patients who discontinued the therapy due to COVID-19 infection or COVID-19-related reasons, they will continue to receive trametinib plus anlotinib therapy if the investigators think the patients still can receive benefit from the medication.

- **Integrative analyses**

For the patients enrolled in phase I clinical study (including part A and part B), the clinical characteristics, ORR, PFS, and AEs will be analyzed.

- **Subgroup analyses**

According to the mutation types of non-G12C KRAS, the FAS will be divided into $KRAS^{G12V}$ subtype, $KRAS^{G12D}$ subtype, and $KRAS^{other}$ subtype. ORR and PFS will be calculated based on the different KRAS mutation subtypes. In addition, tumor shrinkage (percent change from baseline) will be presented for different KRAS mutation subtypes.

- Biomarkers and mutation profiles

All patients participating in the biomarker study will be included in the analysis set.

Ethics

The study protocol (including informed consent form) shall be submitted to the Ethics Committee for approval, and all subjects entering trial screening must sign an informed consent form. The investigator should carry out the trial in strict accordance with the study protocol and Good Clinical Practice (GCP) requirements, and fully guarantee the legitimate rights and interests and safety of subjects.

1. Introduction

1.1. Background

Lung cancer is the most common cause of cancer-related death worldwide. Approximately 85% to 90% of lung cancer cases are NSCLC, of which KRAS is one of the most common driver genes, occurring in 25-30% of lung adenocarcinomas and 3-5% of squamous cell carcinomas(*1, 2*). The most common KRAS mutations mainly occur at codons 12, 13, and 61, but currently only G12C site has an accessible specific allosteric inhibitor, and G12C mutation accounts for 2.8~13% of NSCLC patients (*3*). Other common mutation sites such as G12V, G12D, Q61H, etc., there is no effective targeted strategy (*4*). The second-line classic docetaxel chemotherapy regimen for the treatment of patients with KRAS mutant NSCLC has a median PFS of less than 3 months and an ORR of only about 10% (*5*).

MEK1/2 is the most critical and the most intensively studied KRAS downstream molecule, and phase II and phase III clinical trials have been carried out. However, the efficacy of MEK inhibitors alone and the maintenance time are limited. With the popularity of combined anti-tumor strategies, a variety of MEK inhibitors combined with other treatment methods have been developed (for example, PI3K/AKT/mTOR inhibitors, FGFR1 inhibitors, IGF1R inhibitors, chemotherapy, etc.)(*6, 7*), which indicates another possible option for future clinical trials, but the existing combination strategy is limited by toxicity or poor efficacy (*8*) (*9*)。

Therefore, we aim to develop a new combination therapy containing MEK inhibitors to treat KRAS-mutant (excluded $KRAS^{G12C}$) NSCLC patients, and to cover different mutation subtypes as much as possible to form a targeted treatment regimen that is effective for KRAS mutations. Although MEK inhibitors are initially effective against KRAS mutant lung cancer cell lines and patients, relapses will inevitably occur in the short term, mainly due to the induction of multiple RTK genes or ligands. A large number of previous literatures have described the mechanism of MEK inhibitor

resistance in different types of KRAS mutant tumors, involving the upregulation of FGFR1 (*6*), and ERBB in lung cancer; the upregulation of EGFR in colon cancer; and the upregulation of AXL, DDR1, FGFR2, IGF1R, KIT, PDGFRB, VEGFRB, etc. in triple-negative breast cancer (*10*). The diversity of adaptive resistance mechanisms suggests that combined therapy of MEK inhibitors and a single RTK inhibitor is impractical. However, effectively blocking signals from multiple RTK activations may be a viable strategy to prevent drug resistance.

At present, a variety of anti-angiogenic multi-target small molecule tyrosine kinase inhibitors (TKIs) have been approved for the treatment of tumors. We select drugs that cover the above targets (anlotinib) and MEK inhibitors (trametinib) and plan to explore whether the combination of the two drugs can further inhibit the growth of NSCLC. Anlotinib suppresses tumor growth by inhibiting signal pathways involved in angiogenesis (VEGFR1-3, PDGFα-β, FGFR1-4) and cell proliferation (c-KIT, Ret, Aurora-B, c-FMS, DDR1) (*11*). Currently, anlotinib is approved in China for the third-line and later treatment of advanced NSCLC patients, small cell lung cancer, medullary thyroid carcinoma and soft tissue sarcoma (*12-14*). We analyzed the results of the KRAS mutant subgroup in the Phase III clinical trial (ALTER0303) of anlotinib for the treatment of advanced NSCLC in and after the third line. A total of 29 patients carried KRAS mutations, of which 8 cases achieved curative effect of PR, 13 cases were SD, the ORR rate reached 27.6%. The survival time analysis showed that the median PFS of anlotinib single-agent in the posterior line treatment was 4.23 months, and the median OS time was 8.00 months.

Based on the above evidence, we infer that the combined use of anlotinib and trametinib may form an effective strategy for the treatment of KRAS-mutant (excluded $KRAS^{G12C}$) NSCLC patients. We explored *in vitro* cell lines and mouse xenograft models whether the combination of the two drugs can bring better efficacy and whether the toxicity can be tolerated. The results of preclinical data showed that the two drugs showed strong synergistic anti-tumor effects in a variety of NSCLC cell lines carrying different KRAS mutations (G12C, G12D, G12V, G12S, Q61H), and this result was also

validated in xenograft tumor models. There were no significant differences in the weight, mental state and behavior patterns of mice in the control group, single-drug group, and combination-drug group during the entire observation period, suggesting that the combined application of the two drugs did not cause intolerable toxicity. We intend to further explore whether anlotinib combined with trametinib can bring survival benefits to KRAS-mutant (excluded $KRAS^{G12C}$) NSCLC patients and its possibility as a treatment strategy.

2. Study design

2.1. Overview

This is a phase I, open-label, single-center study aimed at exploring the potential therapeutic efficacy of trametinib (oral) and anlotinib (oral) in the treatment of advanced NSCLC patients with KRAS mutations (excluded KRAS^{G12C}). The purpose of this study is to explore the best combined dosage under the premise of ensuring patient safety through close safety monitoring. This study is mainly divided into two parts: Part A, dose escalation; Part B, dose expansion. If RP2D is reached in part A, part B will be started; if RP2D is not reached in part A, part B will not be started. The number of subjects is determined according to the actual situation of dose climbing.

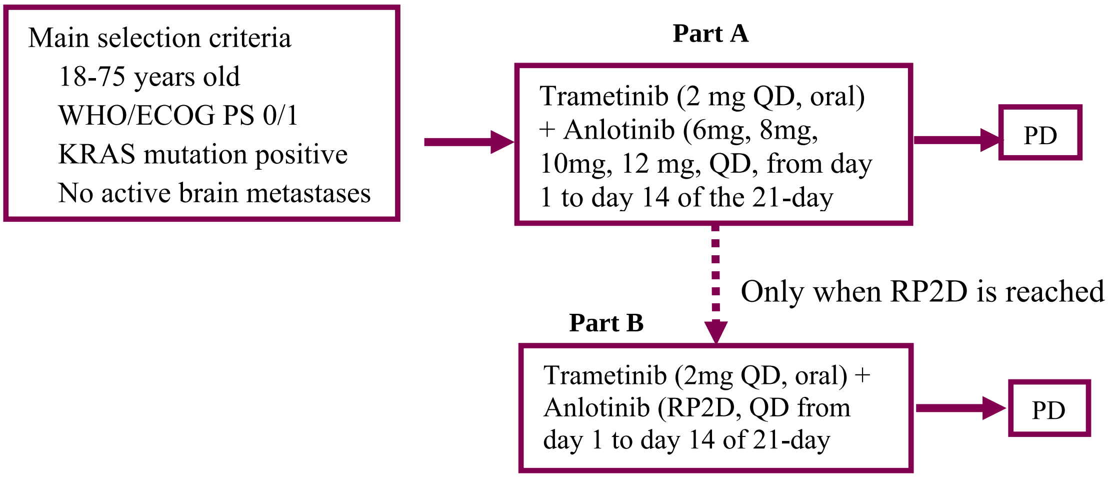

Figure 1. Research flow diagram

In Part A, in each dose cohort, the first 3 patients must undergo the first cycle observation period. The enrollment of the next dose group must be separated from the previous dose by one medication cycle.

If RP2D is not reached, part B will not be activated.

2.2. Part A: Dose Escalation

The purpose of designing part A is to determine the RP2D of trametinib and anlotinib

based on the evaluation of the ORR and safety data for further clinical evaluation. If the ORR and RP2D are reached, the results can be reported, and the part B will be performed. Here, we decide whether perform to explore the next dose evaluation based on the toxicity of first 28-day cycle administration. The 28-day evaluation period was chosen because it is expected that the major toxicity that caused the discontinued dose in this type of phase I oncology studies (hematology, gastrointestinal tract, liver enzymes) will occur during this period. The dose exploration cohort adopts a "3 + 3" design. The therapeutic efficacy and companion diagnostics of the patients enrolled in part A can be reported as a case report or small cohort report before the completion of the clinical trial.

The two drugs involved in this study, anlotinib and trametinib, have been marketed, and the standard dosages and adverse reactions have been relatively clear. However, considering that the interaction mechanism of the two drugs has not yet been fully clarified, in order to fully ensure the safety of patients' medication and to explore effective and tolerable clinical combination doses for further clinical trials, we designed four dose gradients.

Cohort	Anlotinib Dose	Trametinib Dose
1	6mg, (d1-d14 per 21-day cycle) orally every day	2mg, orally every day
2	8mg, (d1-d14 per 21-day cycle) orally every day	2mg, orally every day
3	10mg, (d1-d14 per 21-day cycle) orally every day	2mg, orally every day
4	12mg, (d1-d14 per 21-day cycle) orally every day	2mg, orally every day

Based on the observed toxicity, incremental doses of anlotinib are gradually explored. For the determination of MTD, only the DLT of the first treatment cycle is evaluated. After reviewing the safety data for each dose level, the sponsor and the investigator will jointly determine the DLT.

For a climbing trial designed according to the "3+3" principle, if 0 of the 3 subjects in a given dose level cohort experience DLT, then a higher dose will be explored. If 1 out of 3 subjects develops DLT, the dose level group will recruit 3 more subjects, for a total of 6 subjects. If only 1 of these 6 subjects develops DLT, it is recommended to explore

a higher dose, or stop the trial and choose this dose as the MTD (when the dose group is the highest dose). If more than 1 of the 6 subjects has DLT, it indicates that the exploration can be carried out in the lower dose group, or the entire trial can be stopped (when the dose group is the lowest dose group).

If among the 3 subjects enrolled in a given dose group, more than 1 subject with DLT is observed, it is suggested that the exploration can be carried out in a lower dose group. If only 3 subjects are enrolled in the low-dose group, 3 additional subjects will be recruited. At this time, there will be a total of 6 subjects in this dose group for evaluation. Repeat the above steps to observe the number of subjects with DLT among the 6 subjects.

In order to confirm the MTD, 6 evaluable subjects at this dose level will be evaluated for DLT and intolerable toxicity in cycle 1 of treatment (cannot be controlled by dose interruption and/or reduction). The sponsor and investigators will review the safety and clinical data of all subjects to jointly determine the RP2D of anlotinib combined with trametinib.

If the DLT of 2 or more subjects is level 3, then the enrollment of the study will be stopped.

DLT is defined as the occurrence from the first dose of the study treatment (day 1, cycle 0) to the last day of cycle 1 (28 days after the start of dosing), and is not attributable to the disease under study or any disease-related toxicity. Any of the following events that occurred during the first cycle (the first 3 weeks), if it is possible, likely, or definitely related to the drug (according to the NCI CTEP AE reporting requirements), will be considered as a dose-limiting toxicity:

Grade 4 blood toxicity or grade 3 neutropenia with fever ≥38.5°C;

Non-hematological toxicity of grade 3 or above;

Other toxicities greater than baseline, clinically significant and/or unacceptable, and are judged as DLT by the safety review committee.

Any other toxicity of the stopping criteria defined by the protocol (i.e., interstitial lung disease or increased QTc with symptoms or signs of severe arrhythmia)

Any other toxicity that caused the interruption of the dosing schedule for more than 7 days.

DLT does not include:

Hair loss of any level

Any level of independent laboratory changes without clinical sequelae or clinical significance.

Multiple blood samples will be collected on the first day of the first cycle and the second cycle before dosing and 0.5, 1, 2, 4, 6, 8 and 24 hours after dosing. The investigator will collect 6ml of venous blood at each time point, separate the plasma and store it at -80°C until analysis. The validated high-performance liquid chromatography-tandem mass spectrometry (HPLC-MS/MS) method was used for analysis to understand the plasma concentration-time curves of the two drugs on the first day and steady state of each dose.

Tumor response will be assessed according to RECIST version 1.1 every 4-8 weeks. If the subject has benefited from the efficacy assessment, he/she will continue to receive the next treatment cycle until the PD or unacceptable toxicity occurs to further evaluate the safety, tolerability and efficacy. After the investigators' evaluation, the assessment cycle can extend to 12 weeks or longer due to the uncontrollable factors are also be accepted during the treatment period.

2.3. Part B: dose expansion

If RP2D is reached in Part A, another 20 eligible patients will be enrolled and treated with trametinib (2mg QD, oral) + anlotinib (RP2D, once a day, from day 1 to day 14 of the 21-day cycle), until the PD or unacceptable toxicity occurs to further evaluate the safety, tolerability and efficacy. Therapeutic efficacy evaluation will be assessed according to RECIST version 1.1 every 4-8 weeks. After the investigators' evaluation, the assessment cycle can extend to 12 weeks or longer due to the uncontrollable factors are also be accepted during the treatment period.

For all eligible patients (Part A and Part B), blood samples will be collected at each

evaluation. And if feasible, tissues at PD defined by RECIST will be collected to understand drug resistance. Part A and Part B can be reported respectively.

Accurate identification of genetic changes after progression is not only critical for patient management, but also for advancing the understanding of treatment-induced tumor evolution. Although repeated biopsy is advertised as a tool for monitoring tumor evolution, this method may be too cumbersome in the real world to be applied to every patient. Obtaining a tissue biopsy for molecular analysis can be challenging. In up to 20% of patients, there is a significant risk of bleeding, infection, and other complications. For up to 49% of patients, doctors cannot perform a biopsy for molecular analysis due to comorbidities or insufficient tumor tissue. Therefore, there is an urgent need for a non-invasive liquid biopsy method to assess tumor mutations and achieve simple repeated detection throughout the treatment process. The isolation and subsequent molecular analysis of non-cellular circulating tumor DNA (ctDNA) in plasma is a powerful tool that can help improve the clinical outcome of many cancer types, including NSCLC. At the same time, by collecting a variety of secreted proteins in plasma, proteomics chips can be used to further explore the biomarkers in response to dual-drug combination therapy and the internal mechanism of dual-drug synergy.

In this study, based on plasma proteomics and ctDNA changes, the biomarkers related to the efficacy of dual-drug combination therapy and the internal biological mechanism of dual-drug synergistic anti-tumor will be explored.

3. Study objectives

3.1. Part A

3.1.1. Main purpose

To evaluate define the RP2D of the combined strategy of trametinib and anlotinib for advanced NSCLC patients with KRAS mutation (excluded $KRAS^{G12C}$).

3.1.2. Secondary purpose

To evaluate the ORR, PFS, DCR and safety of the combined strategy of trametinib and anlotinib for advanced NSCLC patients with KRAS mutation (excluded $KRAS^{G12C}$).

3.2. Part B

3.2.1. Main purpose

To evaluate the ORR of the combined strategy of trametinib and anlotinib for advanced NSCLC patients with KRAS mutation (excluded $KRAS^{G12C}$).

3.2.2. Secondary purpose

To evaluate the PFS, OS, DCR, DoR and safety of the combined strategy of trametinib and anlotinib for advanced NSCLC patients with KRAS mutation (excluded $KRAS^{G12C}$).

3.3. Exploratory purpose

To explore biomarkers related to the efficacy and resistance of dual-drug combination therapy and track changes in mutation profiles.

4. Subject selection

Each subject should meet all the inclusion criteria of this study and not meet any exclusion criteria. Under no circumstances can this rule be an exception.

4.1. Inclusion criteria

According to the 8th edition of the AJCC/UICC TNM NSCLC staging system, locally advanced (stage III B/III C), metastatic or recurrent (stage IV) NSCLC confirmed by histology or cytology. The patient cannot receive surgery and radical radiotherapy, and at least one measurable lesion is confirmed according to RECIST 1.1.

KRAS mutation positive

No active brain metastases

Age ≥18 years old and ≤75 years old

ECOG PS score: 0-1

Part A: advanced NSCLC patients with non-G12C mutations who have previously received standard treatment or treatment naïve (Note: the treatment naïve patients who refused immunotherapy/chemotherapy at 1st-line, and willingly enrolled in the clinical trial are also eligible)

Part B: advanced NSCLC patients with non-G12C mutations who have previously received standard 1st or more treatment

Palliative radiotherapy must be completed 7 days before the first dose of study drug is administered

The main organs are functioning normally, that is, they meet the following standards:

Good hematopoietic function, defined as absolute neutrophil count ≥1.5×10^9/L, platelet count ≥100×10^9/L, hemoglobin ≥90 g/L [no blood transfusion or no EPO within 7 days before enrollment dependence]

The biochemical test results should meet the following standards: BIL <1.25 times the ULN; ALT and AST <2.5×ULN; if liver metastasis occurs, ALT and AST<5×ULN; Cr≤1.5×ULN or CCr ≥60 ml/min; good coagulation function, INR and PT≤1.5 times

of ULN; if the subject is receiving anticoagulation therapy, PT should be within the prescribed range of anticoagulation drugs

Women of childbearing age should agree to take contraceptive measures (such as intrauterine devices, contraceptives, or condoms) during the study and within 6 months after the study; the serum or urine pregnancy test of non-breastfeeding patients should be negative; male patients should agree to take contraceptive measures during the study period and within 6 months after the study

The patients voluntarily participated in the study, signed an informed consent and had good compliance

The expected survive time is longer than 3 months

4.2. Exclusion criteria

Patients who meet any of the following criteria will be excluded:

Patients with active central nervous system metastasis are excluded. If a subject has received adequate treatment for CNS metastasis at least 2 weeks before enrollment, and is neurologically restored to baseline levels (except for residual signs or symptoms related to CNS treatment), the subject is eligible. In addition, subjects must stop corticosteroids, or the daily dose of prednisone must be stabilized or gradually reduced to ≤10 mg (or equivalent dose).

Small cell lung cancer (including mixed small cell and non-small cell lung cancer) or hollow central squamous cell carcinoma

There are obvious bleeding symptoms

Patients who have previously received MEK inhibitors, anlotinib or other anti-angiogenic therapy

Patients with dysphagia, gastrointestinal resection, chronic diarrhea, intestinal obstruction and other factors that affect oral medication

Patients who are known to have active brain metastases, spinal cord compression, cancerous meningitis, brain or soft tissue diseases diagnosed by CT or MRI at the time of screening

Patients with severe and/or uncontrollable diseases, such as: unstable angina, symptomatic congestive heart failure, myocardial infarction within 6 months, severe uncontrollable arrhythmia; uncontrolled BP (constriction BP > 140 mmHg, diastolic BP > 90 mmHg)

Active or uncontrolled serious infections

Liver diseases such as cirrhosis, decompensated liver disease, acute or chronic active hepatitis

Incomplete control of eye inflammation or eye infection, or any condition that may cause the above eye diseases

Poor diabetes control (FBG >10 mmol/L)

Routine urine test results show that urine protein is ≥++, and the 24-hour urine protein quantitative is> 1.0 g

Active tuberculosis

Uncontrollable hypercalcemia (calcium ion> 1.5 mmol/L or calcium> 12 mg/dL or corrected serum calcium> ULN), or symptomatic hypercalcemia that requires continued bisphosphonate therapy

Long-term unhealed wounds or fractures

Those who have a history of psychotropic drug abuse and cannot be quit or have mental disorders

Patients with known severe allergies (≥Grade 3) to active ingredients and excipients;

Patients who also suffer from other malignant tumors (except radical cervical carcinoma in situ, non-melanoma skin cancer, etc.); patients who have been assessed by the investigator as having concomitant diseases that seriously endanger the patient's safety or affect the patient's completion of the study;

During the clinical trial, the subjects or their sexual partners cannot or refuse to take effective contraceptive measures;

Pregnant or breastfeeding women; Previous treatment including Chinese medicine treatment; Patients who are allergic to any medicine or any ingredient; the patients with a history of treatments involving MEK inhibitors (trametinib, selumetinib, etc.) and RTKs inhibitors (anlotinib, sorafenib, apatinib, cabozantinib, etc.) were

considered ineligible.

In other cases, patients who are not eligible for inclusion as assessed by the investigator.

The expected survive time is shorter than 3 months.

4.3. Enrollment of subjects

All subjects considered for inclusion in the study must sign an informed consent form within 28 days before the start of treatment and before any study-specific screening procedures. The baseline assessment must be performed within 28 days before the start of study treatment, with the exception of pregnancy tests (if applicable). For serum samples, a pregnancy test needs to be performed within 72 hours before the first day of the first cycle. Before study treatment, it is necessary to confirm that the pregnancy test result is negative. The laboratory assessment and accompanying research blood sampling can be carried out within 72 hours before the 1st day of any cycle, and the tumor/imaging assessment can be carried out within 7 days, so that the investigator can obtain the results of the study during the visit.

4.4. Procedures for handling wrongly enrolled subjects

In any case, subjects who do not meet the eligibility criteria should not be included in or receive study medication. There can be no exceptions to this rule. Subjects who have been enrolled but subsequently found to not meet all eligibility criteria shall not start treatment and must withdraw from the study.

If the subject does not meet all eligibility criteria but starts treatment by mistake, the investigator will immediately notify the sponsor, and the sponsor and investigator should discuss whether to continue or terminate the patient's treatment. The sponsor must ensure that all decisions are properly documented.

4.5. Restrictions

The following restrictions apply to patients during the study drug treatment and specific

times before and after treatment:

(1) Women with reproductive potential must use reliable contraceptive methods from the beginning of screening to 6 months after the drug is stopped. When in line with the subject's preferred and general lifestyle, acceptable contraceptive methods include true abstinence. [Periodic abstinence (for example, calendar, ovulation, symptoms, post-ovulation method), the announcement and withdrawal of abstinence during the trial period are unacceptable contraceptive methods], hormonal contraceptives that are not prone to drug interactions (for example, intrauterine birth control system [IUS] levonorgestrel intrauterine system, medroxyprogesterone injection), copper belt intrauterine device and partner undergoing vasectomy. All hormonal contraceptive methods should be used with condoms and used by male sexual partners during intercourse.

(2) During the trial period (6 months), male patients must be required to use isolation contraceptives (that is, use condoms) during sex with all female partners. The patient should not give birth within 6 months after completing the medication. Patients must avoid donating sperm from the start of administration until 6 months after the discontinuation of study drug treatment. If male patients wish to give birth, they should be advised to arrange for a frozen sperm sample before starting study medication.

(3) If it is medically feasible, the patient should maintain treatment with conventional drugs other than the potent inducer of CYP3A4 (see Appendix B) throughout the study period (90 days after the last dose). If the patient is taking a drug that relies on blood C reactive protein (BCRP) and/or P-glycoprotein (Pgp) for distribution and has a narrow therapeutic index, it should be closely monitored in order to detect the increased exposure and changes in tolerance of the combination. If the patient is taking a drug that relies on CYP3A4, CYP1A2, CYP2C or p-glycoprotein for distribution and has a narrow therapeutic index, the decrease in study drug activity due to the reduced exposure of the combined drug should be closely monitored. Guidance is provided on medications to avoid, medications that require close monitoring, and washout periods (see Appendix B).

5. Research treatment and execution

5.1. Treatment

It is planned to take the combined doses of trametinib (2mg, QD) and anlotinib (6mg, 8mg, 10mg, 12mg, QD, the first day to the 14th day of the 21-day cycle) respectively. Novartis provides trametinib, and Chia Tai Tianqing Pharmaceutical will provide anlotinib. All study drugs should be kept in a safe place under proper storage conditions. The study drug label on the package specifies proper storage conditions. The label will be produced in accordance with Good Manufacturing Practices (GMP) and Chinese regulatory guidelines. The label text will be translated into Chinese. The investigator's handbook will provide more detailed information.

All eligible patients can obtain anlotinib combined with trametinib treatment by participating in this clinical study. Treatment will continue until RECIST-defined disease progression, unacceptable toxic effects, withdrawal from the study, or death occurs. For patients with RECIST-defined progression, if the patient continues to show clinical benefit and there is no discontinuation standard, the investigator can decide whether to continue the study treatment.

Figure 1. Research flow diagram

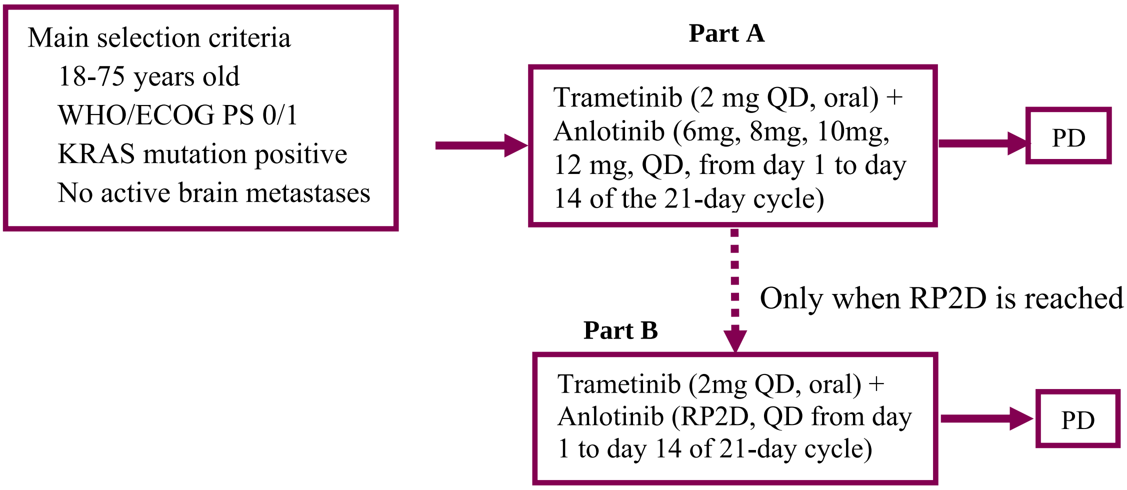

In Part A, in each dose cohort, the first 3 patients must undergo the first cycle observation period. The enrollment of the next dose group must be separated from the

previous dose by one medication cycle.

If RP2D is not reached, part B will not be activated.

5.1.1. Dose Escalation (Part A)

The two drugs involved in this study, anlotinib and trametinib, have been marketed, and their standard dosages and adverse reactions have been relatively clear. However, considering that the interaction mechanism of the two drugs has not yet been fully clarified, in order to fully guarantee the safety of patients' medication and to explore effective and tolerable clinical combination doses for further clinical trials, we designed four doses gradient.

Cohort	Anlotinib Dose	Trametinib Dose
1	6mg, (d1-d14 per 21-day cycle) orally every day	2mg, orally every day
2	8mg, (d1-d14 per 21-day cycle) orally every day	2mg, orally every day
3	10mg, (d1-d14 per 21-day cycle) orally every day	2mg, orally every day
4	12mg, (d1-d14 per 21-day cycle) orally every day	2mg, orally every day

Based on the observed toxicity, incremental doses of anlotinib are gradually explored. For the determination of MTD, only the DLT of the first treatment cycle is evaluated. After reviewing the safety data for each dose level, the sponsor and the investigator will jointly determine the DLT.

For a climbing trial designed according to the "3+3" principle, if 0 of the 3 subjects in a given dose level cohort experience DLT, then a higher dose will be explored. If 1 out of 3 subjects develops DLT, the dose level group will recruit 3 more subjects, for a total of 6 subjects. If only 1 of these 6 subjects develops DLT, it is recommended to explore a higher dose. If more than 1 of the 6 subjects has DLT, it indicates that the exploration can be carried out in the lower dose group, which as MTD. If we can not find more than 1 of the 6 subjects has DLT in Level 3, We will not continue to next dose level.

After part A, the investigators will review the safety and clinical data of all subjects to jointly determine the RP2D of anlotinib combined with trametinib.

5.1.2. Dose Expansion (Part B)

If RP2D is reached in Part A, another 20 eligible patients will be included and receive trametinib (2mg QD, oral) + anlotinib (RP2D, once a day, from day 1 to day 14 of the 21-day cycle), until the disease progression, withdrawal of informed consent, or unacceptable toxicity occurs to further evaluate the safety, tolerability and efficacy.

In the course of the study, once a patient has an AE of CTCAE grade 3 or above, and can recover to grade 1 or below within 2 weeks, the medication can be continued, and the subsequent medication dose will be lowered by one dose based on the original planned medication dose, otherwise the study will be withdrawn. Patients who continue to take the drug after the dose reduction reoccurs CTCAE grade 3 and above AEs during the follow-up process, and the AEs can return to grade 1 or below within 2 weeks, and they can continue to use this drug. The subsequent dose will be reduced by one dose based on the original planned dose, otherwise the study will be withdrawn.

5.1.3. Definition of dose-limiting toxicity

DLT is defined as the occurrence from the first dose of the study treatment (day 1, cycle 0) to the last day of cycle 1 (28 days after the start of dosing), and is not attributable to the disease under study or any disease-related toxicity. Any of the following events that occurred during the first cycle (the first 4 weeks), if it is possible, likely, or definitely related to the drug (according to the NCI CTEP AE reporting requirements), will be considered as a dose-limiting toxicity:

Grade 4 blood toxicity or grade 3 neutropenia with fever ≥38.5°C;

Non-hematological toxicity of grade 3 or above;

Other toxicities greater than baseline, clinically significant and/or unacceptable, and are judged as DLT by the safety review committee.

Any other toxicity of the stopping criteria defined by the protocol (ie, interstitial lung disease or increased QTc with symptoms or signs of severe arrhythmia))

Any other toxicity that caused the interruption of the dosing schedule for more than

7 days.

DLT does not include:

Hair loss of any level

Any level of independent laboratory changes without clinical sequelae or clinical significance.

However, scientific review committee (SRC) will take into account the incidence and type of DLT-type toxicity in cycle 2 and beyond when determining the dose escalation step.

5.1.4. Safety Review Committee

SRC is responsible for evaluating the safety and tolerability of the combination of trametinib and anlotinib, and deciding the next step to ensure patient safety. The SRC will be composed of principal investigators, who will chair the committee and be responsible for routine SRC meetings.

The first SRC meeting will be held after the first patient completes the first cycle of combined therapy. After that, the SRC meeting will be held after the completion of the first cycle of each of the first 3 patients (if necessary) in each dose cohort, and every 2 after 6 months or after the first 3 patients are enrolled. Held once a month.

The decision will include, but is not limited to, stopping further inclusion to ensure patient safety, reducing the dose of combination therapy, and re-evaluating the principles of AE management and dose adjustment to reduce risks.

5.1.5. Effectiveness evaluation

According to the RECIST 1.1 standard, all subjects will be monitored by radiological evaluation every 4-8 weeks to determine changes in tumor size. After the investigators' evaluation, the assessment cycle can extend to 12 weeks or longer due to the uncontrollable factors are also be accepted during the treatment period. For OS, every effort will be made to collect survival data for all subjects, including subjects who withdrew from treatment for any reason, are eligible to participate in the study and have

not revoked their consent for survival data collection. If the death of the subject is not reported, all dates in this study representing the date of contact of the subject will be used to determine the subject's last known survival date.

5.1.6. Toxicity management

Dose reductions, dose delays, and dose interruptions are permitted. If a patient has CTCAE grade 3 or higher and/or unacceptable toxicity (any grade), and the investigator believes that the event of concern is particularly related to the study drug (and includes the disease or disease-related process that is not related to the treatment of the patient DLT), the administration will be interrupted, and supportive treatment will be carried out in accordance with the guidelines.

If the toxicity subsides or returns to CTCAE ≤ 2 within 2 weeks after the onset, the study drug treatment can be restarted at the same or lower dose according to the investigator's assessment and according to the dose adjustment rules (Table 3). If the administration is restarted at the same dose level, the patient should be closely monitored 3 days after restarting treatment. If recurrence or worsening of the same toxicity occurs within 3 days, the investigator should consider reducing the dose as appropriate. Once the dose is reduced, the dose of the study drug cannot be restored to the high dose.

If the toxicity has not been relieved to CTCAE ≤ 2 after 2 weeks, the patient must withdraw from the study treatment and continue to observe the patient until the toxicity subsides.

If the dose needs to be interrupted after an AE, the investigator can restart the study drug at the same dose or a reduced dose according to the resolution/improvement of the event. Toxicity subsided within 14 days:

If the same AE recurs and the dose needs to be interrupted later, the study drug must be restarted with a lower dose after the AE improves.

If a different AE occurs and then the dose needs to be interrupted, according to the judgment of the investigator, after the AE is improved, the study drug can be re-

administered at the same dose or at a lower dose level.

According to the judgment of the investigator, the lowest possible dose can be allowed, the dose has been reduced to the lowest dose in the past, and the patient who shows an acceptable response to the interruption of the dose can be restarted with the lowest dose. Based on previous safety data for anlotinib and trametinib, patients were allowed to discontinue one of the drugs and continue with the remaining drug as monotherapy in the event of specific toxicities. And according to the degree of adverse reactions and remission, it is decided whether to delay, adjust or stop the dose of the drug in the follow-up. Adjustment to monotherapy is acceptable due to toxicity considerations. According to the investigator's judgment, for adverse reactions that may be caused by both drugs or some abnormal lipid metabolism, abnormal renal function and bleeding symptoms specific to anlotinib, the dose of anlotinib should be adjusted. If unacceptable toxicity persists after the lowest dose of anlotinib, trametinib monotherapy should be considered. For adverse reactions considered by the investigator to be particularly related to trametinib, the treatment of trametinib should be suspended, and if the use of trametinib cannot be resumed after supportive treatment, anlotinib monotherapy should be used.

a) Lung symptoms

If new or worsening pulmonary symptoms (such as dyspnea) or radiological abnormalities suggesting interstitial lung disease are observed, it is recommended to interrupt the study treatment dose and notify the sponsor research team. The expert group will discuss to rule out other causes such as lymphatic cancer, infection, allergies, cardiogenic edema or pulmonary hemorrhage. Case report form (CRF) will collect the results of the expert panel (including high-resolution computed tomography (HRCT), blood and sputum culture, hematology parameters). If there is a confirmatory HRCT scan, but other causes of respiratory symptoms are excluded, the diagnosis of interstitial lung disease should be considered and study treatment should be permanently terminated.

b) QTc interval prolongation

In view of the possibility of QT changes related to anlotinib, electrolyte abnormalities (hypokalemia, hypomagnesemia, hypocalcemia) must be corrected to the normal range before the first dose, and tested during the study treatment Electrolyte level. Patients who meet the DLT criteria and have prolonged QTcF (ie, the confirmed QTcF prolongs to an absolute value of >500 milliseconds or an increase of >60 milliseconds from the baseline) should discontinue the study treatment and undergo regular electrocardiogram (ECG) examinations until relief to <481 milliseconds or such as baseline QTcF >481 milliseconds, it returns to the baseline. If the toxicity subsides or returns to ≤1 (<481 milliseconds) within 14 days after the onset, the investigator can restart the study drug at a reduced dose as appropriate. If the QTcF prolonged toxicity does not fall to ≤ Grade 1 within 14 days, the patient will be permanently withdrawn from the study treatment.

c) Keratitis

Signs and symptoms of keratitis, such as acute or worsening: eye inflammation, tearing, sensitivity to light, blurred vision, eye pain and/or red eyes should be referred to an ophthalmologist immediately.

d) Permanent discontinuation due to toxicity

Patients with interstitial lung disease (ILD) or prolonged QTc interval with signs/symptoms of severe arrhythmia will not be allowed to restart study treatment.

e) Bleeding

Bleeding includes hemoptysis, gastrointestinal bleeding, nose bleeding, bronchial bleeding, gum bleeding, gross hematuria, fecal occult blood and cerebral hemorrhage. Patients with grade 2 bleeding should discontinue study treatment and give supportive treatment according to local practice/guidelines. If the toxicity subsides or returns to grade ≤1 within 14 days after the onset, the investigator may restart the study drug at a reduced dose as appropriate. If a patient has level 3 or higher bleeding, and the investigator believes that the event of concern is particularly related to the study drug (and has nothing to do with the disease or disease-related process that the patient is being treated for), the patient should permanently stop the study drug. In addition, symptomatic and supportive treatment should be taken immediately in accordance with

local practice/guidelines.

5.1.7. Treatment time

All eligible patients can obtain anlotinib combined with trametinib treatment plan by participating in this clinical trial. Treatment of the study drug will continue until disease progression, unacceptable toxicity, withdrawal from the study, or death as defined by RECIST. For patients with RECIST-defined progression, if the patient continues to show clinical benefit and there is no discontinuation standard, the investigator can decide whether to continue the study treatment at the discretion of the investigator. Progression follow-up defined after RECIST is optional and will be determined by the investigator. AEs were evaluated from the time of obtaining informed consent until at least 30/90 days after the last administration of trametinib or anlotinib.

If the study drug is discontinued for reasons other than disease progression, the patient must continue the RECIST version 1.1 assessment every 6 weeks until the disease progresses or the next-line treatment begins (whichever occurs first).

For the patients who discontinued the therapy due to COVID-19 infection or COVID-19-related reasons, they will continue to receive trametinib plus anlotinib therapy if the investigators think the patients still can receive benefit from the medication.

5.1.8. Treatment compliance and inventory

Study drugs should only be used as directed in this protocol. The detailed information of each patient treated with the study drug will be recorded in the case record form.

The patient should return all unused drugs and empty containers to the investigator.

Researchers at the research center will count all the medicines distributed and destroy them appropriately. The certificate of delivery and destruction will be signed.

6. Benefit/risk and ethical evaluation

6.1. Potential benefits

This is a phase I, open-label, single-center study of trametinib combined with anlotinib in KRAS (excluded $KRAS^{G12C}$) mutation-positive advanced NSCLC patients. This study aims to explore the best combined dosage while ensuring patient safety through close safety monitoring.

Previous studies have confirmed that MEK inhibitors have a certain clinical effect on KRAS-mutated NSCLC patients, but the effective time is short. The recurrence process is often accompanied by the activation of upstream RTK molecules, such as FGFR, VEGFR, c-KIT, etc. This suggests that the combined application of MEK inhibitors and multi-target anti-angiogenesis inhibitors should produce stronger and longer-lasting anti-tumor effects. We further demonstrated this synergistic effect through in vitro cell lines and in vivo mouse xenograft models. It is a potential targeted therapy for NSCLC with different subtypes of KRAS mutations. The recent emergence of G12C inhibitors has provided the accessible allosteric inhibitors for the $KRAS^{G12C}$-mutant NSCLC patients, but G12C accounts for 2.8% ~ 13% of NSCLC. There is still a large proportion of KRAS-mutant NSCLC patients (8.4% ~ 20% of NSCLC patients) who urgently need effective treatment plan. Therefore, we intend to explore the safety and survival benefits of trametinib combined with anlotinib in the treatment of KRAS-mutant (excluded $KRAS^{G12C}$) NSCLC patients.

6.2. Potential risks

The monitoring and management of potential risks are discussed as follows:

6.2.1. Gastrointestinal tract diseases

Patients with intractable nausea, vomiting, and chronic gastrointestinal diseases were

not included in this study. Investigators should also follow general toxicity management guidelines regarding interruptions and dose reductions.

Patients with active bleeding symptoms were excluded. If gastrointestinal bleeding occurs, including stool occult blood (++) or more, hematemesis or vomiting blood, symptomatic treatment should be given. Fasting, acid suppression, blood transfusion, and supportive treatment are given to patients with upper gastrointestinal bleeding. If necessary, octreotide can be used. If lower gastrointestinal bleeding occurs, hemostasis, blood transfusion, and supportive treatment should be given. If the bleeding cannot be controlled, surgery must be performed.

6.2.2. Skin diseases

This study did not include any specific dermatological exclusion criteria, but patients with unresolved AEs greater than CTCAE Grade 1 in previous treatments will be excluded from the study. For skin disease patients with any CTCAE-level skin reaction that the investigator believes is causally related to the study drug, dermatological treatment should be taken. Investigators should also follow general toxicity management guidelines regarding dose interruption and reduction. Photography can be taken to record any clinically important findings.

6.2.3. Cardiovascular system

Patients with risk factors for unstable cardiac conditions and prolonged QT interval will be excluded from this study. Where feasible, the simultaneous use of conventional drugs that may prolong the QT interval will be restricted, but patients can receive any drugs that are clinically indicated for the treatment of AEs. Throughout the study, electrolytes and vital signs assessments, including pulse rate and BP will be monitored regularly. Within 24 hours after the first study drug is taken, and at the assumed steady state (day 8 of continuous dosing), a series of three repeated digital ECG assessments will be performed, and a single record will be taken at the beginning of each subsequent treatment cycle ECG. The researcher or designated doctor will check each ECG before

leaving the hospital, and if appropriate, consult a local cardiologist to treat the patient immediately. ECG data suggest that a cardiologist should analyze heart rate, PR, R-R, and QT intervals. If the ECG abnormality at screening or baseline is deemed by the investigator to be clinically significant, it should be reported as a concurrent condition. For patients with cardiac risk factors that may affect left ventricular ejection fraction (LVEF), cardiac monitoring should be considered, including assessment of LVEF at baseline and during treatment. For patients with relevant cardiac signs/symptoms during treatment, cardiac monitoring, including LVEF assessment, should be considered.

Patients with uncontrolled hypertension were excluded. In the case of elevated BP, you need to actively communicate with your doctor. In the case of elevated BP, conventional antihypertensive therapy can be controlled. If BP is difficult to control, the target drug dose can be lowered or cancelled.

6.2.4. Respiratory system

Patients with a history of interstitial lung disease, drug-induced interstitial lung disease, radiation pneumonitis requiring steroid therapy, or any evidence of clinically active interstitial lung disease will be excluded from this study. If new or worsening pulmonary symptoms (such as dyspnea) or radiological abnormalities suggesting interstitial lung disease are observed, it is recommended to discontinue the dose of the study treatment and notify the sponsor research team. The expert group will discuss to rule out other causes such as lymphatic cancer, infection, allergies, cardiogenic edema or pulmonary hemorrhage. The results of the discussion will be collected. If there are confirmed HRCT imaging findings, but other causes of respiratory symptoms are excluded, the diagnosis of interstitial lung disease should be considered, and study treatment should be permanently terminated.

6.2.5. Liver diseases

Patients with any signs of severe or uncontrollable systemic liver disease, including

known hepatitis B, hepatitis C, human immunodeficiency virus (HIV) or liver enzyme abnormalities (defined as AST or ALT >2.5 × ULN, if there is no evidence of liver metastasis, total bilirubin >1.5 × ULN; AST or ALT with liver metastasis >5 × ULN, total bilirubin > 3 × ULN) does not participate in the study. During the study period, liver function tests will be regularly monitored during the study period and recorded.

6.2.6. Thyroid

During the entire treatment period, the thyroid function of all patients was closely monitored. When Thyroid Stimulating Hormone (TSH) ≥20mU/L or any value of T3, T4, FT3, FT4 is lower than the normal value, the corresponding treatment method should be used.

6.2.7. Hematopoietic function

If any of the following laboratory results (absolute neutrophil count <1.5 × 10^9/L; platelet count <100 × 10^9/L; hemoglobin <90 g/L) shows that patients with insufficient bone marrow reserve will be excluded from the study outside. Before the first dosing, hematological parameters will be monitored, once a week during the first cycle of multiple dosing, once at the beginning of each subsequent cycle, and once at the stop of the drug.

6.2.8. Hyperlipidemia

The treatment of hyperlipidemia should consider the patient's pre-treatment status and eating habits. In addition to diet control, hypercholesterolemia of grade 2 or higher (≥7.75 mmol/L) or hypertriglyceridemia of grade 2 or higher (≥2.5 × ULN) should also be given appropriate HMG-CoA reductase inhibitors (atorvastatin) or lipid-lowering drugs.

6.2.9. Reproductive organs

According to data from animal studies and the drug mechanisms, the use of anlotinib and trametinib in pregnant women may cause harm. There are currently no available data on the use of anlotinib and trametinib in pregnant women. Therefore, all men and women with reproductive potential will be required to take appropriate contraceptive measures during the study period and for an appropriate period thereafter. Women of reproductive potential must have a negative pregnancy test before receiving the first dose of study treatment. There are currently no data on the presence of study drugs or active metabolites in breast milk, and no data on the effect on breastfed infants or milk production. Women who breastfeed will be excluded from the study. According to studies on animals, treatment with anlotinib and trametinib may damage the fertility of men and women.

6.2.10. CYP450 induction/inhibition

All patients must avoid the simultaneous use of other drugs, herbal supplements and/or food intake that are known to have strong inducing effects on CYP3A4 activity. Such drugs must be discontinued within an appropriate time before entering screening and within 3 months after the last dose of study drug. If the patient is taking a drug that relies on BCRP and PgP. for distribution and has a narrow therapeutic index, it should be closely monitored to detect changes in tolerance due to increased exposure to the combined drug. See Appendix B for guidance on drugs to avoid, drugs that require close monitoring, and washout periods. Where medically feasible, except for strong inducers of CYP3A4 and inhibitors of CYP2C8, patients should maintain regular medication throughout the study. Patients can receive any medications that are clinically used to treat AEs.

7. Discontinue study drugs and withdraw from the study

Patients can stop using the study treatment in the following cases:

The patient's decision. The patient is free to withdraw from the study he/she is participating in at any time, without being affected by any prejudice.

Adverse events

Pregnancy

The researcher believes that serious non-compliance with this research protocol

The investigator assesses that the study drug has no clinical benefit

The patient starts to use the study drug incorrectly

Patients who withdrew from the study but can be evaluated will not be replaced. Any patients whose consent is withdrawn and cannot be evaluated will be replaced to ensure the minimum number of patients that can be evaluated.

7.1. Procedure for patients to stop study drug

Once the study drug is permanently disabled, it cannot be reactivated.

Patients who discontinue study treatment do not always automatically withdraw from the study. If the patient withdraws from the study, see section 7.3.

Any patient who discontinues study treatment for reasons other than objective disease progression should undergo tumor assessment scans in accordance with the plan in the protocol. Serious adverse events (SAEs) and concomitant medications related to the study procedure must be collected until the patient is no longer evaluated by RECIST version 1.1 (disease progression or permanent withdrawal from the study).

7.2. Procedures for patients who started using study drug incorrectly

In any case, subjects who do not meet the eligibility criteria should not be included in or receive study medication. There can be no exceptions to this rule. If a patient who does not meet the enrollment criteria is enrolled by mistake or starts treatment by mistake, or the patient does not meet the research criteria after the start, the investigator should decide when to terminate the study of the unqualified patient based on the patient's medical/safety risk.

7.3. Procedures for withdrawing from the study

Patients can freely withdraw from the study (study drugs and evaluation) at any time without affecting further treatment (withdrawal of consent). Such patients are always asked about the reason and whether there are any AEs. If possible, the investigator will examine them, perform the assessments and procedures required for post-study evaluation, and then withdraw from the study. AEs should be followed up; study medication should be returned by the patient.

8. Research plan and collection of research variables

8.1. The detailed steps of the study plan are shown in the table below

	Screening/Enrollment Visit		Treatment visit	Treatment discontinued	RECIST-defined progression follow-up	Safety 90-day follow-up	Survival follow-up
	-28 days	Day 1	Every 4-12 weeks		Every 4-8 weeks		Every 4 weeks
Time window (days)			+/- 7 days	+/- 7 days	+/- 7 days		+/- 7 days
Informed consent	X						
Confirm driver gene mutation status (a)	X						
Demographics and baseline characteristics	X						
Medical/surgical history	X						
Inclusion/exclusion criteria check	X						
Enough matched tissue and/or blood for biomarker analysis (b)	X						
Patient demographics	X						
Medical history	X						
Disease characteristics	X						
Combination therapy	X	X	X	X	X	X	

	Screening/Enrollment Visit		Treatment visit	Treatment discontinued	RECIST-defined progression follow-up	Safety 90-day follow-up	Survival follow-up
Complete blood count	X		X	If clinically indicated	If clinically indicated	If clinically indicated	
Biochemical tests in blood and urine (c)	X		X	X	X	X	
Thyroid function	X		X	X	X	X	
Coagulation	X		X	X	X	X	
Pregnancy test (only for premenopausal female patients)	X [(d)]						
Physical examination	X		X	X	X	X	
WHO fitness status	X		X	X	X		
ECG record	X		X	X	X	X	
Echocardiogram/MUGA(e)	X		X	X	X	X	
Vision check (slit lamp) (f)			If clinically indicated	If clinically indicated	If clinically indicated	If clinically indicated	
Chest CT	X		X	X	X		
Abdominal CT/BUS (g)	X		If clinically indicated	If clinically indicated	If clinically indicated		
Pelvis CT/BUS	X		If clinically indicated	If clinically indicated	If clinically indicated		
Brain CT/enhanced MRI	X		If clinically indicated	If clinically indicated	If clinically indicated		
Bone ECT	X		If clinically indicated	If clinically indicated	If clinically indicated		
Tumor sample and/or blood	If				If applicable to PD		

	Screening/Enrollment Visit		Treatment visit	Treatment discontinued	RECIST-defined progression follow-up	Safety 90-day follow-up	Survival follow-up
sample	applicable to the baseline						
Tumor evaluation (h)			X (every 4 weeks)	X	X (every 4 weeks)		
SAE, including overdose and pregnancy reports (d)(i)		X	X	X	X	X	
Adverse events		X	X	X	X	X	
Exit and reason				X			
Request for survival status information							X

(a) The driver gene mutation test may have been completed during the screening period.

(b) Tumor sample and blood sample collection: sufficient matched tissue and/or blood for biomarker analysis (formalin fixed paraffin embedded (FFPE) section or fresh tumor tissue or blood, enough for next generation sequencing (NGS) analysis). But the acquisition of tissue is not mandatory when the disease progresses.

(c) Serum or plasma: creatinine, total bilirubin, alkaline phosphatase, aspartate aminotransferase, alanine aminotransferase, albumin, potassium, magnesium, total calcium, sodium, creatine kinase; urine analysis (test paper): hemoglobin/red blood cell/blood, protein/albumin, glucose.

(d) The pregnancy test should be performed in accordance with Chinese standards.

(e) Perform an echocardiogram or multiple uptake gated acquisition (MUGA) scan to assess LVEF at least every 16 weeks during screening (before the first dose) and throughout the treatment period. The form of cardiac function assessment of individual patients must be consistent, that is, if it is used for screening and evaluation of echocardiography, echocardiography should also be used for subsequent scans. It should also be possible to use the same machine and operator

to examine the patient and perform quantitative measurements. If the treatment evaluation performed when the study treatment is stopped is abnormal, a 28-day follow-up evaluation is required to confirm the reversibility of the abnormality.

(f) Patients showing signs and symptoms suggestive of keratitis, such as acute or worsening: eye inflammation, tearing, sensitivity to light, blurred vision, eye pain and/or red eyes should be immediately referred to an ophthalmologist.

(g) Abdominal CT/Bultrasound (BUS): If no lesions are detected at baseline, BUS can be used as a method to identify abdominal lesions; however, if lesions have been detected at baseline and the lesion is the target lesion, BUS should not be used as a measurement Methods: CT scan should be used consistently during the screening and follow-up period as a tumor assessment method; for new lesions detected by BUS, it is recommended to confirm by CT. It is recommended to use brain MR as a diagnostic method and should be used during follow-up.

(h) Tumor evaluation: The investigator uses radiology to evaluate the tumor. However, all aspects of the tumor should be fully covered and recorded initially according to RECIST version 1.1 during the screening period. After that, during the follow-up period, another radiological examination should be performed every 4 to 8 weeks (Ñ7 days). For the same disease, whether it is the screening period or the follow-up period, the same evaluation techniques and methods should be used. Imaging tests used for tumor evaluation include: chest CT scan, abdominal CT scan/BUS, brain-enhanced MRI, and bone ECT (if clinically indicated).

(i) After stopping the study drug, SAEs related to the study procedure should continue to be collected until the disease progresses.

8.2. Enrollment/screening period

a. The enrollment/screening period is 28 days before the start of the study drug. The investigator will determine the eligibility of the patient based on the inclusion/exclusion criteria. The investigator will inform the patient about the potential benefits and risks of the study drugs (trametinib and anlotinib), AE reports, and other information collected within the scope of the study. Subsequently, the researcher will obtain an informed consent form signed by potential patients or their

legally acceptable representatives in accordance with Chinese law.

b. Driver gene mutations: the existence of driver gene mutations should be determined before starting to use investigational drug therapy. The inspection will be performed by NGS or another method (PCR-based method for EGFR detection and fluorescent in situ hybridization (FISH) or immunohistochemistry (IHC) for ALK rearrangement).
c. Baseline demographic characteristics: patient characteristics including age, gender, etc. will be collected.
d. Related medical history: comorbidities and related medical history (including any chronic diseases that currently require medical treatment).
e. Physical examination: it will be recorded at the baseline, and every visit (every 4 weeks) needs to be recorded.
f. Body weight: It will be recorded at the baseline, and every visit (every 4 weeks) needs to be recorded once.
g. Disease characteristics: tumor histology, diagnostic staging, current stage of enrollment study.
h. Medical history: The type of neoadjuvant therapy or adjuvant therapy (if any) received (targeted or non-targeted) and drugs received, start and end date, radiotherapy (yes/no), must be recorded before enrollment, Surgery (yes/no).
i. Related concomitant medications: For SAE, AEs of special concern, AEs that lead to dose adjustment/discontinuation of study drugs.
j. Laboratory evaluation: The researcher evaluates the laboratory parameters as normal or abnormal, and fills in the CRF with detailed information about the abnormality.
k. ECG recording: it will be recorded in the baseline, if there are clinical indications, it needs to be repeated.
l. Visual inspection: it will be recorded in the baseline, if there are clinical indications, it will need to be performed again.
m. Tumor evaluation: A baseline tumor evaluation should be performed within 28 days

before starting the study drug. The imaging evaluation performed at baseline should include: chest CT scan, abdominal CT scan/BUS, brain CT/enhanced MRI (if clinical indications), bone ECT (if clinical indications), fluorodeoxyglucose (FDG) positron emission tomography CT (PET-CT, if any Clinical indications).

AE/SAE: It should be recorded after international classification of functioning (ICF) signature.

8.3. Treatment period

The supply of study medication must always precede the treatment visit.

In addition, during each visit:

Need to screen patients for reportable safety events.

Tumor evaluation (effectiveness evaluation) needs to be recorded in the CRF.

The investigator must evaluate the patient's physical status and confirm that the patient continues to benefit from the study drug when providing the study drug.

If the patient withdraws from treatment and/or completely withdraws from the study, the investigator will record the reason for withdrawal. Please note that if the reason for withdrawal is an event that meets the SAE definition, it must be recorded in the safety and clinical form.

For patients who are lost to follow-up, all reasonable efforts must be made to track toxicity, determine their survival status, and record it in the CRF.

Study drugs must be properly stored and distributed from a safe storage area (or central pharmacy). The research center must maintain an inventory record of the receipt, distribution, destruction, and return of medicines.

Tumor evaluation: The investigator uses radiology to evaluate the tumor. However, all aspects of the tumor should be fully covered and recorded initially according to RECIST version 1.1 during the screening period. After that, radiological examinations should be performed every 4 to 8 weeks (±7 days) during the follow-up period. For the same disease, whether it is the screening period or the follow-up period, the same evaluation techniques and methods should be used. Imaging examinations used for

tumor evaluation include: chest CT scan, abdominal CT scan/BUS (if clinical indications), brain-enhanced MRI (if clinical indications), and bone ECT (if clinical indications).

8.4. Safety follow-up period

The patient must be followed up during the designated follow-up period (30 days after the last dose of study drug is administered) to collect SAEs that occurred during this period, and provide other reports as required.

8.5. Progressive follow-up period

After the study drug is discontinued for reasons other than disease progression, the patient will continue to be evaluated every 4 weeks until objective progress is achieved, the next course of treatment is started or the follow-up is lost, whichever occurs first. Patients who continue to receive treatment after achieving objective progress due to clinical benefits will be assessed for tumors and the investigator's response assessment will be collected.

Blood samples and/or tumor tissue can be collected when disease progression is achieved.

AE/SAE/radiation-induced damage: It should be collected from the informed consent of the entire treatment period, including the follow-up period.

8.6. Survival follow-up period

As the disease progresses, the patient, the patient's family or the patient's current doctor must be contacted every 4 weeks for survival information. The patients who were lost to follow-up whose last contact status was recorded as "alive" will be reviewed.

9. Research Evaluation

The investigator should ensure that the data is recorded on the case report form specified in the research protocol and recorded in accordance with the instructions provided.

Researchers ensure the accuracy, completeness and timeliness of the recorded data, and respond to data queries. The investigator will sign the case report form. A copy of the complete medical record report form will be archived in the research center.

9.1. Effectiveness evaluation

The RECIST version 1.1 standard will be used to assess the patient's response to treatment by determining the ORR, disease control rate (DCR), duration of response (DOR) and PFS.

Within 28 days after starting treatment, CT/BUS of the chest and abdomen (including liver and adrenal glands) at baseline were used for imaging evaluation. Then check every 4 to 12 weeks ± 7 days until objective disease progression occurs or the study is withdrawn. In addition, other areas should be checked based on the patient's signs and symptoms. Any other areas suspected of having a new disease should also undergo appropriate imaging examinations. If an unplanned evaluation is performed and the patient has no disease progression, every effort should be made to conduct a follow-up evaluation at the scheduled visit.

The classification of objective tumor response assessment will be based on RECIST version 1.1 response criteria: CR, PR, SD and PD. Calculate the progression of the target lesion (TL) and compare it with the time when the tumor burden is minimal (that is, the sum of the smallest diameters previously recorded in the study). In the absence of disease progression, the tumor response (CR, PR, SD) is calculated and compared with the baseline tumor measurements obtained before starting treatment. CR/PR/SD should be confirmed at least 4 weeks later and accompanied by the follow-up interval specified in the study plan.

If there is progressive disease, the progress will be recorded as a visit response (RECIST version 1.1).

If the investigator has doubts about the occurrence of disease progression, especially the response to NTL (non-target lesion) or the emergence of new lesions, it is recommended to continue treatment until the next planned evaluation or earlier (if there are clinical indications), and reassess the patient's status. If repeated scans confirm disease progression, the date of the initial scan should be declared as the date of disease progression.

In order to be judged as "clearly progressing" on the basis of non-targeted diseases, non-targeted diseases must have an overall level of substantial deterioration. In this way, even if the targeted disease has SD or PR, the overall tumor burden has been increased enough and it is worth stopping treatment. A moderate "increase" in the size of one or more non-target lesions is usually not enough to ensure a clear state of disease progression.

It is important to follow the assessment schedule as strictly as possible.

9.2. Safety assessment

9.2.1. Laboratory safety assessment

The laboratory safety assessment parameters are listed in the table:

Clinical chemistry	Hematology
Serum (S) / Plasma (P)-Albumin	Blood (B)-hemoglobin
S/P-ALT	B-leukocytes
S/P-AST	B-hematocrit
S/P-Alkaline Phosphatase	B-Red blood cell (RBC) count
S/P-total bilirubin	B-Absolute white blood cell classification count:
S/P-total calcium	Neutrophils
S/P-creatinine	Lymphocytes
S/P-glucose (fasting only on PK days)	Monocyte
S/P-Lactate Dehydrogenase (LDH) 2	Basophils
S/P-magnesium	Eosinophils
S/P-potassium	B-platelet count
S/P-Sodium	B-reticulocyte
S/P-urea nitrogen or blood urea nitrogen	Urinalysis
S/P-lipase	U-glucose
S/P-Amylase	U-protein
S/P-total cholesterol	U-blood
S/P-total triglycerides	Coagulation
S/P- High Density Lipoprotein	P-prothrombin time (PT)
S/P- Low Density Lipoprotein	P-activated partial thromboplastin time (APTT)
Thyroid function	P-thrombin time (TT)
S-Triiodothyronine (T3)	P-fibrinogen (Fbg)
S-thyroxine (T4)	P-D-dimer
S-TSH	P- INR
S-free T3	
S-free T4	

Blood and urine samples will be collected at the time specified in the study plan to determine clinical chemistry, hematology, thyroid function, coagulation function, and urinalysis. If the researcher believes that there are clinical indications, other safety samples can be collected. Acquisition date, time and results (value, unit and reference range) will be recorded in the appropriate CRF.

Clinical chemistry, hematology, thyroid function, coagulation function and urinalysis will be performed in Shanghai Chest Hospital, Shanghai Jiao Tong University School

of Medicine.

In addition, during the screening visit, pregnancy tests are only performed on women with reproductive potential (according to the standard clinical practice of the research center, blood or urine tests are acceptable).

Researchers should evaluate existing results regarding clinically relevant abnormalities. Laboratory results should be signed and dated, and stored in the research center as the source data of laboratory variables.

Electrolyte abnormalities (hypokalemia, hypomagnesemia, hypocalcemia) must be corrected to within the normal range before the first dose, and electrolyte levels should be monitored during the study treatment.

9.2.2. Physical examination

A physical examination will be performed, including evaluation of the following: general appearance, skin, head and neck (including ears, eyes, nose, and throat), respiratory system, cardiovascular, abdomen, lymph nodes, thyroid, abdomen, and central nervous system.

9.2.3. Electrocardiogram

All patients will undergo a 12-lead digital electrocardiogram during the study visit. After the patient rests on the supine side for at least 10 minutes before the specified time, a 12-lead ECG will be obtained, which should be recorded at a speed of 25 mm/sec. All ECGs should be recorded in the same position of the patient. For each time point, three ECG results should be recorded in approximately 5 minutes. The digital ECG records will be collected, analyzed and stored by the central ECG supplier. If there is an abnormality in the evaluation during the treatment when the study treatment is stopped, a 28-day follow-up evaluation is required to confirm the reversibility of the abnormality.

9.2.4. Echocardiography/MUGA Scan

Echocardiography or MUGA scans will be performed at screening (before the first treatment) and at least every 16 weeks throughout the treatment period to assess LVEF. The method of cardiac function assessment must be consistent within the patient (i.e., if echocardiography is used for screening assessment, echocardiography should also be used for subsequent scans). As far as possible, the same machine and operator should be used to examine patients and perform quantitative measurements. If there is an abnormality in the evaluation during the treatment when the study treatment is stopped, a 28-day follow-up evaluation is required to confirm the reversibility of the abnormality.

9.2.5. Vital signs

Pulse and BP

After resting for 10 minutes, BP and pulse rate in the supine position will be measured. As indicated in the research plan, evaluation will be performed at the time of the visit. In addition, if there are clinical indications, it should be at the discretion of the investigator. If applicable, any change in vital signs should be recorded as an AE.

Weight and height

Body weight assessment will be performed at the time of screening, and then on day 1 of each cycle and at the discontinuation visit. Evaluate height only during screening.

10. Safety report and medical management

The principal investigator is responsible for ensuring that all personnel involved in the research are familiar with the content of this section.

10.1. Definition of adverse events

AEs refer to adverse medical conditions or deterioration of the original medical conditions after or during exposure to the study drugs, regardless of whether there is a causal relationship with the drug. A bad medical condition may be symptoms (for example, nausea, chest pain), physical signs (for example, tachycardia, enlarged liver), or abnormal test results (for example, laboratory test results, electrocardiogram). In clinical studies, AEs include adverse medical conditions that occur at any time, including the lead-in period or wash-out period, even if no research treatment has been performed.

The term AE includes serious and non-serious AEs.

10.2. Definition of serious adverse events

SAE refer to AEs that occur during any research phase (i.e., lead-in period, treatment period, washout period, follow-up period), which meet one or more of the following criteria:

Causes death

Immediate life threatening

Need to be hospitalized or extend the current hospital stay

Cause permanent or severe disability/loss of function or severely disrupt normal life functions

Congenital malformations or birth defects

It is a major medical event that may endanger the subject or may require medical intervention to prevent one of the above outcomes

10.3. Adverse Event Record

10.3.1. Time period for collecting adverse events

AEs and SAEs will be collected from the time of informed consent throughout the treatment period, including the follow-up period (90 days after the last study drug administration).

10.3.2. Follow-up of unresolved adverse events

In the study, the investigator will follow up any AEs that the subject has not resolved in the last AE assessment based on medical indications, but no further records are required in the CRF.

10.3.3. Variables

The severity of AE is classified according to NCI CTCAE 5.0, using a 5-point scale (1-5 grades), and is reported in detail on CRF. The grades of AEs not included in CTCAE are as follows:

CTCAE grade	Equal:	definition
Level 1	Mild	Feel uncomfortable and do not interfere with normal activities of daily life.
Level 2	Moderate	Feeling of discomfort is sufficient to reduce or affect activities of daily living; treatment or medical intervention is not indicated, although these measures can improve the patient's overall health or symptoms.
Level 3	Severe	Inability to work or normal activities of daily life; treatment or medical intervention is required to improve the patient's overall health or symptoms; delay in treatment has no direct harmful effect on the patient's survival.
Level 4	Life-threatening or disabling	Directly endanger life or cause permanent mental or physical illness, affecting work or normal activities of daily life; treatment or medical intervention is required to maintain life.
Level 5	Fatal	AE causes death

10.3.4. Causality collection

The investigator will evaluate the causal relationship between the trial drug and each AE, and answer "yes" or "no" to the question "Do you think the event may be caused by the trial drug?"

For SAE, the causal relationship between other drugs and the research process will also be evaluated. Please note that for SAEs that may be related to any research process, causality is "yes".

For guidance on the interpretation of causality issues, please see Appendix A of the "Clinical Research Protocol".

10.3.5. Adverse events based on signs and symptoms

All AEs reported by the subject spontaneously or when answering open-ended questions from the researcher: "Did you have any health problems since the last visit/last interview?" or found through observation. The information will be collected and recorded in the CRF. When collecting AEs, it is best to record the diagnosis, not the signs and symptoms. However, if the diagnosis result is known and there are other signs or symptoms that are not normally included in the diagnosis, the diagnosis and each sign or symptom will be recorded separately.

10.3.6. Adverse events based on inspections and trials

The results of laboratory tests and vital signs specified in the protocol will be summarized in the clinical study report. Therefore, compared to the baseline set by the protocol, if the deterioration meets any SAE criteria or the reason for discontinuation of the trial drug treatment, it should only be reported as an AE.

If the deterioration of laboratory test values/vital signs is related to clinical signs and symptoms, the signs or symptoms will be reported as an AE, and the related laboratory results/vital signs will be treated as other information. Investigators use clinical terms as much as possible for reporting, rather than laboratory terms (for example, anemia

and low hemoglobin values). In the absence of clinical signs or symptoms, clinically relevant deterioration of non-mandatory parameters should be reported as an AE. Deterioration of laboratory test values clearly due to disease progression should not be reported as AE/SAE.

Compared with the baseline assessment, any new or aggravated clinically relevant abnormal medical results found during the physical examination will be reported as an AE.

10.3.7. Disease progression

Disease progression can be considered to be the deterioration of the subject's disease caused by the disease under investigation by the experimental drug. It may be an increase in the severity of the disease under study and/or an increase in disease symptoms. New metastasis or progression to existing metastases should be considered disease progression, not AEs. Events that are clearly due to disease progression should not be reported as AEs during the study period.

The progression of the malignant tumor in the study, including the progression of signs and symptoms, should not be reported as a serious AE. Hospitalization due to signs and symptoms of disease progression should not be reported as a serious AE.

10.4. Report of serious adverse events

The investigator is responsible for notifying the ethics committee and sponsor of any SAE.

The investigator must inform the local ethics committee of any SAE within 24 hours.

All SAEs that lack important or relevant information should be followed up immediately. The investigator should inform the sponsor representative of any follow-up information of previously reported SAEs within 24 hours after learning.

11. Statistical methods

The analysis will be performed by the researcher or other designated third-party provider. A comprehensive statistical analysis plan (SAP) will be prepared, and any subsequent revisions will be recorded and final revisions will be made before reporting the data. All data will be provided for the complete analysis set. All patients who received at least 1 dose of study drug will be included in the study analysis.

11.1. Definition of study endpoint

In order to achieve the purpose of this study, data on the following endpoints will be collected:

Part A

Primary endpoint: To define the RP2D of the combined strategy of trametinib and anlotinib for advanced NSCLC patients with KRAS mutation (excluded KRAS^{G12C}).

Secondary endpoint: To evaluate the ORR, PFS, OS and safety of the combined strategy of trametinib and anlotinib for advanced NSCLC patients with KRAS mutation (excluded KRAS^{G12C}).

Part B

Primary endpoint: To evaluate the ORR of the combined strategy of trametinib and anlotinib for advanced NSCLC patients with KRAS mutation (excluded KRAS^{G12C}) who previous had been received 1st or more lines standard therapy.

Secondary endpoint: To evaluate the PFS, OS and safety of the combined strategy of trametinib and anlotinib for advanced NSCLC patients with KRAS mutation (excluded KRAS^{G12C}) who previous had been received 1st or more lines standard therapy.

Exploratory endpoint: dynamic changes of biomarkers and mutation profiles related to the efficacy and drug resistance of trametinib combined with anlotinib

11.2. Calculation of sample size

The primary endpoint of this study was to determine RP2D based on the incidence of DLT in cycle 1. For the dose-escalation stage (Part A), "3 + 3" principle was used. Briefly, trametinib is set to a single dose (2mg QD, oral) throughout the part A. Anlotinib is set to four levels of dose (6mg, 8mg, 10mg, 12mg, once a day, from day 1 to day 14 of the 21-day cycle). Three patients will be enrolled in primary combination treatment (trametinib, 2mg QD, oral; anlotinib, 6mg, once a day, from day 1 to day 14 of the 21-day cycle). If one patient occurs DLT in cycle 1, another 3 patients will be enrolled to further verification the safety. If two or more patients occur DLT in cycle 1, it is suggested that the exploration can be carried out in a lower dose group. If no patient occurs DLT in cycle 1, next dose combination treatment will be performed. If two patients screened and enrolled in a same dose group simultaneously, four patients in a single dose group are allowable. The sponsor and investigators will review the safety and clinical data of all subjects to jointly determine RP2D of anlotinib combined with trametinib.

If RP2D is reached in Part A, another 20 eligible patients will be enrolled and treated with trametinib (2mg QD, oral) + anlotinib (RP2D, once a day, from day 1 to day 14 of the 21-day cycle), until the PD or unacceptable toxicity occurs to further evaluate the safety, tolerability and efficacy. The primary endpoint for evaluation of efficacy of part B is ORR. The standard-of-care for advanced NSCLC patients with non-G12C KRAS mutations treated in this setting is docetaxel, which is associated with an ORR of up to 23%. Assuming trametinib plus anlotinib will result in an ORR of at least 55% in this setting, a sample size of approximately 20 evaluable patients would be sufficient for the lower bound of a 2-sided 95% confidence interval (Clopper-Pearson method) to exclude an ORR of 23%. If an ORR of at lower 40%, the part B study will be terminated.

11.3. Statistical analysis

All data will be provided for the FAS. All patients who received at least one dose of

study drug will be included in the study analysis. For all variables, descriptive statistics will be performed as appropriate. Continuous variables will be summarized by observations, mean, standard deviation, median, minimum, and maximum. Categorical variables will be aggregated by frequency counts and percentages for each category.

The FAS includes all patients who received at least one dose of study drug. The FAS will be used for all efficacy and safety analysis.

- Safety and tolerability

The safety analysis set will include all patients who have received at least one dose of the therapeutic drug. The patient will be evaluated based on the actual treatment received. The safety and tolerability summary will be based on the safety analysis set.

AE will be coded according to the preferred term of SOC and MedDRA terminology. The severity of AE will be graded according to NCI CTCAE version 5.0. A list of all AEs will be listed, including detailed information collected for each AE. (Description of event, date/time of onset, duration, severity, severity, relationship with study drug, measures taken, clinical outcome).

- Tumor response

Tumor response data will be listed and summarized by dose group, and the following response categories will be used when appropriate: CR, PR, SD, PD, and NE. For the definitions of CR, PR, SD and PD, please refer to the RECISIT version 1.1 standard. Where appropriate, the objective tumor response rate is given with a 95% confidence interval (calculated using the Clopper-Pearson interval).

Objective response rate (ORR) is defined as the percent of patients documented to have a confirmed CR or PR.

Descriptive statistics (frequency and percentage) for ORR based on response assessments by investigator. Patients who cannot be assessed for response will be counted as not evaluable. Descriptive statistics (frequency and percentage) for CR and PR rate will be presented overall.

- Duration of response

The response duration of the responding patients will be summarized, and the number

of responding patients (%) for the duration of remission> 3;> 6;> 9;> 12 months will be given. The Kaplan Meier chart and the median duration of response with 95% confidence interval (95% CI, calculated based on the Kaplan Meier chart) will be given. For the patients who discontinued the therapy due to COVID-19 infection or COVID-19-related reasons, they will continue to receive trametinib plus anlotinib therapy if the investigators think the patients still can receive benefit from the medication.

- PFS analyses

PFS is defined as the time from the date of first treatment to the date of first PD or death due to any cause in the absence of documented PD, whichever occurs first. PFS (in days or months) will be calculated.

The PFS of the part A, part B, overall, subgroups will be summarized. PFS will be displayed using Kaplan-Meier curves. The number of events, the median (calculated based on the Kaplan-Meier curve), and the proportion of patients with no events at 6, 12, and 18 months will be summarized. Where appropriate, a summary of the number and percentage of patients who have died, are still undergoing follow-up, lost follow-up, and dropped out of the study will be provided.

For the patients who discontinued the therapy due to COVID-19 infection or COVID-19-related reasons, they will continue to receive trametinib plus anlotinib therapy if the investigators think the patients still can receive benefit from the medication.

- Integrative analyses

For the patients enrolled in phase I clinical study (including part A and part B), the clinical characteristics, ORR, PFS, and AEs will be analyzed.

- Subgroup analyses

According to the mutation types of non-G12C KRAS, the FAS will be divided into $KRAS^{G12V}$ subtype, $KRAS^{G12D}$ subtype, and $KRAS^{other}$ subtype. ORR and PFS will be calculated based on the different KRAS mutation subtypes. In addition, tumor shrinkage (percent change from baseline) will be presented for different KRAS mutation subtypes.

- Biomarkers and mutation profiles

All patients participating in the biomarker study will be included in the analysis set.

12. Ethical considerations

This research will be conducted in accordance with legal and regulatory requirements, as well as the International Code of Ethics for Biomedical Research Involving Human Subjects (International Council of Medical Scientific Organizations, 2002) and the Declaration of Helsinki (World Medical Association, 1996 and 2008). In addition, this research will be conducted in accordance with the plan and applicable Chinese regulatory requirements and laws.

Unless required by law, all parties should ensure the protection of the subject's personal data and must not include the subject's name in any sponsor form, report, publication or any other disclosure. The subject's name, address, date of birth, and other identifiable data will be replaced with an alphanumeric code composed of a numbering system. The informed consent must comply with International Conference on Harmonization (ICH) GCP, regulatory requirements and legal requirements. The informed consent used in this study and any changes made during the study must be pre-approved by Institutional Review Board (IRB)/ Independent Ethics Committee (IEC) before use. Researchers must ensure that each research subject or his legal representative fully understands the nature and purpose of the research and the possible risks associated with participation. The researcher or the personnel designated by the researcher shall obtain the written informed consent of each subject or the subject's legal representative before conducting any specific research activities. The investigator will retain the original copy of the informed consent that each subject has signed.

References

1. M. Drosten, M. Barbacid, Targeting the MAPK Pathway in KRAS-Driven Tumors. *Cancer Cell* **37**, 543-550 (2020).
2. P. Liu, Y. Wang, X. Li, Targeting the untargetable KRAS in cancer therapy. *Acta Pharm Sin B* **9**, 871-879 (2019).

3. D. S. Hong *et al.*, KRAS(G12C) Inhibition with Sotorasib in Advanced Solid Tumors. *N Engl J Med* **383**, 1207-1217 (2020).

4. A. R. Moore, S. C. Rosenberg, F. McCormick, S. Malek, RAS-targeted therapies: is the undruggable drugged? *Nat Rev Drug Discov* **19**, 533-552 (2020).

5. P. A. Janne *et al.*, Selumetinib Plus Docetaxel Compared With Docetaxel Alone and Progression-Free Survival in Patients With KRAS-Mutant Advanced Non-Small Cell Lung Cancer: The SELECT-1 Randomized Clinical Trial. *Jama* **317**, 1844-1853 (2017).

6. E. Manchado *et al.*, A combinatorial strategy for treating KRAS-mutant lung cancer. *Nature* **534**, 647-651 (2016).

7. C. Sun *et al.*, Intrinsic resistance to MEK inhibition in KRAS mutant lung and colon cancer through transcriptional induction of ERBB3. *Cell reports* **7**, 86-93 (2014).

8. A. W. Tolcher *et al.*, A phase IB trial of the oral MEK inhibitor trametinib (GSK1120212) in combination with everolimus in patients with advanced solid tumors. *Annals of oncology : official journal of the European Society for Medical Oncology* **26**, 58-64 (2015).

9. H. Singh, D. L. Longo, B. A. Chabner, Improving Prospects for Targeting RAS. *Journal of clinical oncology : official journal of the American Society of Clinical Oncology* **33**, 3650-3659 (2015).

10. C. Fedele *et al.*, SHP2 Inhibition Prevents Adaptive Resistance to MEK Inhibitors in Multiple Cancer Models. *Cancer Discov* **8**, 1237-1249 (2018).

11. Y. Sun *et al.*, Safety, pharmacokinetics, and antitumor properties of anlotinib, an oral multi-target tyrosine kinase inhibitor, in patients with advanced refractory solid tumors. *Journal of hematology & oncology* **9**, 105 (2016).

12. B. Han *et al.*, Effect of Anlotinib as a Third-Line or Further Treatment on Overall Survival of Patients With Advanced Non-Small Cell Lung Cancer: The ALTER 0303 Phase 3 Randomized Clinical Trial. *JAMA Oncol* **4**, 1569-1575

(2018).

13. Y. Chi *et al.*, Safety and Efficacy of Anlotinib, a Multikinase Angiogenesis Inhibitor, in Patients with Refractory Metastatic Soft-Tissue Sarcoma. *Clinical cancer research : an official journal of the American Association for Cancer Research* **24**, 5233-5238 (2018).

14. D. Wu *et al.*, A phase II study of anlotinib in 45 patients with relapsed small cell lung cancer. *Int J Cancer* **147**, 3453-3460 (2020).

Appendix material

Appendix A Other safety information

Further guidance on the definition of SAE

- Life threatening

"Life-threatening" means that the subject is immediately at risk of death when an AE occurs, or it is suspected that the use or continued use of the drug will cause the subject's death. "Life-threatening" does not mean that the AE occurs in a more serious form, which may lead to death (for example, hepatitis that has healed without liver failure).

- Hospitalization

Outpatient treatment in the emergency room itself is not a serious AE, although the cause may be (for example, bronchospasm, laryngeal edema). If the disease or disease existed before the subject was included in the study, and the disease did not worsen in an unexpected manner during the study, the hospitalization and/or surgery planned before or during the study is not considered an AE.

- Major medical incident or medical intervention

Major medical events may not immediately endanger life or cause death, hospitalization, disability or incapacity, but may endanger the subject or may require medical intervention to prevent one or more of the outcomes listed in the definition of a serious AE Under the circumstances, medical and scientific judgments should be made to determine whether the case is serious. These should generally be considered serious.

Merely stopping the use of suspicious drugs does not mean that this is a major medical incident; medical judgment must be used.

• Angioedema is not severe enough to require intubation, but requires intravenous hydrocortisone treatment

• Hepatotoxicity caused by paracetamol (acetaminophen) overdose requires N-acetylcysteine treatment

• Intensive treatment of allergic bronchospasm in the emergency room or at home

• Unbalanced blood quality (such as neutropenia or anemia that requires blood

transfusion) or convulsions, but will not lead to hospitalization

- A guide to explaining causality issues

When evaluating causality, the following factors need to be considered to determine whether there is a "reasonable possibility" that the drug may cause an AE.

• Time course. Exposure to suspicious drugs. Did the subject actually take the suspicious drug? Does the AE have a reasonable time relationship with the taking of the suspicious drug?

• Consistent with known drug characteristics. Are AEs consistent with previous knowledge of suspected drugs (pharmacology and toxicology) or drugs of the same pharmacological category? Or can AE be predicted from its pharmacological properties?

• Experience to stimulate. Did the AE heal or improve when the dose of the suspected drug was stopped or reduced?

• There is no other reason. AE cannot be reasonably explained by other causes (such as underlying diseases, other drugs, other hosts, or environmental factors).

• Reinvigorating experience. If the suspicious drug is reintroduced after the drug is stopped, will the AE happen again? It is generally not recommended to re-inspire.

• Laboratory testing. A specific laboratory investigation (if performed) has confirmed this relationship.

- In difficult situations, other factors can be considered, such as:

• Is this a recognized feature of drug overdose?

• Is there a known mechanism of action?

"Relevant" causality refers to the "reasonable possibility" of causality in individual cases after reviewing relevant data. The term "reasonable possibility" of causality usually refers to facts (evidence) or arguments that indicate the existence of causation. Perform causality assessment based on existing data that includes enough information to make informed judgments. If the information in the case is limited or insufficient, the incident may be assessed as "irrelevant."

The causality of the deterioration of the research disease due to lack of validity should be classified as unreasonable possibility.

Appendix B Guidance on potential interactions of concomitant drugs

The use of any natural/herbal products or other "civil treatments" is discouraged, but the use of these products and the use of all vitamins, nutritional supplements and all other combined drugs must be recorded in the electronic documents.

It is strongly recommended that drugs that induce CYP3A4 metabolism should not be used in combination with anlotinib

Anlotinib is metabolized by CYP3A4 and CYP3A5 enzymes.

A drug-drug interaction study of anlotinib in patients showed that when co-administered with a strong inducer of CYP3A4, the effectiveness of anlotinib may be impaired. In vitro and in vivo studies have shown that trametinib is mainly metabolized by deacetylation alone or in combination with oxidation. The metabolites after deacetylation are further metabolized by glucuronidation. CYP3A4 oxidation is regarded as a secondary metabolic pathway. Deacetylation is mediated by carboxyl esterase 1b, 1c, and 2, and other hydrolases may also play a role. Based on the lower dose and lower clinical systemic exposure relative to the in vitro inhibitory value or induction value, trametinib is not considered to be an in vivo inhibitor or inducer of these enzymes or transporters, and it is unlikely to pass through the CYP enzyme or transporter. Protein interactions significantly affect the pharmacokinetics of other drugs.

In this study, any patient receiving anlotinib should not use the following strong CYP3A4 inducers.

Contraindicated drugs	Withdrawal period before starting study drug treatment
Carbamazepine, phenobarbital, phenytoin, rifampicin, rifabutin, rifapentin	3 weeks
St. John's Wort	
Phenobarbital	5 weeks

This list is not exhaustive, and similar contraindications will apply to other drugs known to strongly modulate the activity of CYP3A4. Appropriate medical judgment is required.

Appendix C CTCAE V5.0

<table>
<tr><td rowspan="5">V5.0CTCAE classification</td><td>Level 1</td><td>Mild; asymptomatic or mild; only clinical or diagnostic observation; or no intervention is indicated</td></tr>
<tr><td>level 2</td><td>Moderate; minimal, partial or non-invasive intervention; or restrict age-appropriate instrumental activities of daily living</td></tr>
<tr><td>Level 3</td><td>Severe or medically significant, but not immediately life-threatening; leading to hospitalization or prolonged hospitalization; disabled; or restricting self-care activities of ADL in daily life</td></tr>
<tr><td>level 4</td><td>Life-threatening consequences or emergency intervention</td></tr>
<tr><td>Level 5</td><td>AE-related deaths</td></tr>
<tr><td rowspan="7">Severity</td><td colspan="2">A serious adverse event refers to any adverse event that meets one or more of the following criteria at any dose or during the administration of any study drug:</td></tr>
<tr><td colspan="2">†Causes or causes death;</td></tr>
<tr><td colspan="2">†Life-threatening; or, the researcher believes that the adverse event puts the patient at direct risk of death (Note: This does not include any adverse event that occurs in a more serious form or is allowed to continue to occur, which may lead to death.);</td></tr>
<tr><td colspan="2">†Causes permanent or severe disability/loss of function (i.e., adverse events cause the patient's ability to perform normal life functions to be severely affected);</td></tr>
<tr><td colspan="2">† is a congenital abnormality/birth defect (in the offspring of subjects who used the drug without considering the time of diagnosis);</td></tr>
<tr><td colspan="2">†The investigator judged it to be a major medical event; although the event is not fatal, life-threatening or requires hospitalization, according to appropriate medical judgment, the event may endanger the subject and requires medical or surgical intervention to prevent it from occurring One of the above endings (marked as †). Such events can also be regarded as serious adverse events.</td></tr>
<tr><td colspan="2">† is a congenital abnormality/birth defect (in the offspring of subjects who used the drug without considering the time of diagnosis);</td></tr>
<tr><td>duration</td><td colspan="2">Record the start and end date of the adverse event. If it is less than 1 day, please specify the appropriate time length and unit.</td></tr>
<tr><td>the measures taken</td><td colspan="2">Does the study of adverse drug events lead to the discontinuation of the study drug?</td></tr>
<tr><td rowspan="6">Relationship with the product</td><td colspan="2">Will study drugs cause adverse events? Medically qualified investigators should provide evaluation results of the relationship between study drugs and adverse events. The investigator should sign the name/date (first letter) on the original document or worksheet to support the evaluation of the causality of the adverse event and ensure that the evaluation of the causality meets the medical qualifications. The signed documents must be kept within the prescribed supervision period. The following standards will serve as a reference guide to help investigators assess the relationship between study drugs and adverse events based on existing information.</td></tr>
<tr><td>Exposed</td><td>Is there evidence that the subject has actually been exposed to the study drug, such as true past medical history, acceptable compliance assessment (drug count, log, etc.), expected pharmacological effects, and drug/metabolite effects in the specimens collected in the body Measurements?</td></tr>
<tr><td>Time course</td><td>Is there a reasonable time sequence between AE and study drug treatment?</td></tr>
<tr><td>possible reason</td><td>Does the adverse event occur at the same time as the adverse event caused by the drug?</td></tr>
<tr><td>Deprovocation test</td><td>Has the study drug been discontinued or has the dose/exposure/frequency reduced?
If yes, is the AE cured or improved?
If it is, the deprovocation test result is positive. If it is not, the result of the de-provocation test is negative.
Note: If (1) an adverse event leads to death or permanent disability; (2) despite the continued use of the study drug, the AE can still be cured/improved; (3) the test is a single-dose drug test; (4) the study drug is only administered Once, the standard does not apply.</td></tr>
<tr><td>Reprovocation</td><td>Is the subject repeatedly exposed to the study drug during the trial?</td></tr>
</table>

	test	If yes, does the AE recur or worsen? If it is, the result of the re-provocation test is positive. If it is not, the result of the re-provocation test is negative.<br>Note: If (1) the AE results in death or permanent disability, or (2) the test is a single-dose test, or (3) the study drug is administered only once, then this standard does not apply.<br>Note: Planning a reprovocation test for serious AEs that may be caused by the study drug or re-exposure to the study drug may pose a serious potential risk to the subject/patient. In this case, it is not recommended to conduct a re-provocation test unless the continued use of the study drug is beneficial to the patient, and there is no alternative treatment available, and the test can only be performed with the approval of the sponsor's clinical director.
	Consistent with research treatment characteristics	Are the clinical/pathological manifestations of the adverse event consistent with the previous data records of the study drug or the pharmacological and toxicological trials of such drugs?
A medically qualified investigator should report the evaluation results of the relationship between the study drug and AE in the case report form/worksheet based on his/her best clinical judgment, including consideration of the above factors.		
Causality record	Investigators should evaluate the possible correlation between adverse events and study drugs with reference to the following five criteria: 1) The occurrence of adverse events is consistent with the time of drug use; 2) The adverse events are related to the known adverse reactions of the study drugs; 3) Adverse events cannot be explained in other ways; 4) Adverse reactions disappear after drug withdrawal; 5) Adverse events recur after administration.<br>The result will be judged as "definitely relevant", "probably relevant", "may be relevant", "may not be relevant", "not relevant". The three results, which are determined to be related, likely to be related, and possibly related, are determined as adverse reactions, and the incidence of adverse events is calculated based on this. For details, see 5.2.4.	

Abbreviations

Phrases and terminology	Explanation
AE	Adverse event
AJCC	American Journal of Critical Care
ALT	Alanine aminotransferase
ANC	Absolute neutrophil count
APTT	Activated partial thromboplastin time
AST	Aspartate aminotransferase
BCRP	Blood C reactive protein
BP	Blood pressure
BUS	Bultrasound
CCr	Creatinine clearance rate
CI	Confidence interval
CNS	Central nervous system
CR	Complete response
CRF	Case Report Form
CT	Computed tomography
CTCAE	Common Terminology Criteria for Adverse Events
ctDNA	Circulating tumor DNA
CTEP	Cancer Therapy Evaluation Program
DCR	Disease control rate
DLT	Dose-limiting toxicity
DOR	Duration of remission
ECG	Electrocardiogram
ECOG	Eastern Cooperative Oncology Group Performance Status
EGFR	Epidermal growth factor receptor
EPO	Erythropoietin
FAS	Full analysis set
FBG	Fasting blood glucose
Fbg	Fibrinogen
FDG	Fluorodeoxyglucose
FFPE	Formalin fixed paraffin embedded
FISH	Fluorescent in situ hybridization
GCP	Good Clinical Practice
GMP	Good Manufacturing Practices
HIV	Human immunodeficiency virus
HRCT	High-resolution computed tomography
ICF	International classification of functioning
ICH	International Conference on Harmonization
IEC	Independent Ethics Committee

IHC	Immunohistochemistry
ILD	Interstitial lung disease
INR	International Normalized Ratio
IRB	Institutional Review Board
LDH	Lactate Dehydrogenase
LVEF	Left ventricular ejection fraction
MTD	Maximum tolerated dose
MRI	Magnetic resonance imaging
MUGA	Multiple uptake gated acquisition
NCI	National Cancer Institute
NGS	Next generation sequencing
NSCLC	Non-small cell lung cancer
NTL	Non-target lesion
ORR	Objective response rate
OS	Overall survival
PD	Progression disease
PET-CT	Positron emission tomography computed tomography
PFS	Progression free survival
Pgp	P-glycoprotein
PPS	Per protocol set
PR	Partial response
PT	Prothrombin time
QD	*Quaque die*
RBC	Red blood cell
RECIST	Response Evaluation Criteria in Solid Tumors
RP2D	Recommended phase 2 dose
RTK	Receptor tyrosine kinase
SAE	Serious adverse event
SAS	Safety analysis set
SD	Stable disease
SOC	System organ category
SRC	Scientific review committee
T3	S-Triiodothyronine
T4	S-thyroxine
TL	Target lesion
TKI	Tyrosine kinase inhibitor
TNM	Tumor Node Metastasis
TSH	Thyroid Stimulating Hormone
TT	Thrombin time
UICC	Union for International Cancer Control
ULN	Upper limit of normal
WHO	World Health Organization

Statistical analysis plan

Sponsor: Shanghai Chest Hospital, Shanghai Jiao Tong University School of Medicine/ Chia Tai Tianqing Pharmaceutical Group Co, Ltd/ Novartis

Clinical approval No.: IS2117 (version 1.0)

Protocol No.: ATRAS-LC-1.0

Version No. and Data: 1.0/2021.02.21

Purpose

The statistical analysis plan (SAP) describes the statistical methods to be used during the reporting and analyses of data collected under Shanghai Chest Hospital, Shanghai Jiao Tong University School of Medicine. In particular, this statistical analysis plan 1.0 (SAP1.0) is relevant to Phase 1 (including part A and part B) of the protocol.

Scope

Study objectives and endpoints

Study design

Analysis population

Endpoint and variable definitions

Data handling

Data review

Statistical methos

Introduction

The study is a Phase I clinical trial of trametinib plus anlotinib in non-small cell lung cancer (NSCLC) patients with non-G12C KRAS mutations. The SAP will be divided into separate modules (part A and part B). Part A sought to define the recommended phase II dose (RP2D), and the part B sought to evaluate the objective response rate (ORR).

Study objectives and endpoints

1. Objectives

1.1 Phase I clinical trial

To evaluate the safety and efficacy of trametinib plus anlotinib in advanced NSCLC patients with non-G12C KRAS mutations.

1.2 Part A of phase I clinical trial

To define the RP2D and safety of trametinib plus anlotinib in the NSCLC patients with non-G12C KRAS mutations.

1.3 Part B of phase I clinical trial

To evaluate the therapeutic efficacy of trametinib plus anlotinib in the NSCLC patients with non-G12C KRAS mutations.

2. Endpoints

2.1 Part A

2.1.1 Primary endpoint: To evaluate the ORR and define the RP2D of the combined strategy of trametinib and anlotinib for advanced NSCLC patients with KRAS mutation (excluded $KRAS^{G12C}$).

2.1.2 Secondary endpoint: To evaluate the PFS, OS and safety of the combined strategy of trametinib and anlotinib for advanced NSCLC patients with KRAS mutation (excluded $KRAS^{G12C}$).

2.2 Part B

2.2.1 Primary endpoint: To evaluate the ORR of the combined strategy of trametinib and anlotinib for advanced NSCLC patients with KRAS mutation (excluded $KRAS^{G12C}$).

2.2.2 Secondary endpoint: To evaluate the PFS, OS and safety of the combined strategy of trametinib and anlotinib for advanced NSCLC patients with KRAS mutation (excluded $KRAS^{G12C}$).

Exploratory endpoint: dynamic changes of biomarkers and mutation profiles related to the efficacy and drug resistance of trametinib combined with anlotinib.

Study design

This is an open label, single-center, phase I, two parts (Part A and Part B) cohort trial evaluating the safety, RP2D, and clinical therapeutic efficacy of trametinib plus anlotinib in advanced NSCLC patients with non-G12C KRAS mutations.

Part A is the dose escalation segment of the study. Part B is the expansion cohorts are implemented to ensure sufficient therapeutic efficacy and safety.

The enrolled NSCLC patients with non-G12C KRAS mutations will receive study regime at the discretion of the Investigator until disease progression, unacceptable adverse events, patient refusal or death. Patients experiencing clinical benefit in the judgment of the Investigator may continue study treatment beyond disease progression as defined by RECIST 1.1. Patients discontinuing treatment will be followed for receipt of subsequent anti-cancer therapies and survival.

Sample size considerations

Part A will enroll advanced NSCLC patients with non-G12C mutations who have previously received standard treatment or treatment naïve. The primary endpoint of this study was to determine RP2D based on the incidence of DLT in cycle 1. For the dose-escalation stage (Part A), "3 + 3" principle was used. Briefly, trametinib is set to a single dose (2mg QD, oral) throughout the part A. Anlotinib is set to three levels of dose (8mg, 10mg, 12mg, once a day, from day 1 to day 14 of the 21-day cycle). Three patients will be enrolled in primary combination treatment (trametinib, 2mg QD, oral; anlotinib, 8mg, once a day, from day 1 to day 14 of the 21-day cycle). If one patient occurs DLT in cycle 1, another 3 patients will be enrolled to further verification the safety. If two or more patients occur DLT in cycle 1, it is suggested that the exploration can be carried out in a lower dose group. If no patient occurs DLT in cycle 1, next dose combination treatment will be performed. The sponsor and investigators will review the safety and clinical data of all subjects to jointly determine RP2D of anlotinib combined with trametinib.

Part B will enroll advanced NSCLC patients with non-G12C mutations who have previously received standard treatment. If RP2D is reached in Part A, another 20 eligible patients will be enrolled and treated with trametinib (2mg QD, oral) + anlotinib (RP2D, once a day, from day 1 to day 14 of the 21-day cycle), until the PD or unacceptable toxicity occurs to further evaluate the safety, tolerability and efficacy. The primary endpoint for evaluation of efficacy is ORR. The standard-of-care for advanced NSCLC patients with non-G12C KRAS mutations treated in this setting is docetaxel, which is associated with an ORR of up to 23%. Assuming trametinib plus anlotinib will result in an ORR of at the range of 55% in this setting, a sample size of approximately 20 evaluable patients would be sufficient for the lower bound of a 2-sided 95% confidence interval (Clopper-Pearson method) to exclude an ORR of 23%.

Analysis populations

1.1 Pre-screening population

The pre-screening population is defined as all advanced NSCLC patients for whom a tumor and/or blood sample is tested by professional institute for KRAS mutation.

1.2 Enrolled population

The enrolled population is defined as all advanced NSCLC patients with non-G12C KRAS mutations who sign the main study informed consent form and determined by the Investigator to meet all eligibility criteria during screening assessments.

1.3 Safety population

The safety population is defined as all NSCLC patients with non-G12C KRAS mutations who received at least 1 dose of study medication. The Safety population will be used for all safety analyses.

1.4 Clinical efficacy evaluable population

Advanced NSCLC patients included in the clinical efficacy evaluable population are patients who receive at least one dose of study medication and have an evaluable baseline tumor assessment and at least on post-baseline tumor assessment. The clinical efficacy evaluable population will be used in integrative analysis of this study.

1.5 Full analysis set

The full analysis set (FAS) is defined as all advanced NSCLC patients with non-G12C KRAS mutations who receive at least one dose of trametinib plus anlotinib on this study. The FAS will be used in the primary analyses for efficacy endpoints.

Data handling

1. Imputation of missing dates

The following rules will be applied to impute missing start and stop dates in appropriate data types.

Start date

If the start date is completely missing (i.e., the day, month, and year are all unknown), the start date will be set to the date of first dose of study medication.

Missing Day Only

• If the month and year of the incomplete date are the same as the month and year of the first dose date, then the day of the first dose date will be assigned to the missing day.

• If either the year is before the year of the first dose date or if years are the same but the month is before the month of the first dose date, then the last day of the month will be assigned to the missing day.

• If either the year is after the year of the first dose date or if both years are the same but the month is after the month of the first dose date, then the first day of the month will be assigned to the missing day.

Missing Month Only

• The day will be treated as also missing and both month and day will be replaced according to the below procedure.

Missing Day and Month

• If the year of the incomplete date is the same as the year of the first dose date, then the day and month of the first dose date will be assigned to the missing fields.

• If the year of the incomplete date is not the same as the year of the first dose date, then January 1 will be assigned to the missing fields.

• If the stop date is complete and the imputed start date is after the stop date, then the start date will be imputed using the stop date.

Stop date

Missing Day Only

• If the month and year of the incomplete date are the same as the month and year of the last visit date, then the day of the last visit date will be assigned to the missing day.

• If either the year is before the year of the last visit date or if both years are the same but the month is before the month of the last visit date, then the last day of the month will be assigned to the missing day.

• If either the year is after the year of the last visit date or if both years are the same but the month is after the month of the last visit date, then the first day of the month will be assigned to the missing day.

Missing Month Only

• The day will be treated as missing and both month and day will be replaced according to the below procedure.

Missing Day and Month

• If the year of the incomplete date is the same as the year of the last visit date, then the day and month of the last visit date will be assigned to the missing fields.

• If the year of the incomplete date is before the year of the last visit date, then December 31 will be assigned to the missing fields.

• If the year of the incomplete date is after the year of the last visit date, then last visit date will be assigned to the missing fields.

No imputation will be performed for medications with completely missing start dates.

Medications with completely missing start dates will be listed in both prior medications and con-commitment medication.

2. Imputation of diagnosis and prior disease history

The partial start date for diagnosis of metastatic disease and prior disease history will be assigned to 15th day of the month (if only day is missing) or July 1st (if both month and day are missing).

3. Imputation of laboratory values with character symbol

Missing laboratory data will not be imputed. However, laboratory values of the form of "< x" (i.e., below the lower limit of quantification) or "> x" (i.e., above the upper limit of quantification) will be imputed as "x" for the purpose of calculation of summary statistics and comparing to normal ranges. These values will still be displayed as "< x" or "> x" in the listings.

Statistical methods

The primary endpoint of this study was to determine RP2D based on the incidence of DLT in cycle 1. The specific required sample size will be based on the dose-escalation design of the "3+3" principle, and the DLT situation of each dose group observed during the actual dose escalation process. For the dose expansion phase, it is planned to enroll 20 patients to further clarify the toxicity, tolerability, and efficacy.

All data will be provided for the FAS. All advanced NSCLC patients with non-G12C KRAS mutations who received at least one dose of study drug will be included in the study analysis. For all variables, descriptive statistics will be performed as appropriate. Continuous variables will be summarized by observations, mean, standard deviation, median, minimum, and maximum. Categorical variables will be aggregated by frequency counts and percentages for each category.

The FAS includes all patients who received at least one dose of study drug. The FAS will be used for all efficacy and safety analysis.

The PPS is a subset of FAS, including all cases that meet the research protocol, has good compliance, does not use any prohibited drugs during the research process. The set of compliance protocols will be used for supporting analysis of effectiveness endpoints.

1. **Safety and tolerability**

The safety analysis set will include all patients who have received at least one dose of the therapeutic drug. The patient will be evaluated based on the actual treatment received. The safety and tolerability summary will be based on the safety analysis set. AE will be coded according to the preferred term of SOC and MedDRA terminology. The severity of AE will be graded according to NCI CTCAE version 5.0. A list of all AEs will be listed, including detailed information collected for each AE. (Description of event, date/time of onset, duration, severity, severity, relationship with study drug, measures taken, clinical outcome).

2. **Tumor response**

Tumor response data will be listed and summarized by dose group, and the following response categories will be used when appropriate: CR, PR, SD, PD, and NE. For the definitions of CR, PR, SD and PD, please refer to the RECISIT version 1.1 standard. Where appropriate, the objective tumor response rate is given with a 95% confidence interval (calculated using the Clopper-Pearson interval).

3. **Duration of response**

For the escalation cohort and the expansion cohort, summarize the duration of response of patients, and count the number of patients (%) with remission duration >3; >6; >9; >12 months, draw the Kaplan-Meier curve of median response duration.

4. **PFS and OS**

PFS will be summarized in the expansion phase.

PFS will be displayed using Kaplan-Meier curves. The number of events, the median (calculated based on the Kaplan-Meier curve), and the proportion of patients with no events at 6, 12, and 18 months will be summarized.

Where appropriate, a summary of the number and percentage of patients who died, are still under follow-up, lost to follow-up, and withdrawn from the study will be provided.

---

5. Biomarkers and mutation profiles

All patients participating in the biomarker study will be included in the analysis set.

Protocol deviations

Protocol deviations noted during clinical monitoring will be documented by category (i.e., inclusion and exclusion criteria, study drug administration, study procedures and assessments, study visit schedule, informed consent, and other). All deviations will be reviewed, categorized, designated important or not important. Important protocol deviations will be defined as those potentially impacting safety or efficacy assessments and analyses. Additional details of what will be considered important can be found in the Protocol Deviation Guidance document. Important protocol deviations for patients in the Enrolled population will be summarized by category. Important protocol deviations will be listed.

Demographic and baseline characteristics

Demographic and baseline data will be collected and summarized for the enrolled patients.

Demographic characteristics included gender, age, smoking history, baseline Eastern Cooperative Oncology Group (ECOG) status, KRAS mutation types, pathological types, and metastatic status. Age is calculated from date of informed consent to date

of birth.
Data will be listed by patient.

Prior and concomitant medications

Prior and Concomitant medications will be coded using World Health Organization (WHO) Drug Enhanced (version: March 2018).

Censoring rules for time-to-event endpoints based on radiographic evaluations

According to the RECIST 1.1 standard, all subjects will be monitored by radiological evaluation every 4-8 weeks to determine changes in tumor size. After the investigators' evaluation, the assessment cycle can extend to 12 weeks or longer due to the uncontrollable factors are also be accepted during the treatment period.

Safety analyses

The principal investigator is responsible for ensuring that all personnel involved in the research are familiar with the content of this section.

1. Definition of adverse events

AEs refer to adverse medical conditions or deterioration of the original medical conditions after or during exposure to the study drugs, regardless of whether there is a causal relationship with the drug. A bad medical condition may be symptoms (for example, nausea, chest pain), physical signs (for example, tachycardia, enlarged liver), or abnormal test results (for example, laboratory test results, electrocardiogram). In clinical studies, AEs include adverse medical conditions that occur at any time, including the lead-in period or wash-out period, even if no research treatment has been performed.
The term AE includes serious and non-serious AEs.

2. Definition of serious adverse events

SAE refer to AEs that occur during any research phase (i.e., lead-in period, treatment period, washout period, follow-up period), which meet one or more of the following criteria:

Causes death

Immediate life threatening

Need to be hospitalized or extend the current hospital stay

Cause permanent or severe disability/loss of function or severely disrupt normal life functions

Congenital malformations or birth defects

It is a major medical event that may endanger the subject or may require medical intervention to prevent one of the above outcomes

3. Adverse Event Record

Time period for collecting adverse events

AEs and SAEs will be collected from the time of informed consent throughout the treatment period, including the follow-up period (90 days after the last study drug administration).

Follow-up of unresolved adverse events

In the study, the investigator will follow up any AEs that the subject has not resolved in the last AE assessment based on medical indications, but no further records are required in the CRF.

4. Variables

The severity of AE is classified according to NCI CTCAE 5.0, using a 5-point scale (1-5 grades), and is reported in detail on CRF. The grades of AEs not included in

CTCAE are as follows:

CTCAE grade	Equal:	definition
Level 1	Mild	Feel uncomfortable and do not interfere with normal activities of daily life.
Level 2	Moderate	Feeling of discomfort is sufficient to reduce or affect activities of daily living; treatment or medical intervention is not indicated, although these measures can improve the patient's overall health or symptoms.
Level 3	Severe	Inability to work or normal activities of daily life; treatment or medical intervention is required to improve the patient's overall health or symptoms; delay in treatment has no direct harmful effect on the patient's survival.
Level 4	Life-threatening or disabling	Directly endanger life or cause permanent mental or physical illness, affecting work or normal activities of daily life; treatment or medical intervention is required to maintain life.
Level 5	Fatal	AE causes death

5. Causality collection

The investigator will evaluate the causal relationship between the trial drug and each AE, and answer "yes" or "no" to the question "Do you think the event may be caused by the trial drug?"

For SAE, the causal relationship between other drugs and the research process will also be evaluated. Please note that for SAEs that may be related to any research process, causality is "yes".

For guidance on the interpretation of causality issues, please see Appendix A of the "Clinical Research Protocol".

6. Adverse events based on signs and symptoms

All AEs reported by the subject spontaneously or when answering open-ended questions from the researcher: "Did you have any health problems since the last visit/last interview?" or found through observation. The information will be collected and recorded in the CRF. When collecting AEs, it is best to record the diagnosis, not

the signs and symptoms. However, if the diagnosis result is known and there are other signs or symptoms that are not normally included in the diagnosis, the diagnosis and each sign or symptom will be recorded separately.

7. Adverse events based on inspections and trials

The results of laboratory tests and vital signs specified in the protocol will be summarized in the clinical study report. Therefore, compared to the baseline set by the protocol, if the deterioration meets any SAE criteria or the reason for discontinuation of the trial drug treatment, it should only be reported as an AE.

If the deterioration of laboratory test values/vital signs is related to clinical signs and symptoms, the signs or symptoms will be reported as an AE, and the related laboratory results/vital signs will be treated as other information. Investigators use clinical terms as much as possible for reporting, rather than laboratory terms (for example, anemia and low hemoglobin values). In the absence of clinical signs or symptoms, clinically relevant deterioration of non-mandatory parameters should be reported as an AE. Deterioration of laboratory test values clearly due to disease progression should not be reported as AE/SAE.

Compared with the baseline assessment, any new or aggravated clinically relevant abnormal medical results found during the physical examination will be reported as an AE.

8. Disease progression

Disease progression can be considered to be the deterioration of the subject's disease caused by the disease under investigation by the experimental drug. It may be an increase in the severity of the disease under study and/or an increase in disease symptoms. New metastasis or progression to existing metastases should be considered disease progression, not AEs. Events that are clearly due to disease progression should not be reported as AEs during the study period.

The progression of the malignant tumor in the study, including the progression of

signs and symptoms, should not be reported as a serious AE. Hospitalization due to signs and symptoms of disease progression should not be reported as a serious AE.

9. Report of serious adverse events

The investigator is responsible for notifying the ethics committee and sponsor of any SAE.

The investigator must inform the local ethics committee of any SAE within 24 hours.

All SAEs that lack important or relevant information should be followed up immediately. The investigator should inform the sponsor representative of any follow-up information of previously reported SAEs within 24 hours after learning.

Laboratory safety assessment

The laboratory safety assessment parameters are listed in the table:

Clinical chemistry	Hematology
Serum (S) / Plasma (P)-Albumin	Blood (B)-hemoglobin
S/P-ALT	B-leukocytes
S/P-AST	B-hematocrit
S/P-Alkaline Phosphatase	B-Red blood cell (RBC) count
S/P-total bilirubin	B-Absolute white blood cell classification count:
S/P-total calcium	Neutrophils
S/P-creatinine	Lymphocytes
S/P-glucose (fasting only on PK days)	Monocyte
S/P-Lactate Dehydrogenase (LDH) 2	Basophils
S/P-magnesium	Eosinophils
S/P-potassium	B-platelet count
S/P-Sodium	B-reticulocyte
S/P-urea nitrogen or blood urea nitrogen	Urinalysis
S/P-lipase	U-glucose
S/P-Amylase	U-protein
S/P-total cholesterol	U-blood
S/P-total triglycerides	Coagulation
S/P- High Density Lipoprotein	P-prothrombin time (PT)
S/P- Low Density Lipoprotein	P-activated partial thromboplastin time (APTT)
Thyroid function	P-thrombin time (TT)
S-Triiodothyronine (T3)	P-fibrinogen (Fbg)
S-thyroxine (T4)	P-D-dimer
S-TSH	P- INR
S-free T3	
S-free T4	

Blood and urine samples will be collected at the time specified in the study plan to determine clinical chemistry, hematology, thyroid function, coagulation function, and

urinalysis. If the researcher believes that there are clinical indications, other safety samples can be collected. Acquisition date, time and results (value, unit and reference range) will be recorded in the appropriate CRF.

Clinical chemistry, hematology, thyroid function, coagulation function and urinalysis will be performed in Shanghai Chest Hospital, Shanghai Jiao Tong University School of Medicine.

In addition, during the screening visit, pregnancy tests are only performed on women with reproductive potential (according to the standard clinical practice of the research center, blood or urine tests are acceptable).

Researchers should evaluate existing results regarding clinically relevant abnormalities. Laboratory results should be signed and dated, and stored in the research center as the source data of laboratory variables.

Electrolyte abnormalities (hypokalemia, hypomagnesemia, hypocalcemia) must be corrected to within the normal range before the first dose, and electrolyte levels should be monitored during the study treatment.

Physical examination

A physical examination will be performed, including evaluation of the following: general appearance, skin, head and neck (including ears, eyes, nose, and throat), respiratory system, cardiovascular, abdomen, lymph nodes, thyroid, abdomen, and central nervous system.

Electrocardiogram

All patients will undergo a 12-lead digital electrocardiogram during the study visit. After the patient rests on the supine side for at least 10 minutes before the specified time, a 12-lead ECG will be obtained, which should be recorded at a speed of 25 mm/sec. All ECGs should be recorded in the same position of the patient. For each time point, three ECG results should be recorded in approximately 5 minutes. The digital ECG records will be collected, analyzed and stored by the central ECG supplier. If there is an abnormality in the evaluation during the treatment when the

study treatment is stopped, a 28-day follow-up evaluation is required to confirm the reversibility of the abnormality.

Echocardiography/MUGA Scan

Echocardiography or MUGA scans will be performed at screening (before the first treatment) and at least every 16 weeks throughout the treatment period to assess LVEF. The method of cardiac function assessment must be consistent within the patient (i.e., if echocardiography is used for screening assessment, echocardiography should also be used for subsequent scans). As far as possible, the same machine and operator should be used to examine patients and perform quantitative measurements. If there is an abnormality in the evaluation during the treatment when the study treatment is stopped, a 28-day follow-up evaluation is required to confirm the reversibility of the abnormality.

Vital signs

Pulse and BP

After resting for 10 minutes, BP and pulse rate in the supine position will be measured. As indicated in the research plan, evaluation will be performed at the time of the visit. In addition, if there are clinical indications, it should be at the discretion of the investigator. If applicable, any change in vital signs should be recorded as an AE.

Weight and height

Body weight assessment will be performed at the time of screening, and then on day 1 of each cycle and at the discontinuation visit. Evaluate height only during screening.

Summary of changes for statistical analysis plan

Document	Version date	Summary of changes
Original protocol, Version 1.0	2021.02.21	N/A
Amendment 1, Version 3.0	2022.05.27	1. Update the enrolled patients' requirement for Part B: "advanced NSCLC patients with non-G12C mutations who have previously received standard 1st or more treatment".<br>2. Update the dosage design in part A. Add a group of anlotinib (6mg) plus trametinib (2mg). The combined doses of trametinib (2mg, QD) and anlotinib (6mg, 8mg, 10mg, 12mg, QD, the first day to the 14th day of the 21-day cycle) respectively.<br>3. Update the "Calculation of sample size". Part A: "Part A will enroll advanced NSCLC patients with non-G12C mutations who have previously received standard treatment or treatment naïve. The primary endpoint of this study was to determine RP2D based on the incidence of DLT in cycle 1. For the dose-escalation stage (Part A), "3 + 3" principle was used. Briefly, trametinib is set to a single dose (2mg QD, oral) throughout the part A. Anlotinib is set to four levels of dose (6mg, 8mg, 10mg, 12mg, once a day, from day 1 to day 14 of the 21-day cycle). Three patients will be enrolled in primary combination treatment (trametinib, 2mg QD, oral; anlotinib, 6mg, once a day, from day 1 to day 14 of the 21-day cycle). If one patient occurs

		DLT in cycle 1, another 3 patients will be enrolled to further verification the safety. If two or more patients occur DLT in cycle 1, it is suggested that the exploration can be carried out in a lower dose group. If no patient occurs DLT in cycle 1, next dose combination treatment will be performed. If two patients screened and enrolled in a same dose group simultaneously, four patients in a single dose group are allowable. The sponsor and investigators will review the safety and clinical data of all subjects to jointly determine RP2D of anlotinib combined with trametinib”.<br>Part B: “Part B will enroll advanced NSCLC patients with non-G12C mutations who have previously received standard treatment. If RP2D is reached in Part A, another 20 eligible patients will be enrolled and treated with trametinib (2mg QD, oral) + anlotinib (RP2D, once a day, from day 1 to day 14 of the 21-day cycle), until the PD or unacceptable toxicity occurs to further evaluate the safety, tolerability and efficacy. The primary endpoint for evaluation of efficacy is ORR. The standard-of-care for advanced NSCLC patients with non-G12C KRAS mutations treated in this setting is docetaxel, which is associated with an ORR of up to 23%. Assuming trametinib plus anlotinib will result in an ORR of at least 55% in this setting, a sample size of approximately 20 evaluable patients would be sufficient for the lower bound of a 2-sided 95% confidence interval

		(Clopper-Pearson method) to exclude an ORR of 23%. If an ORR of at lower 40%, the part B study will be terminated".<br>4. Add the effects of COVID-19 on the clinical trial. Add: "For the patients who discontinued the therapy due to COVID-19 infection or COVID-19-related reasons, they will continue to receive trametinib plus anlotinib therapy if the investigators think the patients still can receive benefit from the medication".
Amendment 2, Version 4.0	2022.05.09	1. Delete the PPS-related analysis in the statis methods section.<br>2. Re-define the tumor response, duration of response, and progression-free survival.<br>5. Add integrative analyses: "For the patients enrolled in phase I clinical study (including part A and part B), the clinical characteristics, ORR, PFS, and AEs will be analyzed".<br>6. Add subgroup analyses: "According to the mutation types of non-G12C KRAS, the FAS will be divided into KRASG12V subtype, KRASG12D subtype, and KRASother subtype. ORR and PFS will be calculated based on the different KRAS mutation subtypes. In addition, tumor shrinkage (percent change from baseline) will be presented for different KRAS mutation subtypes".

Statistical analysis plan

Sponsor: Shanghai Chest Hospital, Shanghai Jiao Tong University School of Medicine/ Chia Tai Tianqing Pharmaceutical Group Co, Ltd/ Novartis

Clinical approval No.: IS2117 (Version 4.0)

Protocol No.: ATRAS-LC-4.0

Version No. and Data: 4.0/2023.05.09

Purpose

The statistical analysis plan (SAP) describes the statistical methods to be used during the reporting and analyses of data collected under Shanghai Chest Hospital, Shanghai Jiao Tong University School of Medicine. In particular, this statistical analysis plan 1.0 (SAP1.0) is relevant to Phase 1 (including part A and part B) of the protocol.

Scope

Study objectives and endpoints

Study design

Analysis population

Endpoint and variable definitions

Data handling

Data review

Statistical methos

Introduction

The study is a Phase I clinical trial of trametinib plus anlotinib in non-small cell lung cancer (NSCLC) patients with non-G12C KRAS mutations. The SAP will be divided into separate modules (part A and part B). Part A sought to define the recommended phase II dose (RP2D), and the part B sought to evaluate the objective response rate (ORR).

Study objectives and endpoints

1. Objectives

1.1 Phase I clinical trial

To evaluate the safety and efficacy of trametinib plus anlotinib in advanced NSCLC patients with non-G12C KRAS mutations.

1.2 Part A of phase I clinical trial

To define the RP2D and safety of trametinib plus anlotinib in the NSCLC patients with non-G12C KRAS mutations.

1.3 Part B of phase I clinical trial

To evaluate the therapeutic efficacy of trametinib plus anlotinib in the NSCLC patients with non-G12C KRAS mutations.

2. Endpoints

2.1 Part A

2.1.1 Primary endpoint: To evaluate the ORR and define the RP2D of the combined strategy of trametinib and anlotinib for advanced NSCLC patients with KRAS mutation (excluded KRAS^{G12C}).

2.1.2 Secondary endpoint: To evaluate the PFS, OS and safety of the combined strategy of trametinib and anlotinib for advanced NSCLC patients with KRAS mutation (excluded KRAS^{G12C}).

2.2 Part B

2.2.1 Primary endpoint: To evaluate the ORR of the combined strategy of trametinib and anlotinib for advanced NSCLC patients with KRAS mutation (excluded KRAS^{G12C}) who previous had been received 1st or more lines standard therapy.

2.2.2 Secondary endpoint: To evaluate the PFS, OS and safety of the combined strategy of trametinib and anlotinib for advanced NSCLC patients with KRAS mutation (excluded KRAS^{G12C}) who previous had been received 1st or more lines standard therapy.

Exploratory endpoint: dynamic changes of biomarkers and mutation profiles related to the efficacy and drug resistance of trametinib combined with anlotinib

Study design

This is an open label, single-center, phase I, two parts (Part A and Part B) cohort trial evaluating the safety, RP2D, and clinical therapeutic efficacy of trametinib plus anlotinib in advanced NSCLC patients with non-G12C KRAS mutations.

Part A is the dose escalation segment of the study. Part B is the expansion cohorts are implemented to ensure sufficient therapeutic efficacy and safety.

The enrolled NSCLC patients with non-G12C KRAS mutations will receive study regime at the discretion of the Investigator until disease progression, unacceptable adverse events, patient refusal or death. Patients experiencing clinical benefit in the judgment of the Investigator may continue study treatment beyond disease progression as defined by RECIST 1.1. Patients discontinuing treatment will be followed for receipt of subsequent anti-cancer therapies and survival.

Sample size considerations

Part A will enroll advanced NSCLC patients with non-G12C mutations who have previously received standard treatment or treatment naïve. The primary endpoint of this study was to determine RP2D based on the incidence of DLT in cycle 1. For the dose-escalation stage (Part A), "3 + 3" principle was used. Briefly, trametinib is set to a single dose (2mg QD, oral) throughout the part A. Anlotinib is set to four levels of dose (6mg, 8mg, 10mg, 12mg, once a day, from day 1 to day 14 of the 21-day cycle). Three patients will be enrolled in primary combination treatment (trametinib, 2mg QD, oral; anlotinib, 6mg, once a day, from day 1 to day 14 of the 21-day cycle). If one patient occurs DLT in cycle 1, another 3 patients will be enrolled to further verification the safety. If two or more patients occur DLT in cycle 1, it is suggested that the exploration can be carried out in a lower dose group. If no patient occurs DLT in cycle 1, next dose combination treatment will be performed. If two patients screened and enrolled in a same dose group simultaneously, four patients in a single

dose group are allowable. The sponsor and investigators will review the safety and clinical data of all subjects to jointly determine RP2D of anlotinib combined with trametinib.

Part B will enroll advanced NSCLC patients with non-G12C mutations who have previously received standard treatment. If RP2D is reached in Part A, another 20 eligible patients will be enrolled and treated with trametinib (2mg QD, oral) + anlotinib (RP2D, once a day, from day 1 to day 14 of the 21-day cycle), until the PD or unacceptable toxicity occurs to further evaluate the safety, tolerability and efficacy. The primary endpoint for evaluation of efficacy is ORR. The standard-of-care for advanced NSCLC patients with non-G12C KRAS mutations treated in this setting is docetaxel, which is associated with an ORR of up to 23%. Assuming trametinib plus anlotinib will result in an ORR of at least 55% in this setting, a sample size of approximately 20 evaluable patients would be sufficient for the lower bound of a 2-sided 95% confidence interval (Clopper-Pearson method) to exclude an ORR of 23%. If an ORR of at lower 40%, the part B study will be terminated.

Analysis populations

1.1 Pre-screening population

The pre-screening population is defined as all advanced NSCLC patients for whom a tumor and/or blood sample is tested by professional institute for KRAS mutation.

1.2 Enrolled population

The enrolled population is defined as all advanced NSCLC patients with non-G12C KRAS mutations who sign the main study informed consent form and determined by the Investigator to meet all eligibility criteria during screening assessments.

1.3 Safety population

The safety population is defined as all NSCLC patients with non-G12C KRAS

mutations who received at least 1 dose of study medication. The Safety population will be used for all safety analyses.

1.4 Clinical efficacy evaluable population

Advanced NSCLC patients included in the clinical efficacy evaluable population are patients who receive at least one dose of study medication and have an evaluable baseline tumor assessment and at least on post-baseline tumor assessment. The clinical efficacy evaluable population will be used in integrative analysis of this study.

1.5 Full analysis set

The full analysis set (FAS) is defined as all advanced NSCLC patients with non-G12C KRAS mutations who receive at least one dose of trametinib plus anlotinib on this study. The FAS will be used in the primary analyses for efficacy endpoints.

Data handling

1. Imputation of missing dates

The following rules will be applied to impute missing start and stop dates in appropriate data types.

Start date

If the start date is completely missing (i.e., the day, month, and year are all unknown), the start date will be set to the date of first dose of study medication.

Missing Day Only

• If the month and year of the incomplete date are the same as the month and year of the first dose date, then the day of the first dose date will be assigned to the missing day.

• If either the year is before the year of the first dose date or if years are the same but the month is before the month of the first dose date, then the last day of the month will be assigned to the missing day.

• If either the year is after the year of the first dose date or if both years are the same

but the month is after the month of the first dose date, then the first day of the month will be assigned to the missing day.

Missing Month Only

• The day will be treated as also missing and both month and day will be replaced according to the below procedure.

Missing Day and Month

• If the year of the incomplete date is the same as the year of the first dose date, then the day and month of the first dose date will be assigned to the missing fields.

• If the year of the incomplete date is not the same as the year of the first dose date, then January 1 will be assigned to the missing fields.

• If the stop date is complete and the imputed start date is after the stop date, then the start date will be imputed using the stop date.

Stop date

Missing Day Only

• If the month and year of the incomplete date are the same as the month and year of the last visit date, then the day of the last visit date will be assigned to the missing day.

• If either the year is before the year of the last visit date or if both years are the same but the month is before the month of the last visit date, then the last day of the month will be assigned to the missing day.

• If either the year is after the year of the last visit date or if both years are the same but the month is after the month of the last visit date, then the first day of the month will be assigned to the missing day.

Missing Month Only

• The day will be treated as missing and both month and day will be replaced according to the below procedure.

Missing Day and Month

• If the year of the incomplete date is the same as the year of the last visit date, then the day and month of the last visit date will be assigned to the missing fields.

• If the year of the incomplete date is before the year of the last visit date, then December 31 will be assigned to the missing fields.

• If the year of the incomplete date is after the year of the last visit date, then last visit date will be assigned to the missing fields.

No imputation will be performed for medications with completely missing start dates. Medications with completely missing start dates will be listed in both prior medications and con-commitment medication.

2. Imputation of diagnosis and prior disease history

The partial start date for diagnosis of metastatic disease and prior disease history will be assigned to 15th day of the month (if only day is missing) or July 1st (if both month and day are missing).

3. Imputation of laboratory values with character symbol

Missing laboratory data will not be imputed. However, laboratory values of the form of “< x” (i.e., below the lower limit of quantification) or “> x” (i.e., above the upper limit of quantification) will be imputed as “x” for the purpose of calculation of summary statistics and comparing to normal ranges. These values will still be displayed as “< x” or “> x” in the listings.

Statistical methods

The primary endpoint of this study was to determine RP2D based on the incidence of DLT in cycle 1. The specific required sample size will be based on the dose-escalation design of the "3+3" principle, and the DLT situation of each dose group observed during the actual dose escalation process. For the dose expansion phase, it is planned to enroll 20 patients to further clarify the toxicity, tolerability, and efficacy.

All data will be provided for the FAS. All advanced NSCLC patients with non-G12C KRAS mutations who received at least one dose of study drug will be included in the study analysis. For all variables, descriptive statistics will be performed as appropriate. Continuous variables will be summarized by observations, mean, standard deviation, median, minimum, and maximum. Categorical variables will be aggregated by frequency counts and percentages for each category.

The FAS includes all patients who received at least one dose of study drug. The FAS will be used for all efficacy and safety analysis.

1. Safety and tolerability

The safety analysis set will include all patients who have received at least one dose of the therapeutic drug. The patient will be evaluated based on the actual treatment received. The safety and tolerability summary will be based on the safety analysis set.

AE will be coded according to the preferred term of SOC and MedDRA terminology. The severity of AE will be graded according to NCI CTCAE version 5.0. A list of all AEs will be listed, including detailed information collected for each AE. (Description of event, date/time of onset, duration, severity, severity, relationship with study drug, measures taken, clinical outcome).

2. Tumor response

Tumor response data will be listed and summarized by dose group, and the following response categories will be used when appropriate: CR, PR, SD, PD, and NE. For the definitions of CR, PR, SD and PD, please refer to the RECISIT version 1.1 standard. Where appropriate, the objective tumor response rate is given with a 95% confidence interval (calculated using the Clopper-Pearson interval).

Objective response rate (ORR) is defined as the percent of patients documented to have a confirmed CR or PR.

Descriptive statistics (frequency and percentage) for ORR based on response assessments by investigator. Patients who cannot be assessed for response will be counted as not evaluable. Descriptive statistics (frequency and percentage) for CR and PR rate will be presented overall.

3. Duration of response

Duration of Response (DOR) in months is defined as the time from date of the first documentation of objective response (CR or PR) to the first documentation of PD or

to death due to any cause in the absence of documented PD. DOR will only be calculated for the subgroup of patients achieving an efficacy of CR or PR. The response duration of the responding patients will be summarized, and the number of responding patients (%) for the duration of remission> 3; > 6; > 9; > 12 months will be given. The Kaplan Meier chart and the median duration of response with 95% confidence interval (95% CI, calculated based on the Kaplan Meier chart) will be given. DOR will be evaluated based on response assessments by the investigator.

For the patients who discontinued the therapy due to COVID-19 infection or COVID-19-related reasons, they will continue to receive trametinib plus anlotinib therapy if the investigators think the patients still can receive benefit from the medication.

4. PFS analyses

PFS is defined as the time from the date of first treatment to the date of first PD or death due to any cause in the absence of documented PD, whichever occurs first. PFS (in days or months) will be calculated.

The PFS of the part A, part B, overall, subgroups will be summarized. PFS will be displayed using Kaplan-Meier curves. The number of events, the median (calculated based on the Kaplan-Meier curve), and the proportion of patients with no events at 6, 12, and 18 months will be summarized. Where appropriate, a summary of the number and percentage of patients who have died, are still undergoing follow-up, lost follow-up, and dropped out of the study will be provided.

For the patients who discontinued the therapy due to COVID-19 infection or COVID-19-related reasons, they will continue to receive trametinib plus anlotinib therapy if the investigators think the patients still can receive benefit from the medication.

5. Integrative analyses

For the patients enrolled in phase I clinical study (including part A and part B), the clinical characteristics, ORR, PFS, and AEs will be analyzed.

6. Subgroup analyses

According to the mutation types of non-G12C KRAS, the FAS will be divided into KRAS^{G12V} subtype, KRAS^{G12D} subtype, and KRAS^{other} subtype. ORR and PFS will be calculated based on the different KRAS mutation subtypes. In addition, tumor shrinkage (percent change from baseline) will be presented for different KRAS mutation subtypes.

7. Biomarkers and mutation profiles

All patients participating in the biomarker study will be included in the analysis set.

Protocol deviations

Protocol deviations noted during clinical monitoring will be documented by category (i.e., inclusion and exclusion criteria, study drug administration, study procedures and assessments, study visit schedule, informed consent, and other). All deviations will be reviewed, categorized, designated important or not important. Important protocol deviations will be defined as those potentially impacting safety or efficacy assessments and analyses. Additional details of what will be considered important can be found in the Protocol Deviation Guidance document. Important protocol deviations for patients in the Enrolled population will be summarized by category. Important protocol deviations will be listed. Any COVID-19-specific protocol deviations will be listed separately. The number of patients missing visits due to the COVID-19 pandemic will be summarized. If the number of patients impacted by the COVID-19 pandemic is large (eg, >10%) and thought to be impacting the efficacy and safety endpoints, sensitivity analyses may be carried out.

Demographic and baseline characteristics

Demographic and baseline data will be collected and summarized for the enrolled patients.

Demographic characteristics included gender, age, smoking history, baseline Eastern

Cooperative Oncology Group (ECOG) status, KRAS mutation types, pathological types, and metastatic status. Age is calculated from date of informed consent to date of birth.
Data will be listed by patient.

Prior and concomitant medications

Prior and Concomitant medications will be coded using World Health Organization (WHO) Drug Enhanced (version: March 2018).

Censoring rules for time-to-event endpoints based on radiographic evaluations

According to the RECIST 1.1 standard, all subjects will be monitored by radiological evaluation every 4-8 weeks to determine changes in tumor size. After the investigators' evaluation, the assessment cycle can extend to 12 weeks or longer due to the uncontrollable factors are also be accepted during the treatment period.

Safety analyses

The principal investigator is responsible for ensuring that all personnel involved in the research are familiar with the content of this section.

1. Definition of adverse events

AEs refer to adverse medical conditions or deterioration of the original medical conditions after or during exposure to the study drugs, regardless of whether there is a causal relationship with the drug. A bad medical condition may be symptoms (for example, nausea, chest pain), physical signs (for example, tachycardia, enlarged liver), or abnormal test results (for example, laboratory test results, electrocardiogram). In clinical studies, AEs include adverse medical conditions that occur at any time, including the lead-in period or wash-out period, even if no research treatment has

been performed.

The term AE includes serious and non-serious AEs.

2. Definition of serious adverse events

SAE refer to AEs that occur during any research phase (i.e., lead-in period, treatment period, washout period, follow-up period), which meet one or more of the following criteria:

Causes death

Immediate life threatening

Need to be hospitalized or extend the current hospital stay

Cause permanent or severe disability/loss of function or severely disrupt normal life functions

Congenital malformations or birth defects

It is a major medical event that may endanger the subject or may require medical intervention to prevent one of the above outcomes

3. Adverse Event Record

Time period for collecting adverse events

AEs and SAEs will be collected from the time of informed consent throughout the treatment period, including the follow-up period (90 days after the last study drug administration).

Follow-up of unresolved adverse events

In the study, the investigator will follow up any AEs that the subject has not resolved in the last AE assessment based on medical indications, but no further records are required in the CRF.

4. Variables

The severity of AE is classified according to NCI CTCAE 5.0, using a 5-point scale (1-5 grades), and is reported in detail on CRF. The grades of AEs not included in CTCAE are as follows:

CTCAE grade	Equal:	definition
Level 1	Mild	Feel uncomfortable and do not interfere with normal activities of daily life.
Level 2	Moderate	Feeling of discomfort is sufficient to reduce or affect activities of daily living; treatment or medical intervention is not indicated, although these measures can improve the patient's overall health or symptoms.
Level 3	Severe	Inability to work or normal activities of daily life; treatment or medical intervention is required to improve the patient's overall health or symptoms; delay in treatment has no direct harmful effect on the patient's survival.
Level 4	Life-threatening or disabling	Directly endanger life or cause permanent mental or physical illness, affecting work or normal activities of daily life; treatment or medical intervention is required to maintain life.
Level 5	Fatal	AE causes death

5. Causality collection

The investigator will evaluate the causal relationship between the trial drug and each AE, and answer "yes" or "no" to the question "Do you think the event may be caused by the trial drug?"

For SAE, the causal relationship between other drugs and the research process will also be evaluated. Please note that for SAEs that may be related to any research process, causality is "yes".

For guidance on the interpretation of causality issues, please see Appendix A of the "Clinical Research Protocol".

6. Adverse events based on signs and symptoms

All AEs reported by the subject spontaneously or when answering open-ended

questions from the researcher: "Did you have any health problems since the last visit/last interview?" or found through observation. The information will be collected and recorded in the CRF. When collecting AEs, it is best to record the diagnosis, not the signs and symptoms. However, if the diagnosis result is known and there are other signs or symptoms that are not normally included in the diagnosis, the diagnosis and each sign or symptom will be recorded separately.

7. Adverse events based on inspections and trials

The results of laboratory tests and vital signs specified in the protocol will be summarized in the clinical study report. Therefore, compared to the baseline set by the protocol, if the deterioration meets any SAE criteria or the reason for discontinuation of the trial drug treatment, it should only be reported as an AE.

If the deterioration of laboratory test values/vital signs is related to clinical signs and symptoms, the signs or symptoms will be reported as an AE, and the related laboratory results/vital signs will be treated as other information. Investigators use clinical terms as much as possible for reporting, rather than laboratory terms (for example, anemia and low hemoglobin values). In the absence of clinical signs or symptoms, clinically relevant deterioration of non-mandatory parameters should be reported as an AE. Deterioration of laboratory test values clearly due to disease progression should not be reported as AE/SAE.

Compared with the baseline assessment, any new or aggravated clinically relevant abnormal medical results found during the physical examination will be reported as an AE.

8. Disease progression

Disease progression can be considered to be the deterioration of the subject's disease caused by the disease under investigation by the experimental drug. It may be an increase in the severity of the disease under study and/or an increase in disease symptoms. New metastasis or progression to existing metastases should be considered

disease progression, not AEs. Events that are clearly due to disease progression should not be reported as AEs during the study period.
The progression of the malignant tumor in the study, including the progression of signs and symptoms, should not be reported as a serious AE. Hospitalization due to signs and symptoms of disease progression should not be reported as a serious AE.

9. Report of serious adverse events

The investigator is responsible for notifying the ethics committee and sponsor of any SAE.
The investigator must inform the local ethics committee of any SAE within 24 hours.
All SAEs that lack important or relevant information should be followed up immediately. The investigator should inform the sponsor representative of any follow-up information of previously reported SAEs within 24 hours after learning.

Laboratory safety assessment

The laboratory safety assessment parameters are listed in the table:

Clinical chemistry	Hematology
Serum (S) / Plasma (P)-Albumin	Blood (B)-hemoglobin
S/P-ALT	B-leukocytes
S/P-AST	B-hematocrit
S/P-Alkaline Phosphatase	B-Red blood cell (RBC) count
S/P-total bilirubin	B-Absolute white blood cell classification count:
S/P-total calcium	Neutrophils
S/P-creatinine	Lymphocytes
S/P-glucose (fasting only on PK days)	Monocyte
S/P-Lactate Dehydrogenase (LDH) 2	Basophils
S/P-magnesium	Eosinophils
S/P-potassium	B-platelet count
S/P-Sodium	B-reticulocyte
S/P-urea nitrogen or blood urea nitrogen	Urinalysis
S/P-lipase	U-glucose
S/P-Amylase	U-protein
S/P-total cholesterol	U-blood
S/P-total triglycerides	Coagulation
S/P- High Density Lipoprotein	P-prothrombin time (PT)
S/P- Low Density Lipoprotein	P-activated partial thromboplastin time (APTT)
Thyroid function	P-thrombin time (TT)
S-Triiodothyronine (T3)	P-fibrinogen (Fbg)
S-thyroxine (T4)	P-D-dimer
S-TSH	P- INR
S-free T3	
S-free T4	

Blood and urine samples will be collected at the time specified in the study plan to determine clinical chemistry, hematology, thyroid function, coagulation function, and urinalysis. If the researcher believes that there are clinical indications, other safety samples can be collected. Acquisition date, time and results (value, unit and reference range) will be recorded in the appropriate CRF.

Clinical chemistry, hematology, thyroid function, coagulation function and urinalysis will be performed in Shanghai Chest Hospital, Shanghai Jiao Tong University School of Medicine.

In addition, during the screening visit, pregnancy tests are only performed on women with reproductive potential (according to the standard clinical practice of the research center, blood or urine tests are acceptable).

Researchers should evaluate existing results regarding clinically relevant abnormalities. Laboratory results should be signed and dated, and stored in the research center as the source data of laboratory variables.

Electrolyte abnormalities (hypokalemia, hypomagnesemia, hypocalcemia) must be corrected to within the normal range before the first dose, and electrolyte levels should be monitored during the study treatment.

Physical examination

A physical examination will be performed, including evaluation of the following: general appearance, skin, head and neck (including ears, eyes, nose, and throat), respiratory system, cardiovascular, abdomen, lymph nodes, thyroid, abdomen, and central nervous system.

Electrocardiogram

All patients will undergo a 12-lead digital electrocardiogram during the study visit. After the patient rests on the supine side for at least 10 minutes before the specified time, a 12-lead ECG will be obtained, which should be recorded at a speed of 25 mm/sec. All ECGs should be recorded in the same position of the patient. For each time point, three ECG results should be recorded in approximately 5 minutes. The

digital ECG records will be collected, analyzed and stored by the central ECG supplier. If there is an abnormality in the evaluation during the treatment when the study treatment is stopped, a 28-day follow-up evaluation is required to confirm the reversibility of the abnormality.

Echocardiography/MUGA Scan

Echocardiography or MUGA scans will be performed at screening (before the first treatment) and at least every 16 weeks throughout the treatment period to assess LVEF. The method of cardiac function assessment must be consistent within the patient (i.e., if echocardiography is used for screening assessment, echocardiography should also be used for subsequent scans). As far as possible, the same machine and operator should be used to examine patients and perform quantitative measurements. If there is an abnormality in the evaluation during the treatment when the study treatment is stopped, a 28-day follow-up evaluation is required to confirm the reversibility of the abnormality.

Vital signs

Pulse and BP

After resting for 10 minutes, BP and pulse rate in the supine position will be measured. As indicated in the research plan, evaluation will be performed at the time of the visit. In addition, if there are clinical indications, it should be at the discretion of the investigator. If applicable, any change in vital signs should be recorded as an AE.

Weight and height

Body weight assessment will be performed at the time of screening, and then on day 1 of each cycle and at the discontinuation visit. Evaluate height only during screening.
