# Supplementary material for: Coinhibition of the MEK/RTK pathway has high therapeutic efficacy in KRAS-mutant non-small cell lung cancer
Source: Signal Transduct Target Ther. 2025 Sep 12;10:299. doi: 10.1038/s41392-025-02382-w (PMC12426211; doi:10.1038/s41392-025-02382-w)

Supplementary Materials for

Co-inhibition of MEK/RTK pathways induces high therapeutic efficacy in KRAS- mutant non-small cell lung cancer

Jun Lu^1,2,3,4,11^, Minjuan Hu^1,11^, Yikai Zhao^5,11^, Tianqing Chu^1^, Wei Zhang^1^, Yijia Zhou^5,6^, Xinlei Cai^7^, Jun Wu^8^, Liang Hu^5^, Chunlei Shi^1^, Liwen Xiong^1^, Aiqin Gu^1^, Huimin Wang^1^, Yanwei Zhang^1^, Yuqing Lou^1^, Runbo Zhong^1^, Zhiqiang Gao^1^, Hongyu Liu^1^, Chao Zhou^1^, Yingli Wu^9^, Liang Zhu^10^, Hua Zhong^1,3,*^, Hongbin Ji^5,6,7,*^, Baohui Han^1,2,3,*^

Correspondence to: [18930858216@163.com](mailto:18930858216@163.com), [hbji@sibcb.ac.cn,](mailto:hbji@sibcb.ac.cn) [eddiedong8@hotmail.com](mailto:eddiedong8@hotmail.com)

**This file includes:**

Original films of western blots

1

Fig. 1k


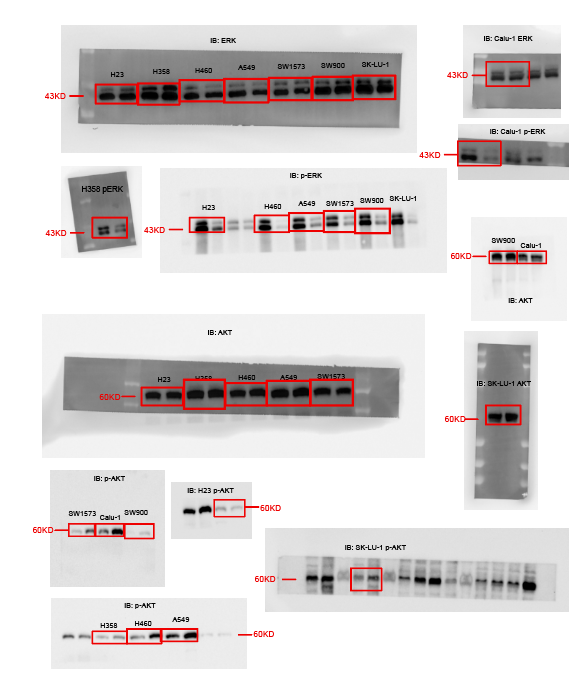


Fig. 2a


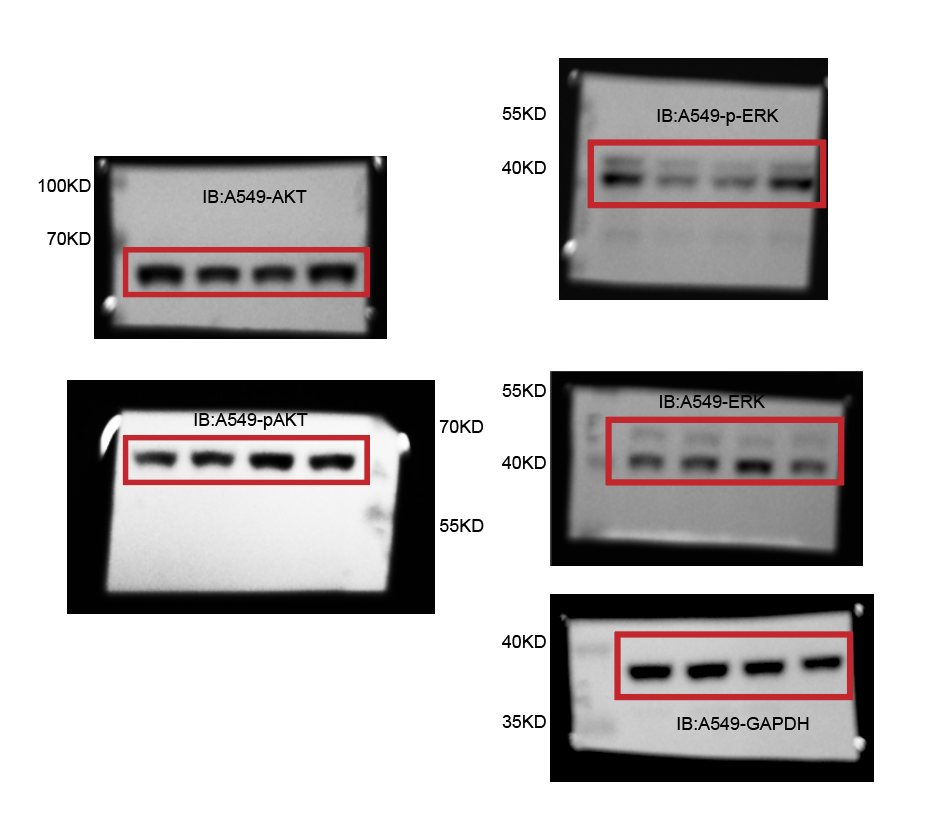


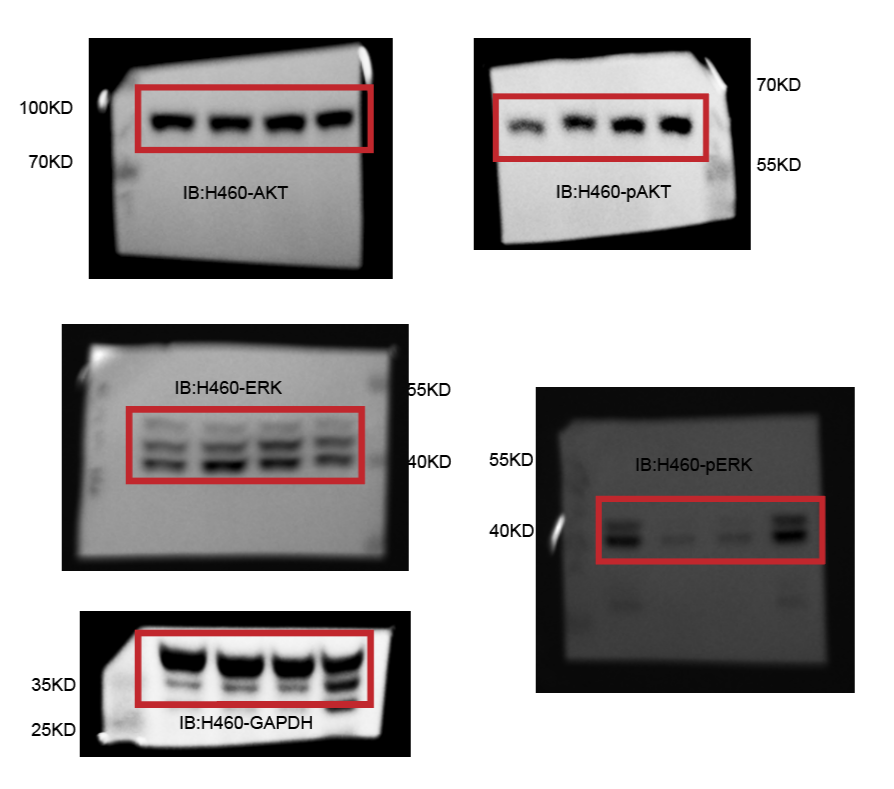


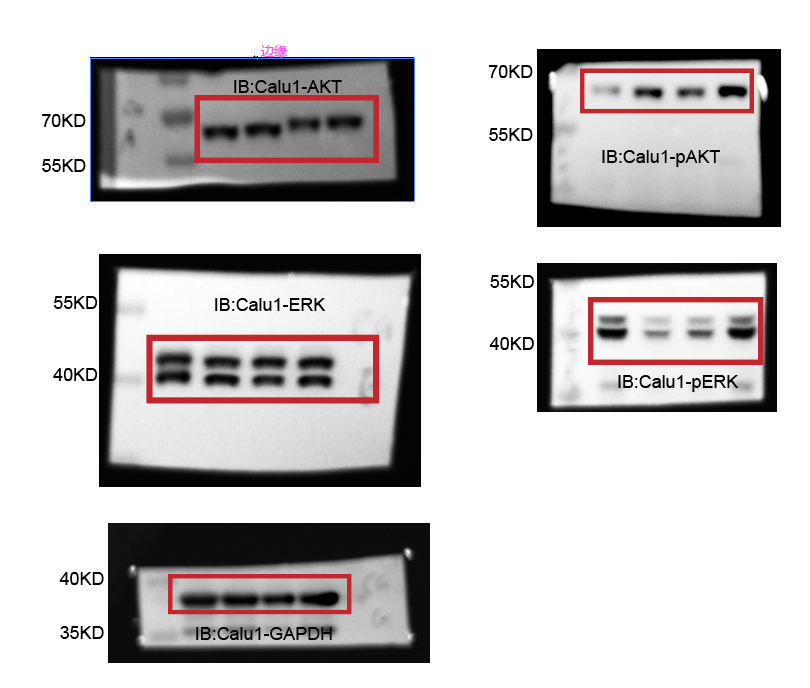


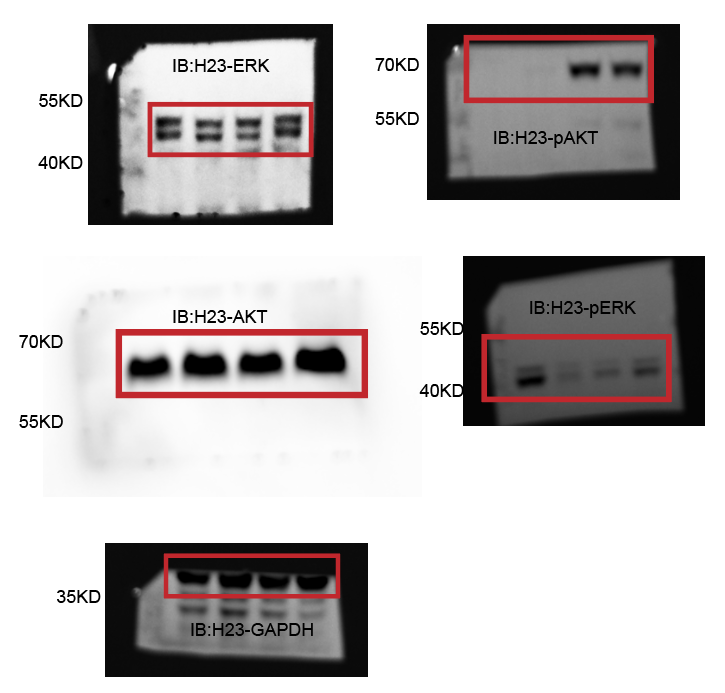


Fig. 2g


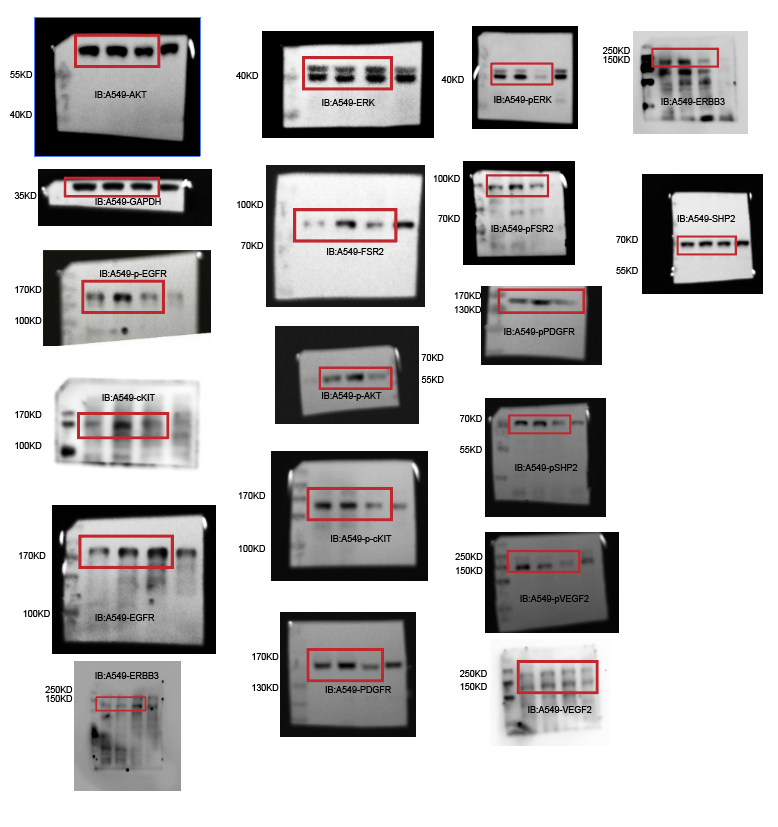


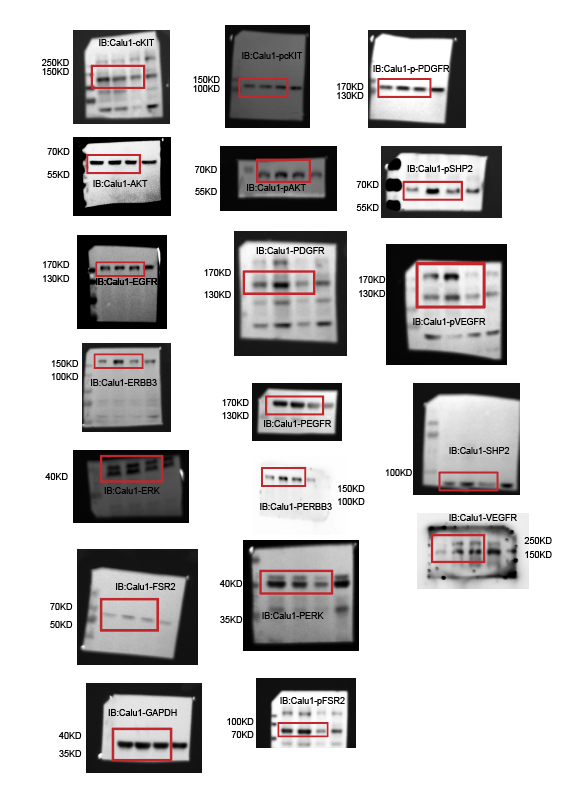


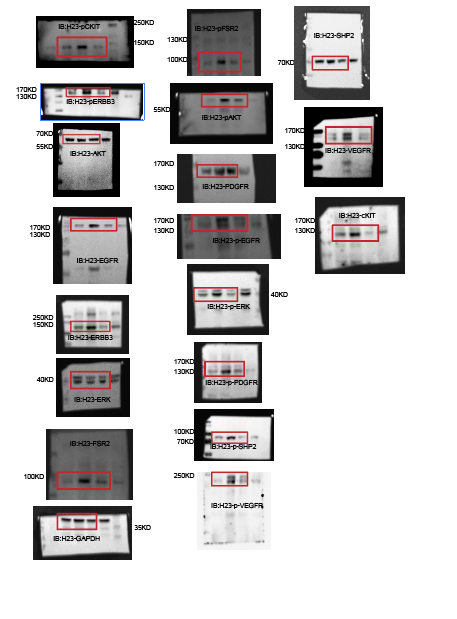


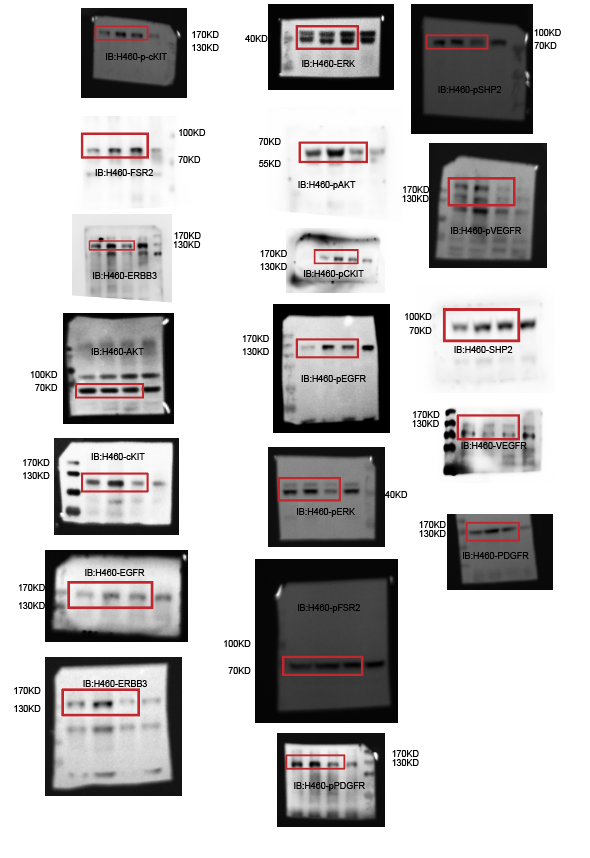


Fig. 4c


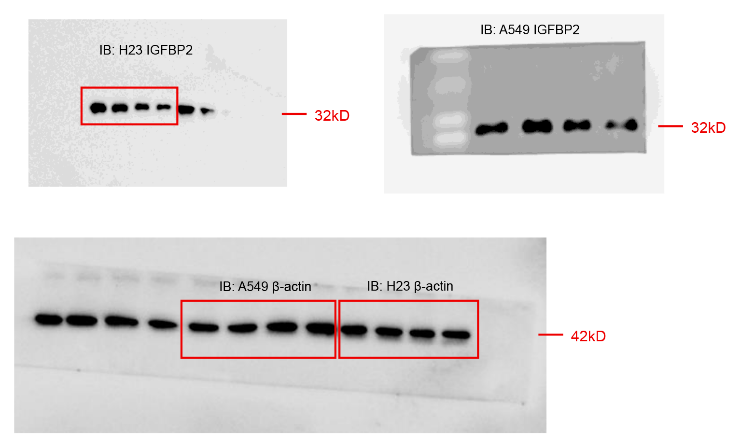


Fig. 4m


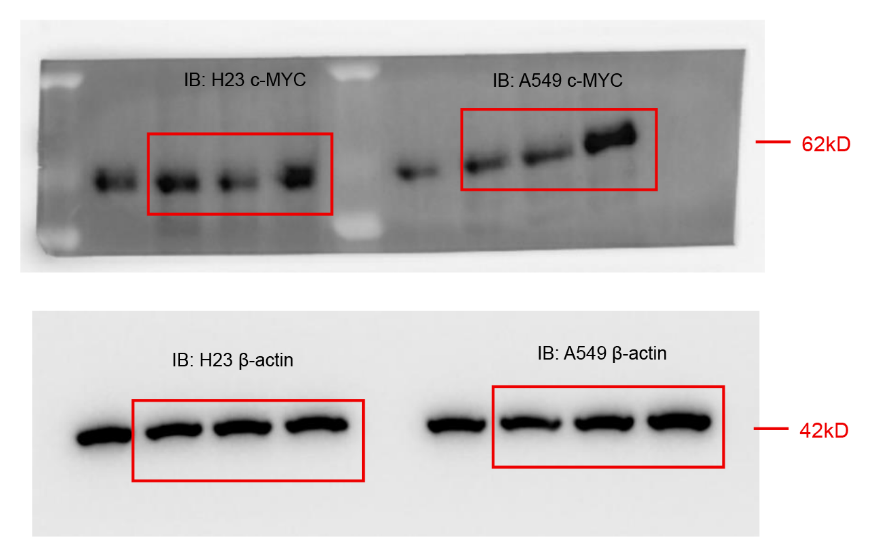


Fig. 4o


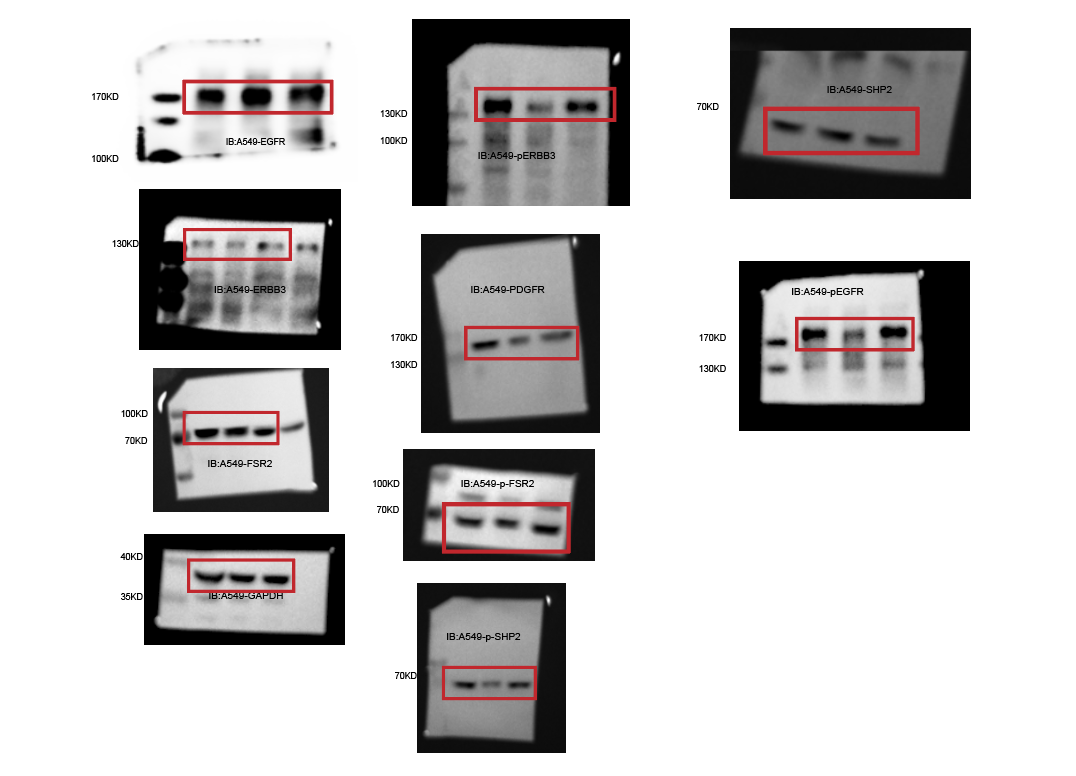


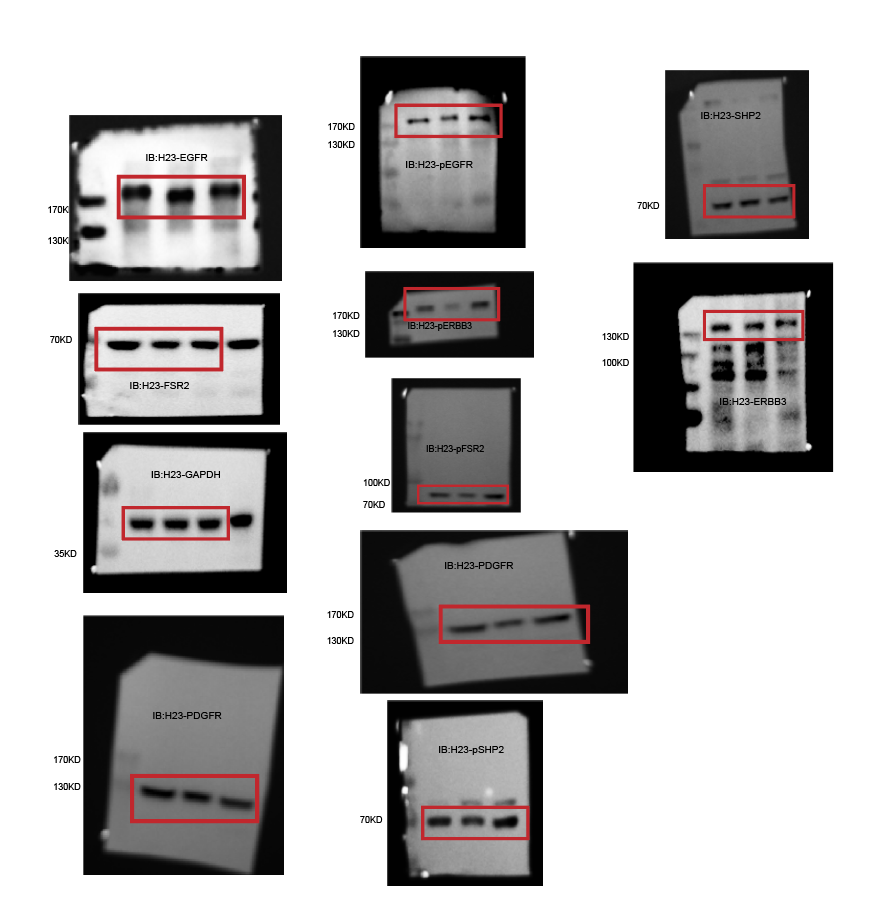


Fig. S4m


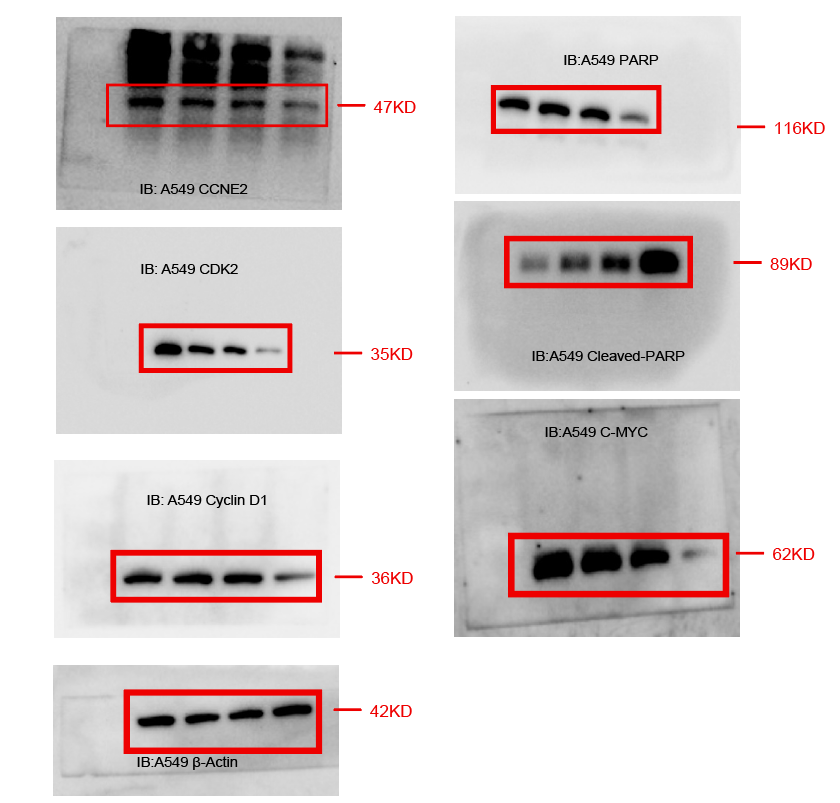

Supplement: Supplementary file 3 — Raw WB [file 41392_2025_2382_MOESM3_ESM.docx]
